# Author Correction: A chromatin-remodeling-independent role for ATRX in protecting centromeric cohesion

**DOI:** 10.1038/s44318-026-00866-1

**Published:** 2026-07-16

**Authors:** Lei Zhao, Xueying Yuan, Qinfu Chen, Haiyan Yan, Fangwei Wang

**Affiliations:** 1 Department of Gynecologic Oncology, Women’s Hospital, School of Medicine and MOE Laboratory of Biosystems Homeostasis & Protection, Life Sciences Institute, Zhejiang University, Hangzhou, China; 2 Zhejiang Key Laboratory of Molecular Cancer Biology, Life Sciences Institute, Zhejiang University, Hangzhou, China; 3 Zhejiang Key Laboratory of Geriatrics and Geriatrics Institute of Zhejiang Province, Affiliated Zhejiang Hospital, Zhejiang University School of Medicine, Hangzhou, China; 4 Zhejiang Key Laboratory of New Targets and Drugs for Nerve Injury Repair, School of Medicine, Hangzhou City University, Hangzhou, China; 5 State Key Laboratory of Transvascular Implantation Devices, Hangzhou, China

## Abstract

**Figure:**
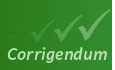

**Correction to:** *The EMBO Journal* (2025) 44:4037–4064. 10.1038/s44318-025-00465-6 | Published online 28 May 2025

The authors contacted the journal after identifying an error in the Acknowledgements section of the published article.

**The Acknowledgements section is corrected**.

The Acknowledgements section is corrected from:

This work was supported by … the National Natural Science Foundation of China (32270772 to HY, 32061160470 to FW, 32100583 to QC, and 324B2019 to LZ).

To:

This work was supported by … the National Natural Science Foundation of China (32270772 to HY, 32061160470 to FW, and 32100583 to QC).

Author statement:

An incorrect grant number was inadvertently included in the Acknowledgements section. This does not affect the conclusions of the study.

All authors agree to this correction.

