# Supplementary material for: A chromatin-remodeling-independent role for ATRX in protecting centromeric cohesion
Source: EMBO J. 2025 May 28;44(14):4037–64. doi: 10.1038/s44318-025-00465-6 (PMC12264150; doi:10.1038/s44318-025-00465-6)
Supplement: Supplementary file 1 — Appendix [file 44318_2025_465_MOESM1_ESM.pdf]

Appendix for:

A chromatin remodeling-independent role for ATRX in protecting centromeric cohesion

Lei Zhao[1, 2], Xueying Yuan[1, 3], Qinfu Chen[1], Haiyan Yan[4, *], Fangwei Wang[1, 3, 5, *]

[1] Department of Gynecologic Oncology, Women's Hospital, School of Medicine and MOE Laboratory of Biosystems Homeostasis & Protection, Life Sciences Institute, Zhejiang University, Hangzhou, China

[2] Zhejiang Key Laboratory of Molecular Cancer Biology, Life Sciences Institute, Zhejiang University, Hangzhou, China

[3] Zhejiang Key Laboratory of Geriatrics and Geriatrics Institute of Zhejiang Province, Affiliated Zhejiang Hospital, Zhejiang University School of Medicine, Hangzhou, China

[4] Zhejiang Key Laboratory of New Targets and Drugs for Nerve Injury Repair, School of Medicine, Hangzhou City University, Hangzhou, China

[5] State Key Laboratory of Transvascular Implantation Devices, Hangzhou, China

* Correspondence: fwwang@zju.edu.cn (F.W.), yanhy@hzcu.edu.cn (H.Y.).

Table of contents:

Appendix Figures

***Appendix Figure S1: Centromere-tethering of a Pds5B-binding fragment of ATRX is sufficient to protect centromeric cohesion in ATRX-depleted cells.* 2**

***Appendix Figure S2: ATRX does not interfere with Pds5B binding to Wapl and Sororin in interphase.* 3**

***Appendix Figure S3: ATRX protects sister chromatid cohesion independently of Haspin.* 4**

Appendix Figure S1

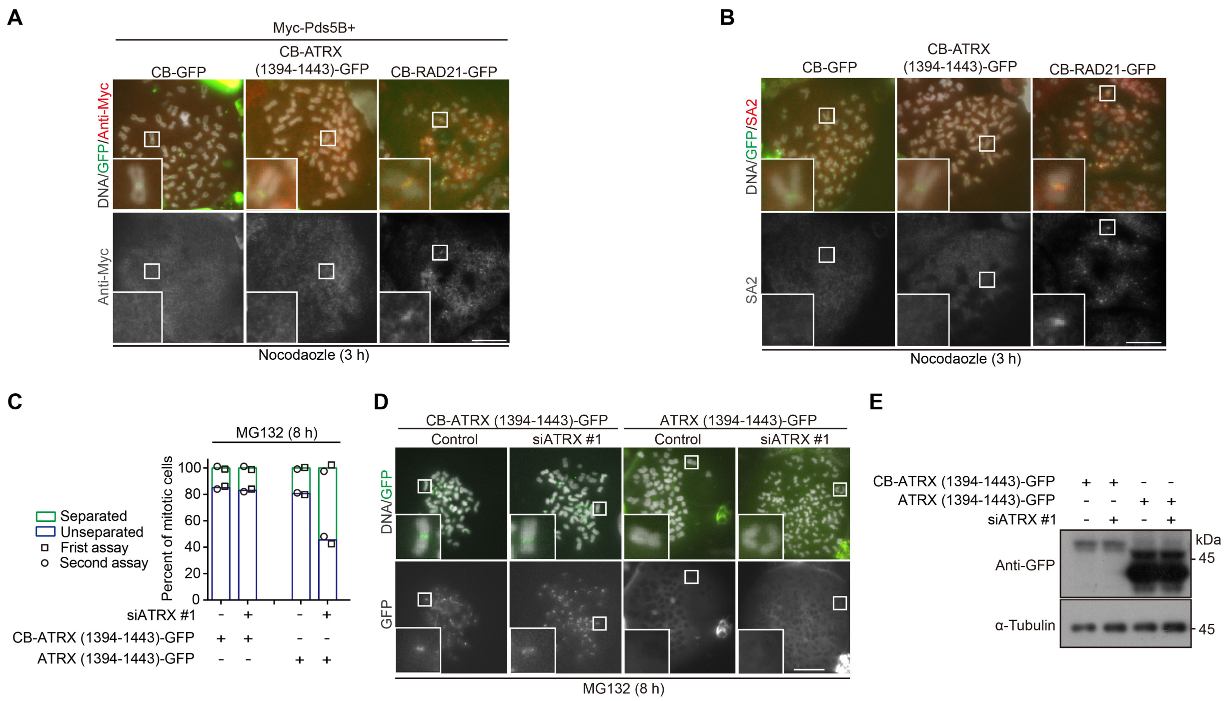

Appendix Figure S1: Centromere-tethering of a Pds5B-binding fragment of ATRX is sufficient to protect centromeric cohesion in ATRX-depleted cells.

(A) HeLa cells stably expressing Myc-Pds5B were transfected with plasmids encoding CB-GFP, CB-ATRX (1394-1443)-GFP, or CB-RAD21-GFP. Twenty-four hours post-transfection, cells were treated with nocodazole for 3 h, and mitotic chromosome spreads were stained for the Myc tag and DAPI. Representative images are shown.

(B) HeLa cells expressing CB-GFP, CB-ATRX (1394-1443)-GFP, or CB-RAD21-GFP were treated with nocodazole for 3 h, and mitotic chromosome spreads were stained for SA2 and DAPI. Representative images are shown.

(C-E) HeLa cells were transfected with control or ATRX siRNA and plasmids encoding CB-ATRX (1394-1443)-GFP or ATRX (1394-1443)-GFP, then treated with MG132 for 8 h, and mitotic chromosome spreads were stained with DAPI. The percentage of mitotic cells exhibiting predominantly separated or unseparated sister chromatids was quantified from more than 720 cells per condition across two independent experiments (C). Representative images are shown (D). Cell lysates were analyzed by immunoblotting for GFP and GAPDH (E).

Data information: Scale bars, 10 µm (A, B, D).

Appendix Figure S2

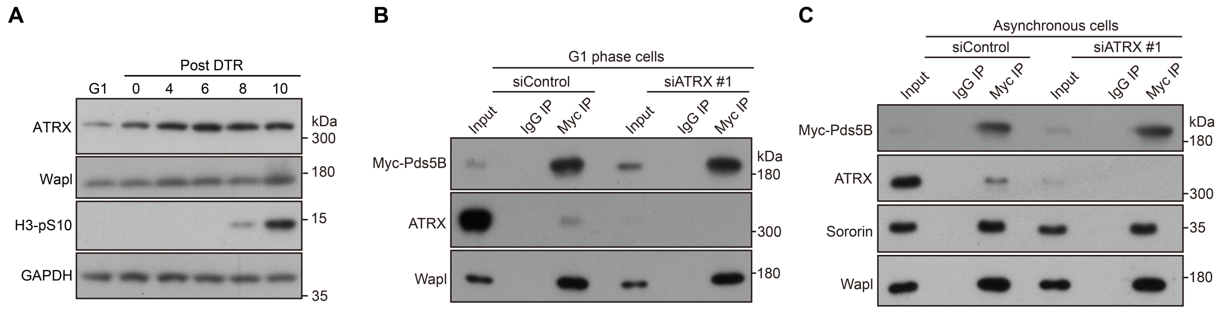

Appendix Figure S2: ATRX does not interfere with Pds5B binding to Wapl and Sororin in interphase.

(A) HeLa cells were synchronized in G1 phase following release from nocodazole-induced mitotic arrest. S-phase and G2-phase cells were collected at the indicated time points after release from double thymidine treatment. Cell lysates were analyzed by immunoblotting for ATRX, Wapl, histone H3 Ser10 phosphorylation (H3-pS10), and GAPDH.

(B) HeLa cells stably expressing Myc-Pds5B were transfected with control or ATRX siRNA and synchronized in G1 phase as described above. Cells were immunoprecipitated with anti-Myc beads or control IgG, followed by immunoblotting for the Myc tag, ATRX, and Wapl.

(C) HeLa cells stably expressing Myc-Pds5B were transfected with control or ATRX siRNA. Forty-eight hours post-transfection, asynchronous cells were immunoprecipitated with anti-Myc beads or control IgG, followed by immunoblotting for the Myc tag, ATRX, Sororin, and Wapl.

Appendix Figure S3

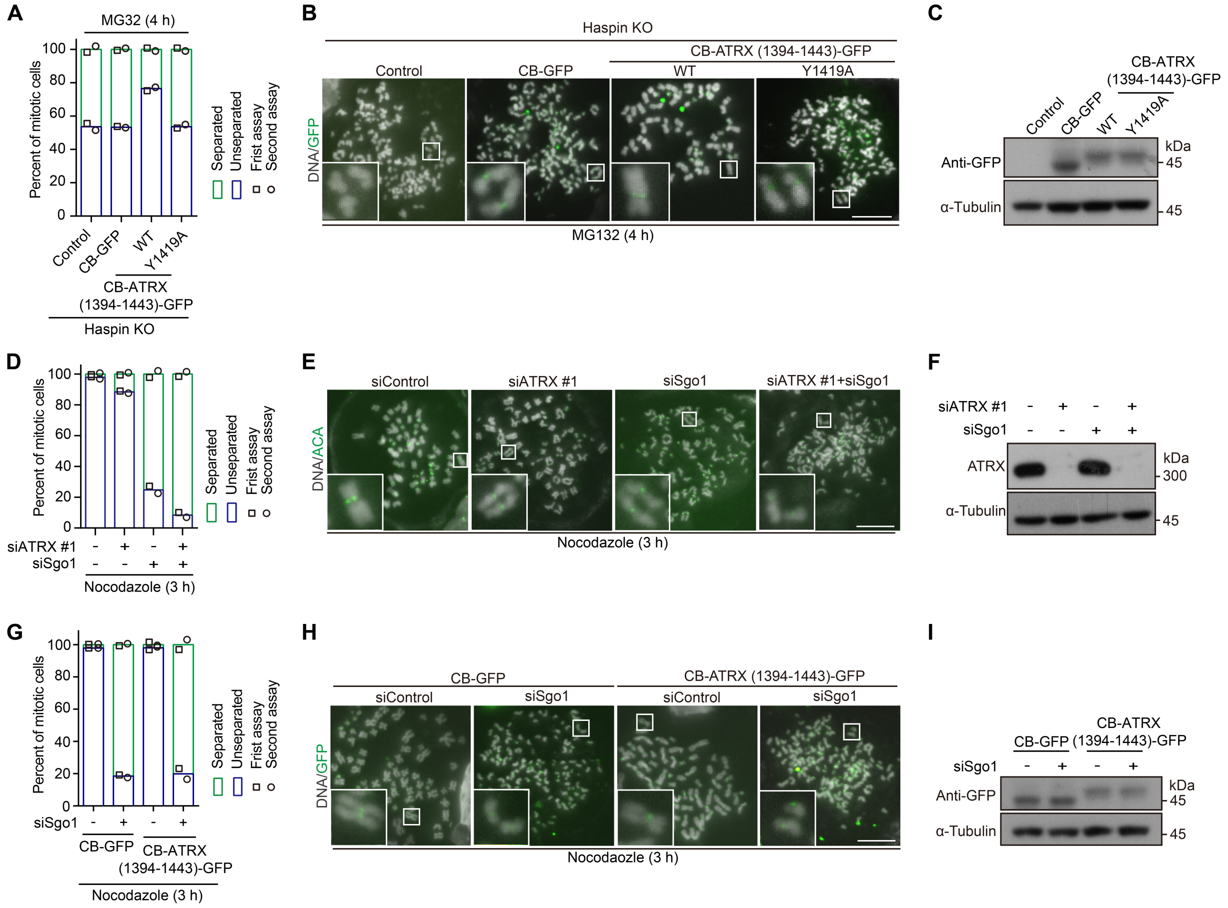

Appendix Figure S3: ATRX protects sister chromatid cohesion independently of Haspin.

(A-C) Haspin KO cells expressing CB-GFP or CB-ATRX (1394-1443)-GFP (WT or Y1419A) were treated with MG132 for 4 h, and mitotic chromosome spreads were stained for DAPI. The percentage of mitotic cells exhibiting predominantly separated or unseparated sister chromatids was quantified from more than 640 cells per condition across two independent experiments (A). Representative images are shown (B). Cell lysates were analyzed by immunoblotting for GFP and α-Tubulin (C).

(D-F) HeLa cells were transfected with control siRNA, ATRX siRNA, and/or Sgo1 siRNA. Forty-eight hours post-transfection, cells were treated with nocodazole for 3 h, and mitotic chromosome spreads were stained for ACA and DAPI. The percentage of mitotic cells exhibiting predominantly separated or unseparated sister chromatids was quantified from more than 620 cells per condition across two independent experiments (D). Representative images are shown (E). Cell lysates were analyzed by immunoblotting for ATRX and α-Tubulin (F).

(G-I) HeLa cells were transfected with control or Sgo1 siRNA and plasmids encoding CB-GFP or CB-ATRX (1394-1443)-GFP. Cells were treated with nocodazole for 3 h, and mitotic chromosome spreads were stained for DAPI. The percentage of mitotic cells exhibiting predominantly separated or unseparated sister chromatids was quantified from more than 620 cells per condition across two independent experiments (G). Representative images are shown (H). Cell lysates were analyzed by immunoblotting for GFP and α-Tubulin (I).

Data information: Scale bars, 10 µm (B, E, and H).
