# Supplementary material for: A chromatin-remodeling-independent role for ATRX in protecting centromeric cohesion
Source: EMBO J. 2025 May 28;44(14):4037–64. doi: 10.1038/s44318-025-00465-6 (PMC12264150; doi:10.1038/s44318-025-00465-6)
Supplement: Supplementary file 5 — Movie EV1 [file 44318_2025_465_MOESM5_ESM.zip › EMBOJ-2025-120195R_Movie EV1-Source data/EMBOJ-2025-120195R_-Legend for Movie EV1.docx]

**Figure legend for Movie EV1.**

**Movie EV1 (Related to Figs. 3D and EV3A).** Live imaging of control HeLa cells expressing H2B-GFP, during the release from STLC into MG132.
