# Supplementary figures and images for: A chromatin-remodeling-independent role for ATRX in protecting centromeric cohesion (part 1 of 3)
Source: EMBO J. 2025 May 28;44(14):4037–64. doi: 10.1038/s44318-025-00465-6 (PMC12264150; doi:10.1038/s44318-025-00465-6)

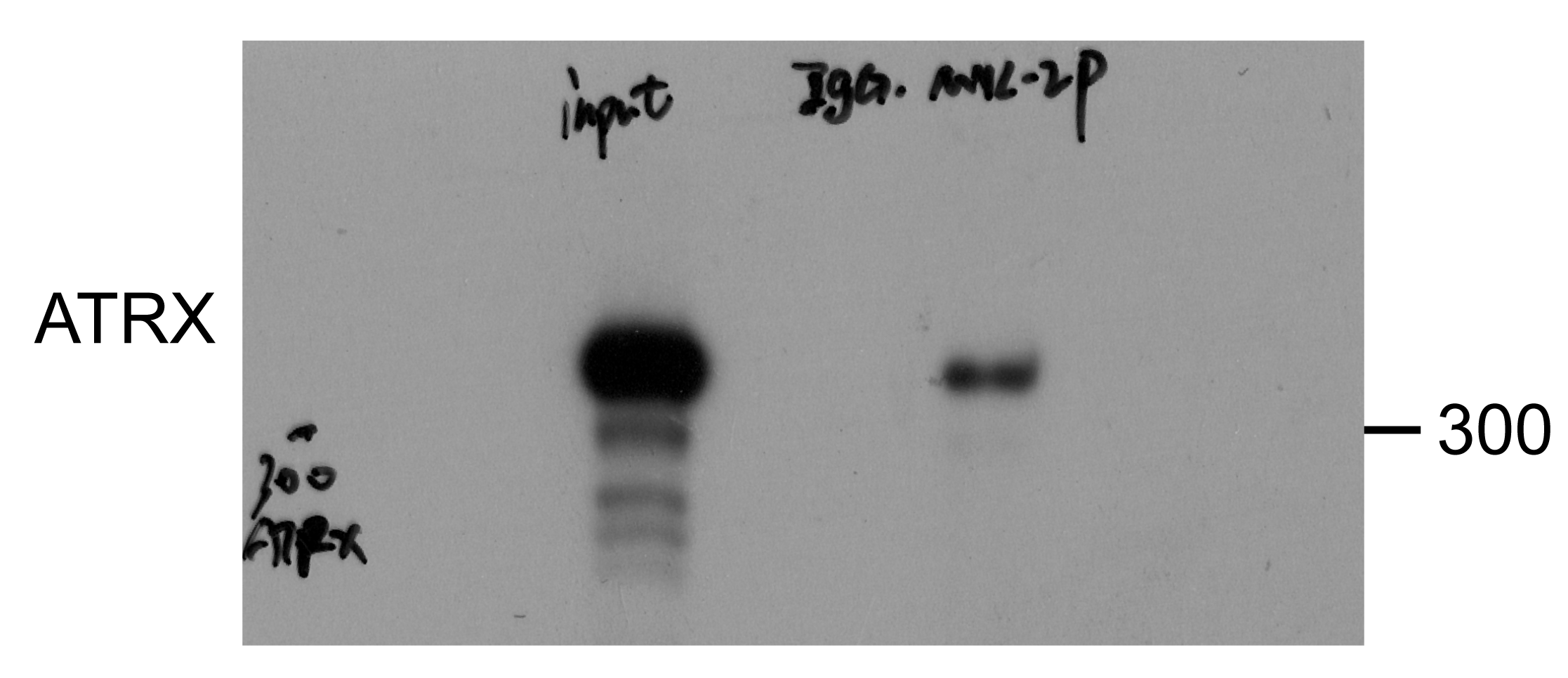

Supplement: Supplementary file 7 — Source data Fig. 1 [file 44318_2025_465_MOESM7_ESM.zip › EMBOJ-2025-120195-Figure 1-Source data/Figure 1/1B/western ATRX.tif]

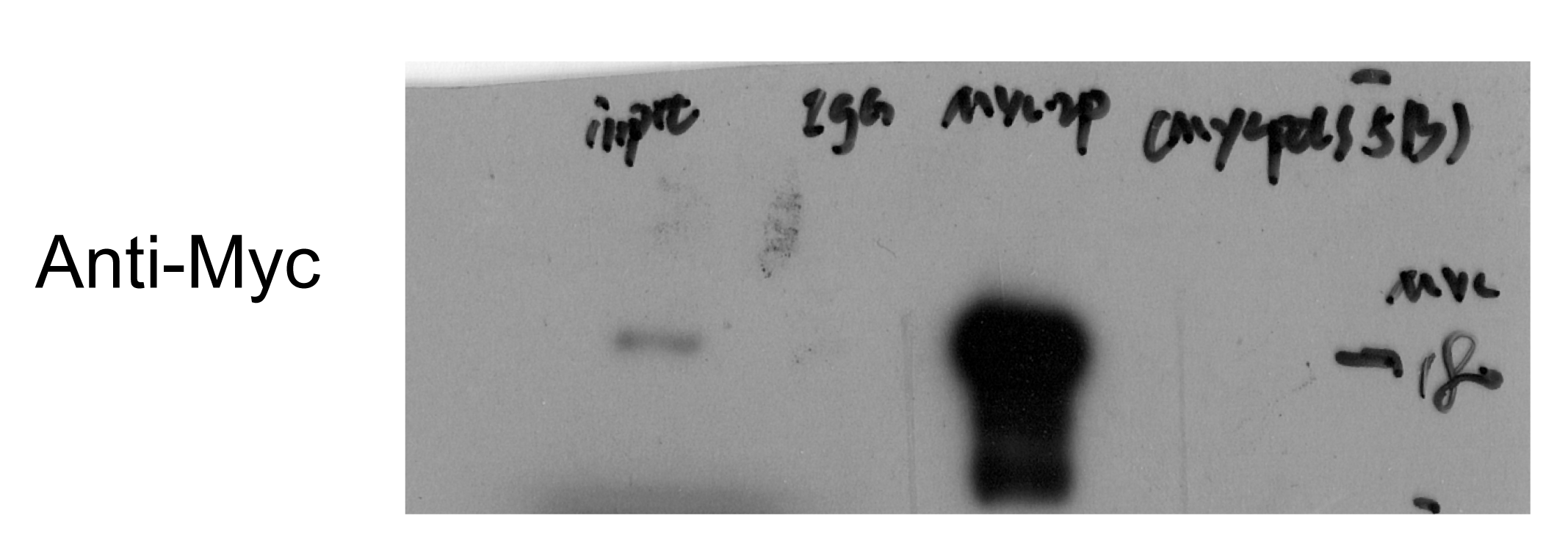

Supplement: Supplementary file 7 — Source data Fig. 1 [file 44318_2025_465_MOESM7_ESM.zip › EMBOJ-2025-120195-Figure 1-Source data/Figure 1/1B/western Myc.tif]

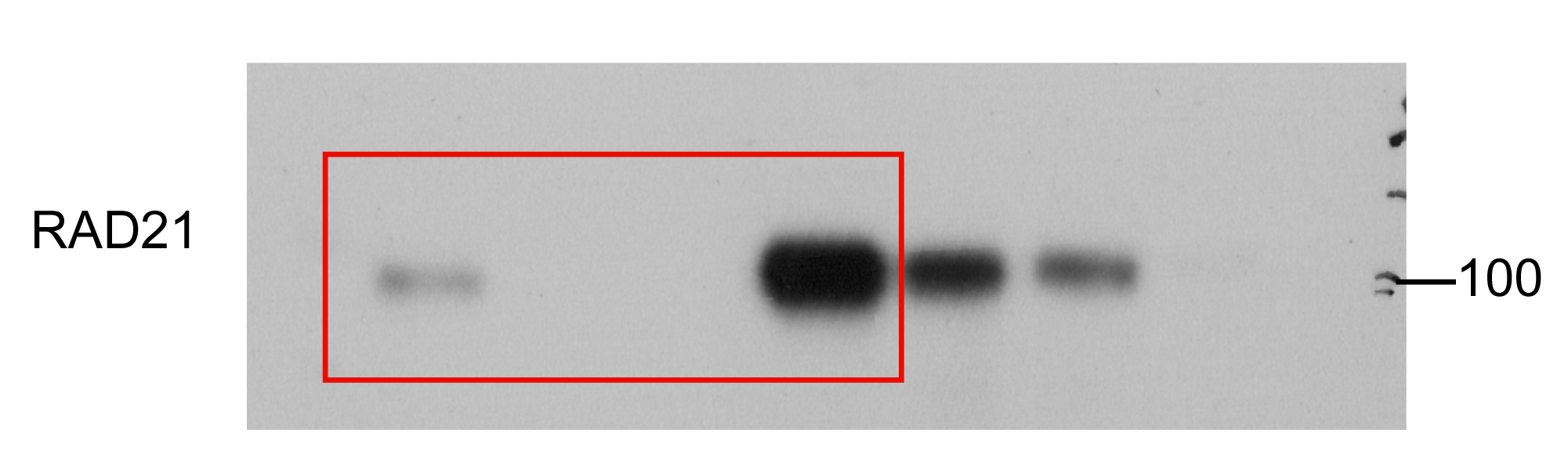

Supplement: Supplementary file 7 — Source data Fig. 1 [file 44318_2025_465_MOESM7_ESM.zip › EMBOJ-2025-120195-Figure 1-Source data/Figure 1/1B/western RAD21.tif]

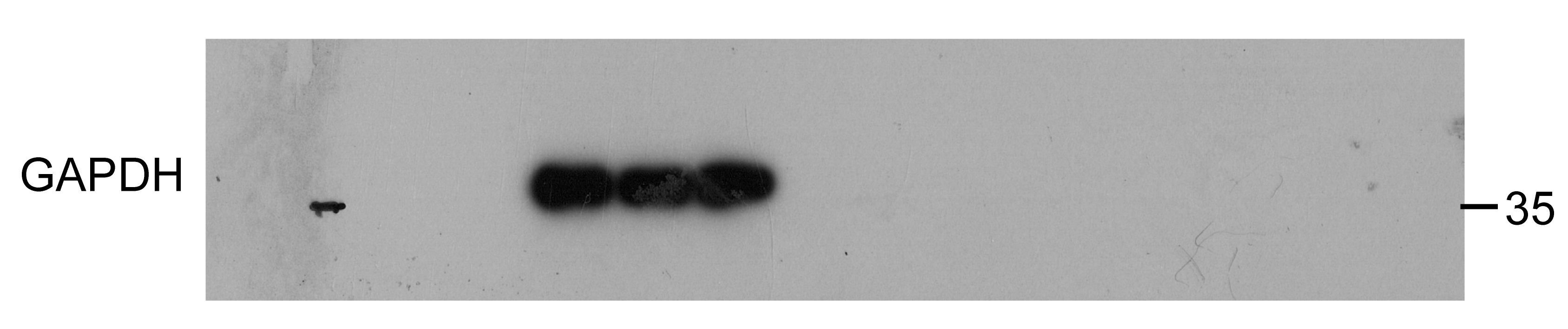

Supplement: Supplementary file 7 — Source data Fig. 1 [file 44318_2025_465_MOESM7_ESM.zip › EMBOJ-2025-120195-Figure 1-Source data/Figure 1/1C/western GAPDH.tif]

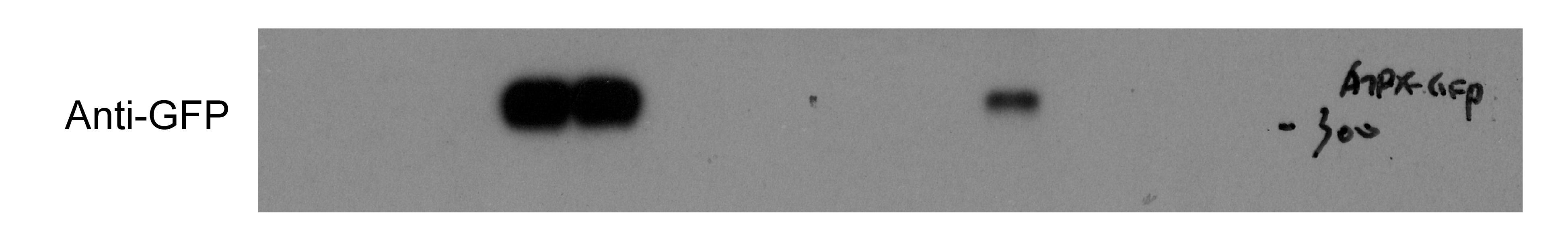

Supplement: Supplementary file 7 — Source data Fig. 1 [file 44318_2025_465_MOESM7_ESM.zip › EMBOJ-2025-120195-Figure 1-Source data/Figure 1/1C/western GFP.tif]

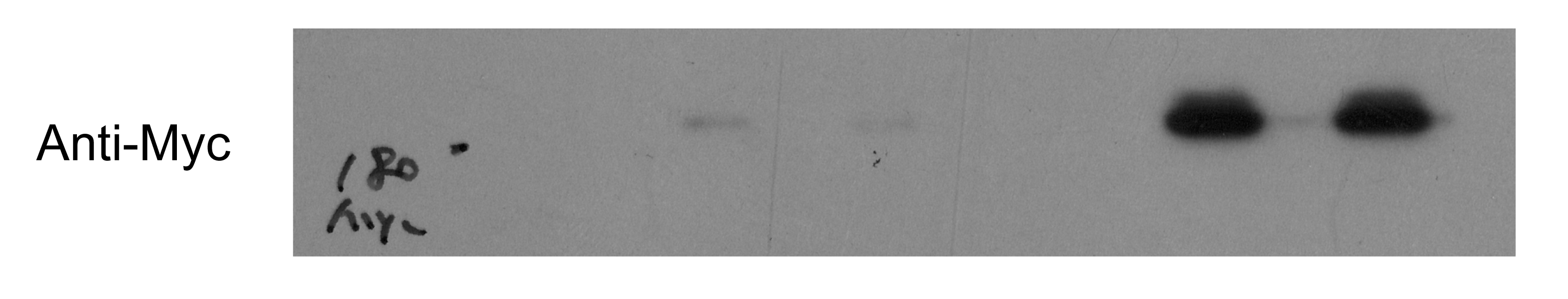

Supplement: Supplementary file 7 — Source data Fig. 1 [file 44318_2025_465_MOESM7_ESM.zip › EMBOJ-2025-120195-Figure 1-Source data/Figure 1/1C/western Myc.tif]

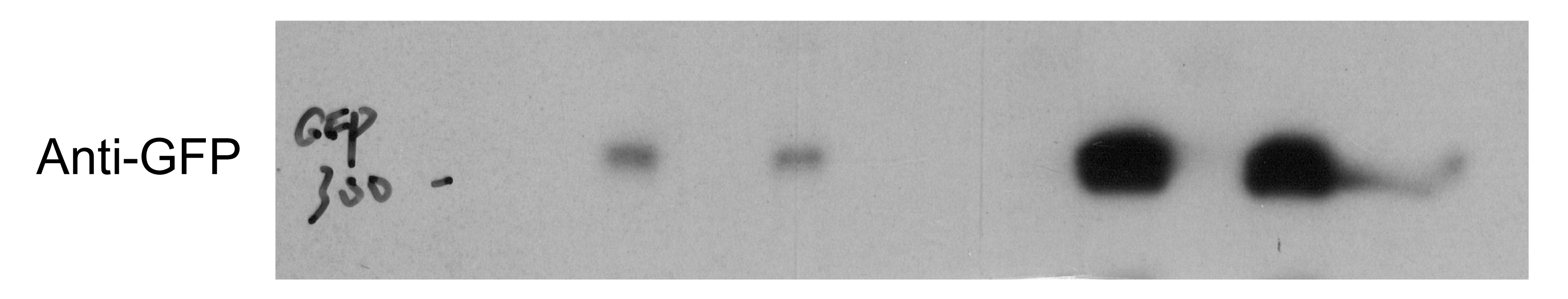

Supplement: Supplementary file 7 — Source data Fig. 1 [file 44318_2025_465_MOESM7_ESM.zip › EMBOJ-2025-120195-Figure 1-Source data/Figure 1/1D/western GFP.tif]

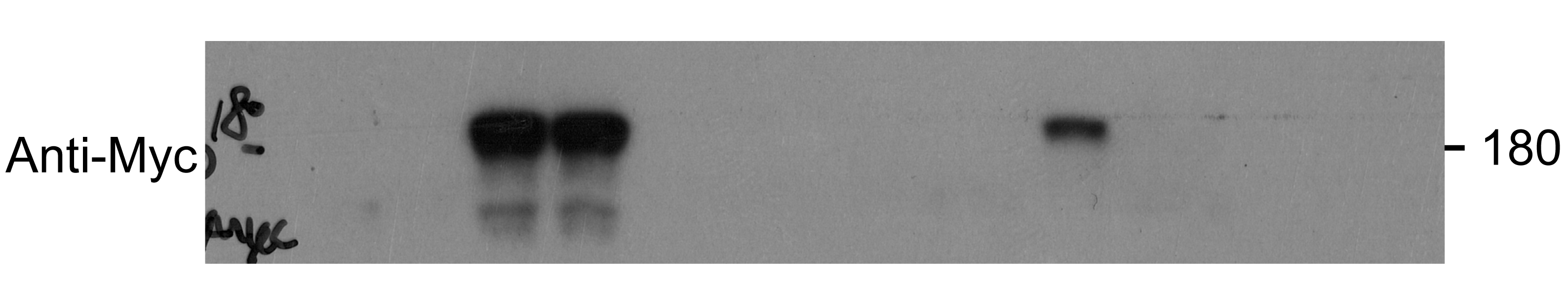

Supplement: Supplementary file 7 — Source data Fig. 1 [file 44318_2025_465_MOESM7_ESM.zip › EMBOJ-2025-120195-Figure 1-Source data/Figure 1/1D/western Myc.tif]

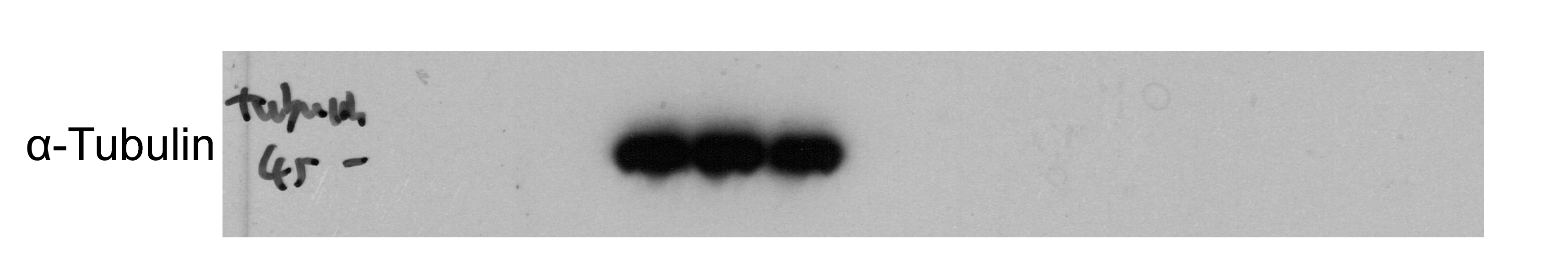

Supplement: Supplementary file 7 — Source data Fig. 1 [file 44318_2025_465_MOESM7_ESM.zip › EMBOJ-2025-120195-Figure 1-Source data/Figure 1/1D/western α-Tubulin.tif]

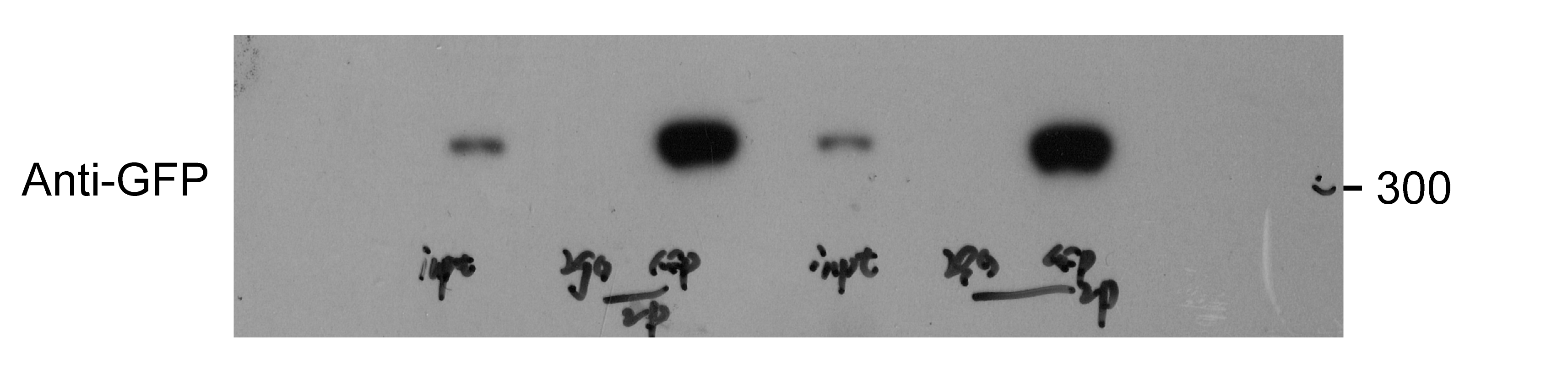

Supplement: Supplementary file 7 — Source data Fig. 1 [file 44318_2025_465_MOESM7_ESM.zip › EMBOJ-2025-120195-Figure 1-Source data/Figure 1/1E/western GFP.tif]

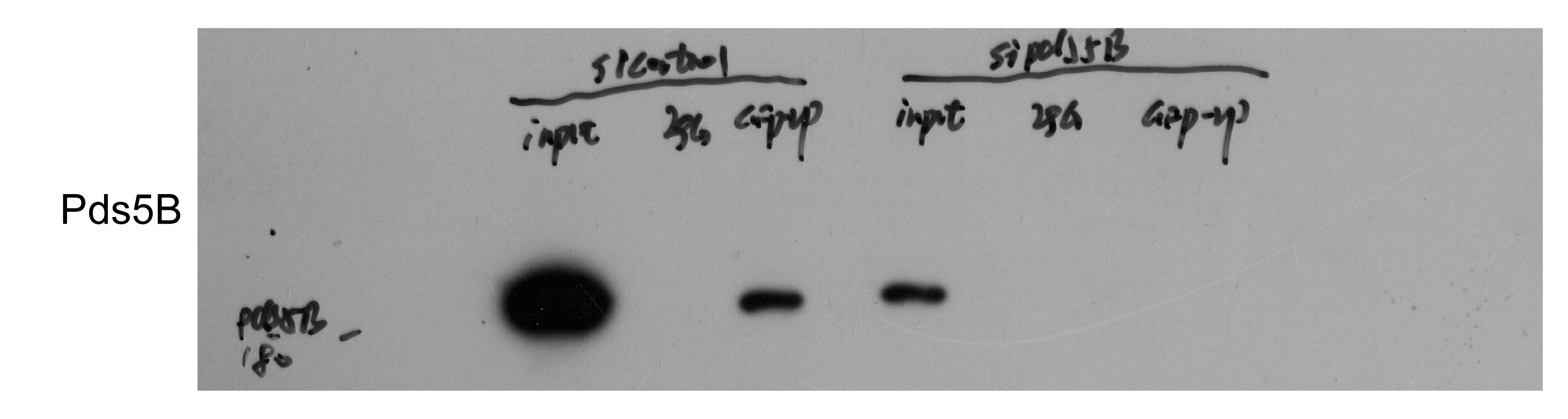

Supplement: Supplementary file 7 — Source data Fig. 1 [file 44318_2025_465_MOESM7_ESM.zip › EMBOJ-2025-120195-Figure 1-Source data/Figure 1/1E/western Pds5B.tif]

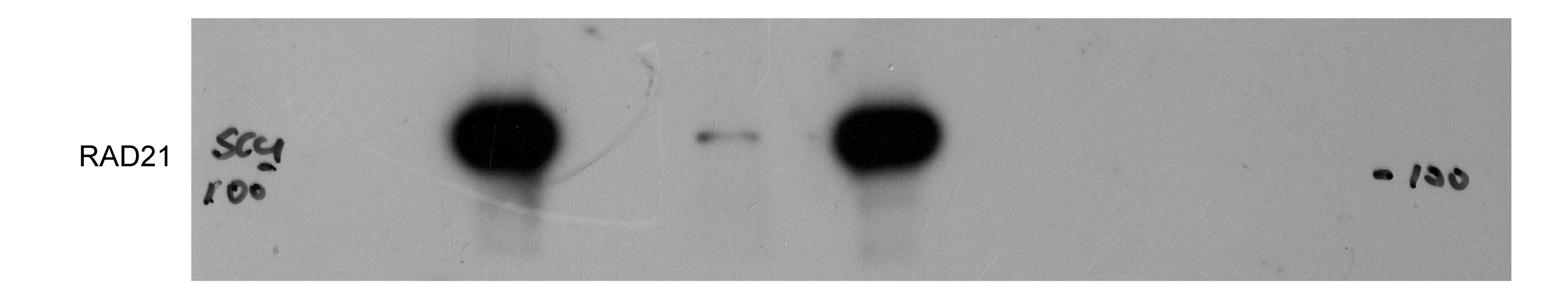

Supplement: Supplementary file 7 — Source data Fig. 1 [file 44318_2025_465_MOESM7_ESM.zip › EMBOJ-2025-120195-Figure 1-Source data/Figure 1/1E/western RAD21.tif]

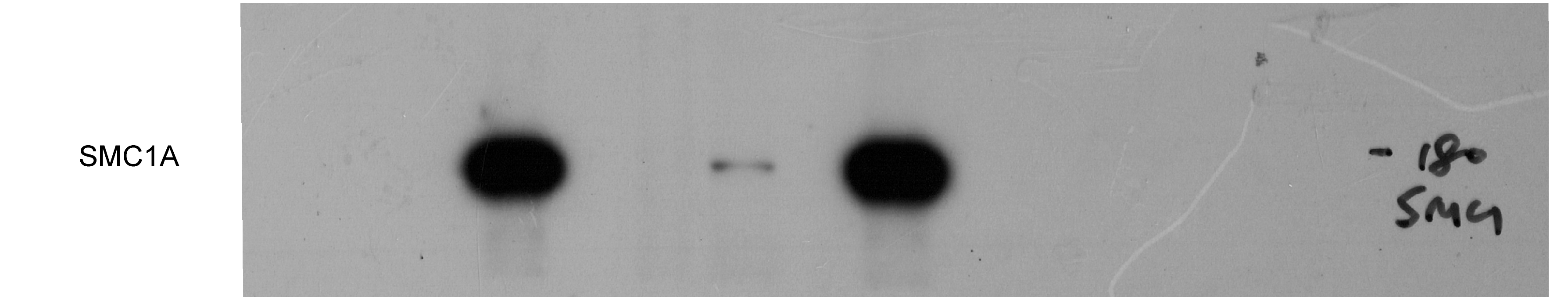

Supplement: Supplementary file 7 — Source data Fig. 1 [file 44318_2025_465_MOESM7_ESM.zip › EMBOJ-2025-120195-Figure 1-Source data/Figure 1/1E/western SMC1A.tif]

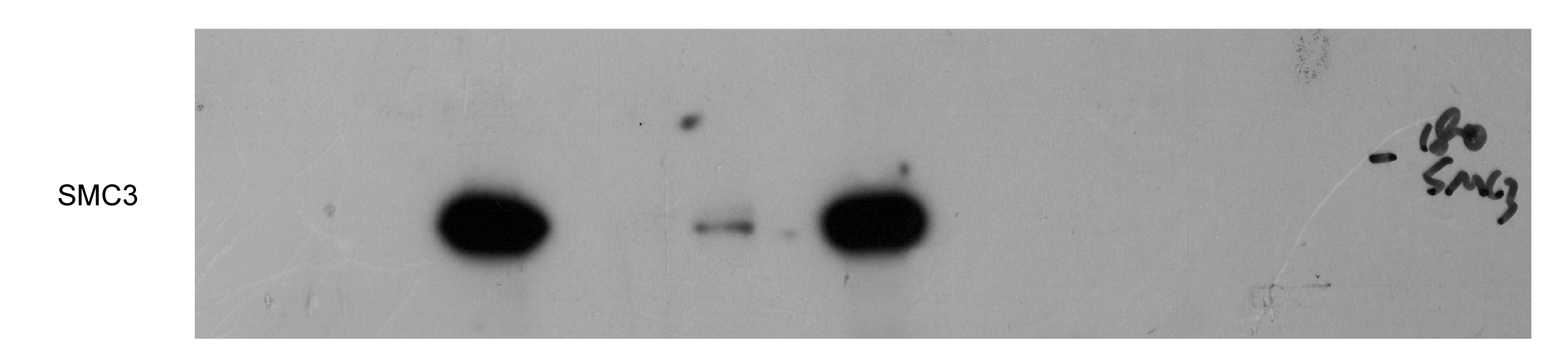

Supplement: Supplementary file 7 — Source data Fig. 1 [file 44318_2025_465_MOESM7_ESM.zip › EMBOJ-2025-120195-Figure 1-Source data/Figure 1/1E/western SMC3.tif]

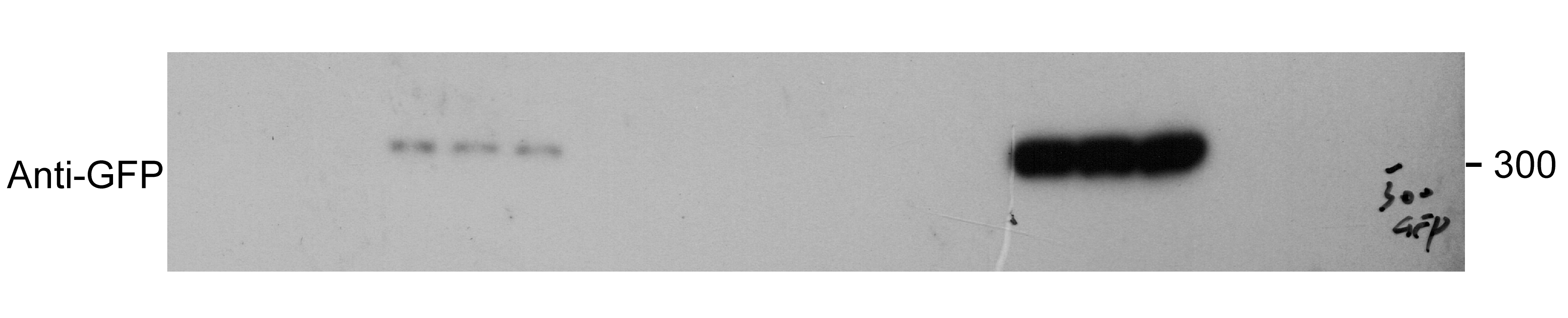

Supplement: Supplementary file 7 — Source data Fig. 1 [file 44318_2025_465_MOESM7_ESM.zip › EMBOJ-2025-120195-Figure 1-Source data/Figure 1/1F/western GFP.tif]

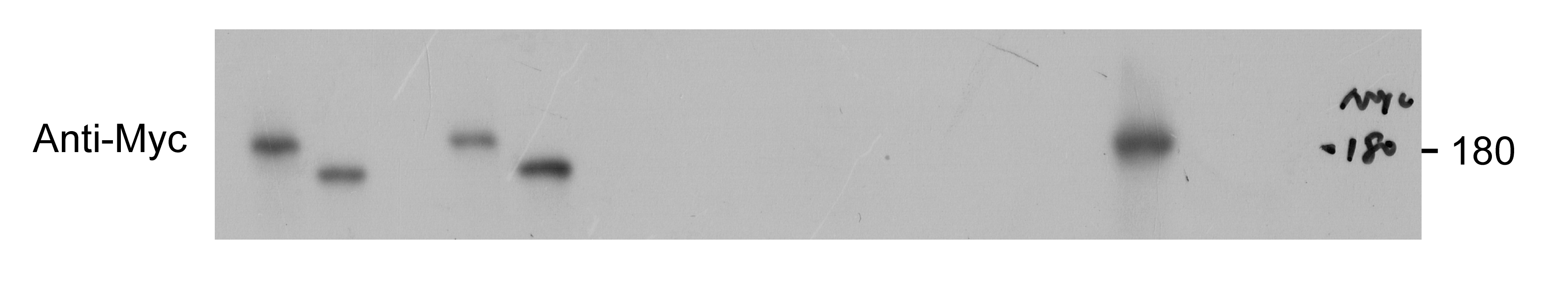

Supplement: Supplementary file 7 — Source data Fig. 1 [file 44318_2025_465_MOESM7_ESM.zip › EMBOJ-2025-120195-Figure 1-Source data/Figure 1/1F/western Myc.tif]

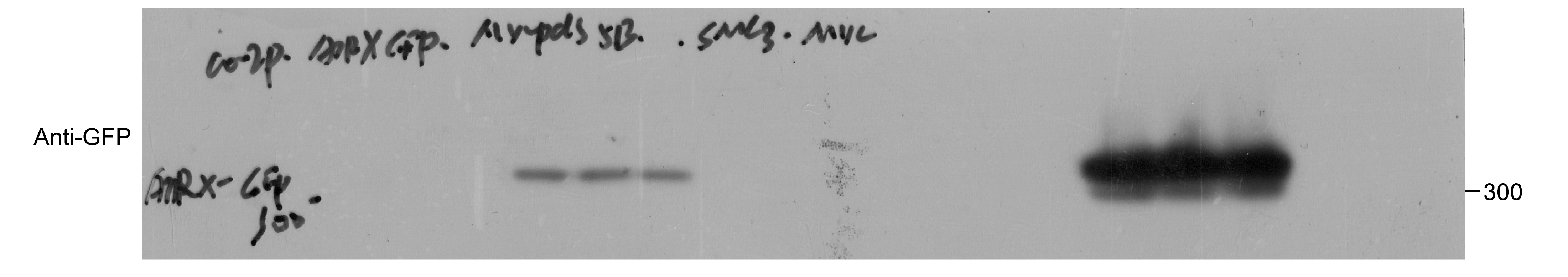

Supplement: Supplementary file 7 — Source data Fig. 1 [file 44318_2025_465_MOESM7_ESM.zip › EMBOJ-2025-120195-Figure 1-Source data/Figure 1/1G/western GFP.tif]

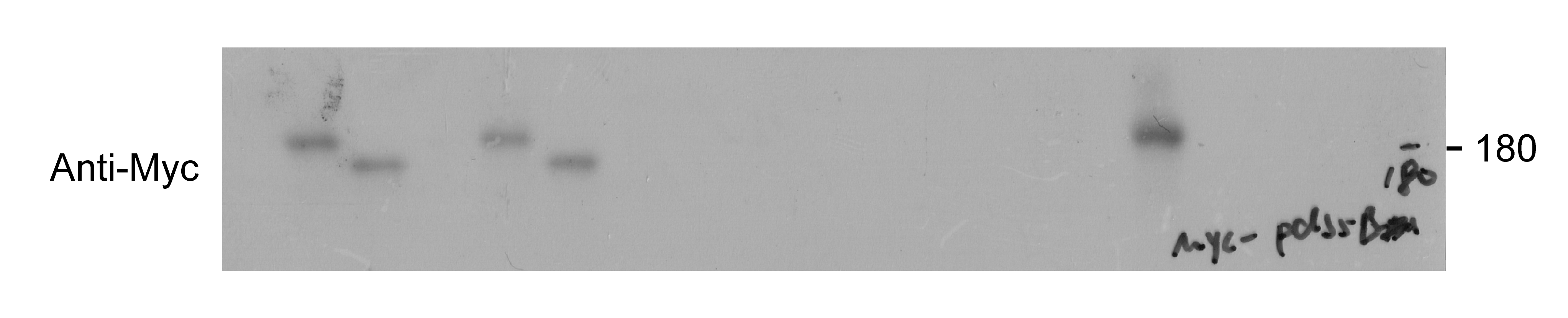

Supplement: Supplementary file 7 — Source data Fig. 1 [file 44318_2025_465_MOESM7_ESM.zip › EMBOJ-2025-120195-Figure 1-Source data/Figure 1/1G/western Myc.tif]

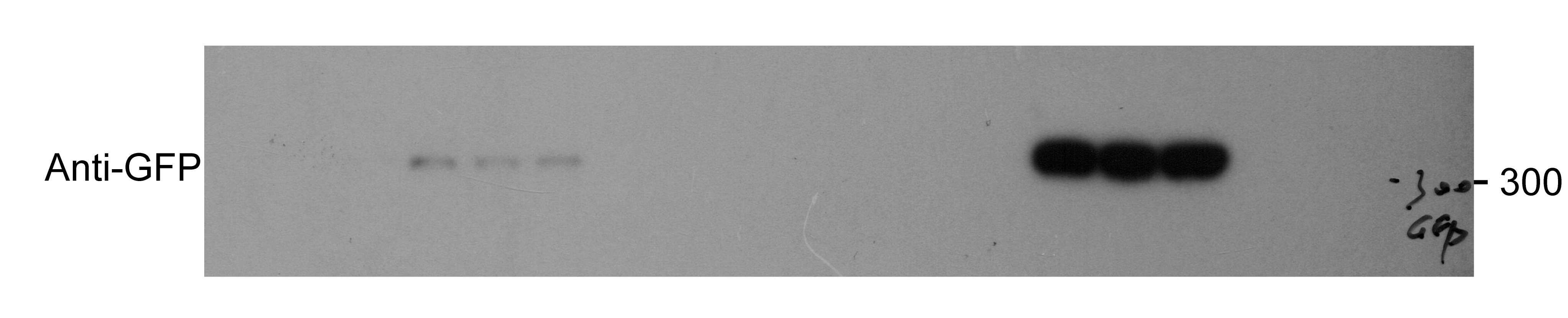

Supplement: Supplementary file 7 — Source data Fig. 1 [file 44318_2025_465_MOESM7_ESM.zip › EMBOJ-2025-120195-Figure 1-Source data/Figure 1/1H/western GFP.tif]

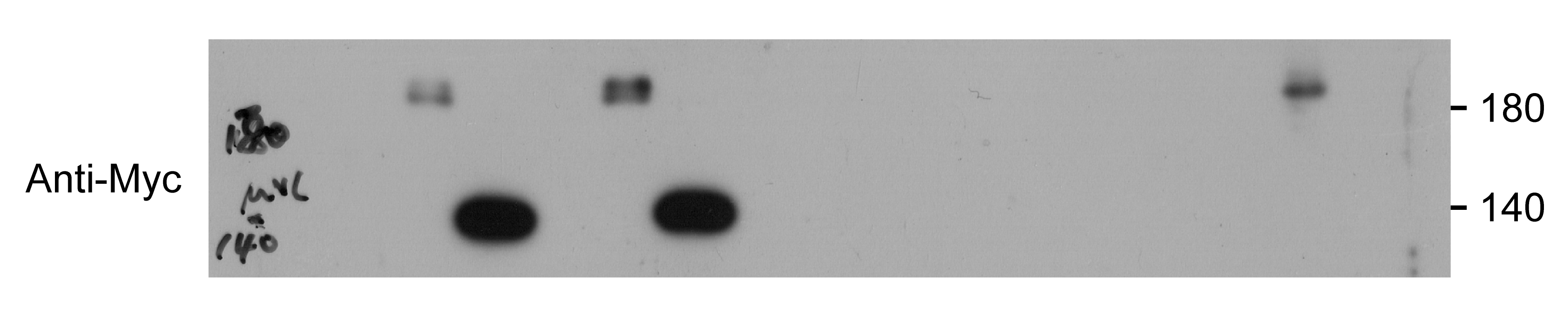

Supplement: Supplementary file 7 — Source data Fig. 1 [file 44318_2025_465_MOESM7_ESM.zip › EMBOJ-2025-120195-Figure 1-Source data/Figure 1/1H/western Myc.tif]

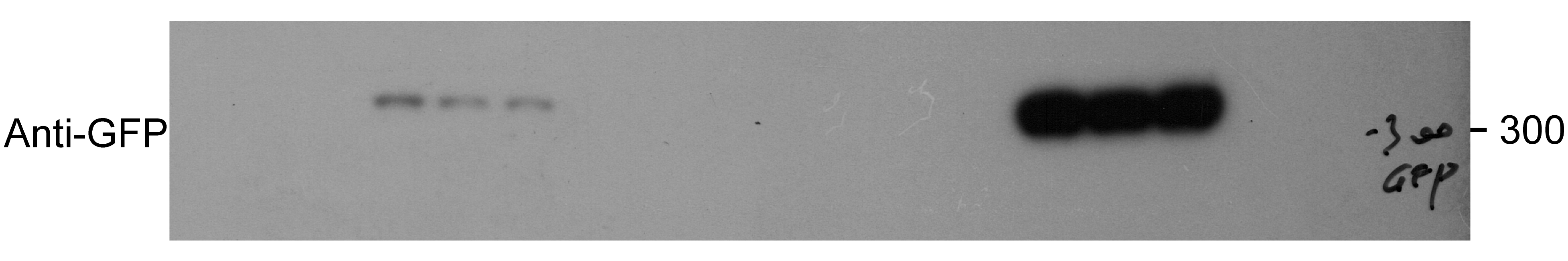

Supplement: Supplementary file 7 — Source data Fig. 1 [file 44318_2025_465_MOESM7_ESM.zip › EMBOJ-2025-120195-Figure 1-Source data/Figure 1/1I/western GFP.tif]

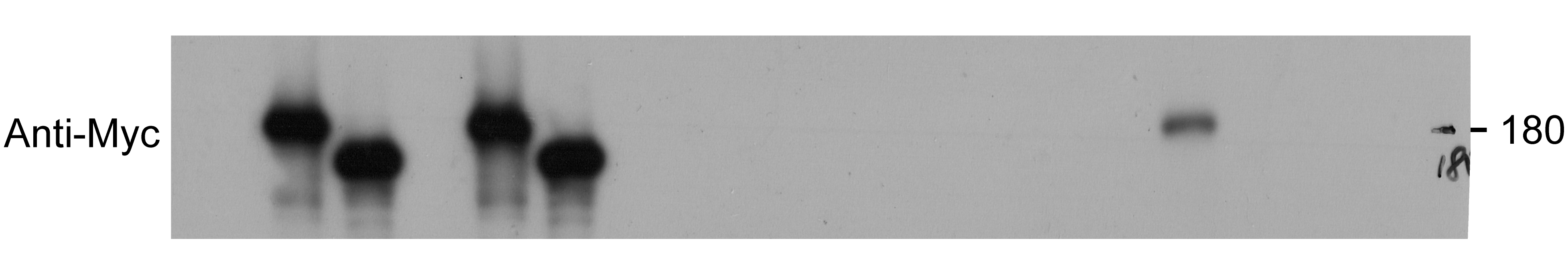

Supplement: Supplementary file 7 — Source data Fig. 1 [file 44318_2025_465_MOESM7_ESM.zip › EMBOJ-2025-120195-Figure 1-Source data/Figure 1/1I/western Myc.tif]

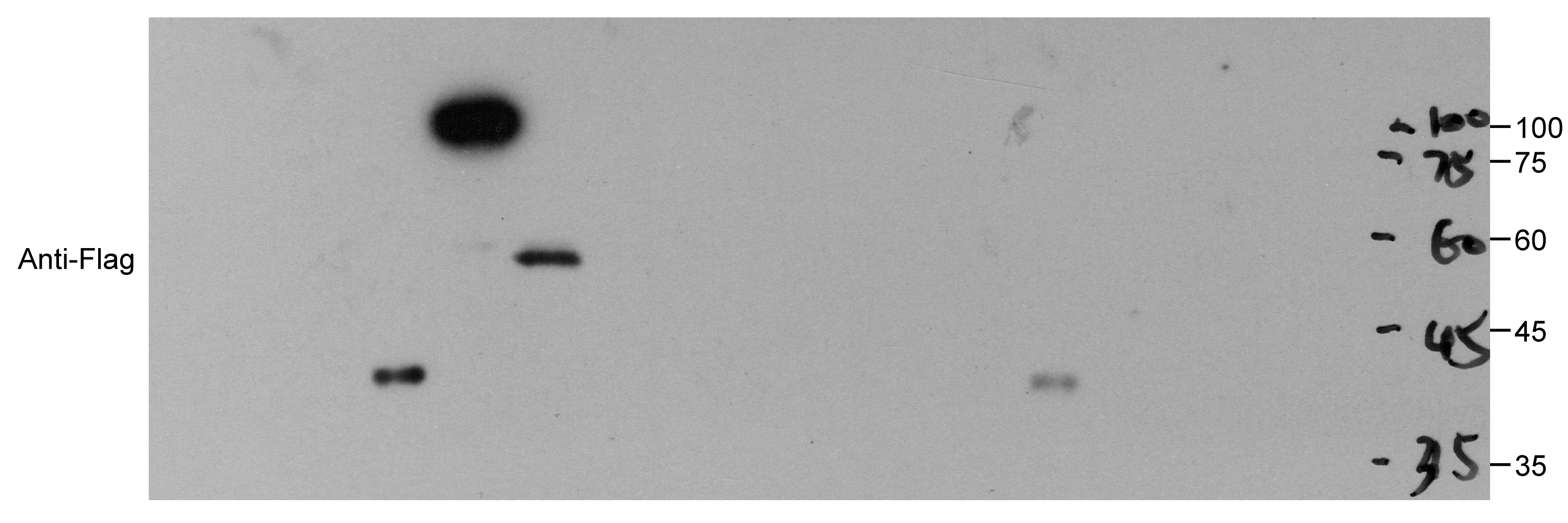

Supplement: Supplementary file 8 — Source data Fig. 2 [file 44318_2025_465_MOESM8_ESM.zip › EMBOJ-2025-120195-Figure 2-Source data/Figure 2/2A/western Flag.tif]

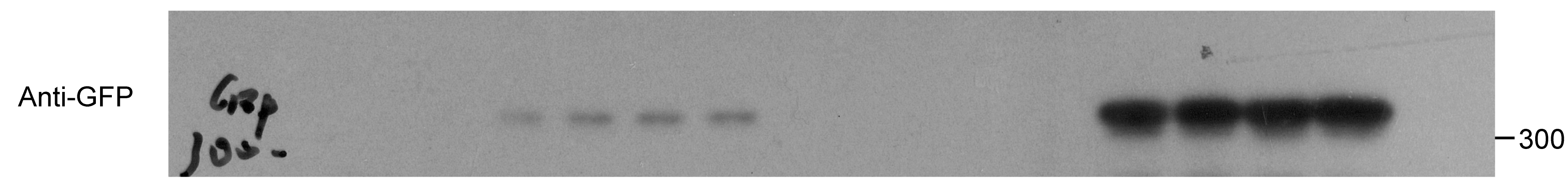

Supplement: Supplementary file 8 — Source data Fig. 2 [file 44318_2025_465_MOESM8_ESM.zip › EMBOJ-2025-120195-Figure 2-Source data/Figure 2/2A/western GFP.tif]

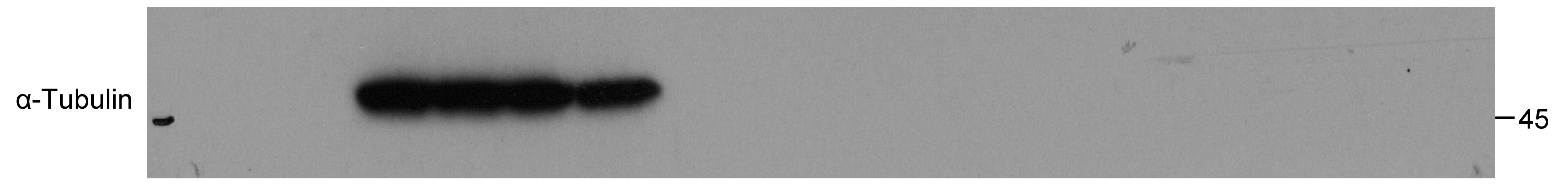

Supplement: Supplementary file 8 — Source data Fig. 2 [file 44318_2025_465_MOESM8_ESM.zip › EMBOJ-2025-120195-Figure 2-Source data/Figure 2/2A/western α-Tubulin.tif]

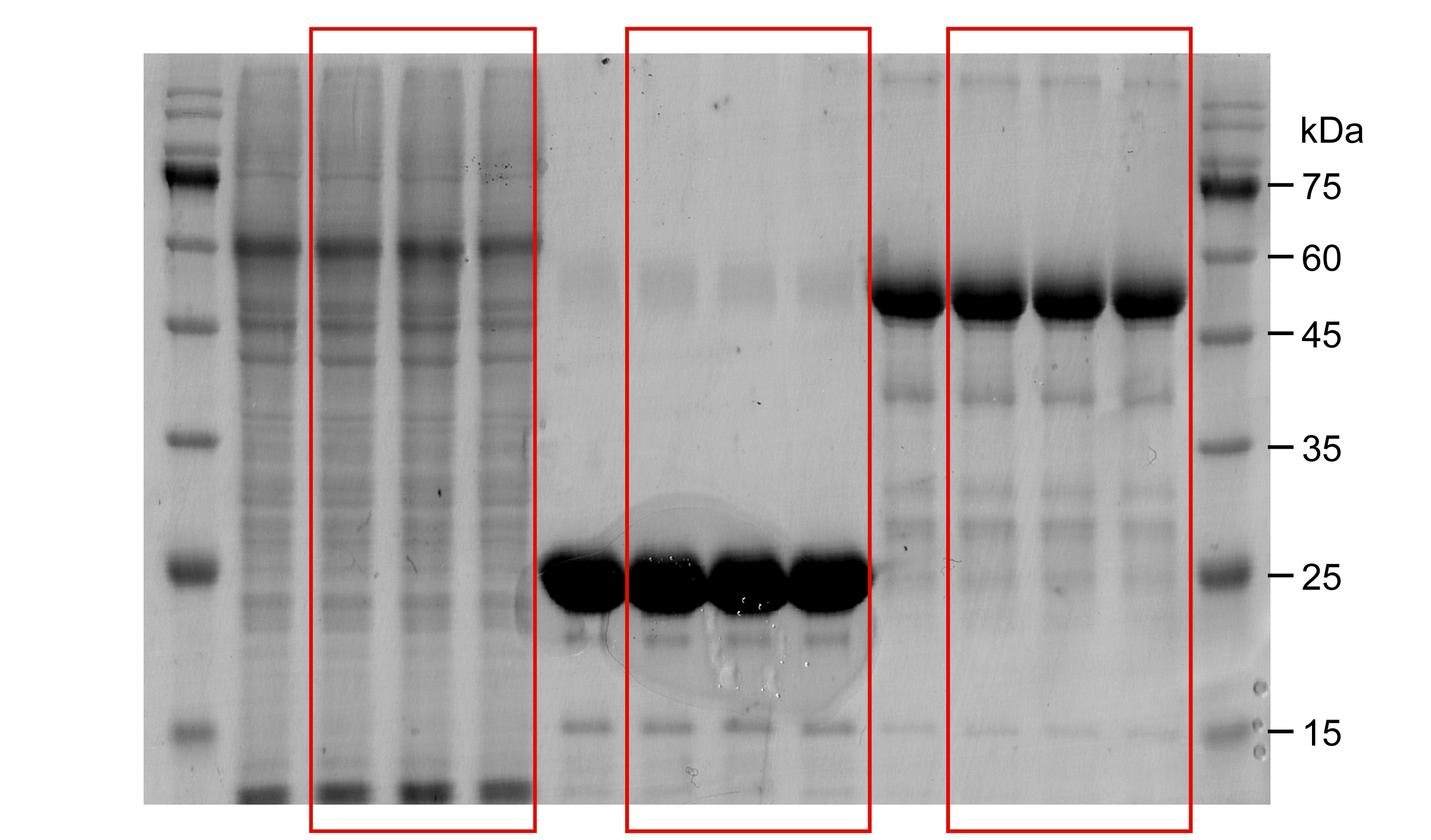

Supplement: Supplementary file 8 — Source data Fig. 2 [file 44318_2025_465_MOESM8_ESM.zip › EMBOJ-2025-120195-Figure 2-Source data/Figure 2/2C/CBB.tif]

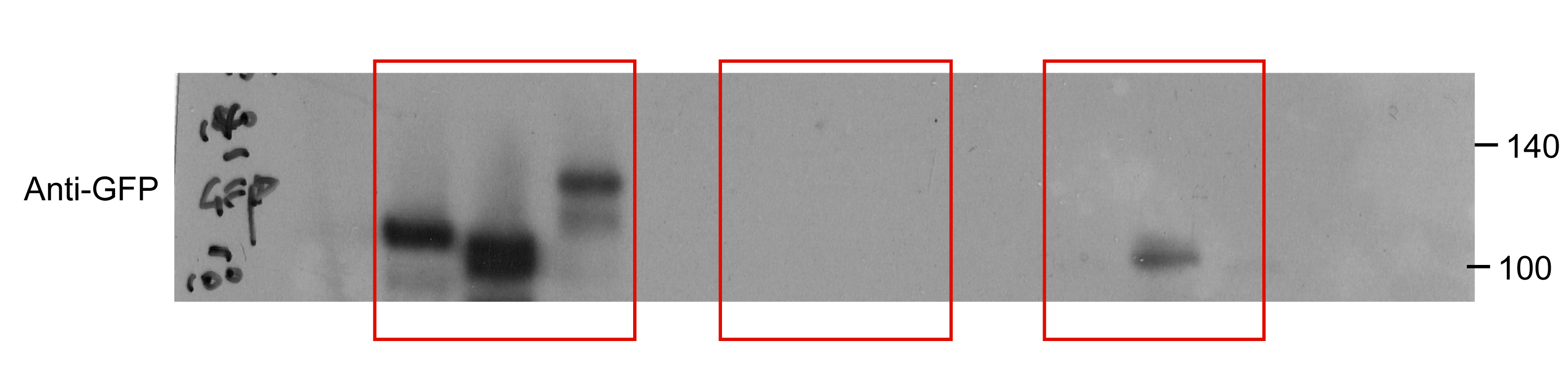

Supplement: Supplementary file 8 — Source data Fig. 2 [file 44318_2025_465_MOESM8_ESM.zip › EMBOJ-2025-120195-Figure 2-Source data/Figure 2/2C/western GFP.tif]

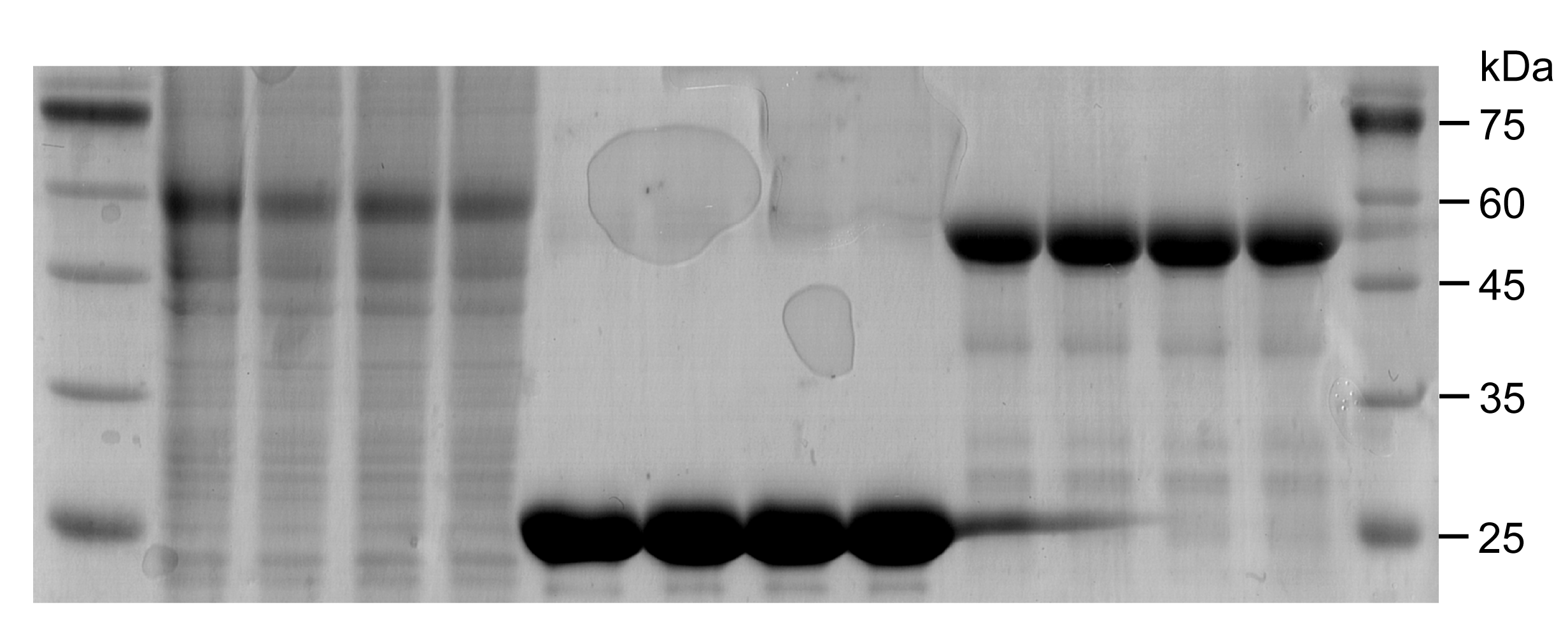

Supplement: Supplementary file 8 — Source data Fig. 2 [file 44318_2025_465_MOESM8_ESM.zip › EMBOJ-2025-120195-Figure 2-Source data/Figure 2/2D/CBB.tif]

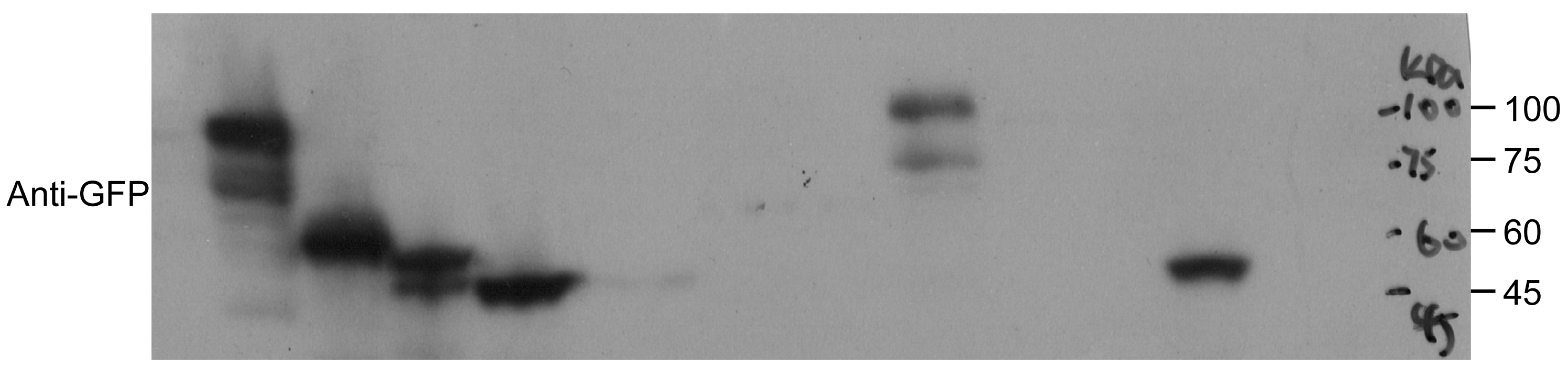

Supplement: Supplementary file 8 — Source data Fig. 2 [file 44318_2025_465_MOESM8_ESM.zip › EMBOJ-2025-120195-Figure 2-Source data/Figure 2/2D/western GFP.tif]

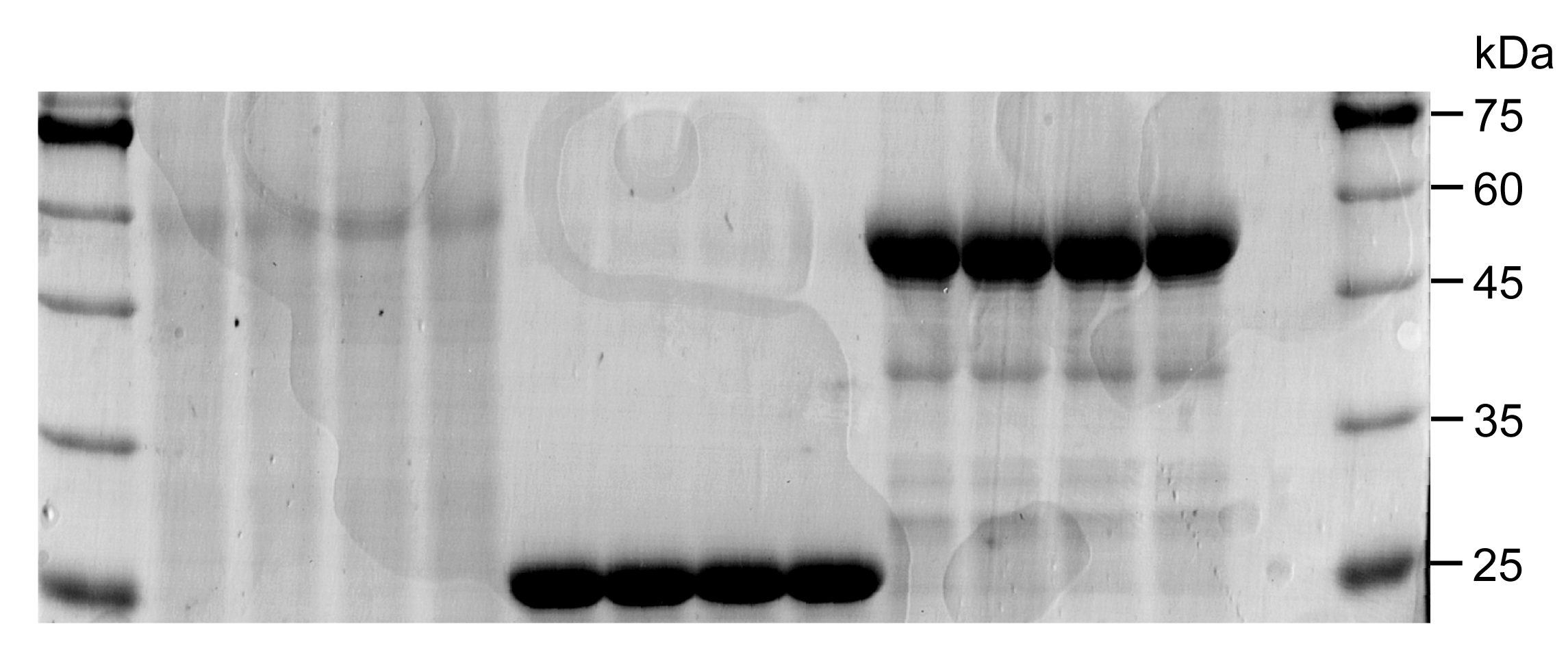

Supplement: Supplementary file 8 — Source data Fig. 2 [file 44318_2025_465_MOESM8_ESM.zip › EMBOJ-2025-120195-Figure 2-Source data/Figure 2/2E/CBB.tif]

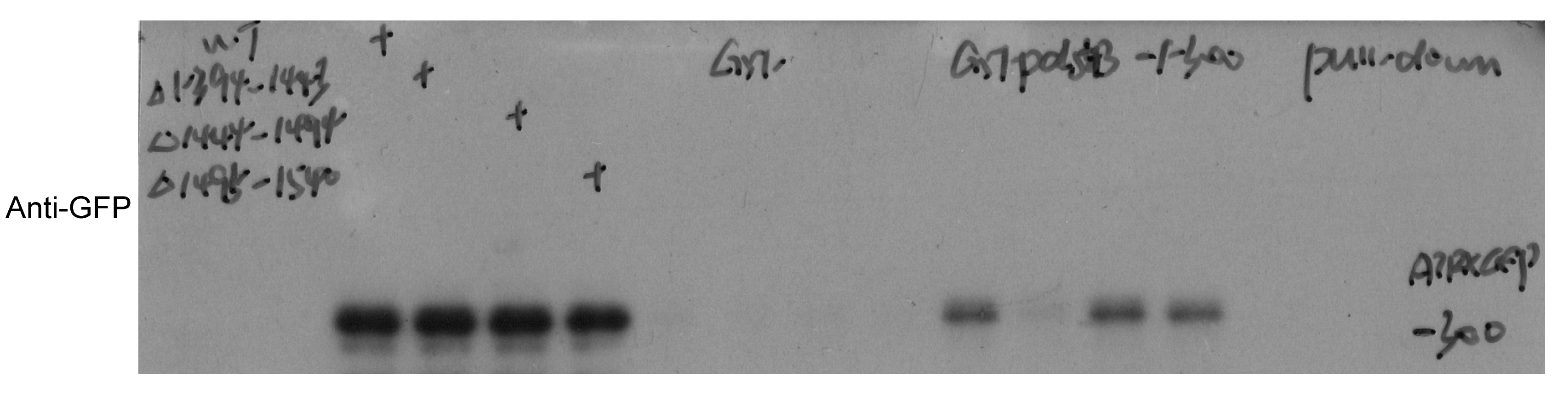

Supplement: Supplementary file 8 — Source data Fig. 2 [file 44318_2025_465_MOESM8_ESM.zip › EMBOJ-2025-120195-Figure 2-Source data/Figure 2/2E/western GFP.tif]

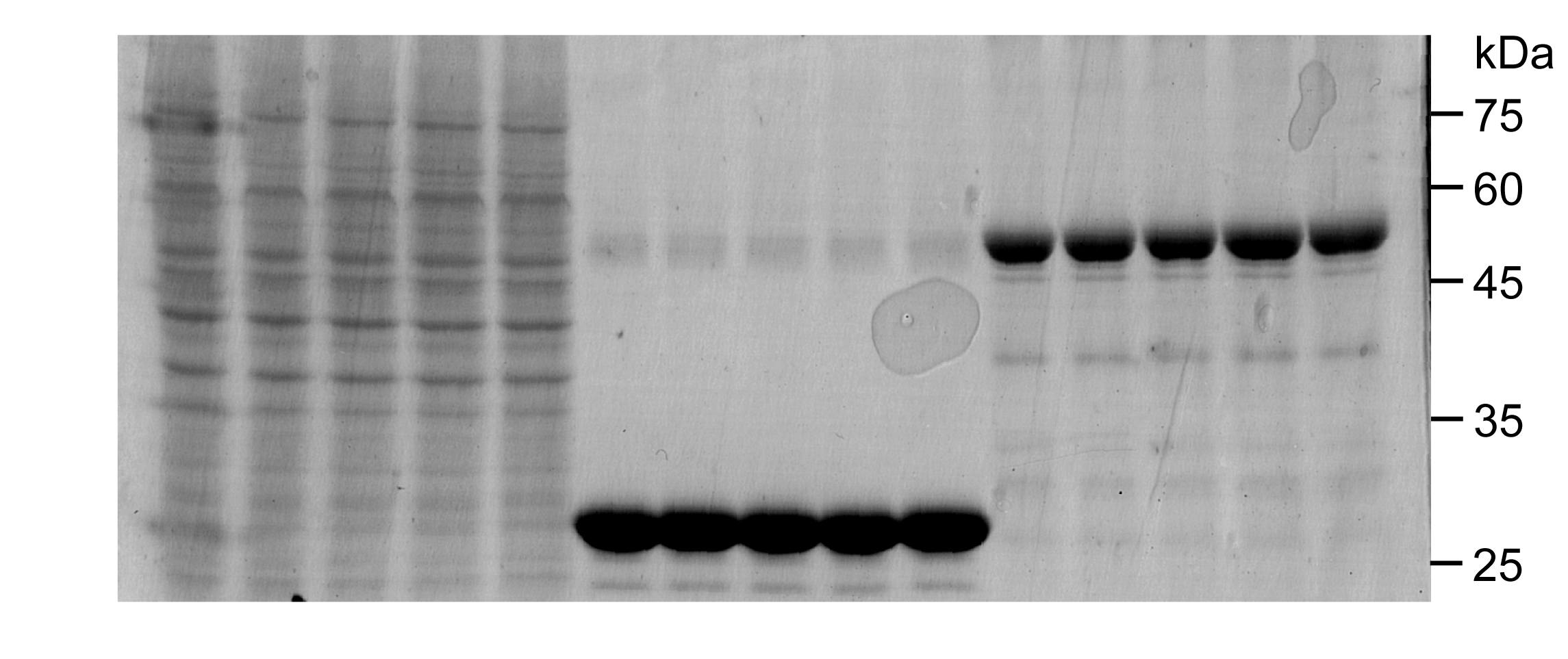

Supplement: Supplementary file 8 — Source data Fig. 2 [file 44318_2025_465_MOESM8_ESM.zip › EMBOJ-2025-120195-Figure 2-Source data/Figure 2/2F/CBB.tif]

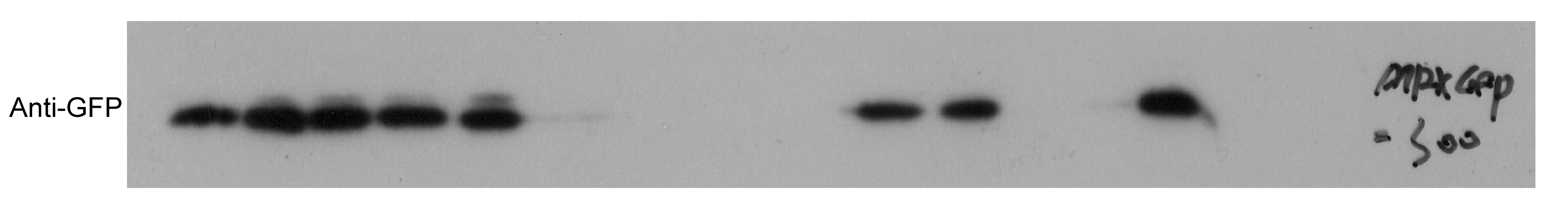

Supplement: Supplementary file 8 — Source data Fig. 2 [file 44318_2025_465_MOESM8_ESM.zip › EMBOJ-2025-120195-Figure 2-Source data/Figure 2/2F/western GFP.tif]

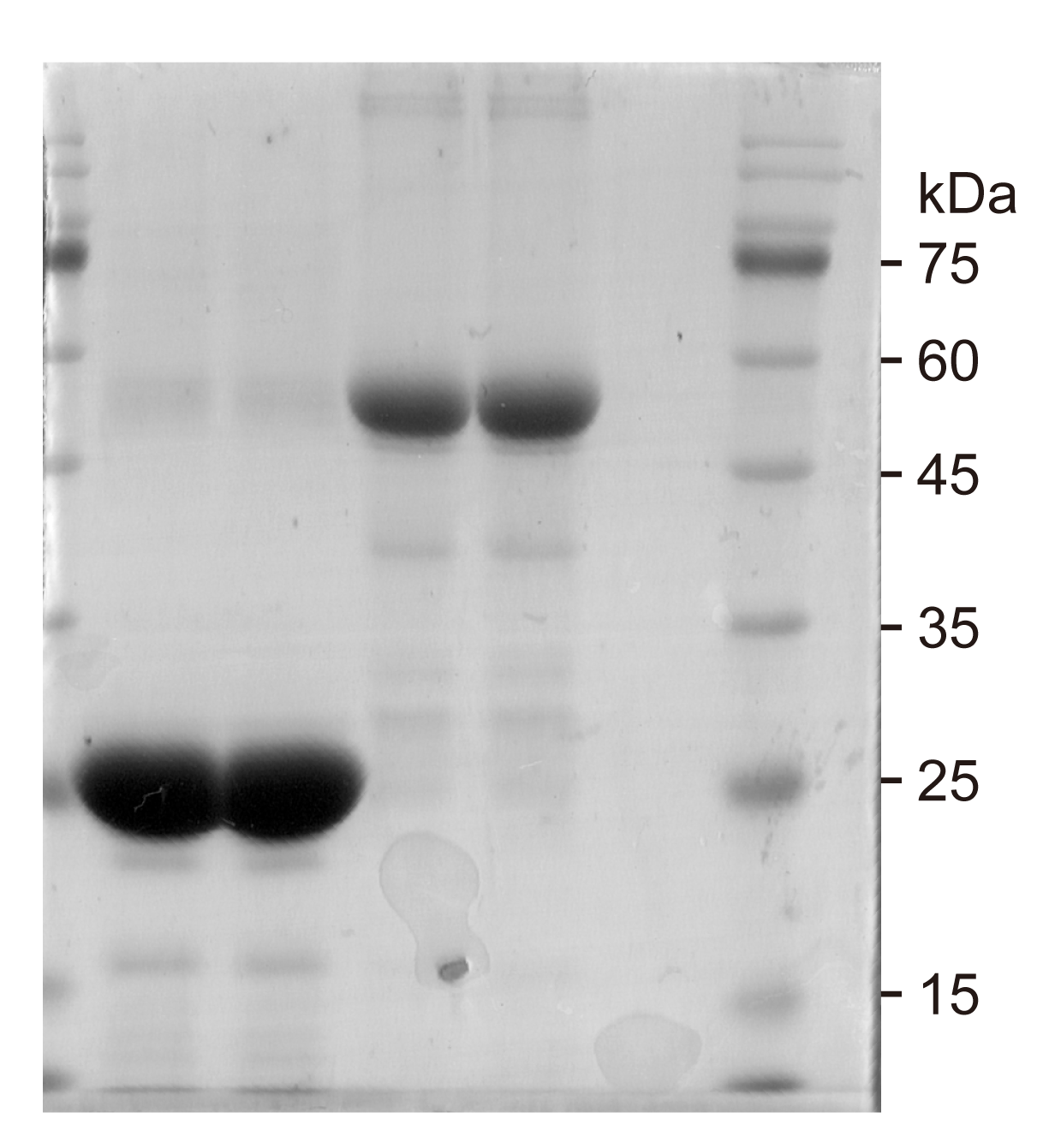

Supplement: Supplementary file 8 — Source data Fig. 2 [file 44318_2025_465_MOESM8_ESM.zip › EMBOJ-2025-120195-Figure 2-Source data/Figure 2/2G/CBB.tif]

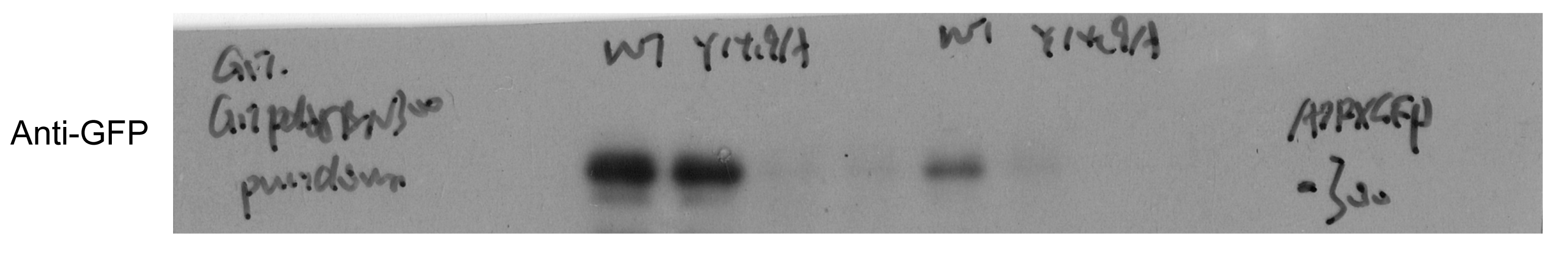

Supplement: Supplementary file 8 — Source data Fig. 2 [file 44318_2025_465_MOESM8_ESM.zip › EMBOJ-2025-120195-Figure 2-Source data/Figure 2/2G/western GFP.tif]

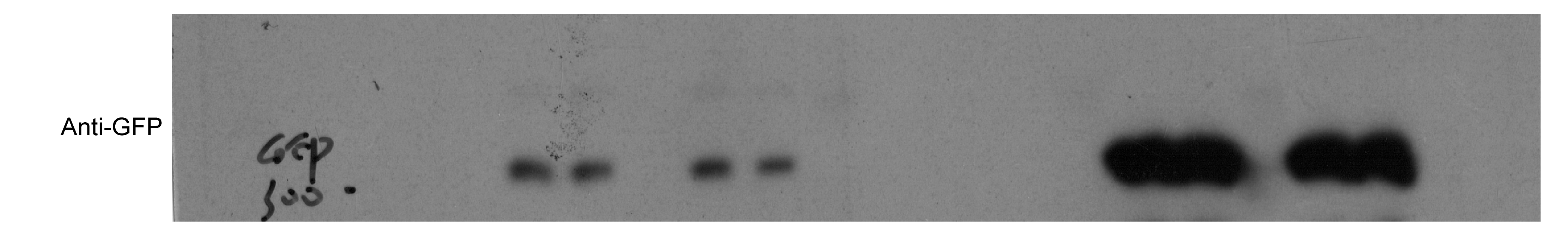

Supplement: Supplementary file 8 — Source data Fig. 2 [file 44318_2025_465_MOESM8_ESM.zip › EMBOJ-2025-120195-Figure 2-Source data/Figure 2/2H/western GFP.tif]

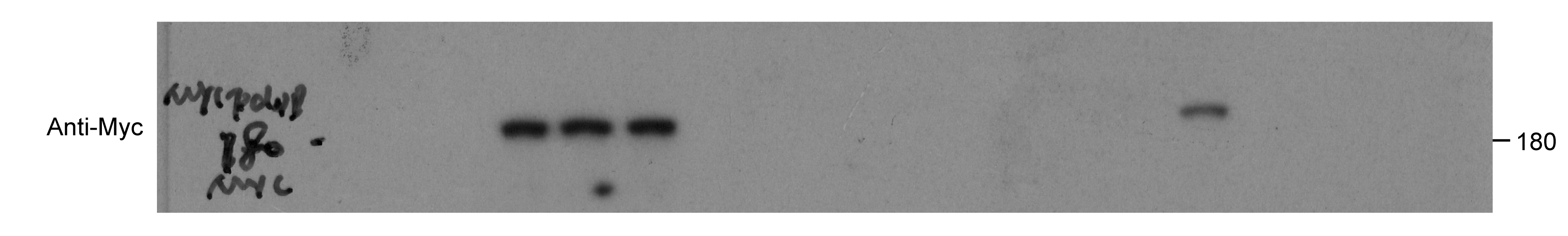

Supplement: Supplementary file 8 — Source data Fig. 2 [file 44318_2025_465_MOESM8_ESM.zip › EMBOJ-2025-120195-Figure 2-Source data/Figure 2/2H/western Myc.tif]

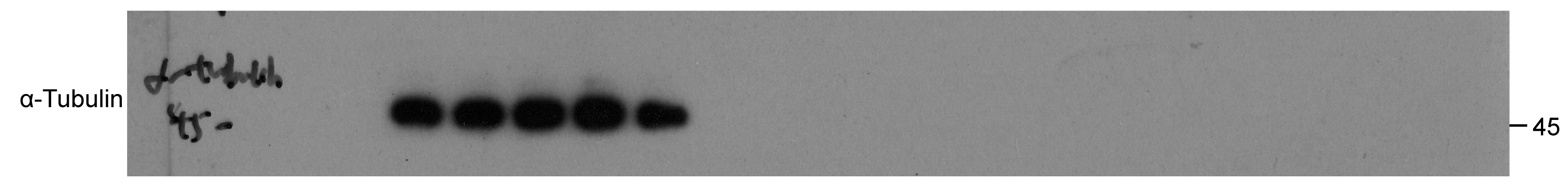

Supplement: Supplementary file 8 — Source data Fig. 2 [file 44318_2025_465_MOESM8_ESM.zip › EMBOJ-2025-120195-Figure 2-Source data/Figure 2/2H/western α-Tubulin.tif]

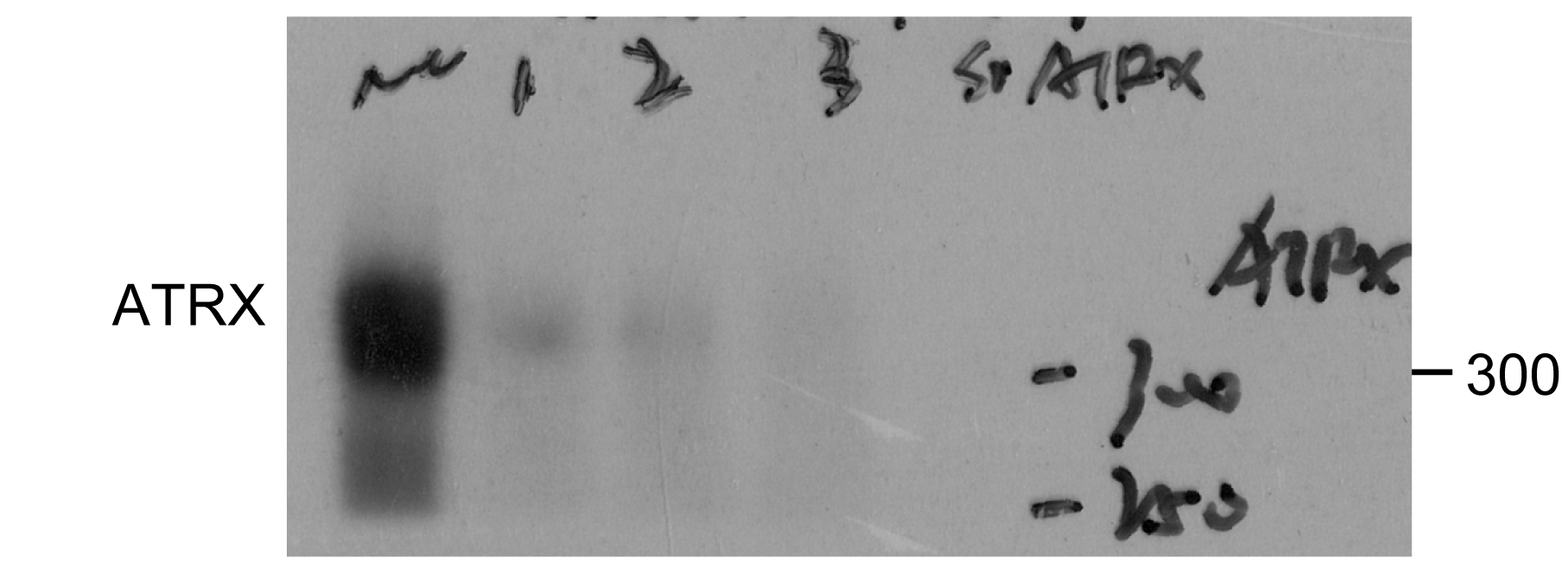

Supplement: Supplementary file 9 — Source data Fig. 3 [file 44318_2025_465_MOESM9_ESM.zip › EMBOJ-2025-120195-Figure 3-Source data/Figure 3/3A/western ATRX.tif]

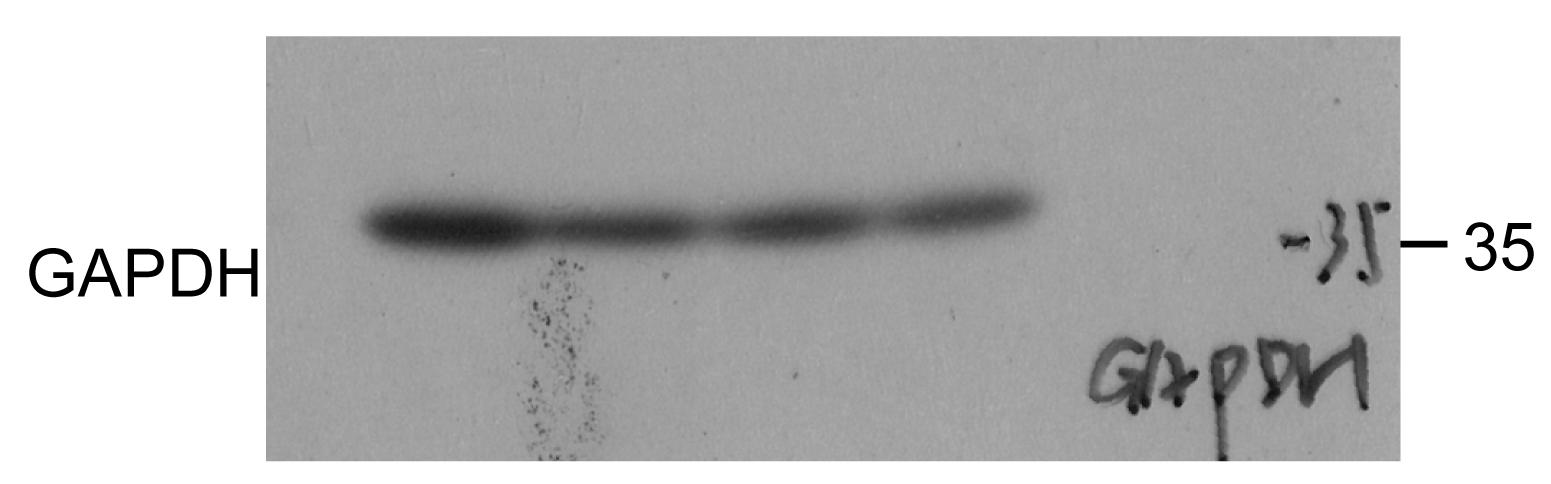

Supplement: Supplementary file 9 — Source data Fig. 3 [file 44318_2025_465_MOESM9_ESM.zip › EMBOJ-2025-120195-Figure 3-Source data/Figure 3/3A/western GAPDH.tif]

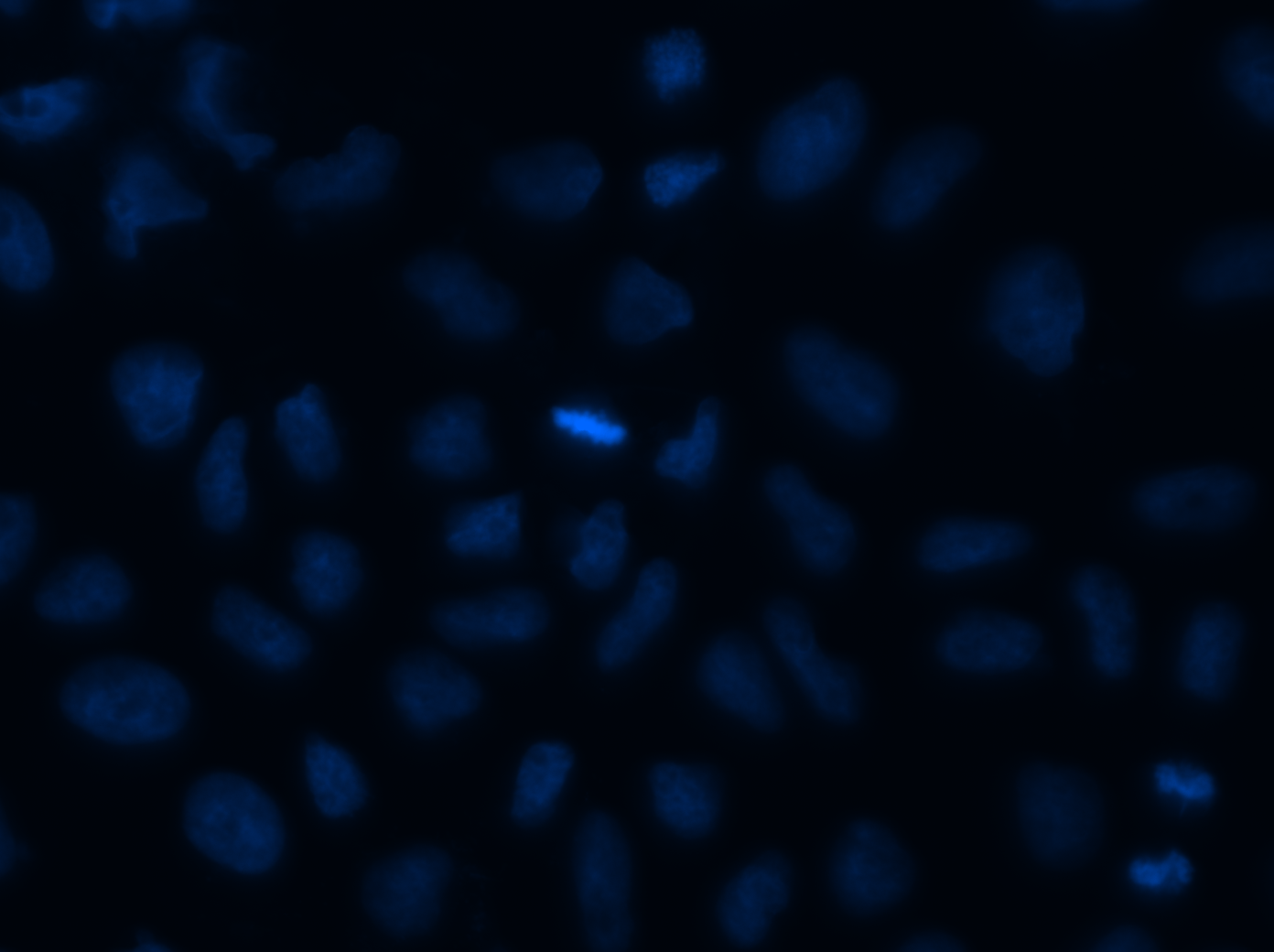

Supplement: Supplementary file 9 — Source data Fig. 3 [file 44318_2025_465_MOESM9_ESM.zip › EMBOJ-2025-120195-Figure 3-Source data/Figure 3/3C/Metaphase.tif]

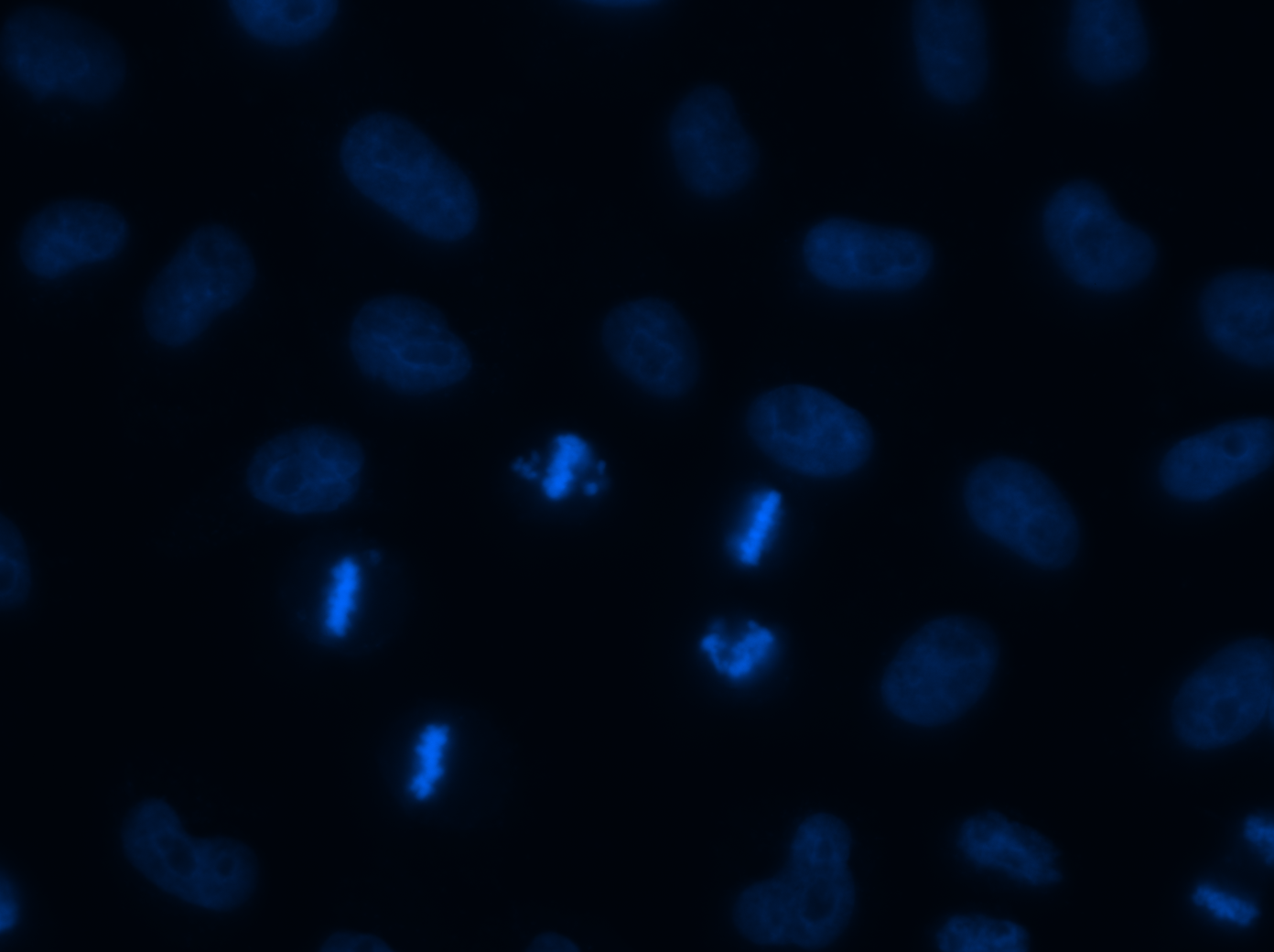

Supplement: Supplementary file 9 — Source data Fig. 3 [file 44318_2025_465_MOESM9_ESM.zip › EMBOJ-2025-120195-Figure 3-Source data/Figure 3/3C/Misalignment.tif]

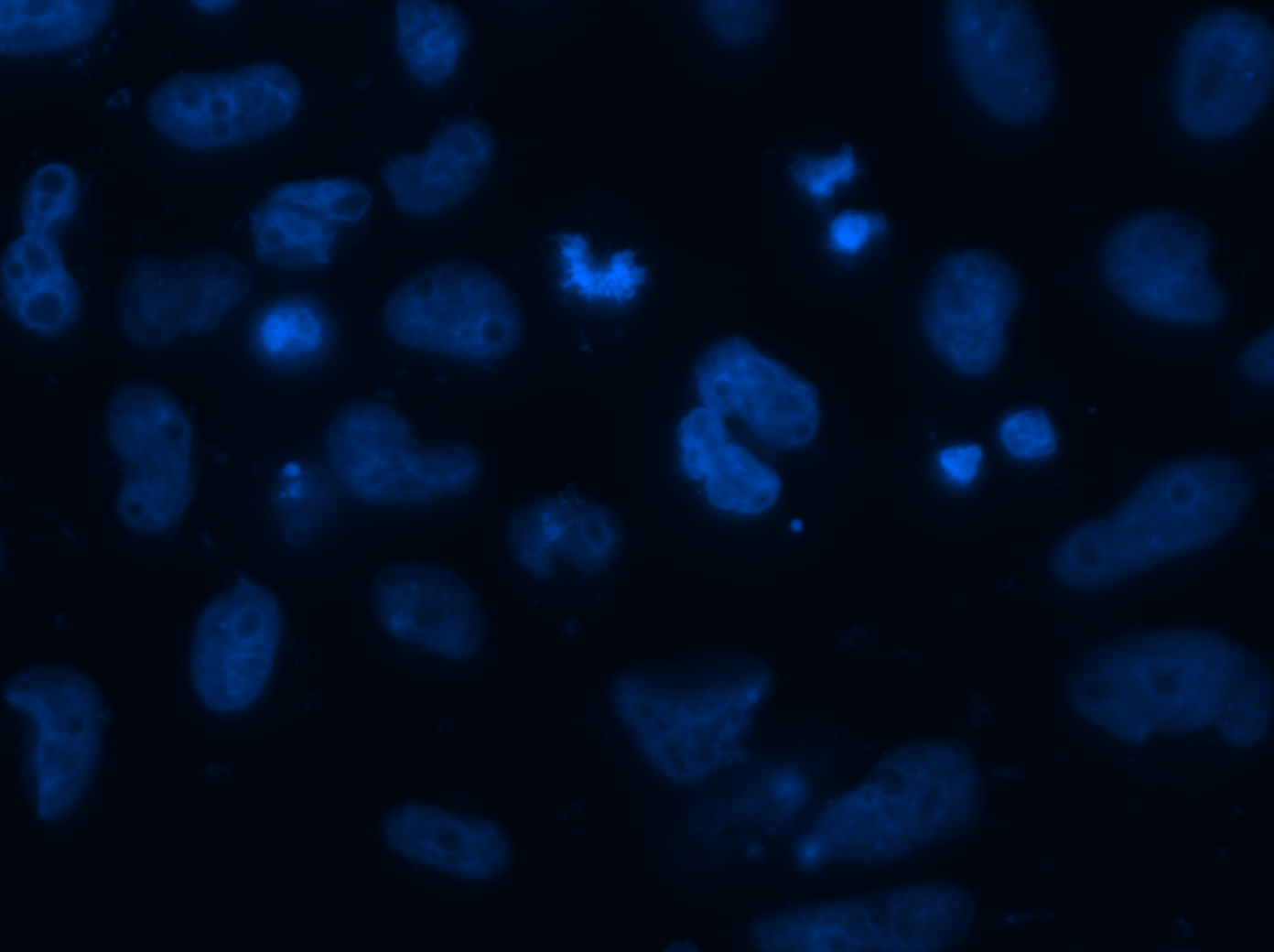

Supplement: Supplementary file 9 — Source data Fig. 3 [file 44318_2025_465_MOESM9_ESM.zip › EMBOJ-2025-120195-Figure 3-Source data/Figure 3/3C/Prometaphase.tif]

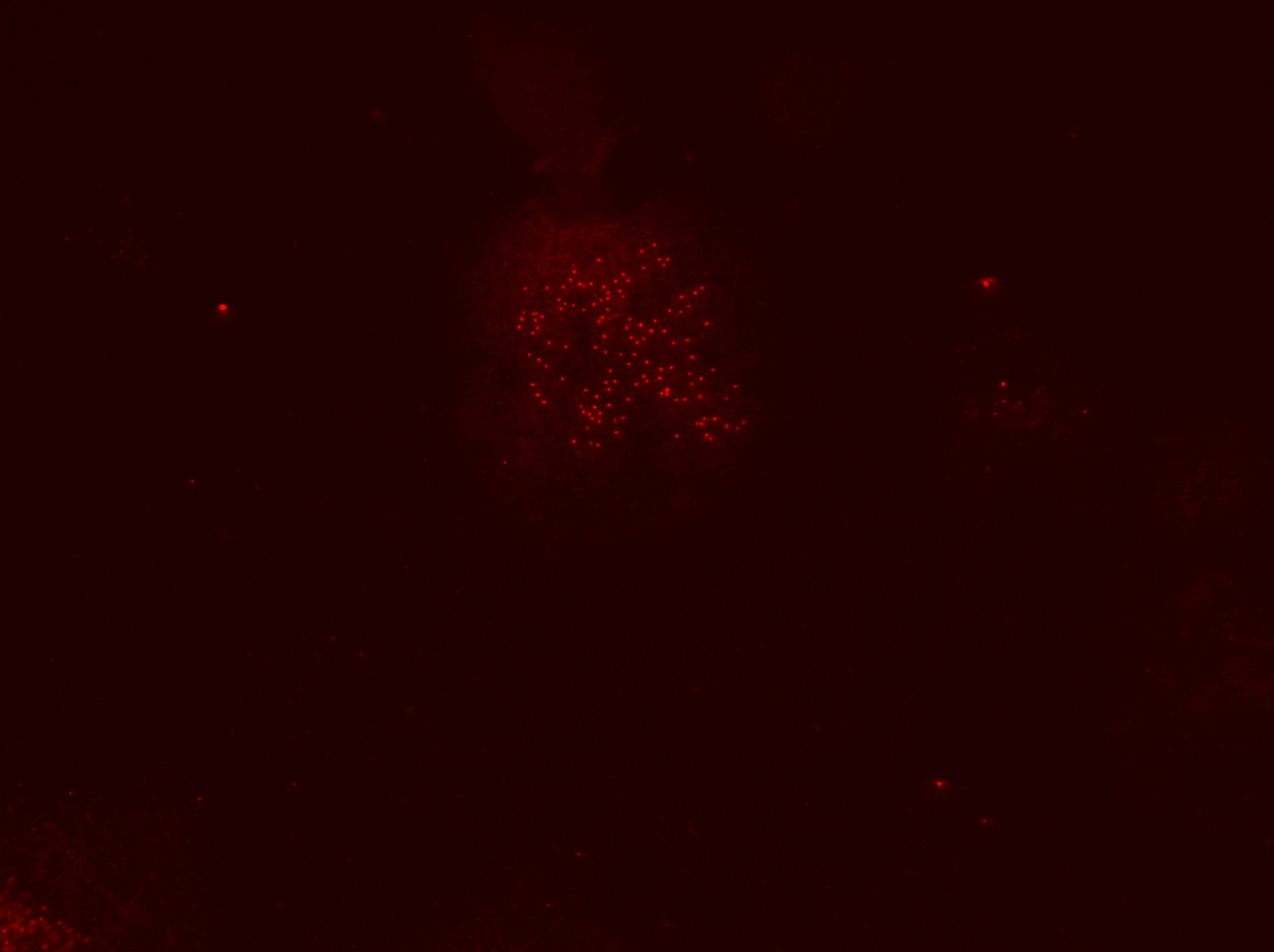

Supplement: Supplementary file 9 — Source data Fig. 3 [file 44318_2025_465_MOESM9_ESM.zip › EMBOJ-2025-120195-Figure 3-Source data/Figure 3/3F/HeLa-siATRX# 1 CENP-C.tif]

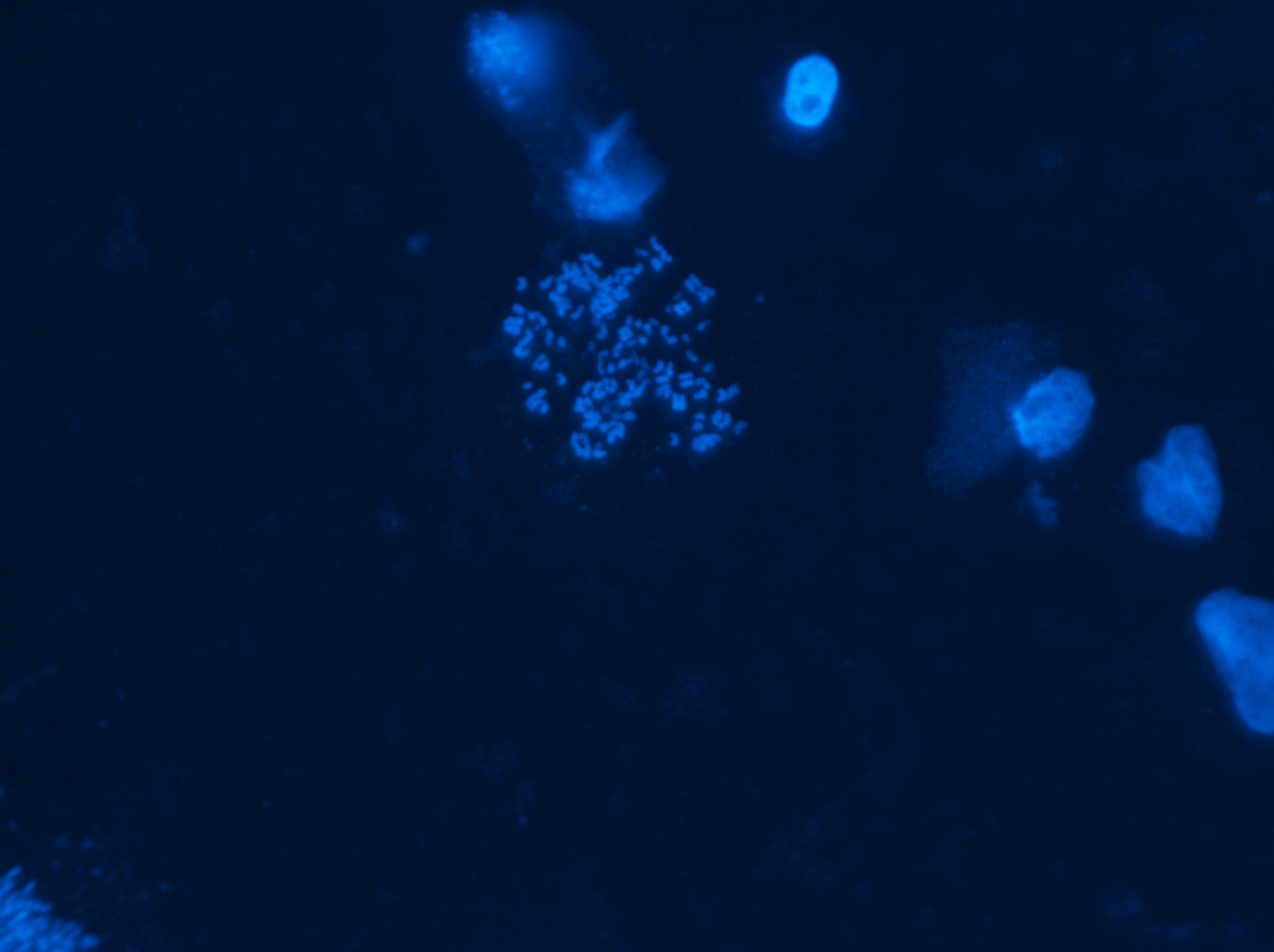

Supplement: Supplementary file 9 — Source data Fig. 3 [file 44318_2025_465_MOESM9_ESM.zip › EMBOJ-2025-120195-Figure 3-Source data/Figure 3/3F/HeLa-siATRX# 1 DNA.tif]

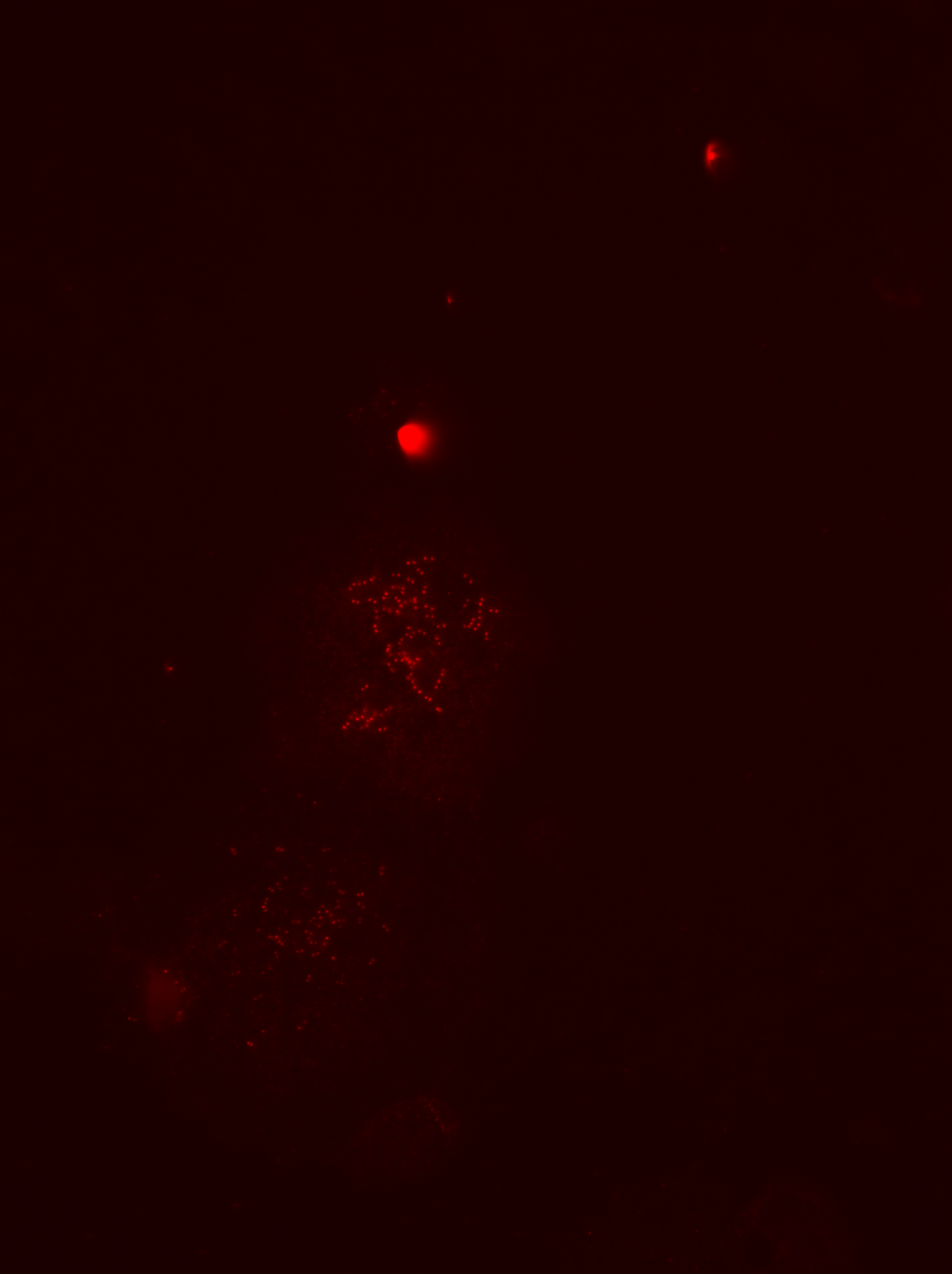

Supplement: Supplementary file 9 — Source data Fig. 3 [file 44318_2025_465_MOESM9_ESM.zip › EMBOJ-2025-120195-Figure 3-Source data/Figure 3/3F/HeLa-siControl CENP-C.tif]

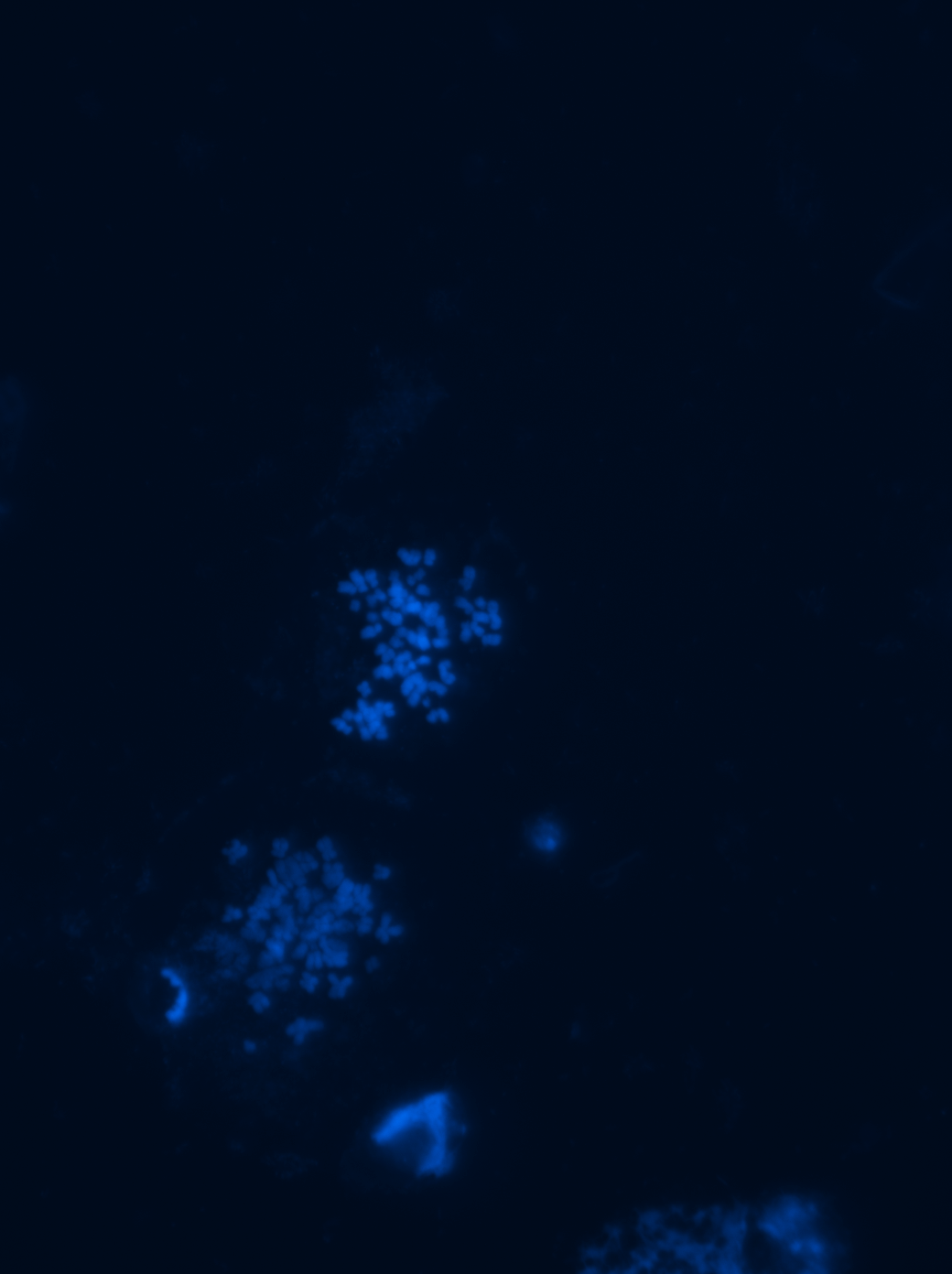

Supplement: Supplementary file 9 — Source data Fig. 3 [file 44318_2025_465_MOESM9_ESM.zip › EMBOJ-2025-120195-Figure 3-Source data/Figure 3/3F/HeLa-siControl DNA.tif]

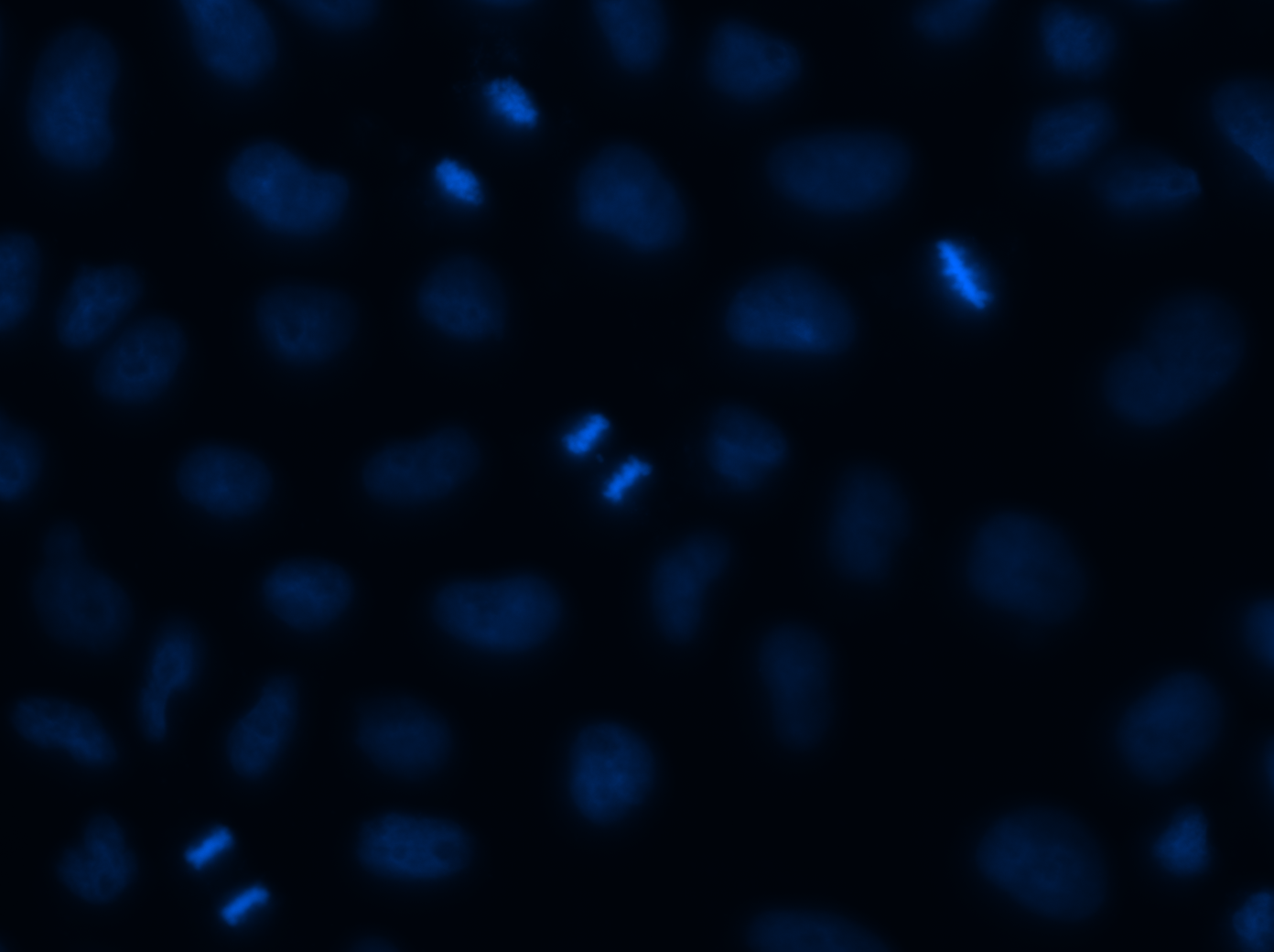

Supplement: Supplementary file 9 — Source data Fig. 3 [file 44318_2025_465_MOESM9_ESM.zip › EMBOJ-2025-120195-Figure 3-Source data/Figure 3/3H/Anaphase cells with lagging chromosomes (1).tif]

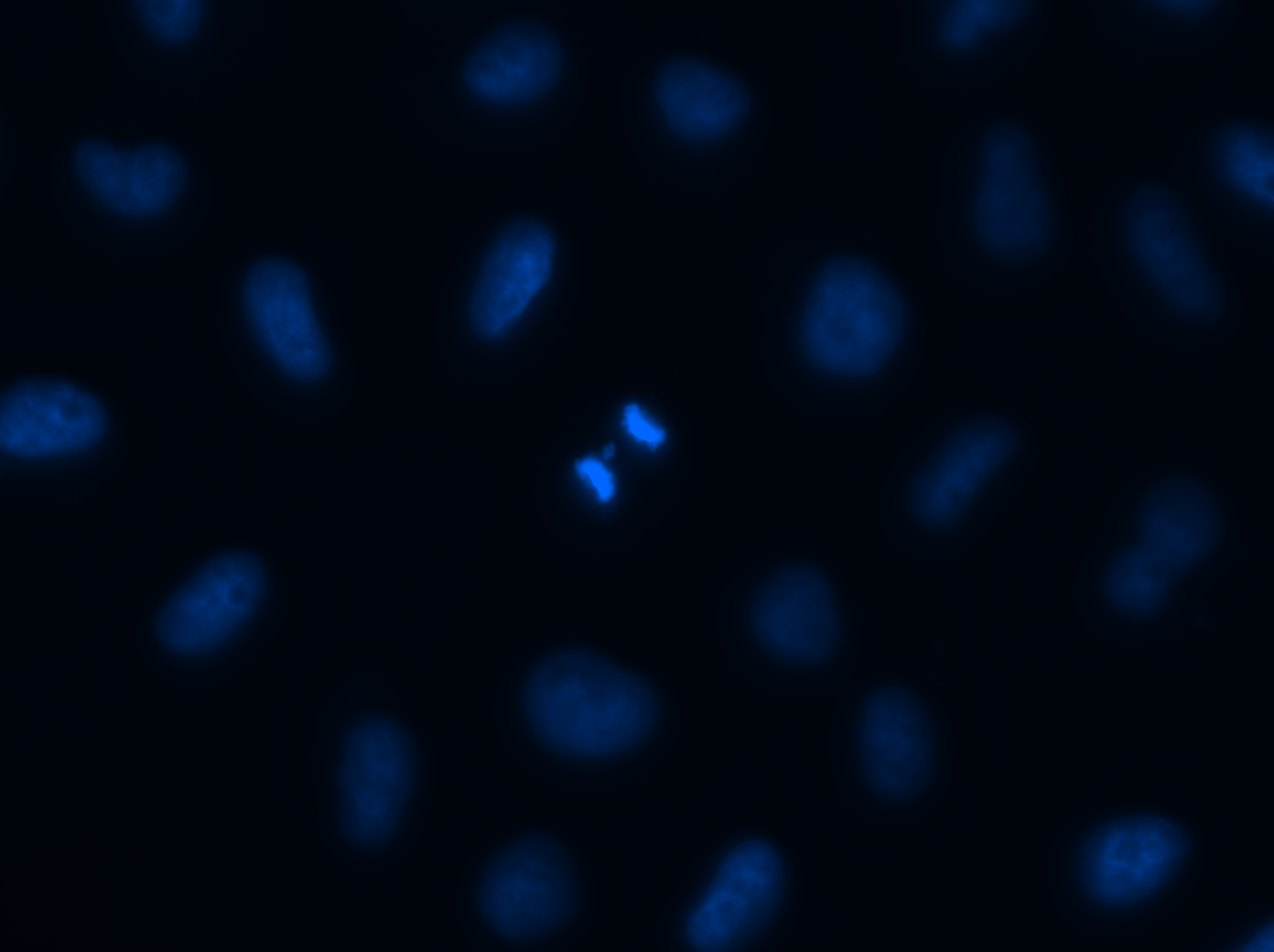

Supplement: Supplementary file 9 — Source data Fig. 3 [file 44318_2025_465_MOESM9_ESM.zip › EMBOJ-2025-120195-Figure 3-Source data/Figure 3/3H/Anaphase cells with lagging chromosomes (2).tif]

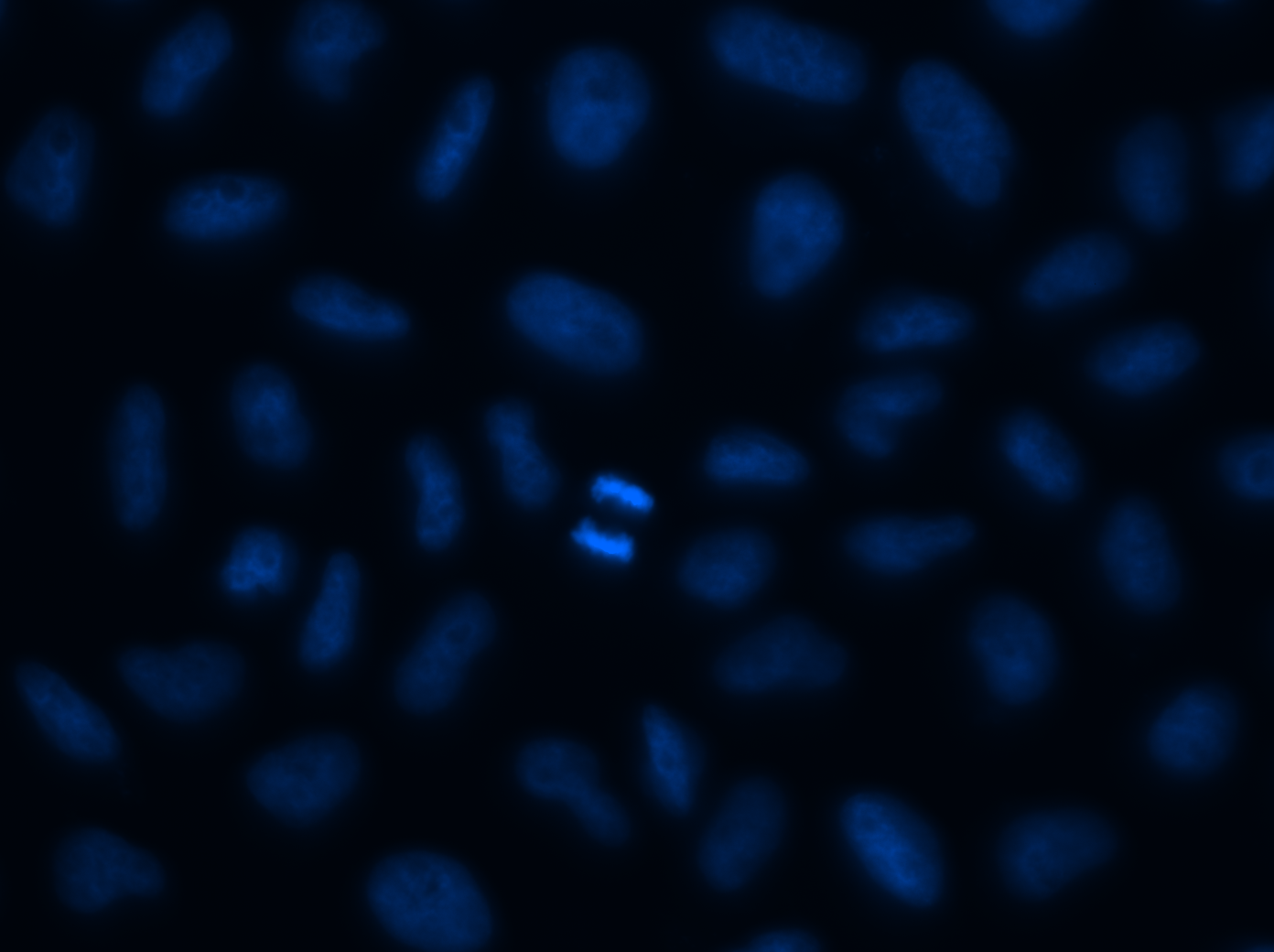

Supplement: Supplementary file 9 — Source data Fig. 3 [file 44318_2025_465_MOESM9_ESM.zip › EMBOJ-2025-120195-Figure 3-Source data/Figure 3/3H/Normal Anaphase.tif]

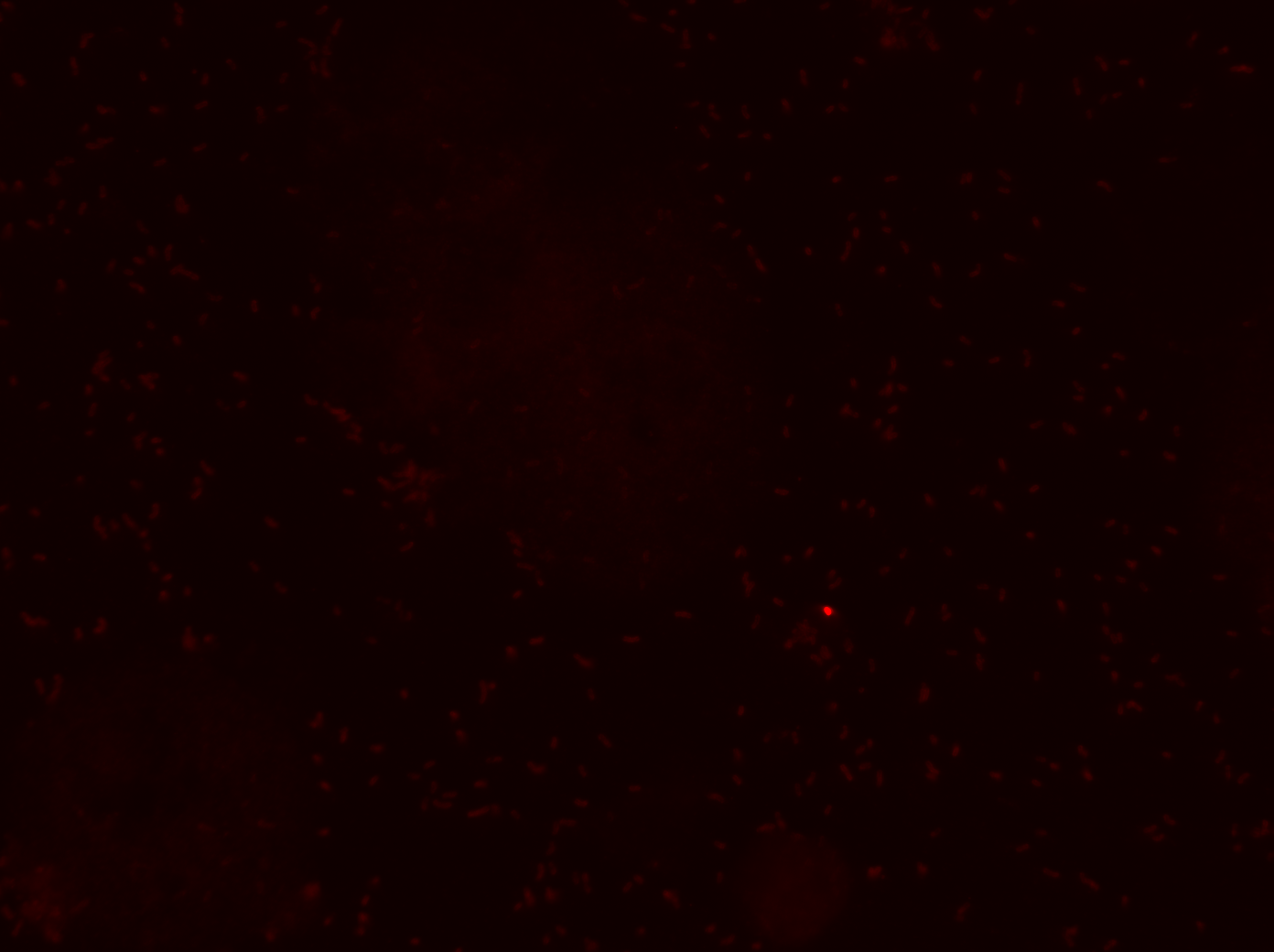

Supplement: Supplementary file 10 — Source data Fig. 4 [file 44318_2025_465_MOESM10_ESM.zip › EMBOJ-2025-120195-Figure 4-Source data/Figure 4/4A/siATRX#1 ATRX.tif]

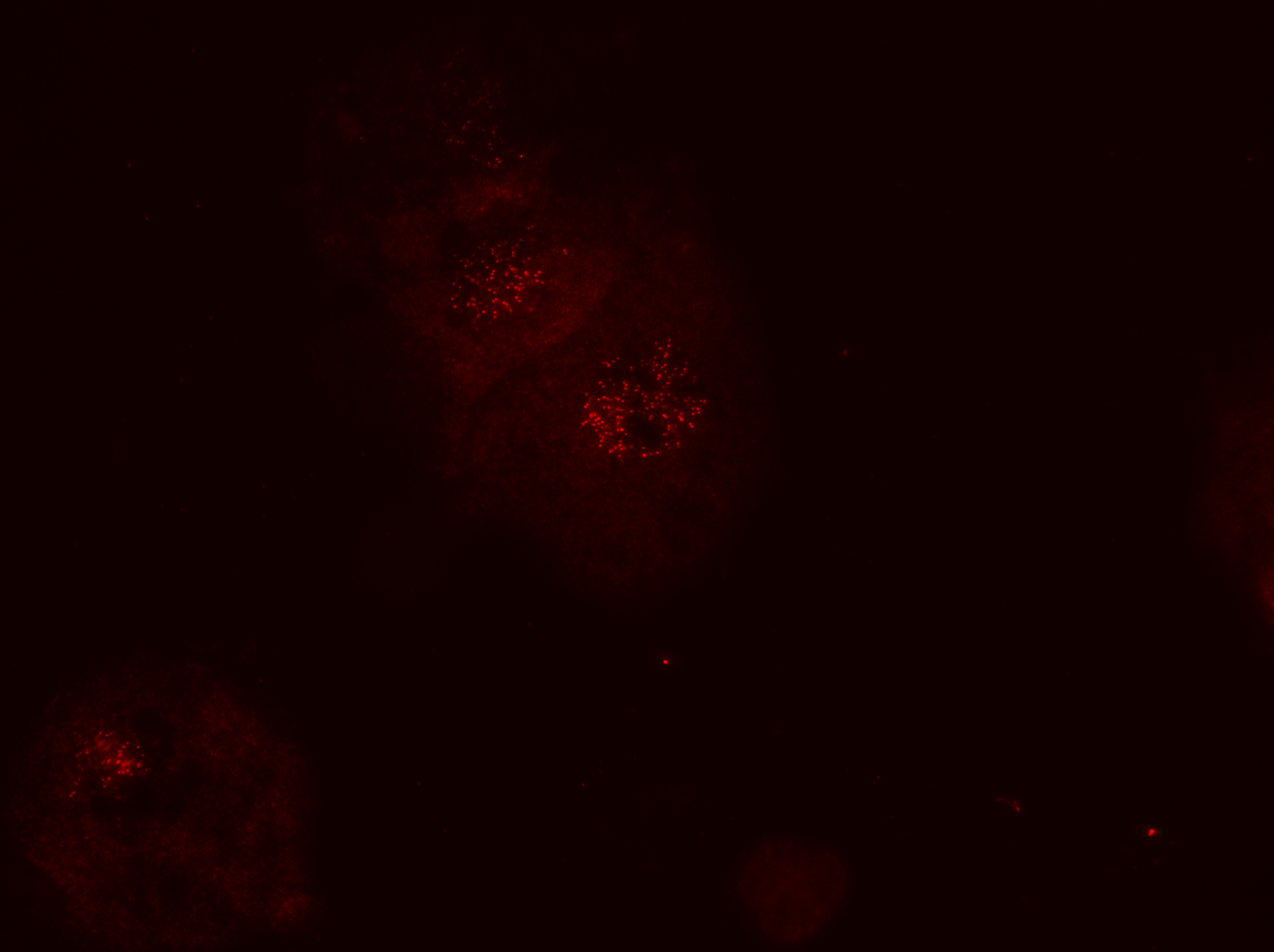

Supplement: Supplementary file 10 — Source data Fig. 4 [file 44318_2025_465_MOESM10_ESM.zip › EMBOJ-2025-120195-Figure 4-Source data/Figure 4/4A/siATRX#1 CENP-C.tif]

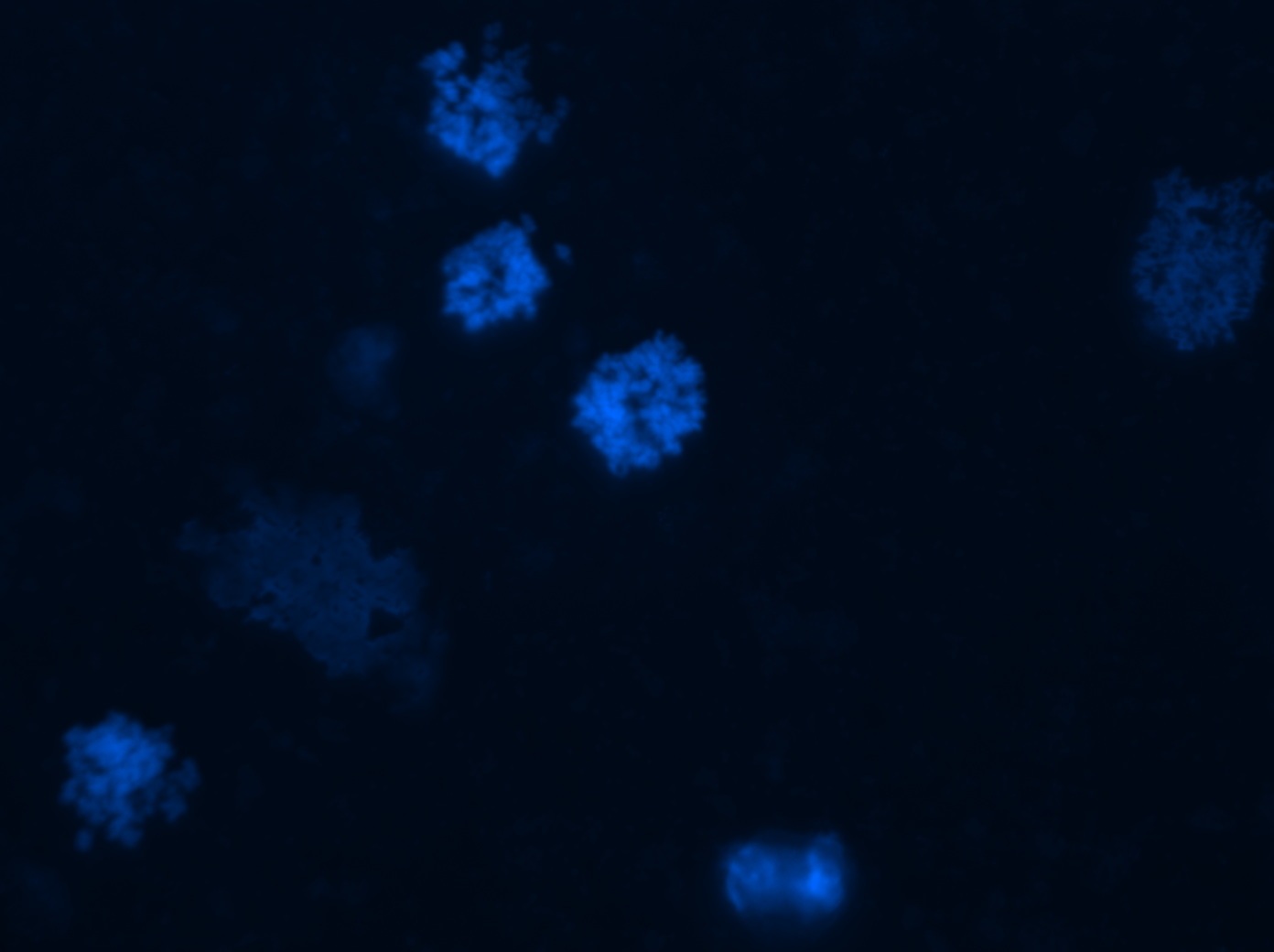

Supplement: Supplementary file 10 — Source data Fig. 4 [file 44318_2025_465_MOESM10_ESM.zip › EMBOJ-2025-120195-Figure 4-Source data/Figure 4/4A/siATRX#1 DNA.tif]

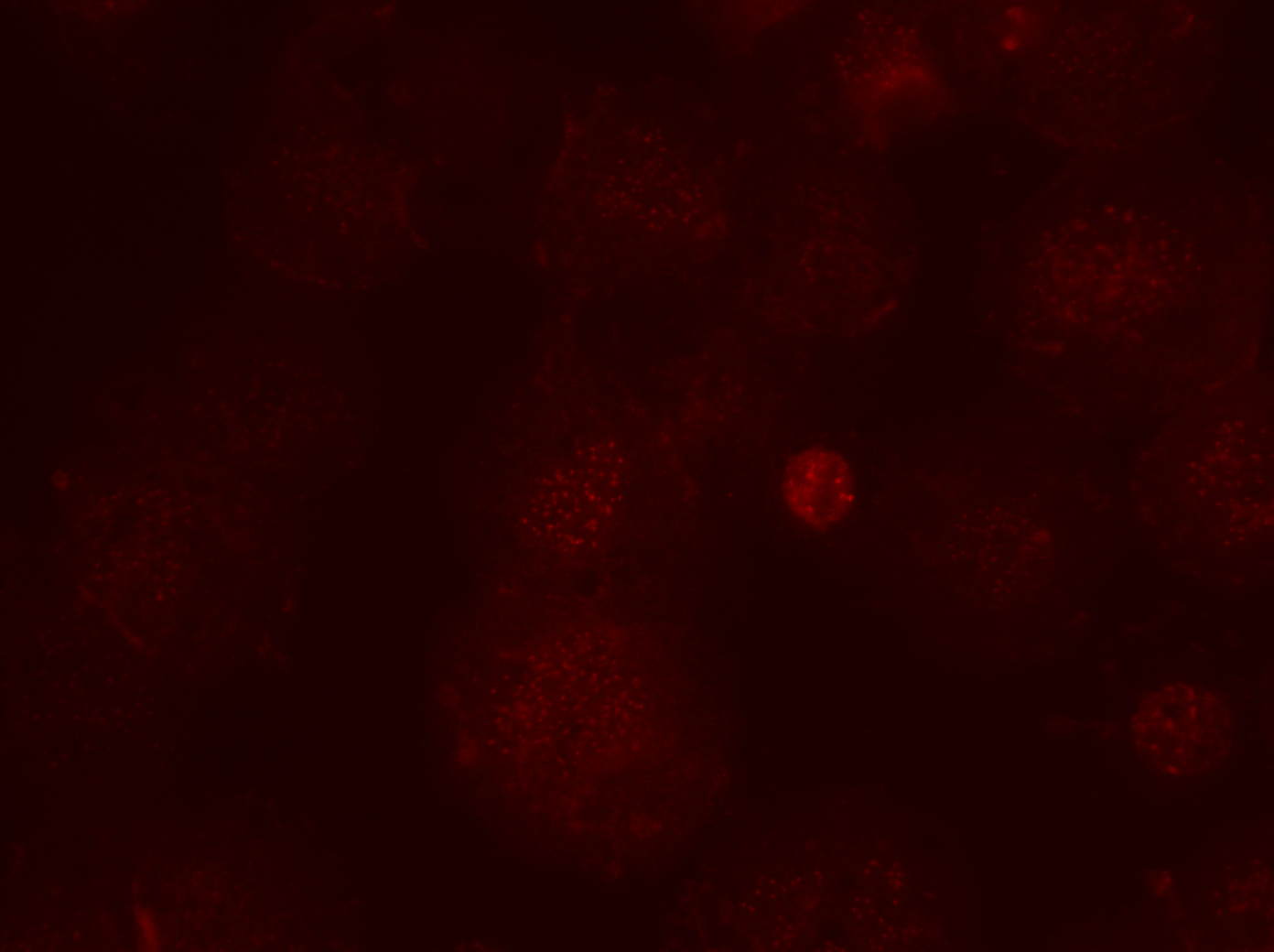

Supplement: Supplementary file 10 — Source data Fig. 4 [file 44318_2025_465_MOESM10_ESM.zip › EMBOJ-2025-120195-Figure 4-Source data/Figure 4/4A/siControl ATRX.tif]

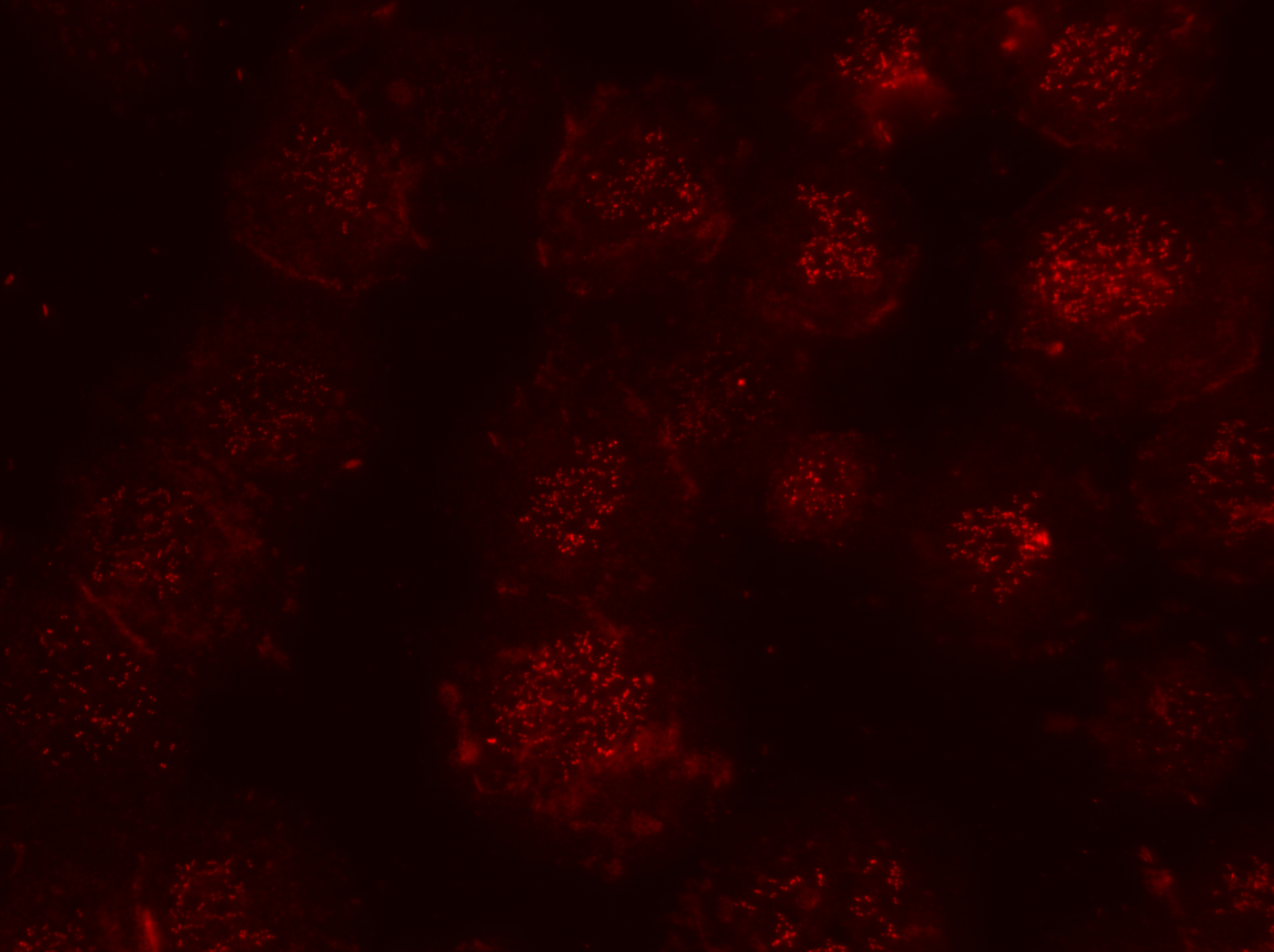

Supplement: Supplementary file 10 — Source data Fig. 4 [file 44318_2025_465_MOESM10_ESM.zip › EMBOJ-2025-120195-Figure 4-Source data/Figure 4/4A/siControl CENP-C.tif]

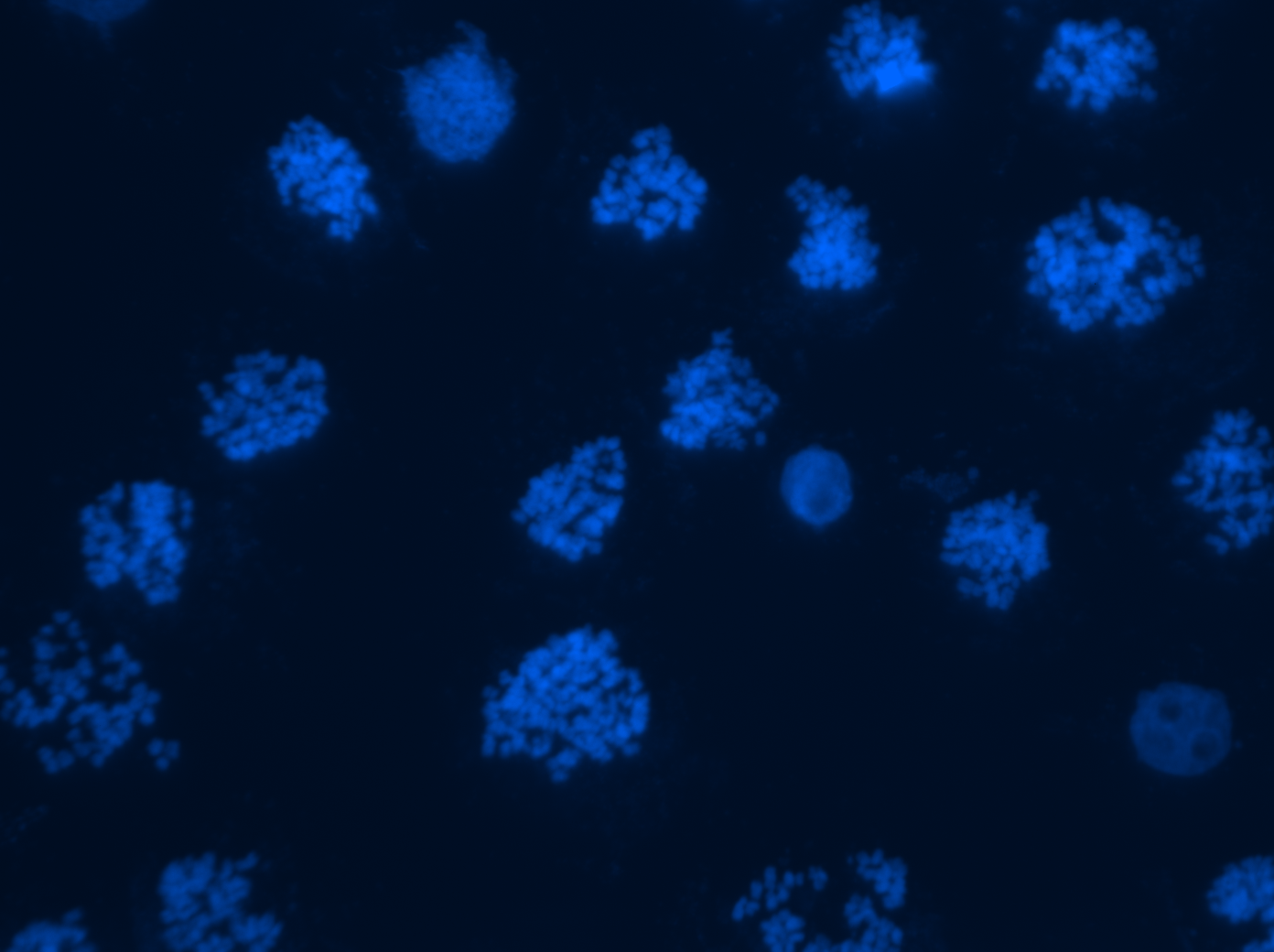

Supplement: Supplementary file 10 — Source data Fig. 4 [file 44318_2025_465_MOESM10_ESM.zip › EMBOJ-2025-120195-Figure 4-Source data/Figure 4/4A/siControl DNA.tif]

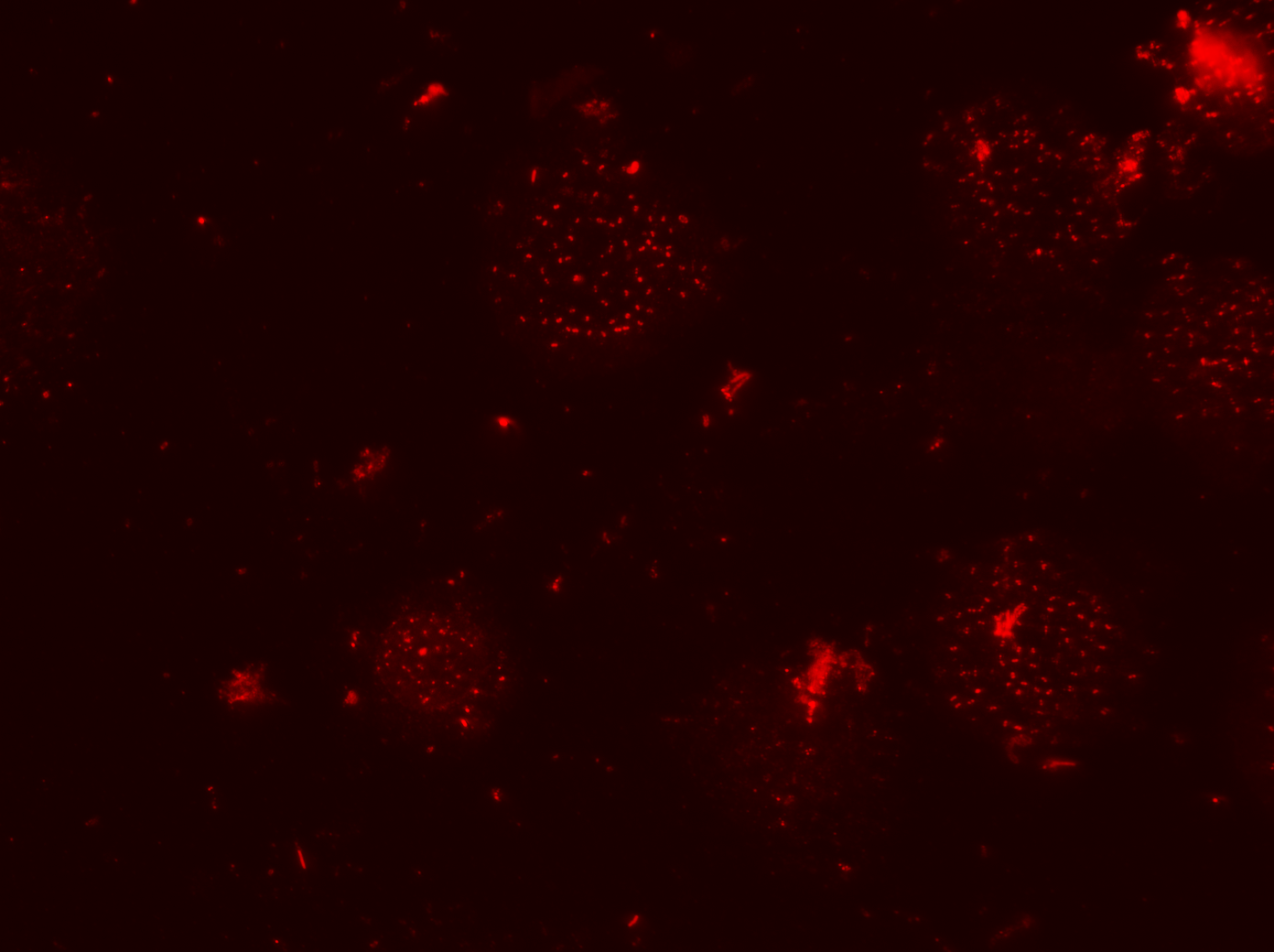

Supplement: Supplementary file 10 — Source data Fig. 4 [file 44318_2025_465_MOESM10_ESM.zip › EMBOJ-2025-120195-Figure 4-Source data/Figure 4/4B/HeLa ACA.tif]

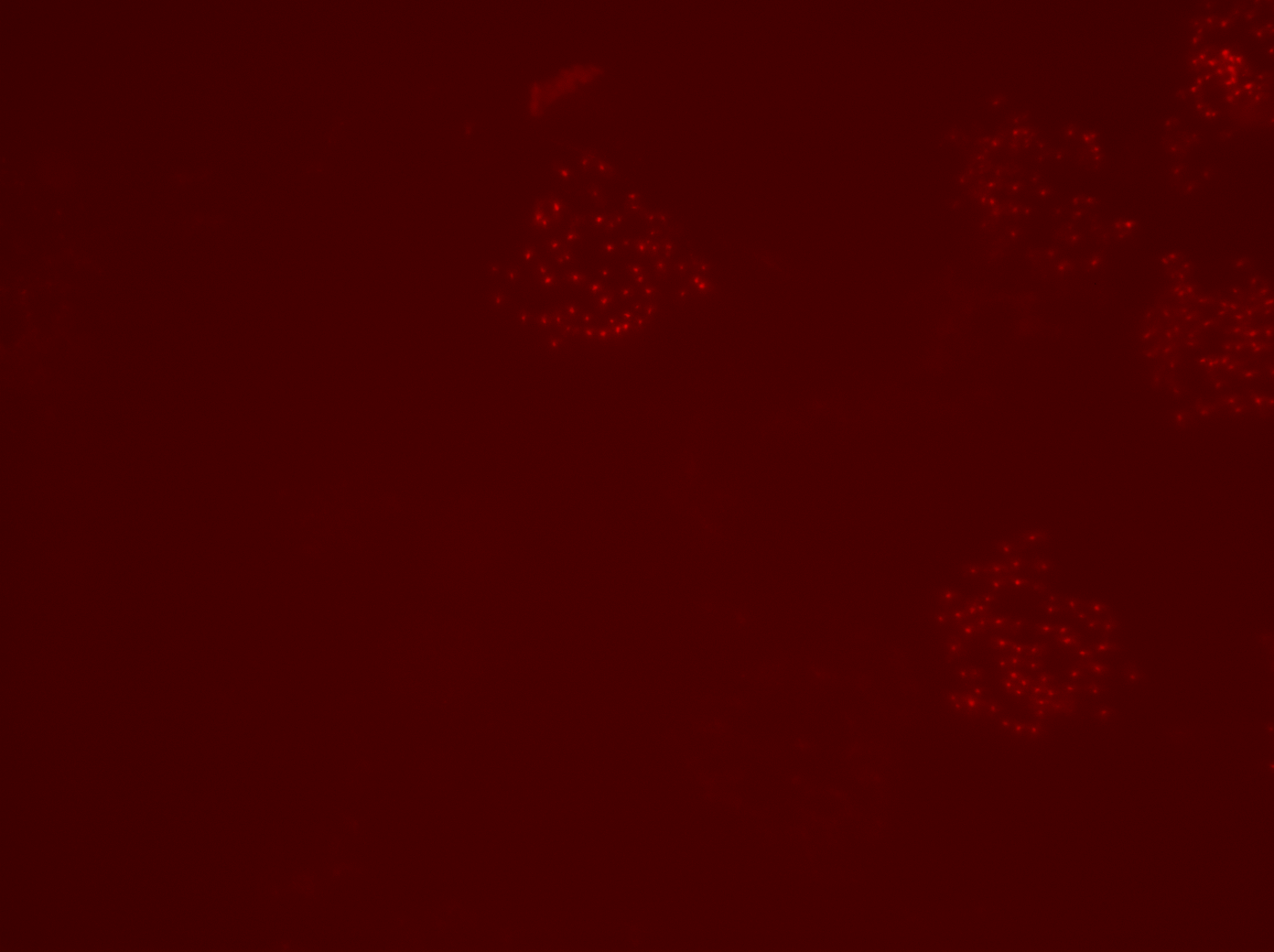

Supplement: Supplementary file 10 — Source data Fig. 4 [file 44318_2025_465_MOESM10_ESM.zip › EMBOJ-2025-120195-Figure 4-Source data/Figure 4/4B/HeLa ATRX.tif]

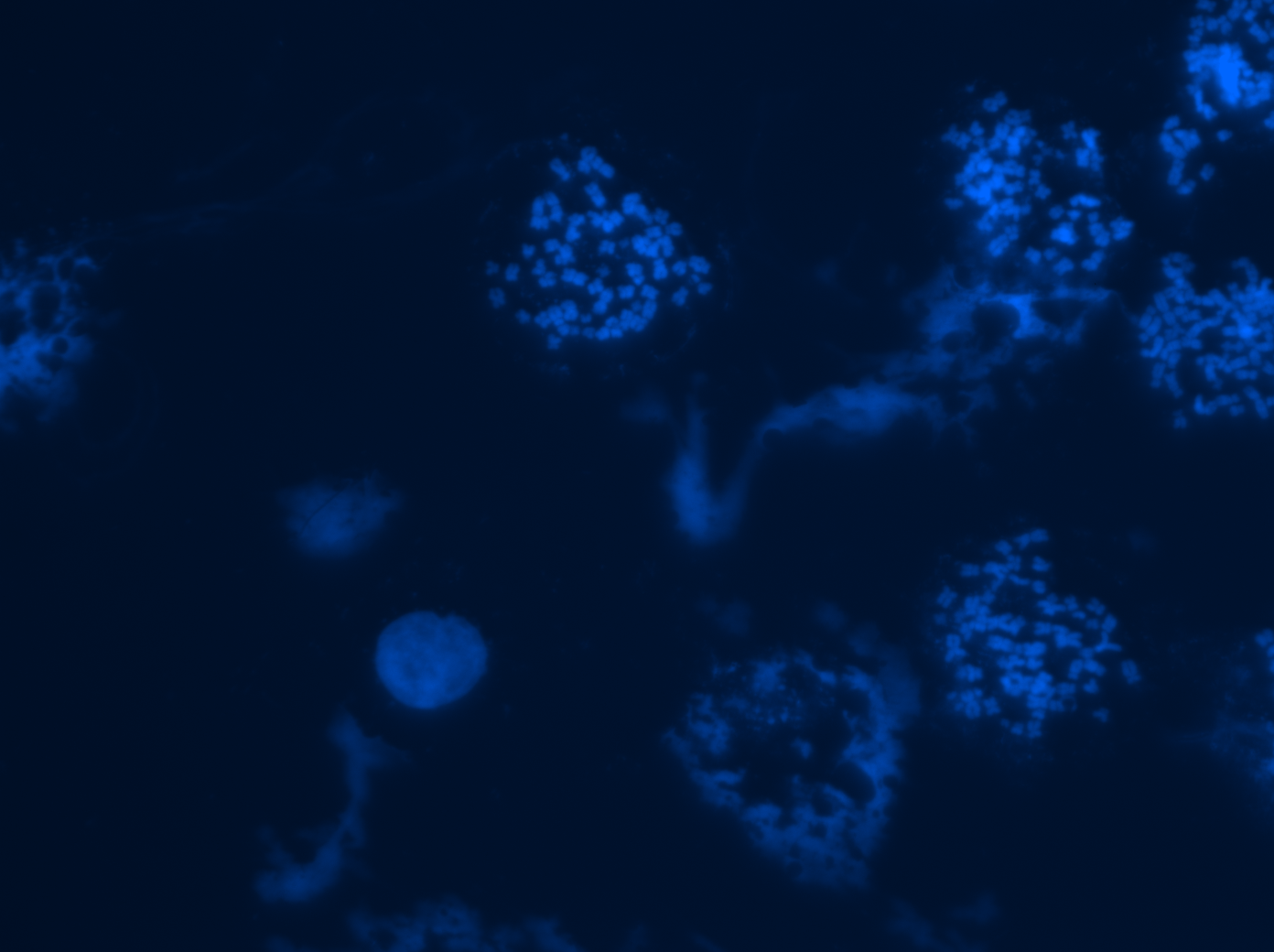

Supplement: Supplementary file 10 — Source data Fig. 4 [file 44318_2025_465_MOESM10_ESM.zip › EMBOJ-2025-120195-Figure 4-Source data/Figure 4/4B/HeLa DNA.tif]

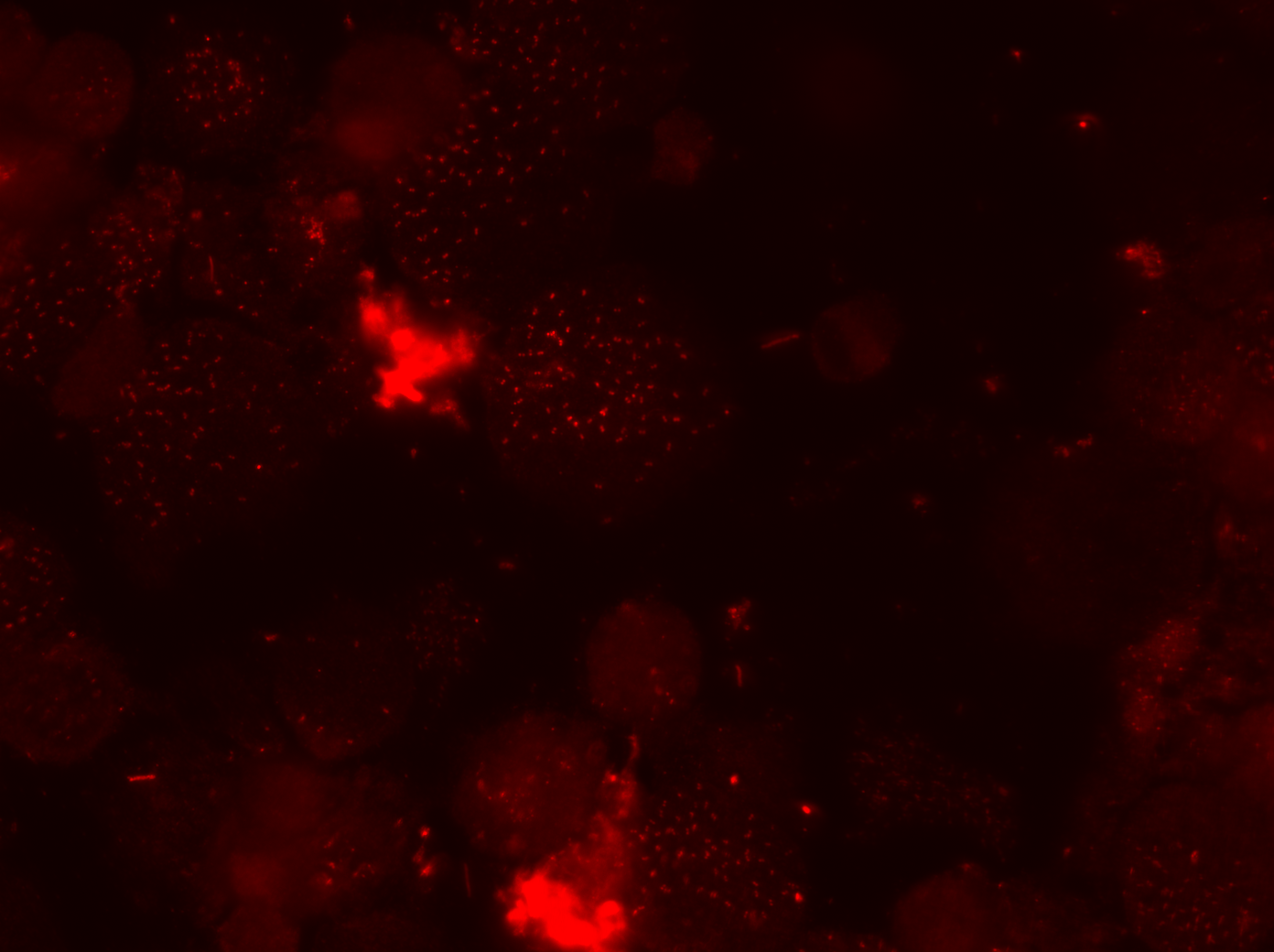

Supplement: Supplementary file 10 — Source data Fig. 4 [file 44318_2025_465_MOESM10_ESM.zip › EMBOJ-2025-120195-Figure 4-Source data/Figure 4/4B/HP1α γ DKO ACA.tif]

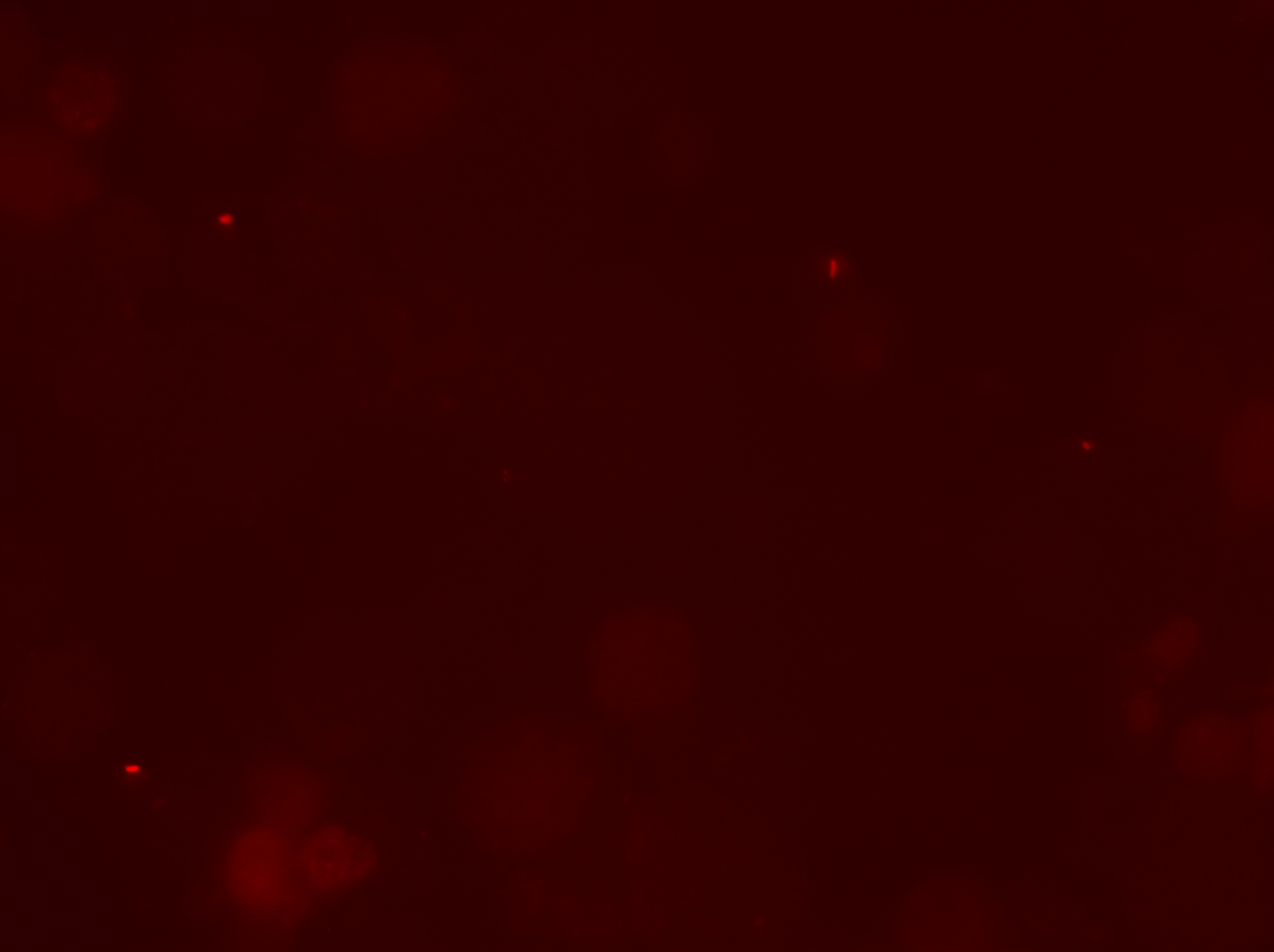

Supplement: Supplementary file 10 — Source data Fig. 4 [file 44318_2025_465_MOESM10_ESM.zip › EMBOJ-2025-120195-Figure 4-Source data/Figure 4/4B/HP1α γ DKO ATRX.tif]

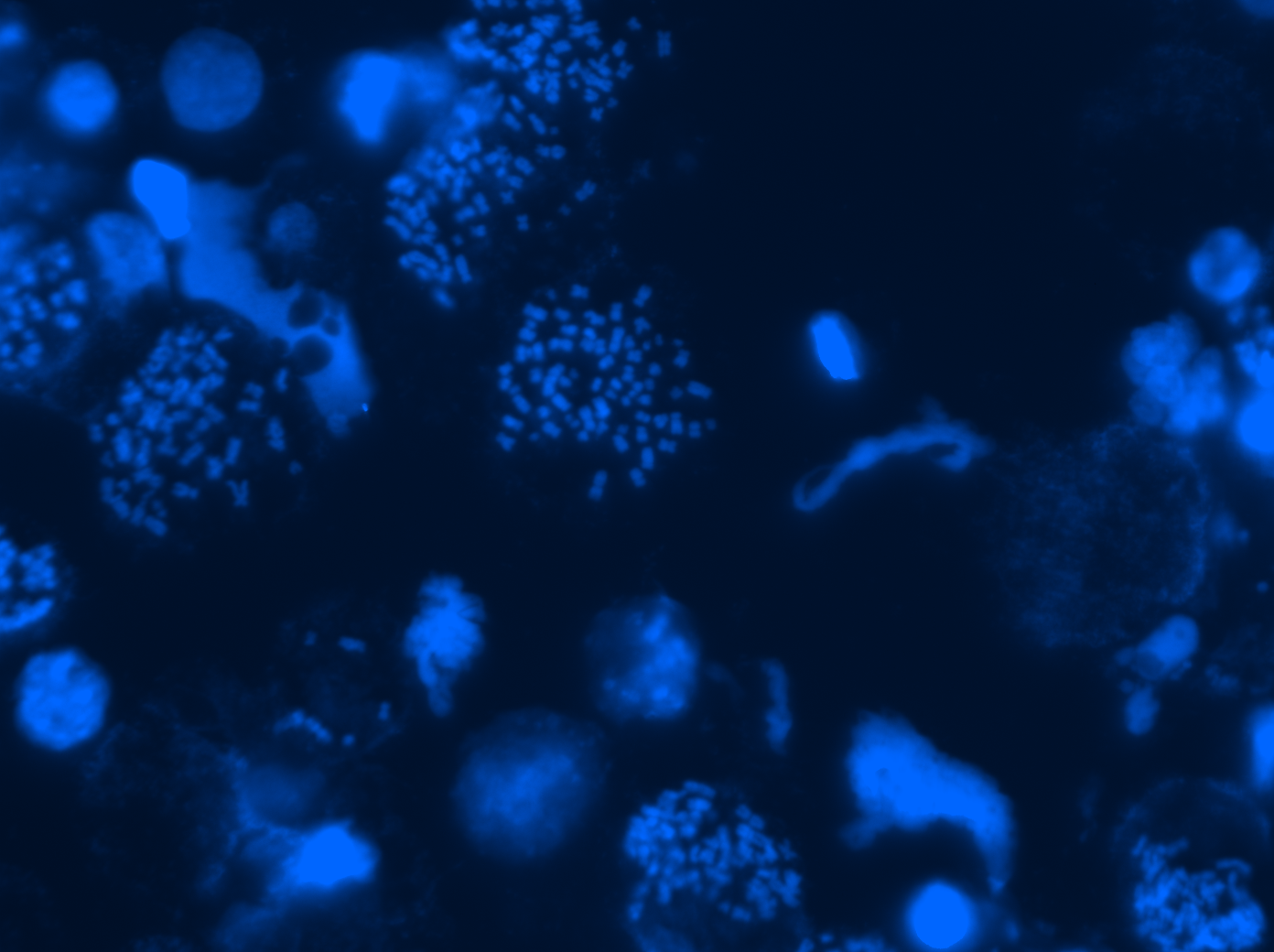

Supplement: Supplementary file 10 — Source data Fig. 4 [file 44318_2025_465_MOESM10_ESM.zip › EMBOJ-2025-120195-Figure 4-Source data/Figure 4/4B/HP1α γ DKO DNA.tif]

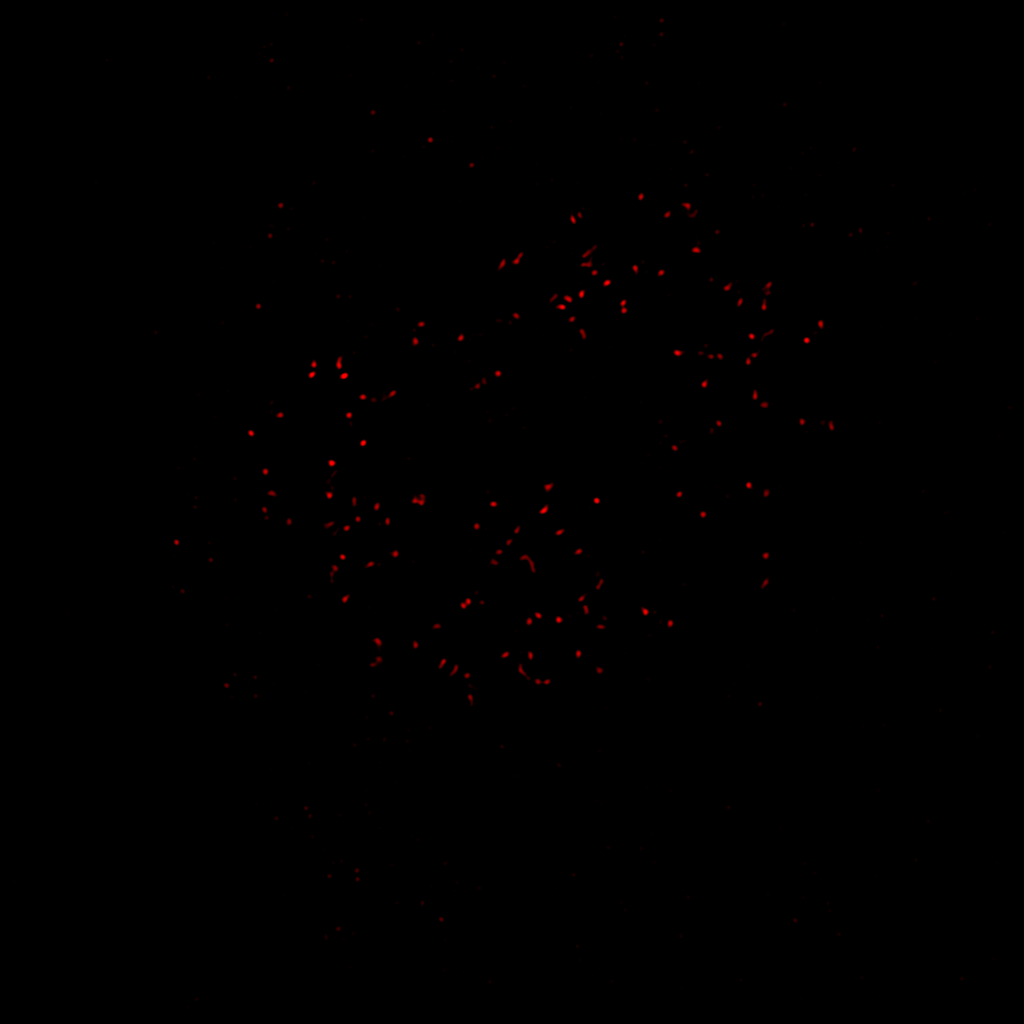

Supplement: Supplementary file 10 — Source data Fig. 4 [file 44318_2025_465_MOESM10_ESM.zip › EMBOJ-2025-120195-Figure 4-Source data/Figure 4/4C/siATRX#1 ACA.tif]

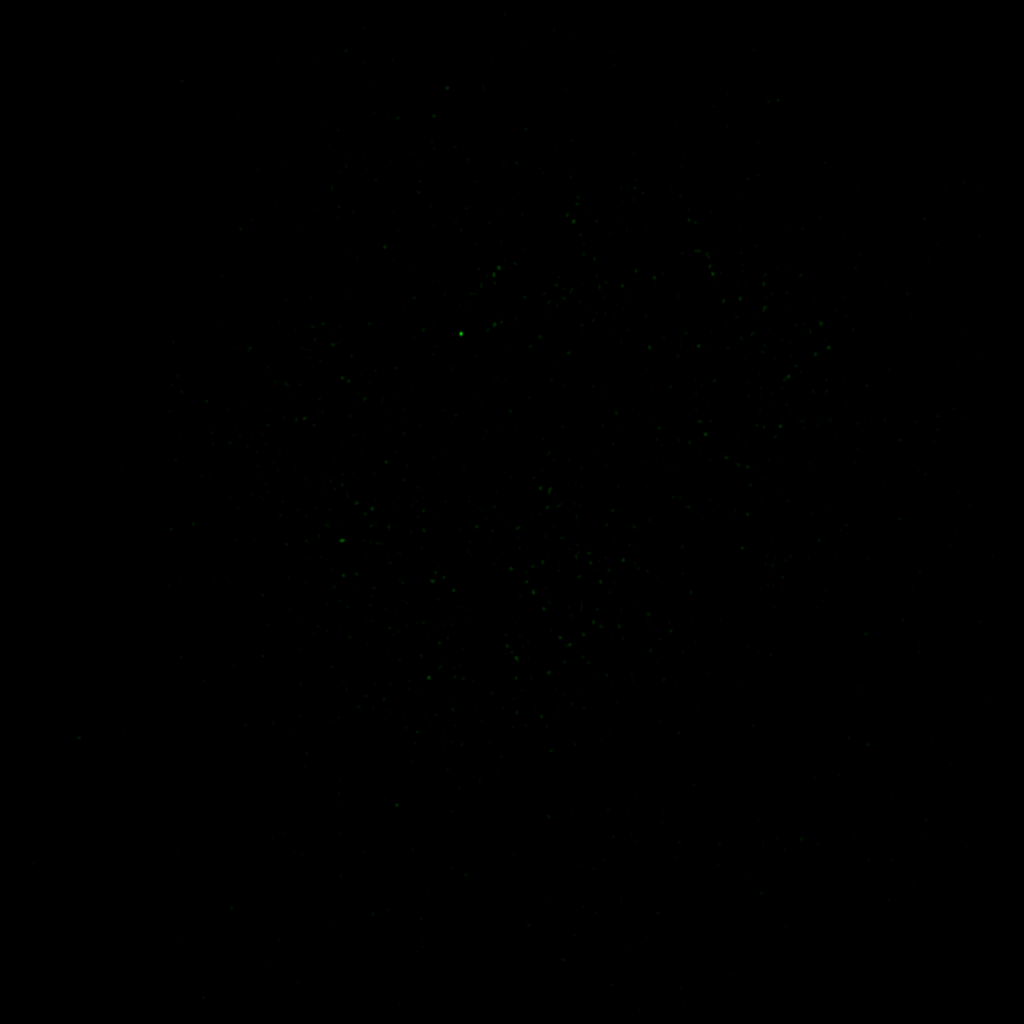

Supplement: Supplementary file 10 — Source data Fig. 4 [file 44318_2025_465_MOESM10_ESM.zip › EMBOJ-2025-120195-Figure 4-Source data/Figure 4/4C/siATRX#1 ATRX.tif]

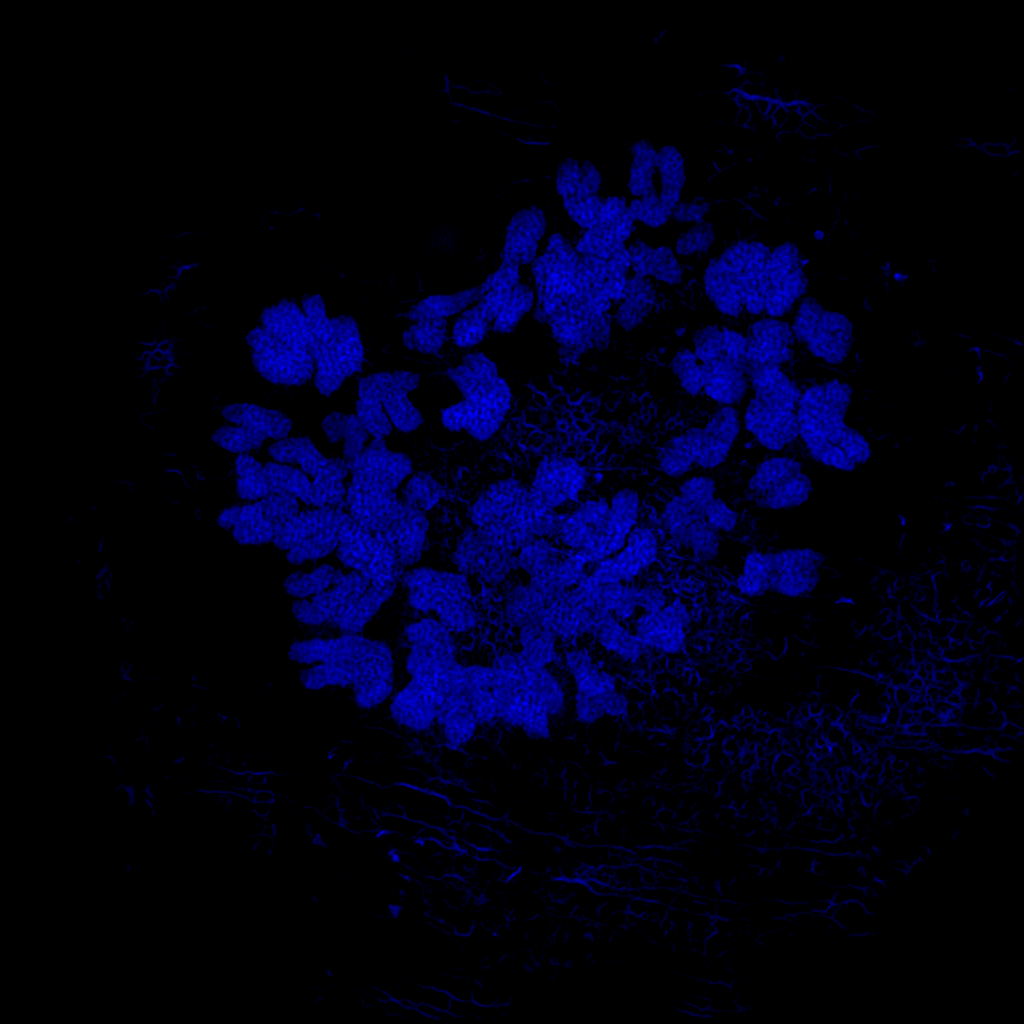

Supplement: Supplementary file 10 — Source data Fig. 4 [file 44318_2025_465_MOESM10_ESM.zip › EMBOJ-2025-120195-Figure 4-Source data/Figure 4/4C/siATRX#1 DNA.tif]

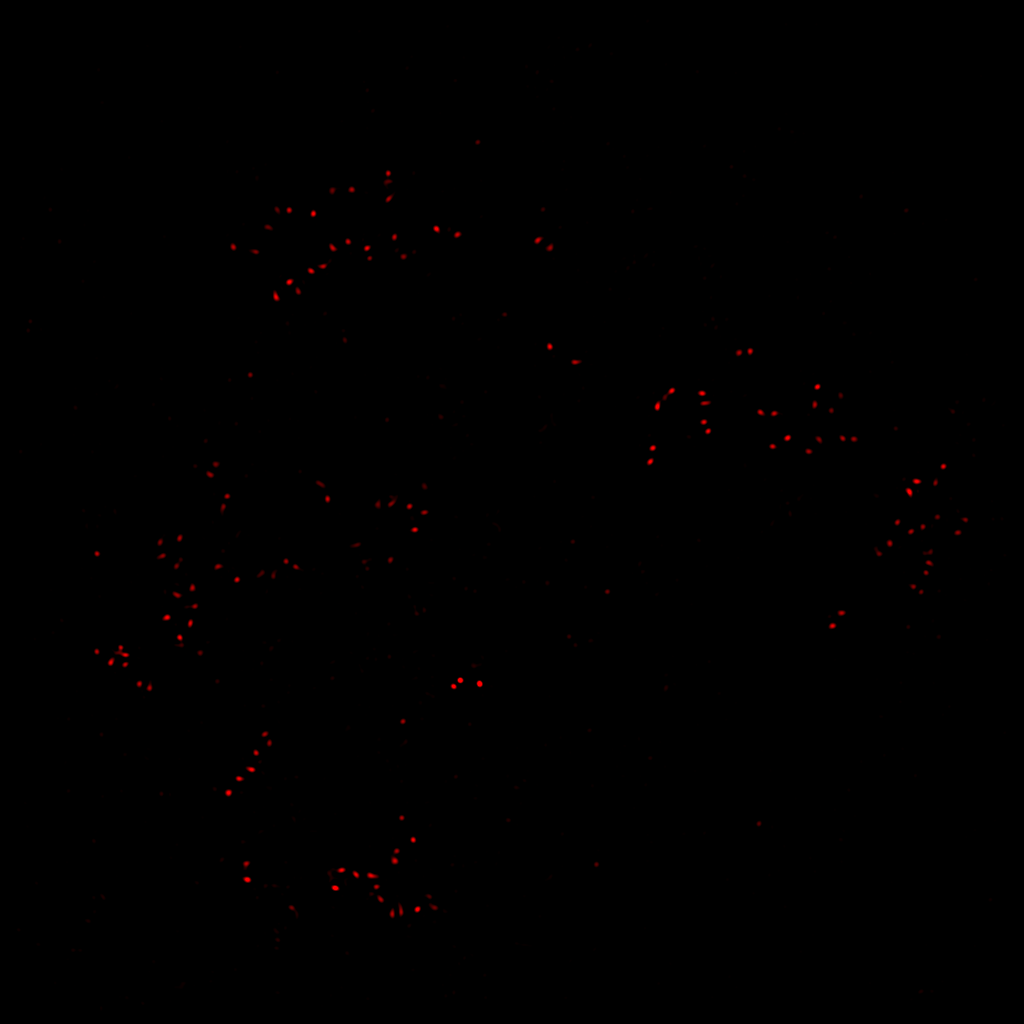

Supplement: Supplementary file 10 — Source data Fig. 4 [file 44318_2025_465_MOESM10_ESM.zip › EMBOJ-2025-120195-Figure 4-Source data/Figure 4/4C/siControl ACA.tif]

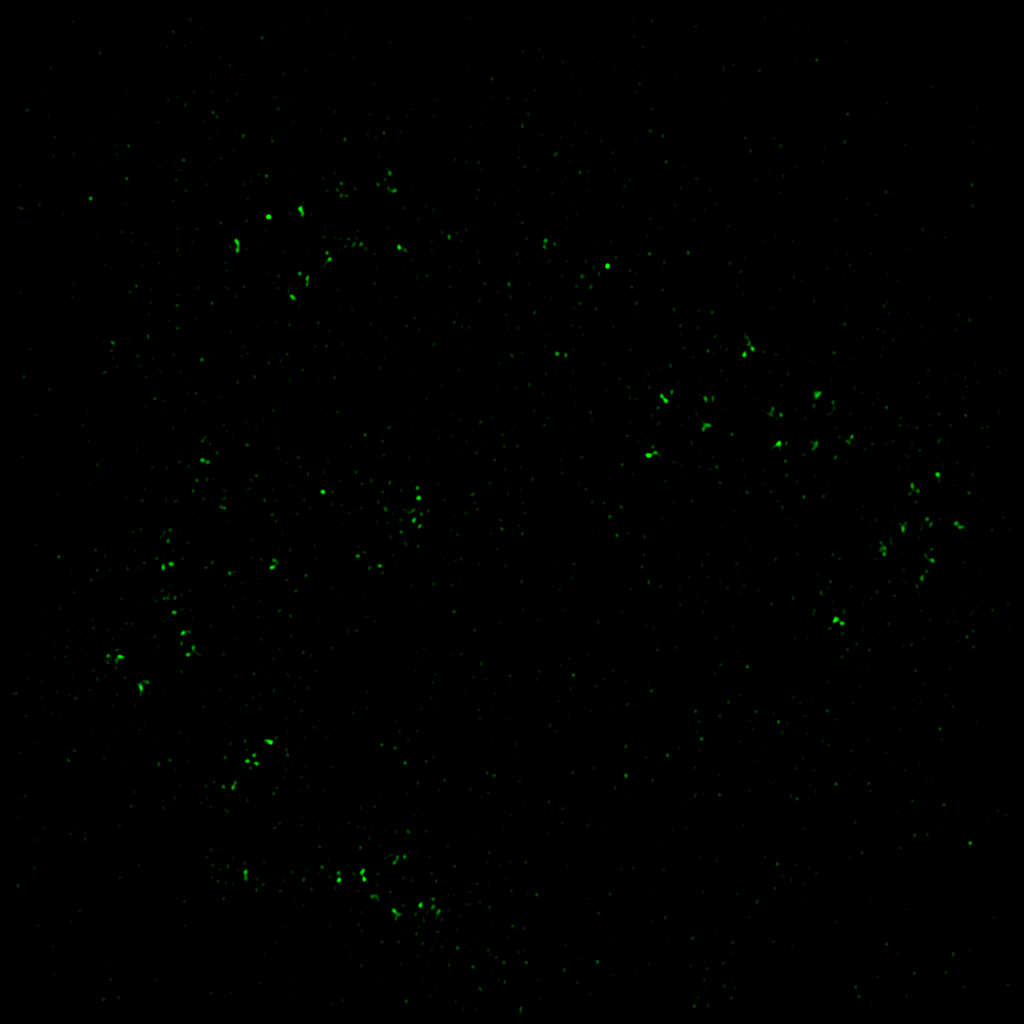

Supplement: Supplementary file 10 — Source data Fig. 4 [file 44318_2025_465_MOESM10_ESM.zip › EMBOJ-2025-120195-Figure 4-Source data/Figure 4/4C/siControl ATRX.tif]

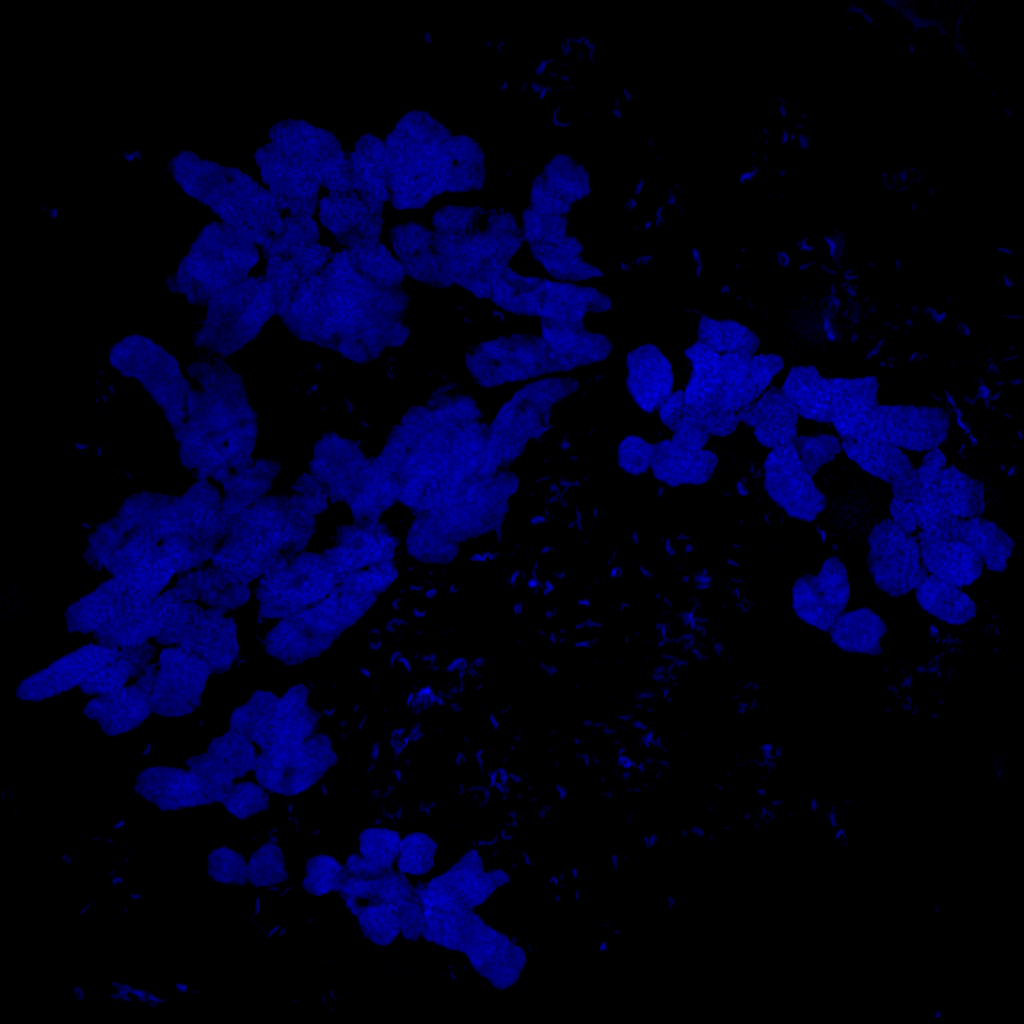

Supplement: Supplementary file 10 — Source data Fig. 4 [file 44318_2025_465_MOESM10_ESM.zip › EMBOJ-2025-120195-Figure 4-Source data/Figure 4/4C/siControl DNA.tif]

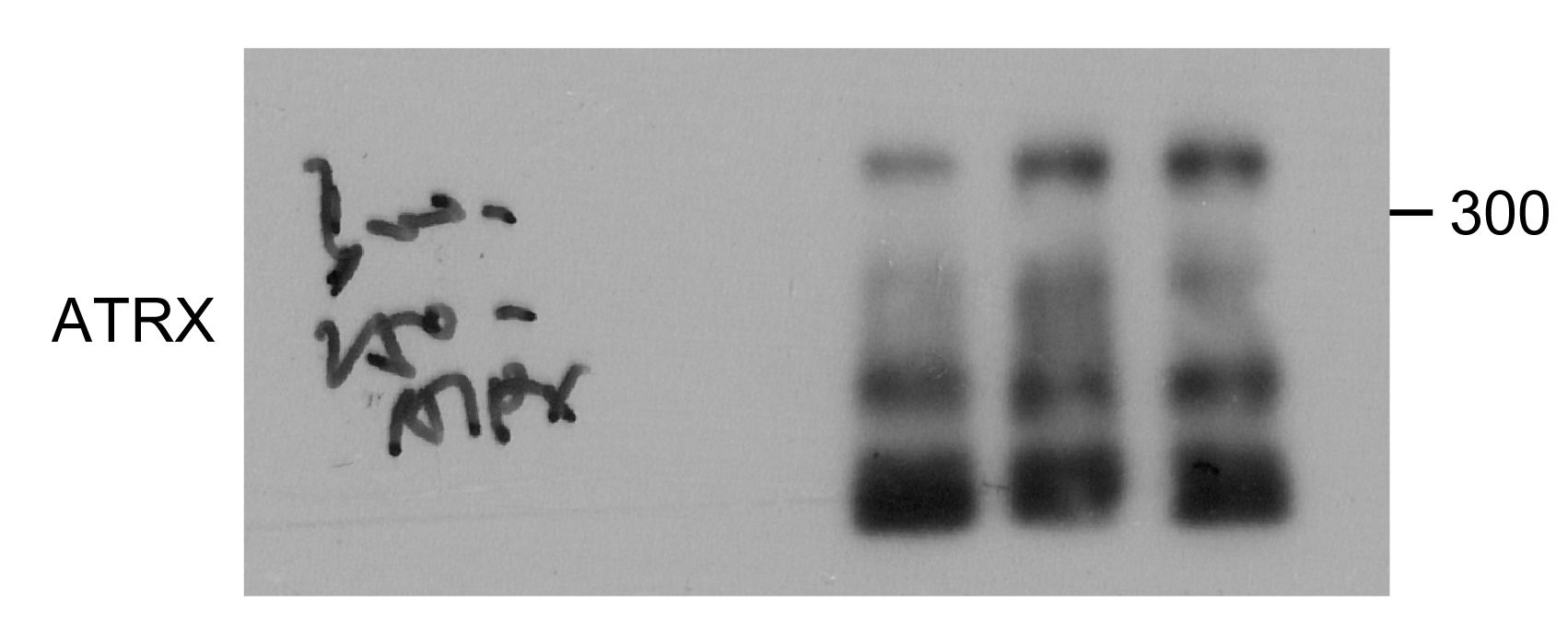

Supplement: Supplementary file 10 — Source data Fig. 4 [file 44318_2025_465_MOESM10_ESM.zip › EMBOJ-2025-120195-Figure 4-Source data/Figure 4/4E/western ATRX.tif]

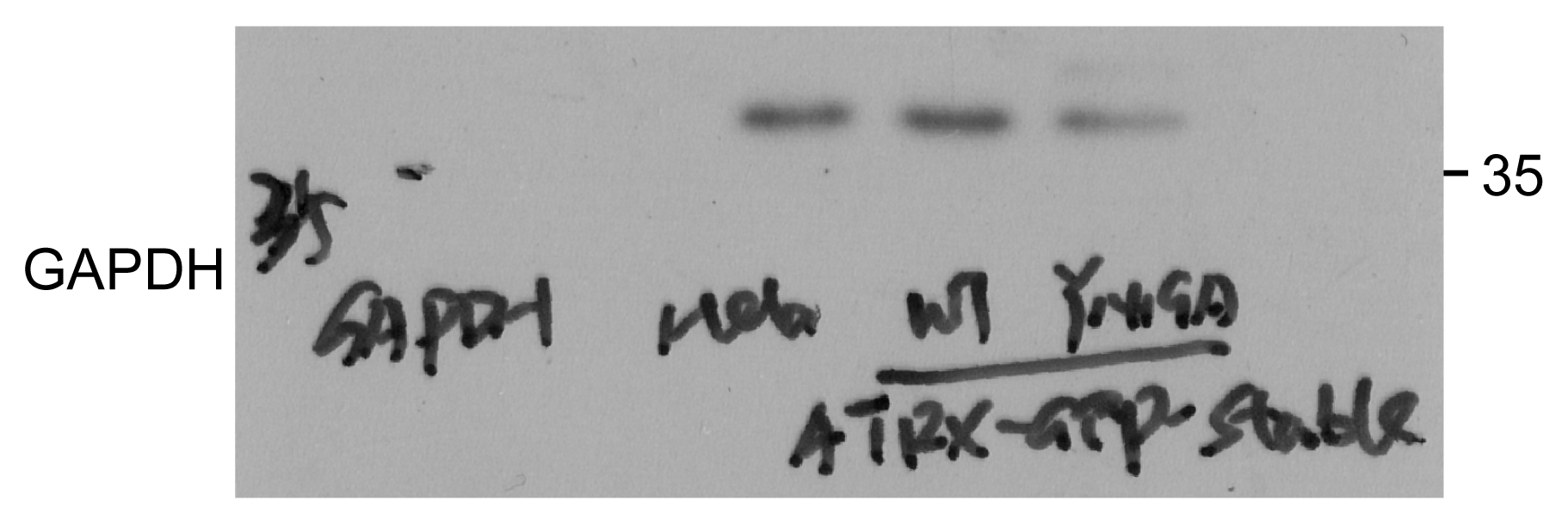

Supplement: Supplementary file 10 — Source data Fig. 4 [file 44318_2025_465_MOESM10_ESM.zip › EMBOJ-2025-120195-Figure 4-Source data/Figure 4/4E/western GAPDH.tif]

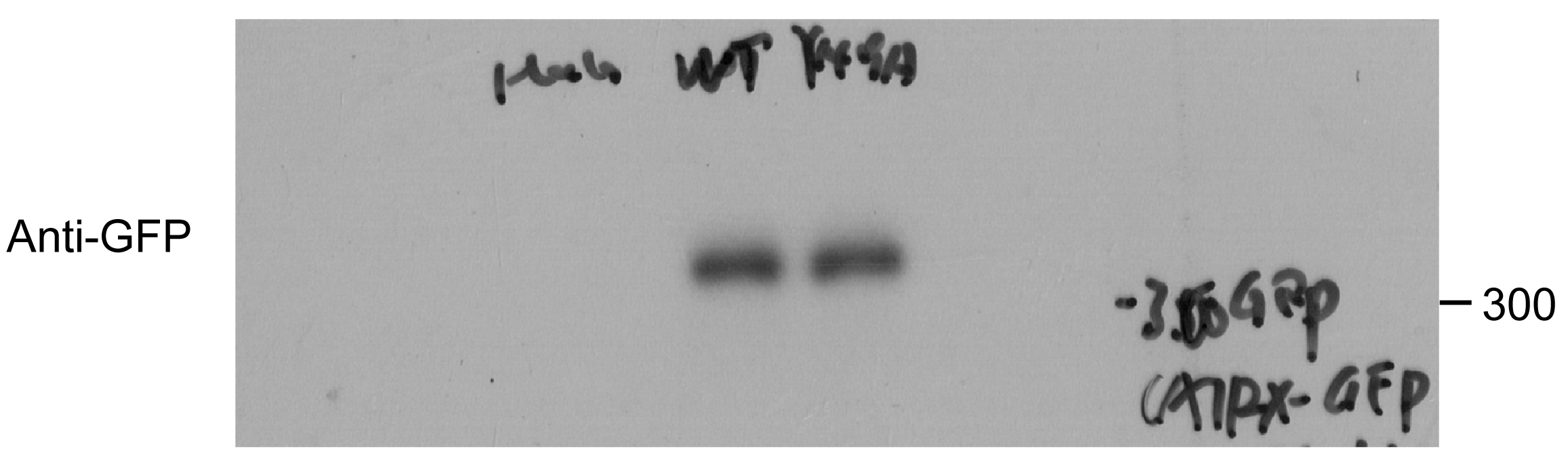

Supplement: Supplementary file 10 — Source data Fig. 4 [file 44318_2025_465_MOESM10_ESM.zip › EMBOJ-2025-120195-Figure 4-Source data/Figure 4/4E/western GFP.tif]

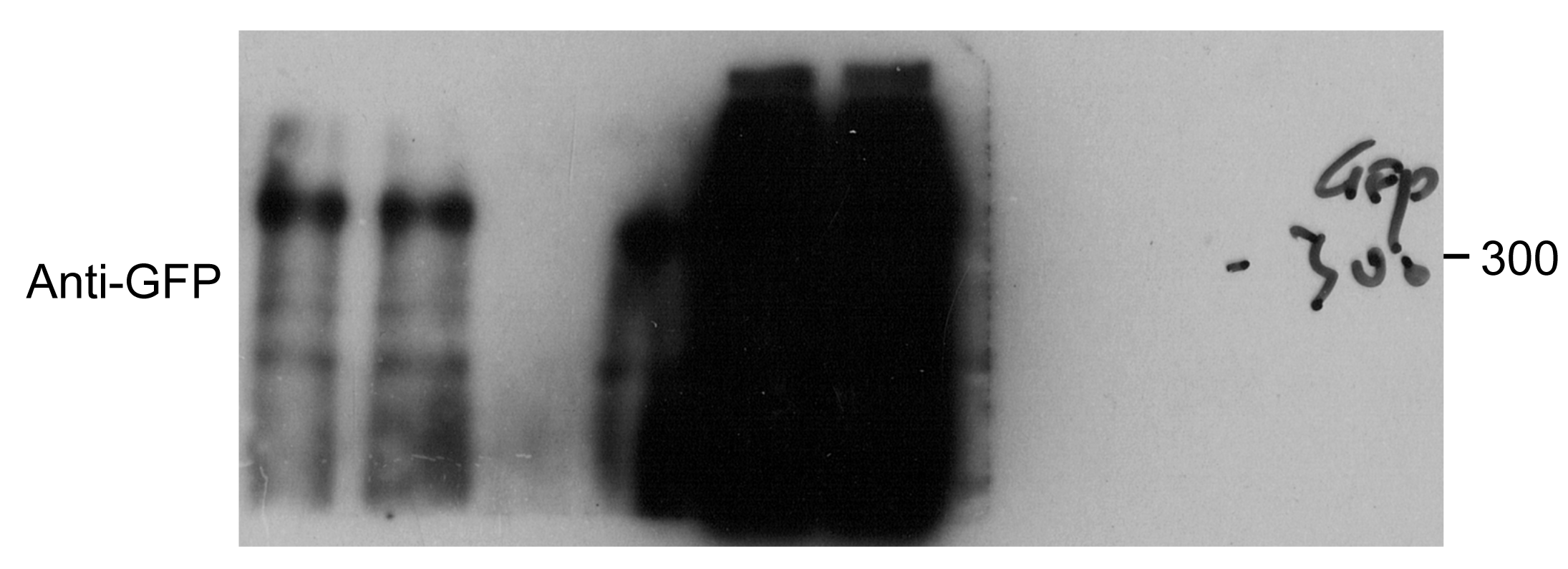

Supplement: Supplementary file 10 — Source data Fig. 4 [file 44318_2025_465_MOESM10_ESM.zip › EMBOJ-2025-120195-Figure 4-Source data/Figure 4/4F/western GFP L.exp..tif]

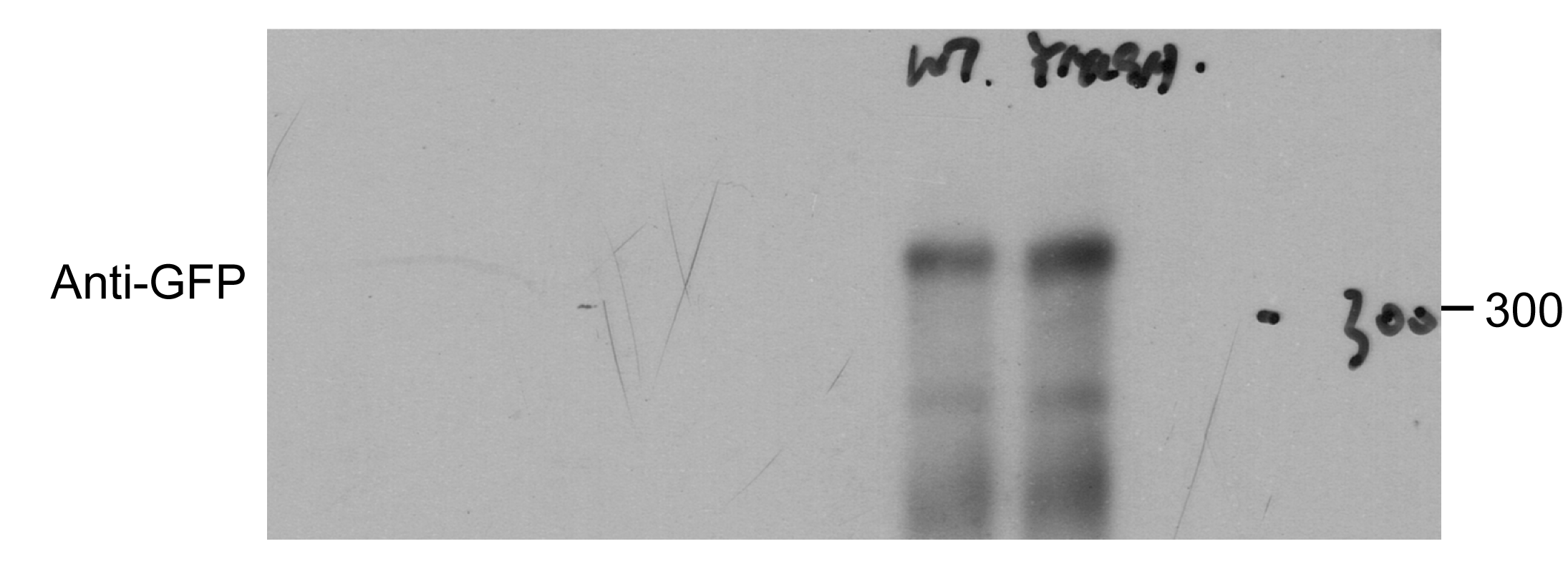

Supplement: Supplementary file 10 — Source data Fig. 4 [file 44318_2025_465_MOESM10_ESM.zip › EMBOJ-2025-120195-Figure 4-Source data/Figure 4/4F/western GFP S.exp..tif]

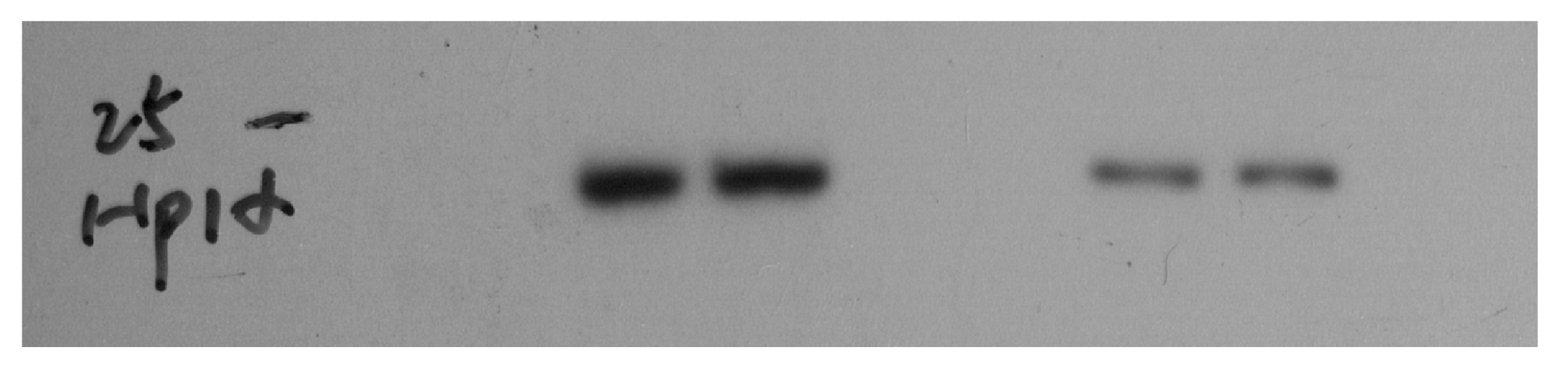

Supplement: Supplementary file 10 — Source data Fig. 4 [file 44318_2025_465_MOESM10_ESM.zip › EMBOJ-2025-120195-Figure 4-Source data/Figure 4/4F/western HP1α.tif]

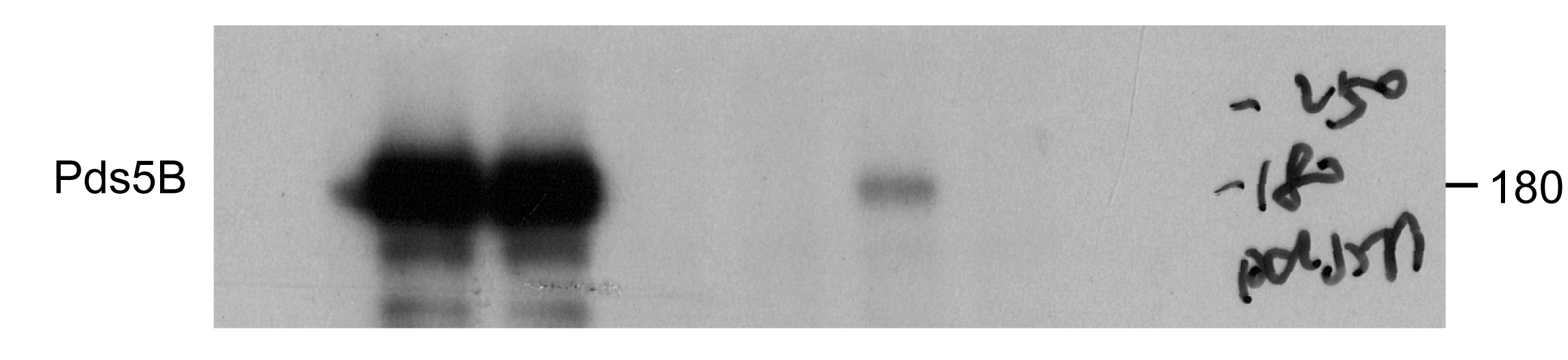

Supplement: Supplementary file 10 — Source data Fig. 4 [file 44318_2025_465_MOESM10_ESM.zip › EMBOJ-2025-120195-Figure 4-Source data/Figure 4/4F/western Pds5B.tif]

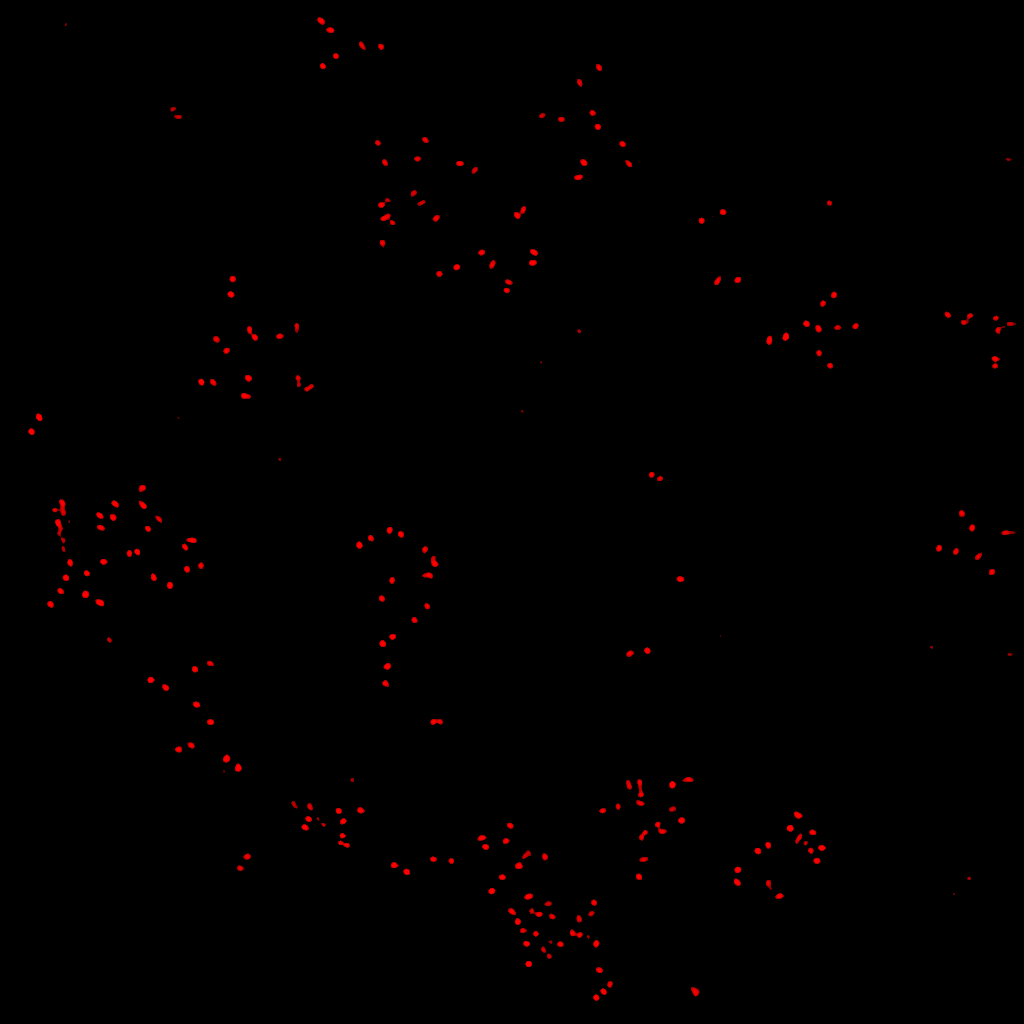

Supplement: Supplementary file 10 — Source data Fig. 4 [file 44318_2025_465_MOESM10_ESM.zip › EMBOJ-2025-120195-Figure 4-Source data/Figure 4/4G/ATRX (Y1419A)-GFP ACA.tif]

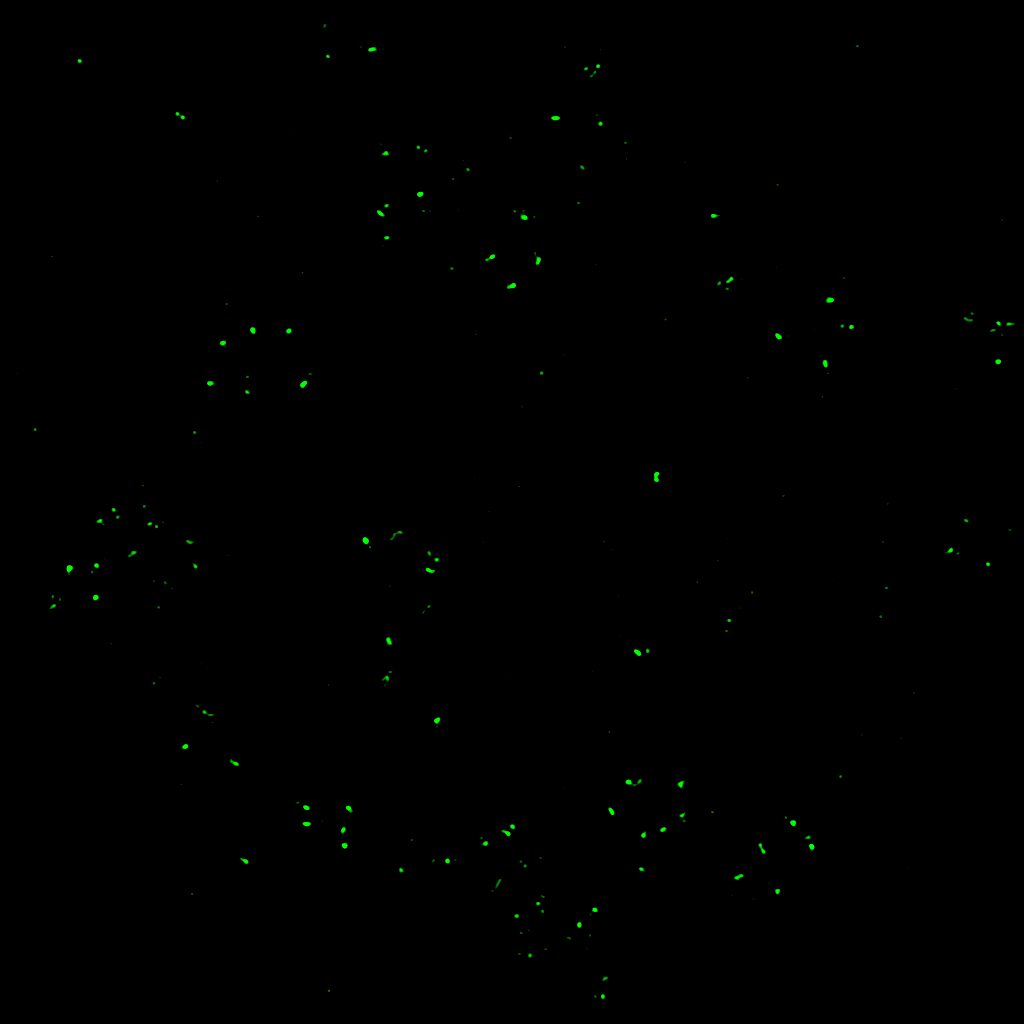

Supplement: Supplementary file 10 — Source data Fig. 4 [file 44318_2025_465_MOESM10_ESM.zip › EMBOJ-2025-120195-Figure 4-Source data/Figure 4/4G/ATRX (Y1419A)-GFP Anti-GFP.tif]

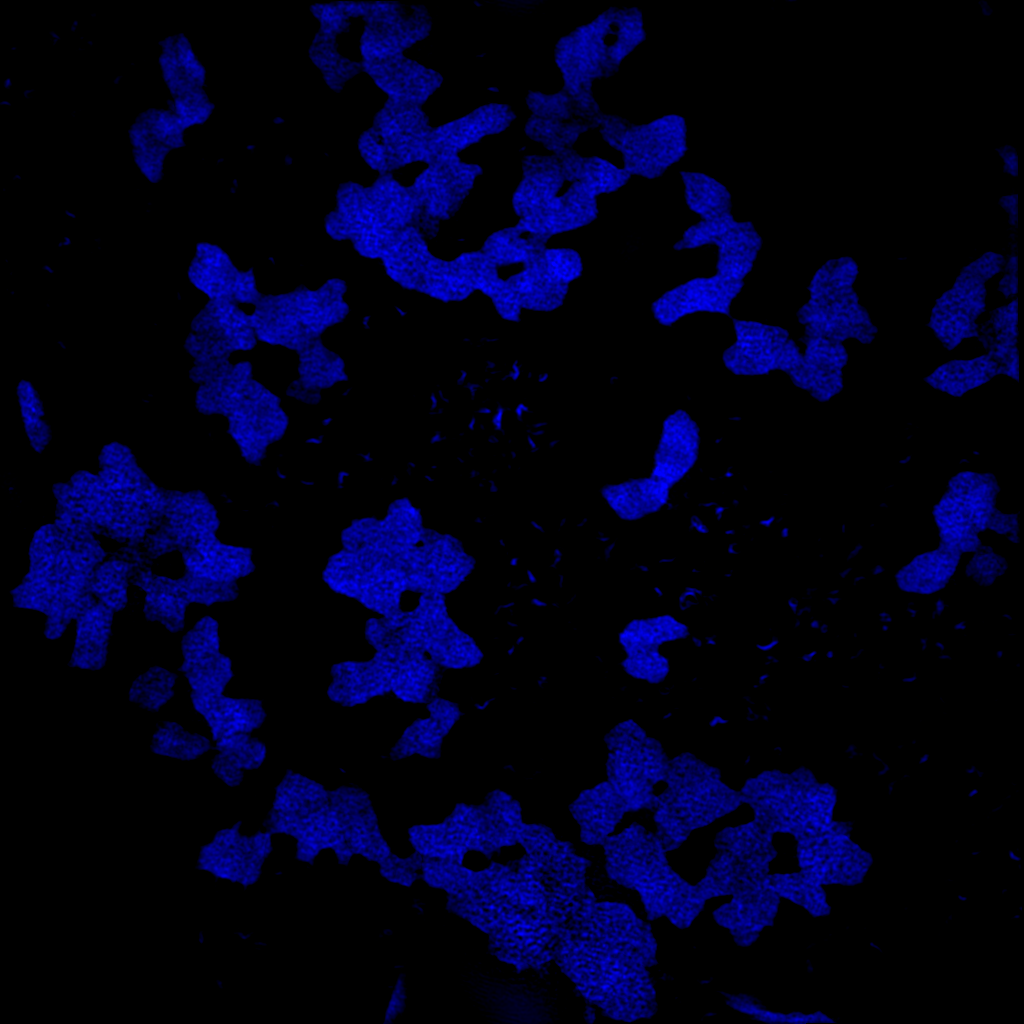

Supplement: Supplementary file 10 — Source data Fig. 4 [file 44318_2025_465_MOESM10_ESM.zip › EMBOJ-2025-120195-Figure 4-Source data/Figure 4/4G/ATRX (Y1419A)-GFP DNA.tif]

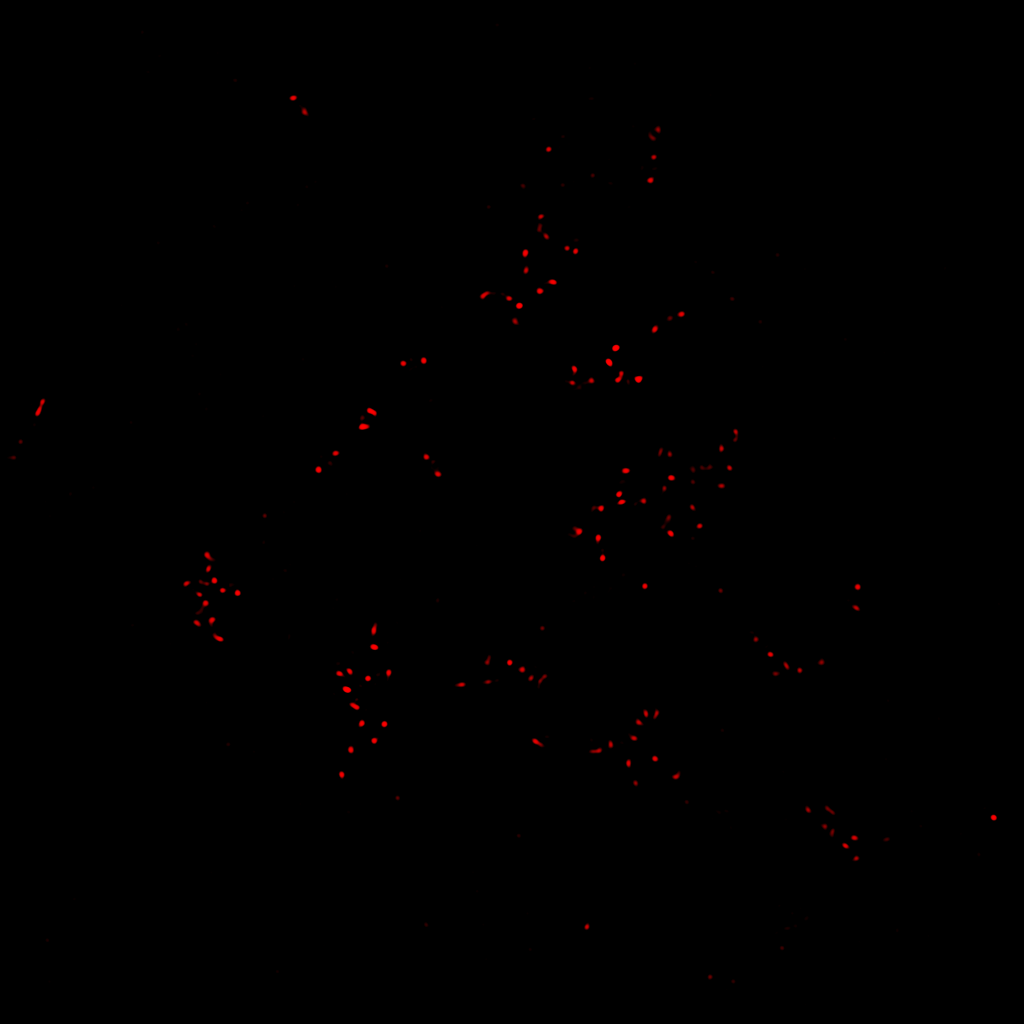

Supplement: Supplementary file 10 — Source data Fig. 4 [file 44318_2025_465_MOESM10_ESM.zip › EMBOJ-2025-120195-Figure 4-Source data/Figure 4/4G/ATRX-GFP ACA.tif]

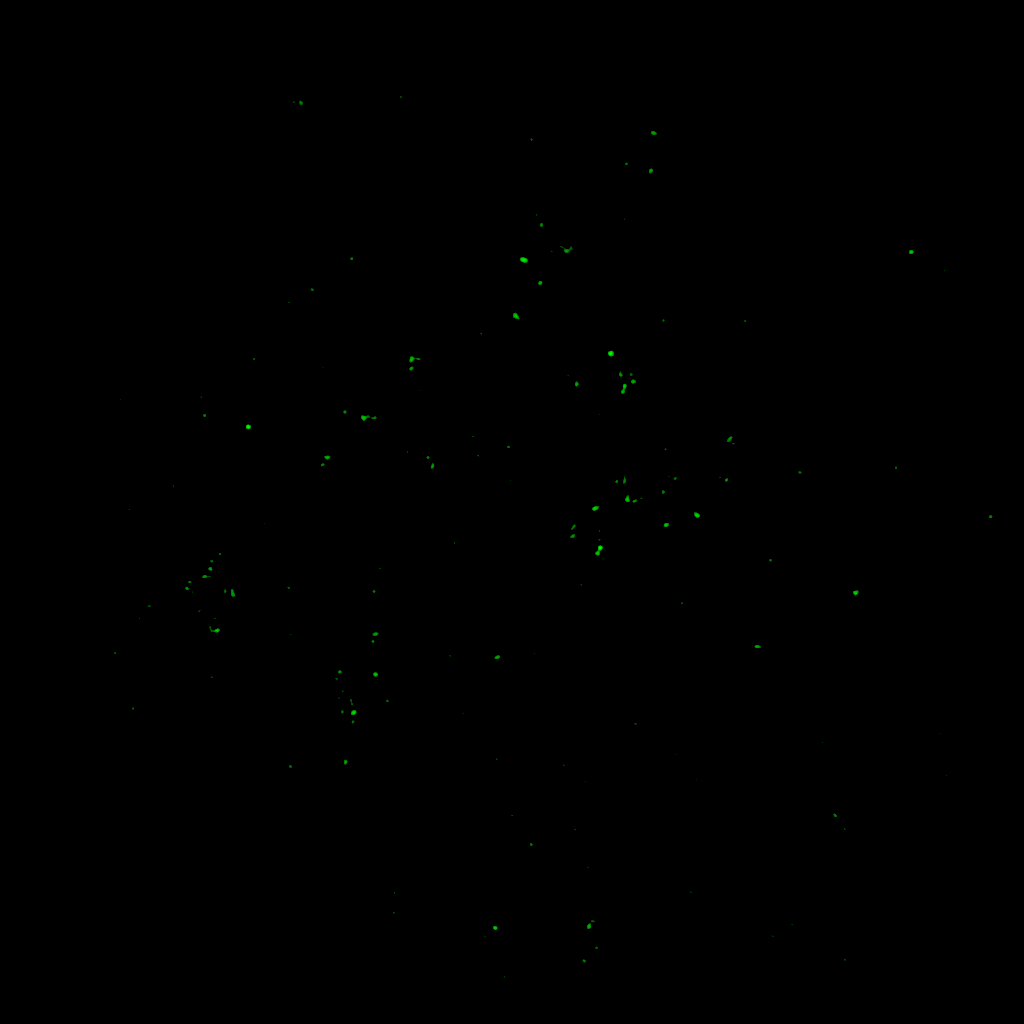

Supplement: Supplementary file 10 — Source data Fig. 4 [file 44318_2025_465_MOESM10_ESM.zip › EMBOJ-2025-120195-Figure 4-Source data/Figure 4/4G/ATRX-GFP Anti-GFP.tif]

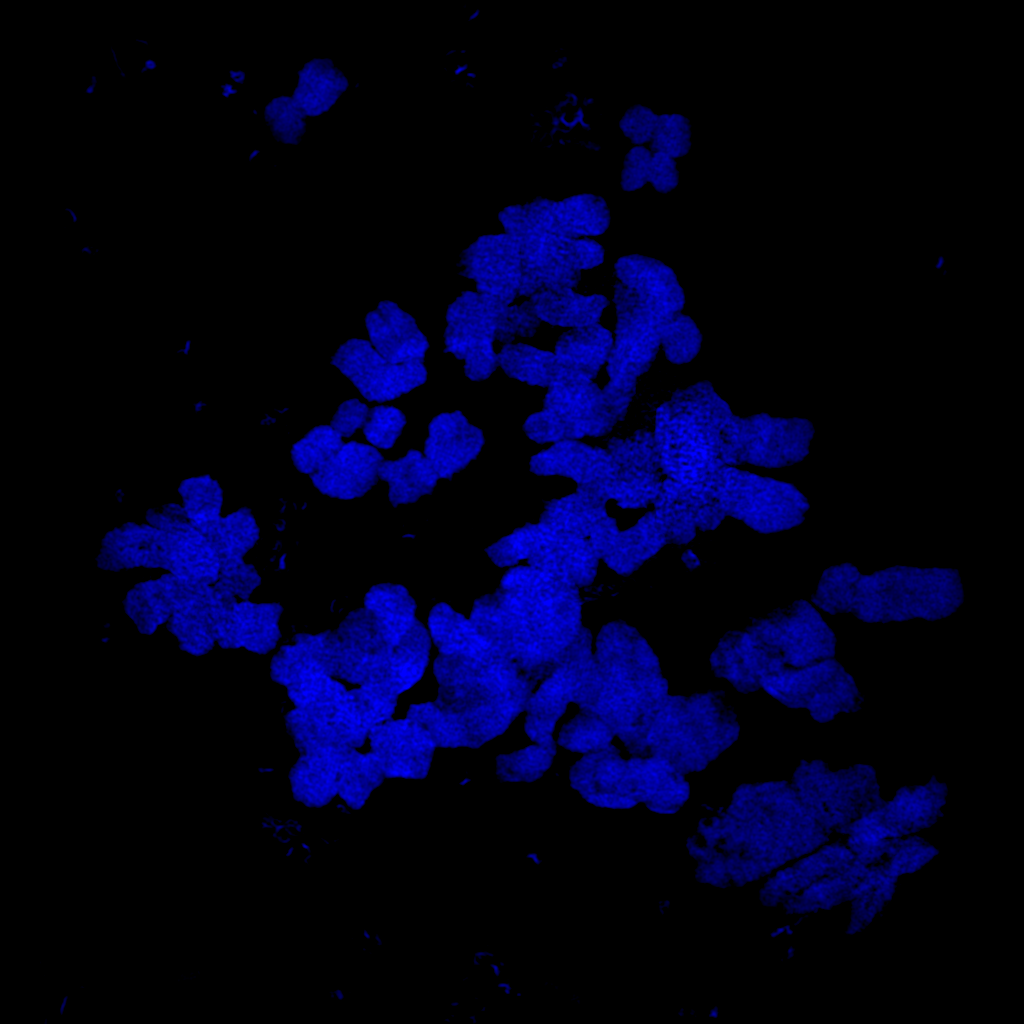

Supplement: Supplementary file 10 — Source data Fig. 4 [file 44318_2025_465_MOESM10_ESM.zip › EMBOJ-2025-120195-Figure 4-Source data/Figure 4/4G/ATRX-GFP DNA.tif]

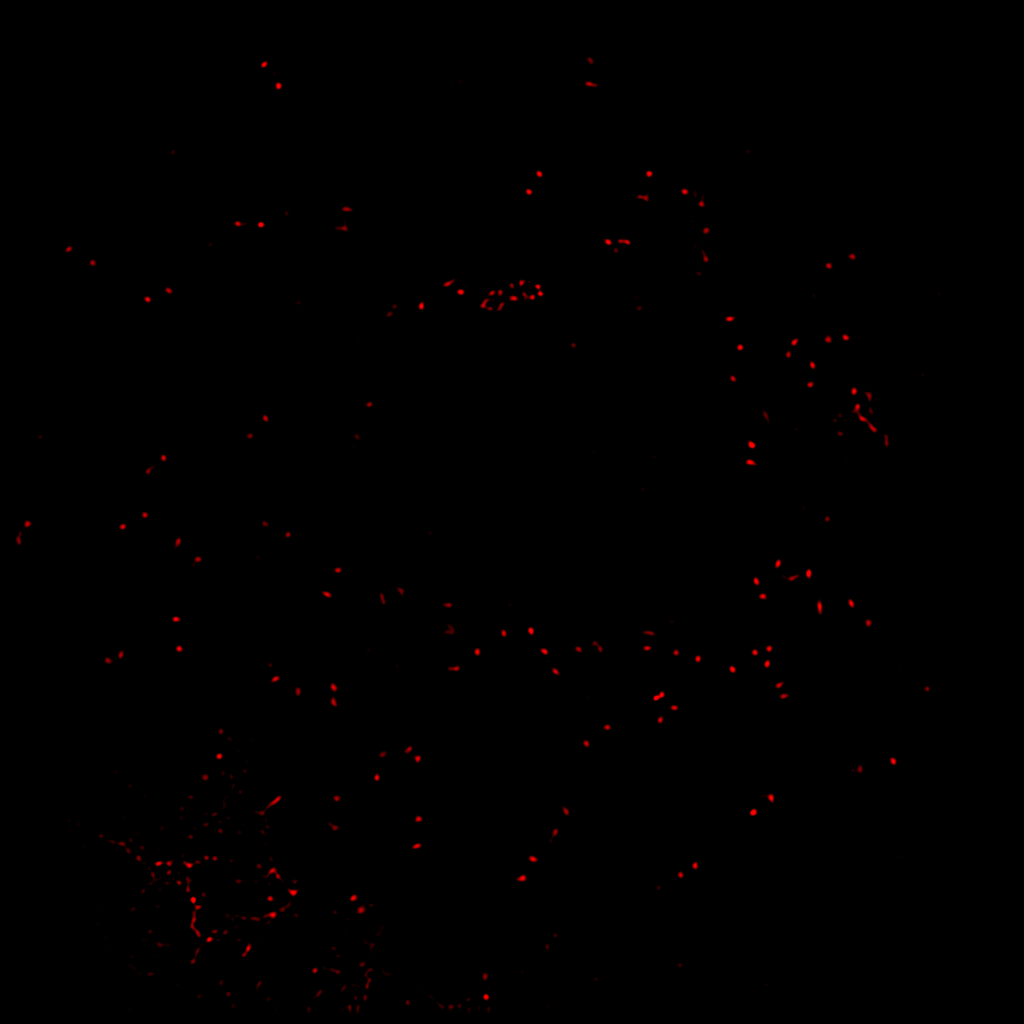

Supplement: Supplementary file 10 — Source data Fig. 4 [file 44318_2025_465_MOESM10_ESM.zip › EMBOJ-2025-120195-Figure 4-Source data/Figure 4/4G/HeLa ACA.tif]

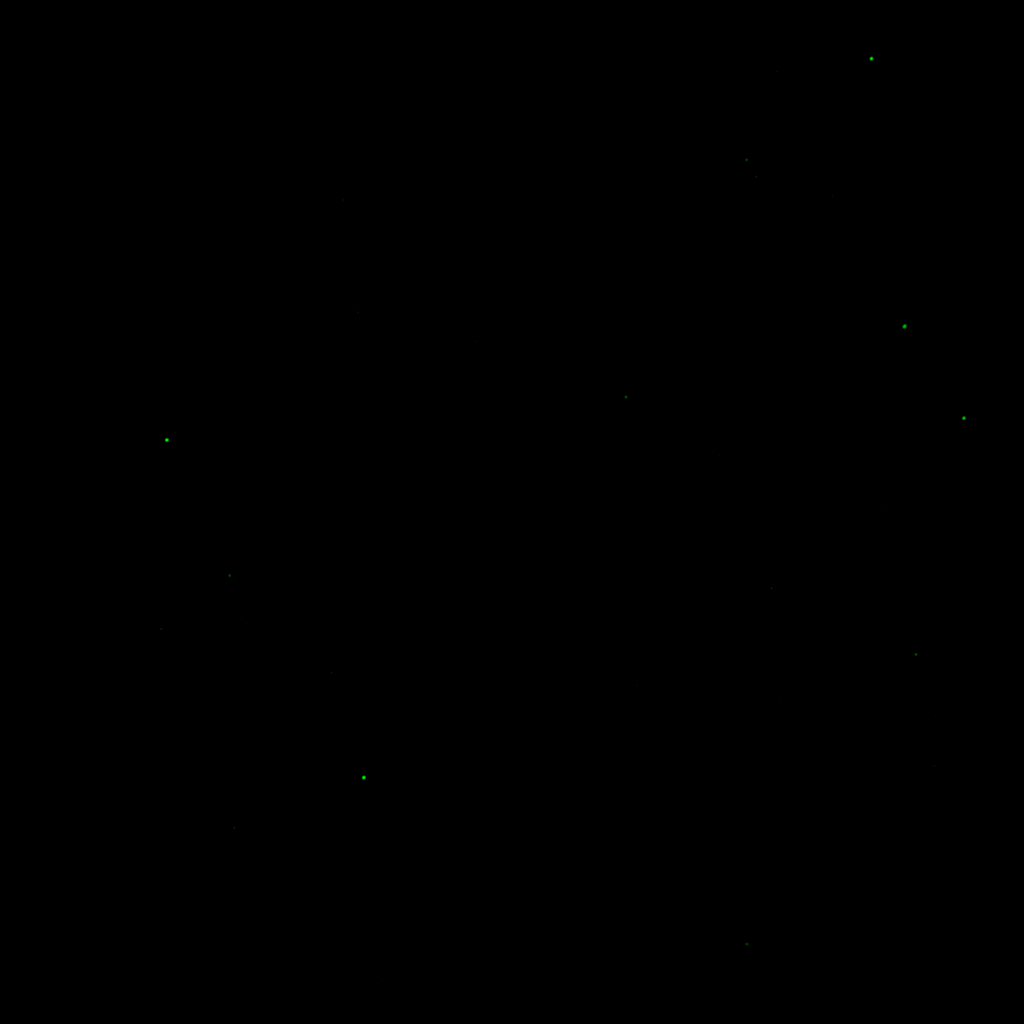

Supplement: Supplementary file 10 — Source data Fig. 4 [file 44318_2025_465_MOESM10_ESM.zip › EMBOJ-2025-120195-Figure 4-Source data/Figure 4/4G/HeLa Anti-GFP.tif]

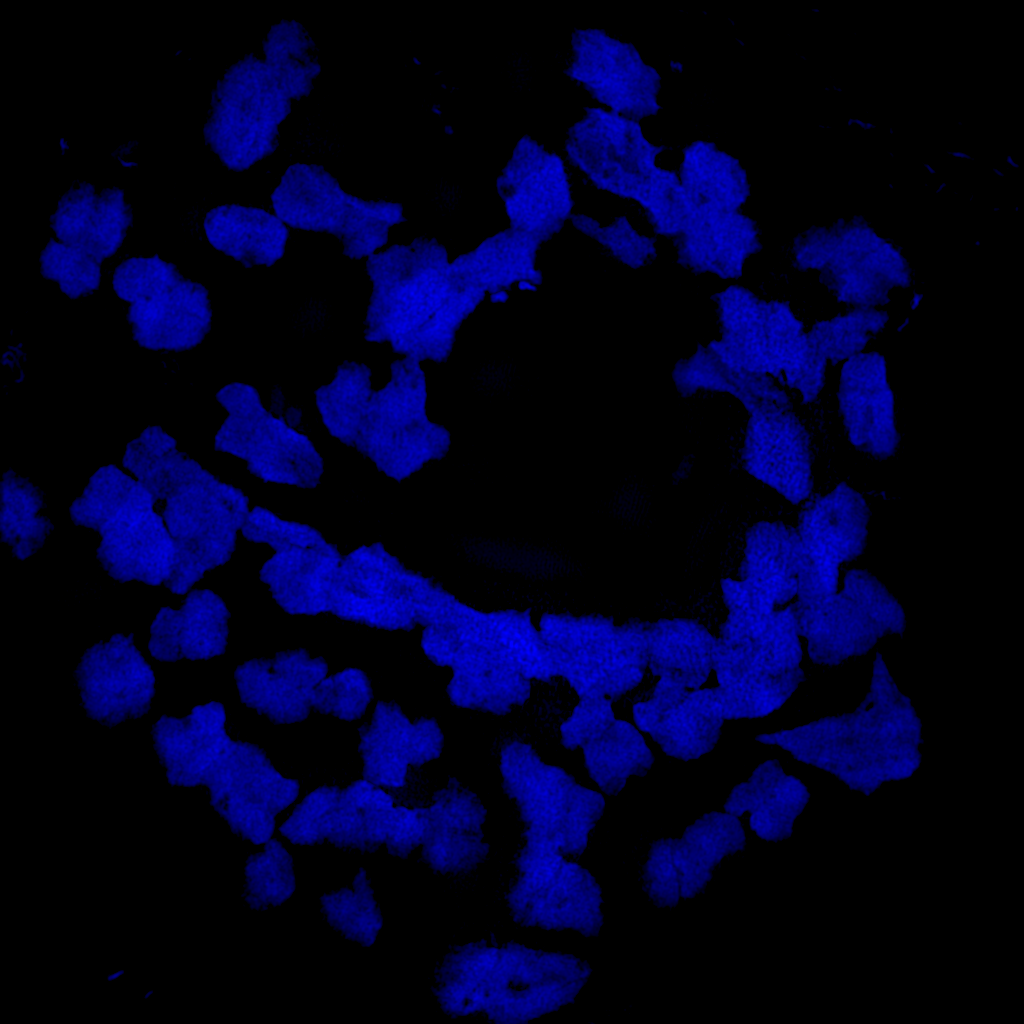

Supplement: Supplementary file 10 — Source data Fig. 4 [file 44318_2025_465_MOESM10_ESM.zip › EMBOJ-2025-120195-Figure 4-Source data/Figure 4/4G/HeLa DNA.tif]

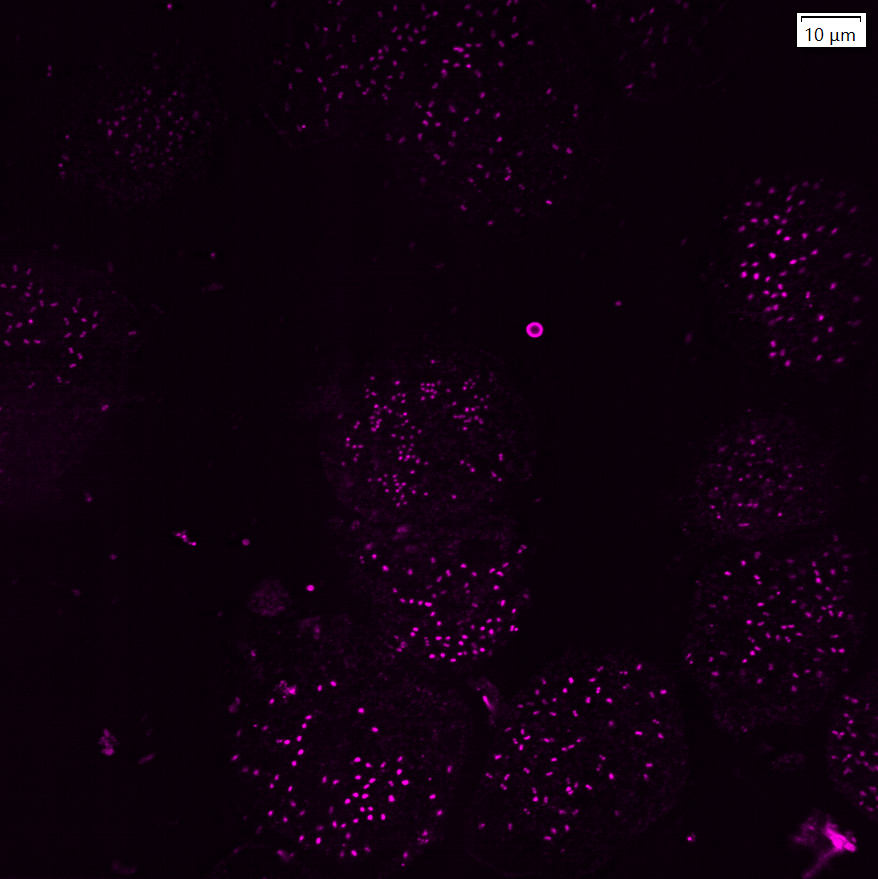

Supplement: Supplementary file 11 — Source data Fig. 5 [file 44318_2025_465_MOESM11_ESM.zip › EMBOJ-2025-120195-Figure 5-Source data/Figure 5/5B/ATRX (Y1419A)-GFP siATRX #1 ACA.tif]

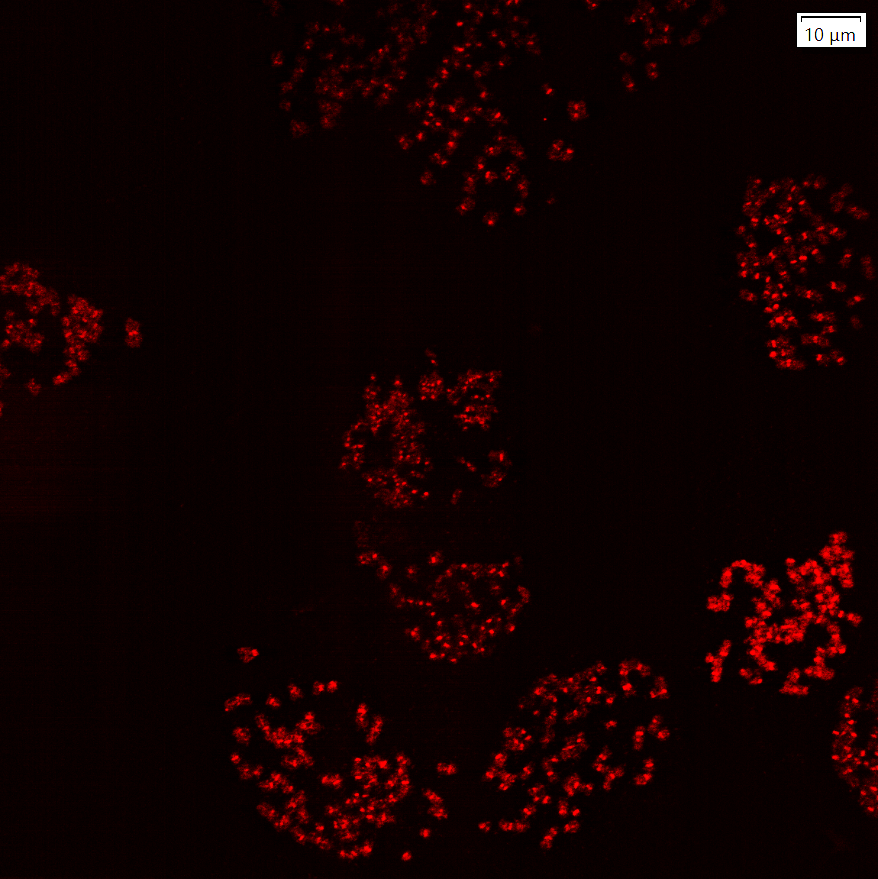

Supplement: Supplementary file 11 — Source data Fig. 5 [file 44318_2025_465_MOESM11_ESM.zip › EMBOJ-2025-120195-Figure 5-Source data/Figure 5/5B/ATRX (Y1419A)-GFP siATRX #1 Anti-GFP.tif]

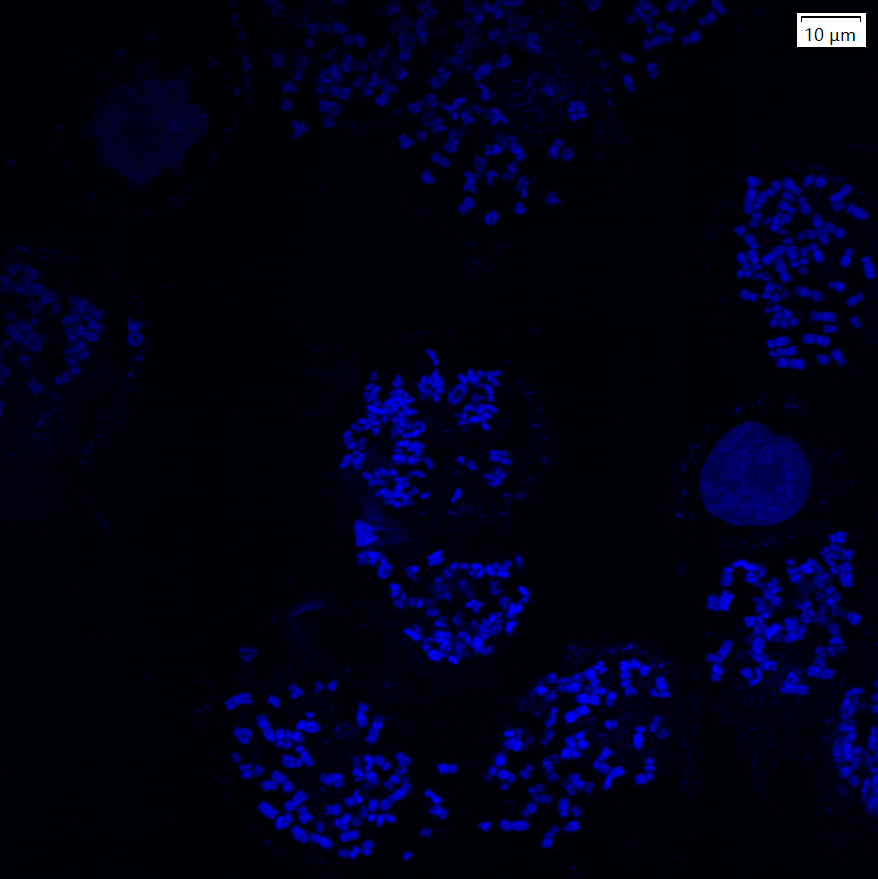

Supplement: Supplementary file 11 — Source data Fig. 5 [file 44318_2025_465_MOESM11_ESM.zip › EMBOJ-2025-120195-Figure 5-Source data/Figure 5/5B/ATRX (Y1419A)-GFP siATRX #1 DNA.tif]

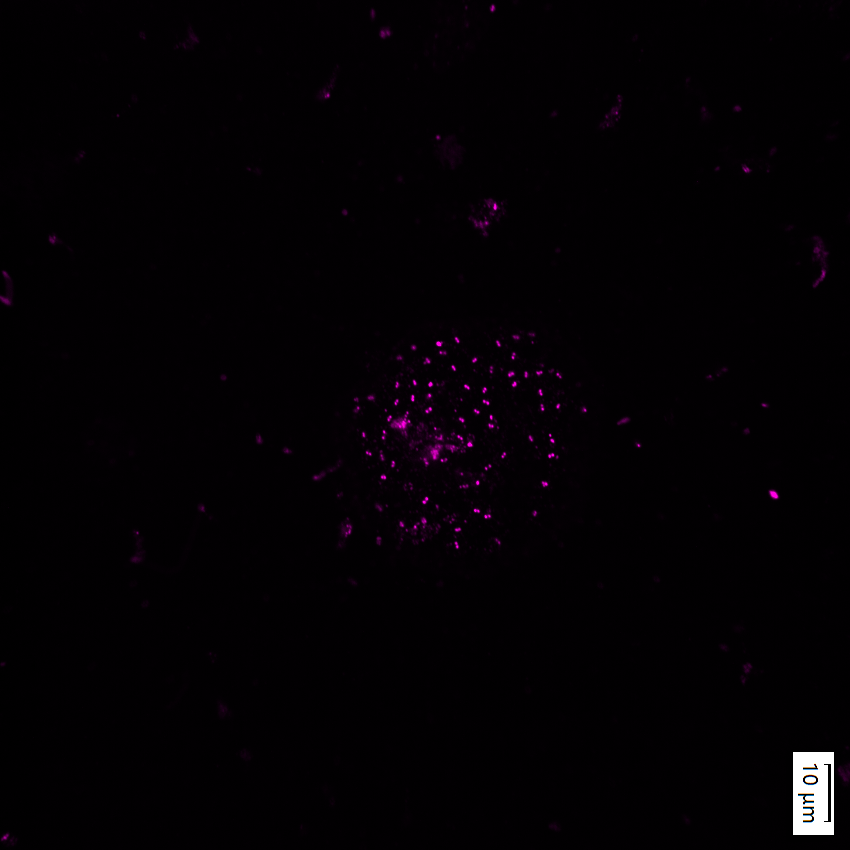

Supplement: Supplementary file 11 — Source data Fig. 5 [file 44318_2025_465_MOESM11_ESM.zip › EMBOJ-2025-120195-Figure 5-Source data/Figure 5/5B/ATRX (Y1419A)-GFP siControl ACA.tif]

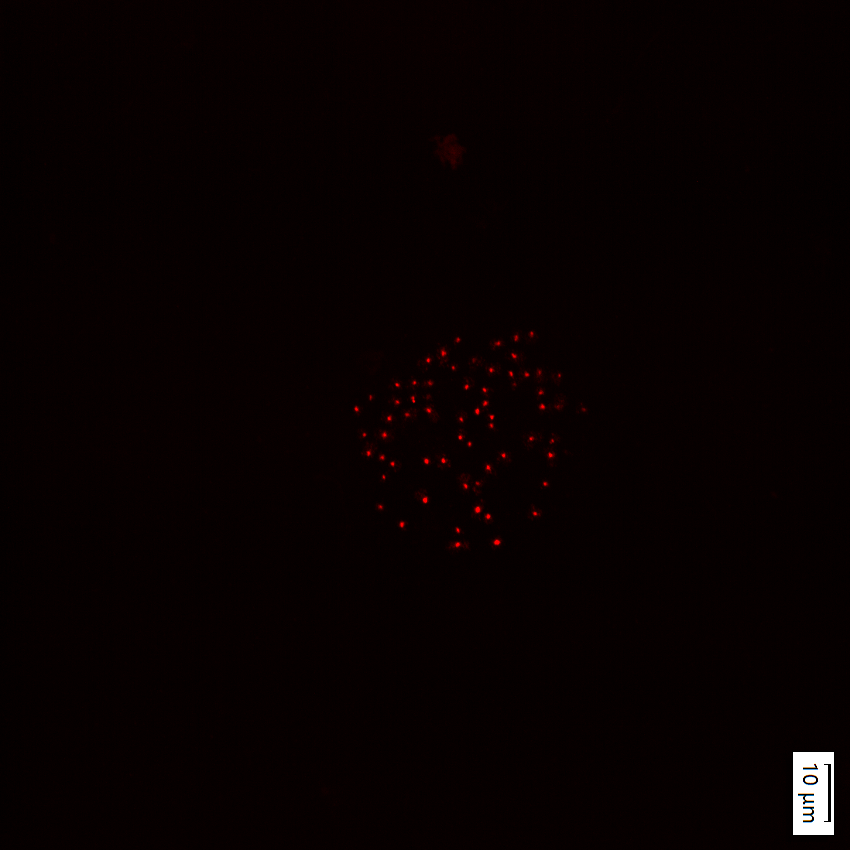

Supplement: Supplementary file 11 — Source data Fig. 5 [file 44318_2025_465_MOESM11_ESM.zip › EMBOJ-2025-120195-Figure 5-Source data/Figure 5/5B/ATRX (Y1419A)-GFP siControl Anti-GFP.tif]

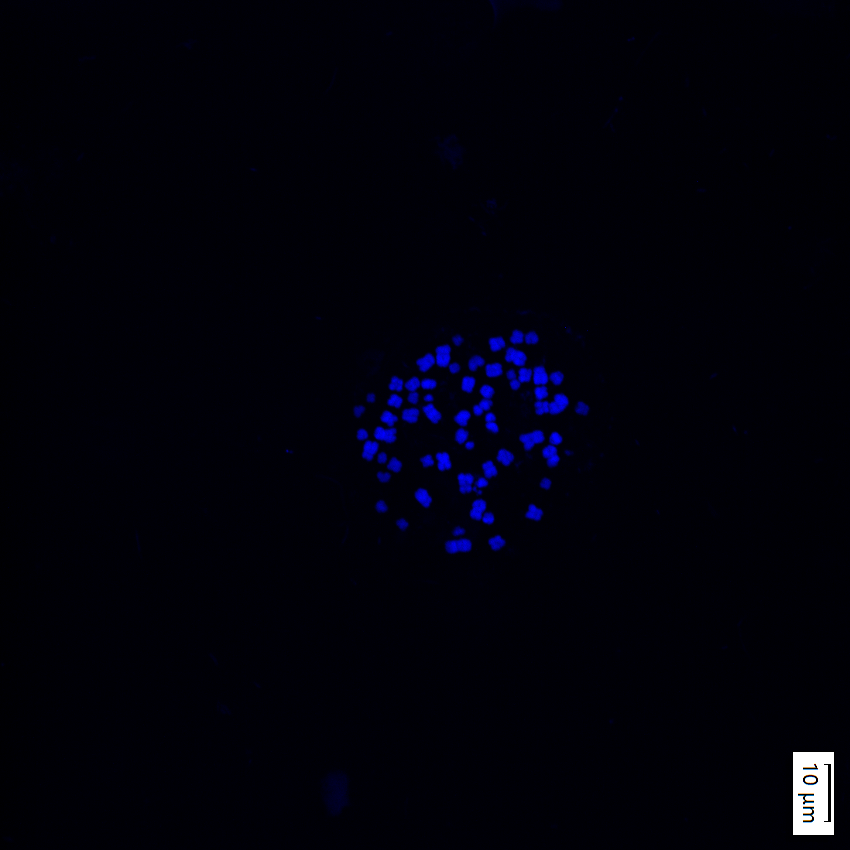

Supplement: Supplementary file 11 — Source data Fig. 5 [file 44318_2025_465_MOESM11_ESM.zip › EMBOJ-2025-120195-Figure 5-Source data/Figure 5/5B/ATRX (Y1419A)-GFP siControl DNA.tif]

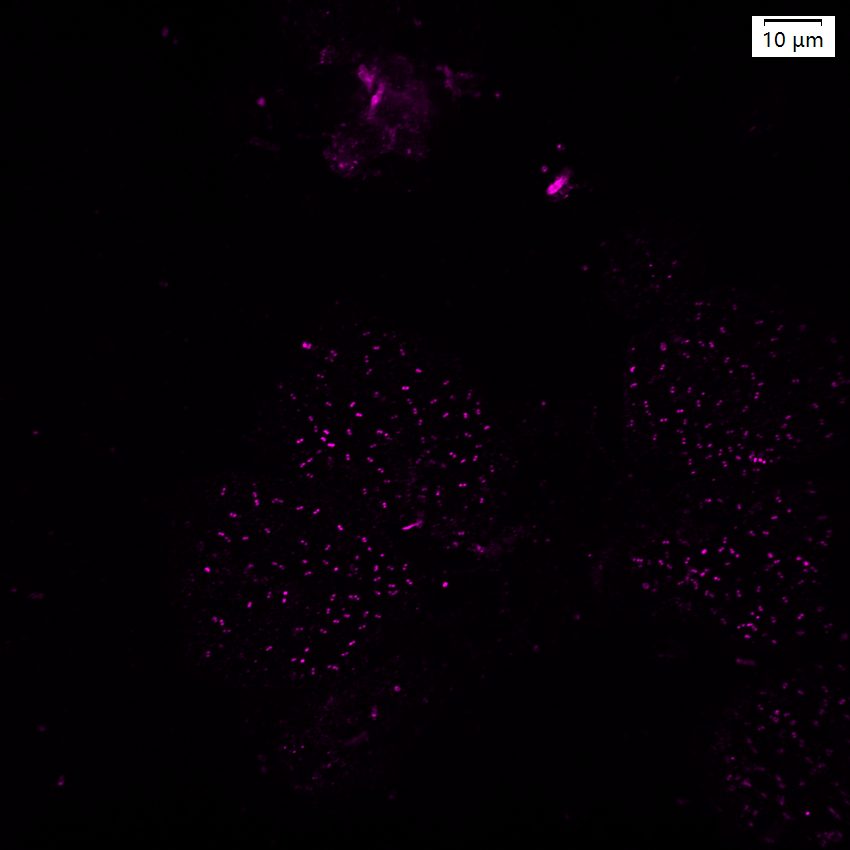

Supplement: Supplementary file 11 — Source data Fig. 5 [file 44318_2025_465_MOESM11_ESM.zip › EMBOJ-2025-120195-Figure 5-Source data/Figure 5/5B/ATRX-GFP siATRX #1 ACA.tif]

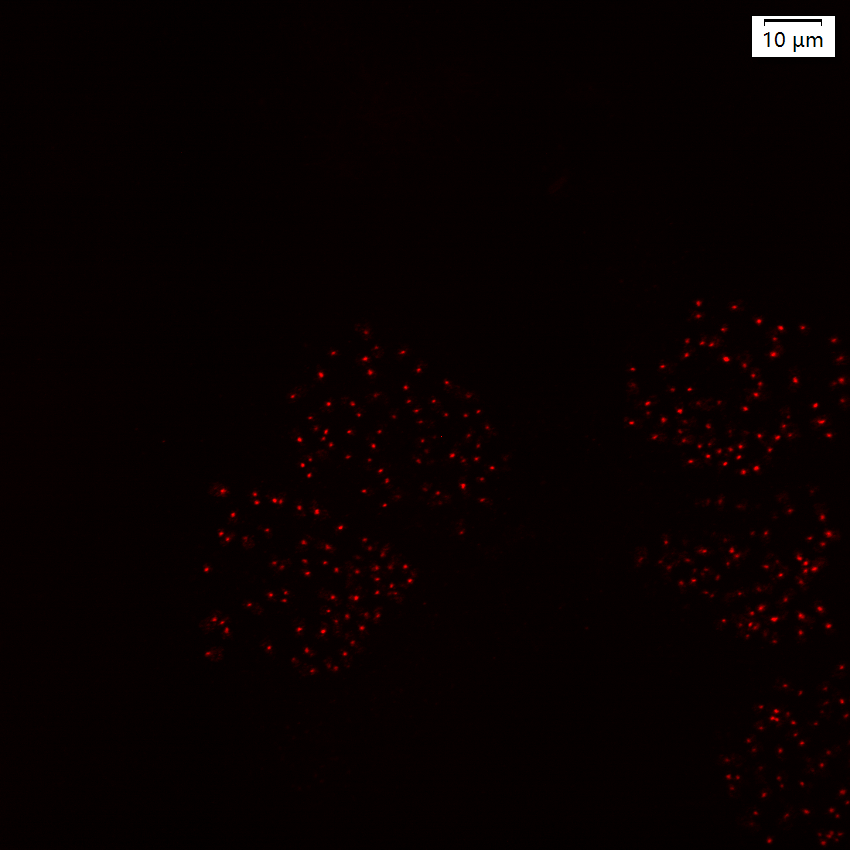

Supplement: Supplementary file 11 — Source data Fig. 5 [file 44318_2025_465_MOESM11_ESM.zip › EMBOJ-2025-120195-Figure 5-Source data/Figure 5/5B/ATRX-GFP siATRX #1 Anti-GFP.tif]

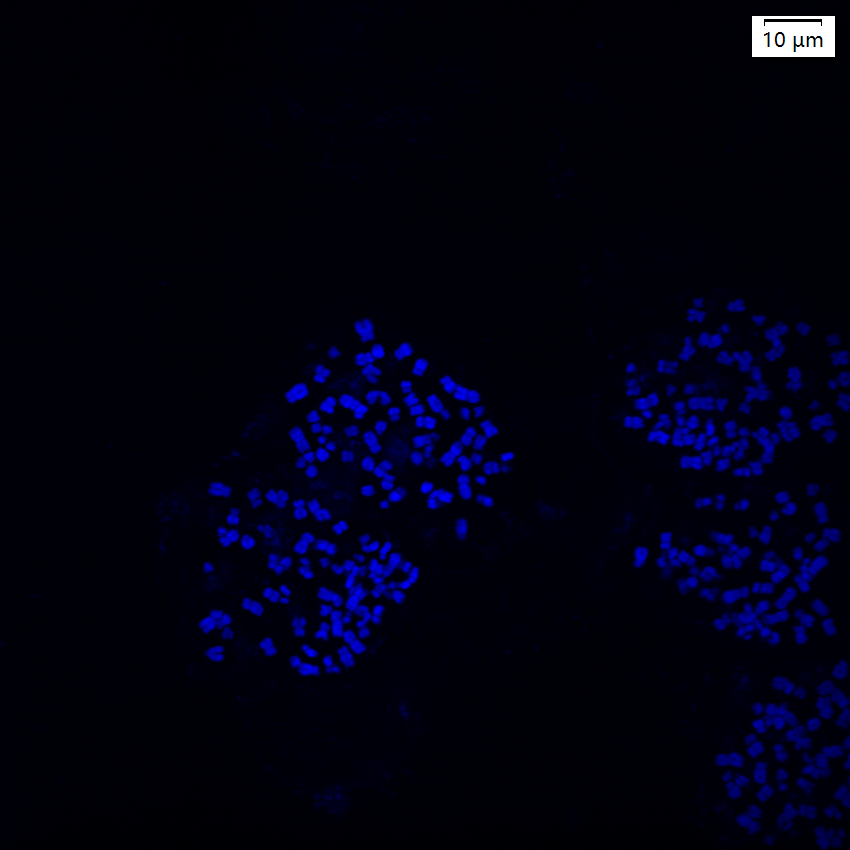

Supplement: Supplementary file 11 — Source data Fig. 5 [file 44318_2025_465_MOESM11_ESM.zip › EMBOJ-2025-120195-Figure 5-Source data/Figure 5/5B/ATRX-GFP siATRX #1 DNA.tif]

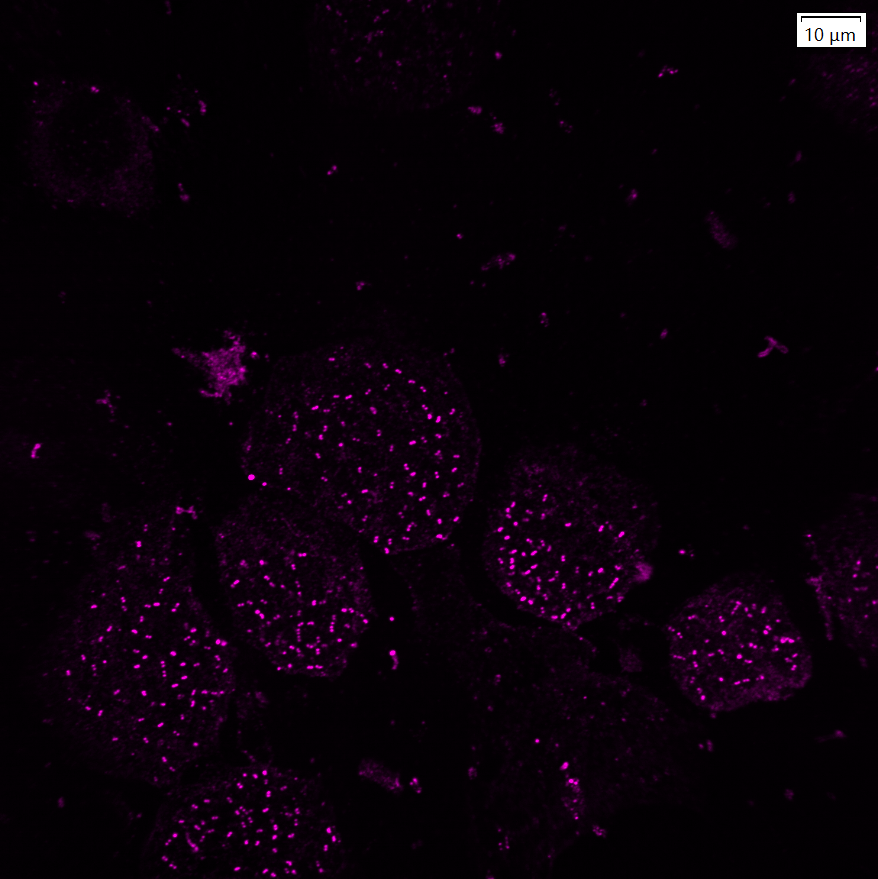

Supplement: Supplementary file 11 — Source data Fig. 5 [file 44318_2025_465_MOESM11_ESM.zip › EMBOJ-2025-120195-Figure 5-Source data/Figure 5/5B/ATRX-GFP siControl ACA.tif]

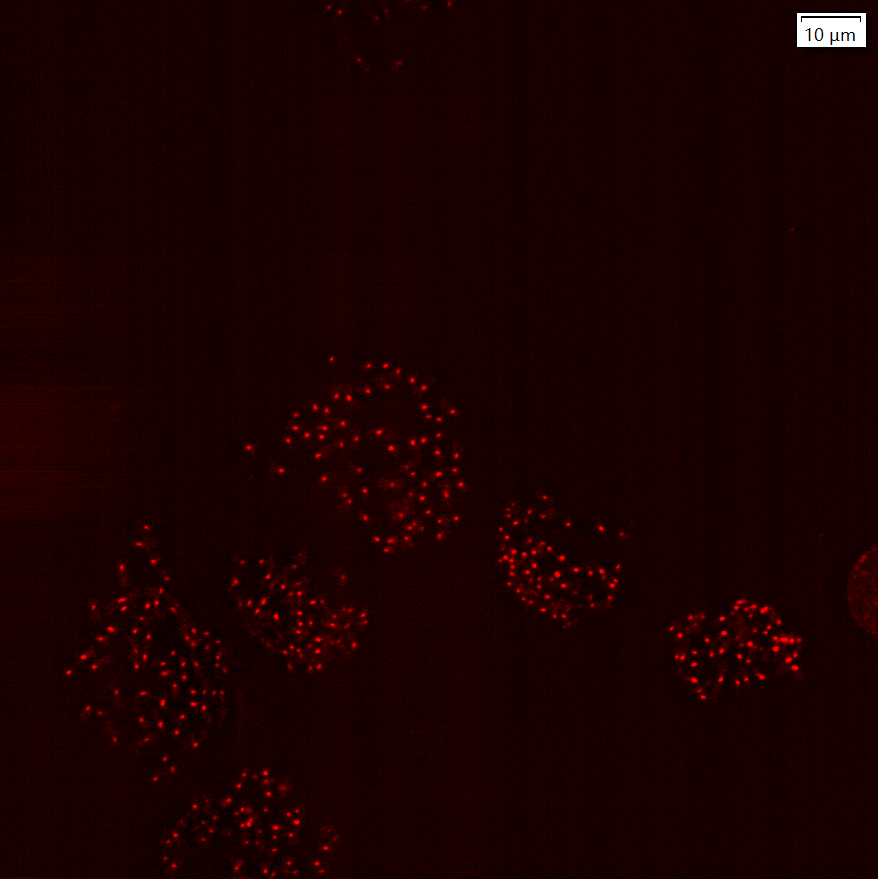

Supplement: Supplementary file 11 — Source data Fig. 5 [file 44318_2025_465_MOESM11_ESM.zip › EMBOJ-2025-120195-Figure 5-Source data/Figure 5/5B/ATRX-GFP siControl Anti-GFP.tif]

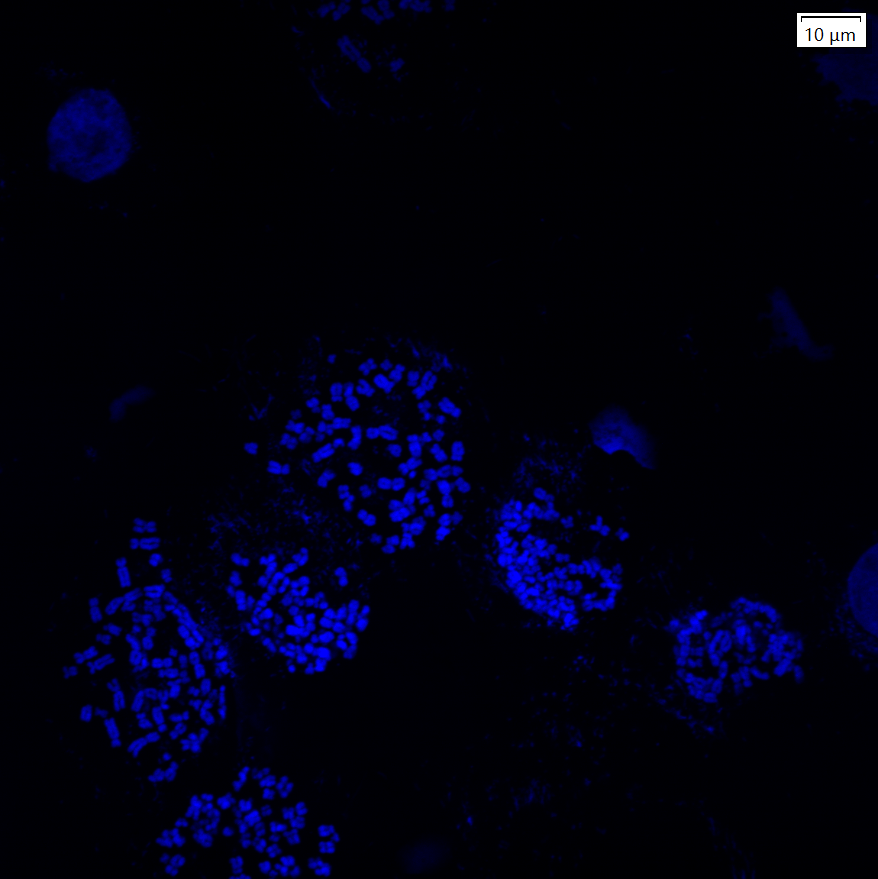

Supplement: Supplementary file 11 — Source data Fig. 5 [file 44318_2025_465_MOESM11_ESM.zip › EMBOJ-2025-120195-Figure 5-Source data/Figure 5/5B/ATRX-GFP siControl DNA.tif]

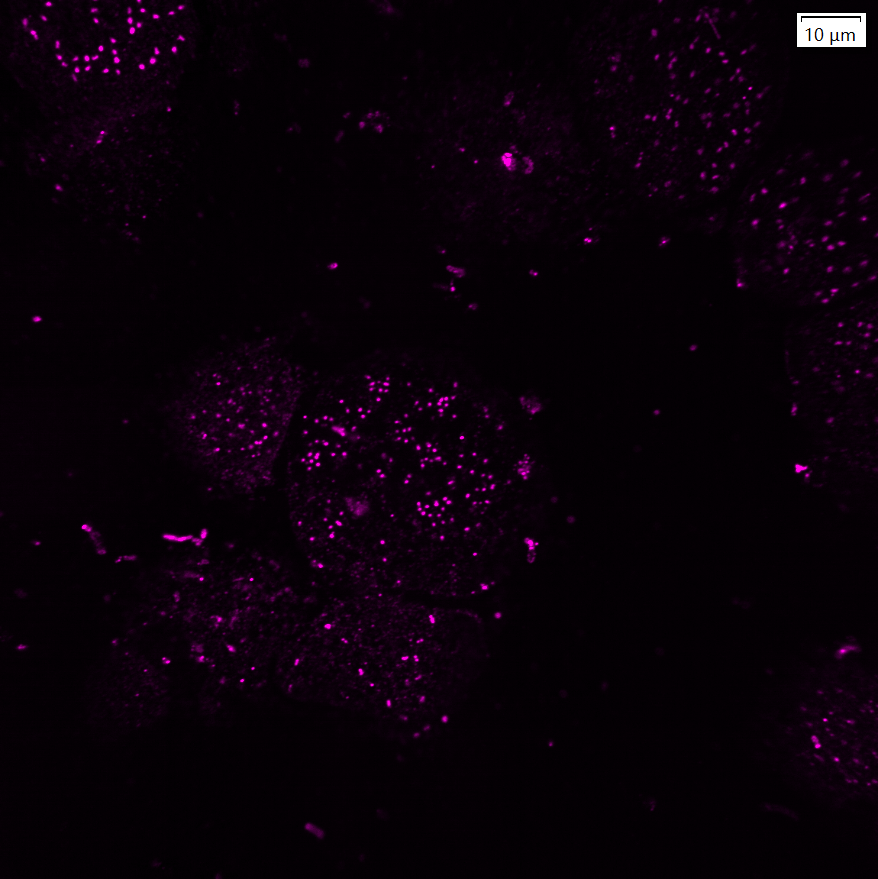

Supplement: Supplementary file 11 — Source data Fig. 5 [file 44318_2025_465_MOESM11_ESM.zip › EMBOJ-2025-120195-Figure 5-Source data/Figure 5/5B/HeLa siATRX #1 ACA.tif]

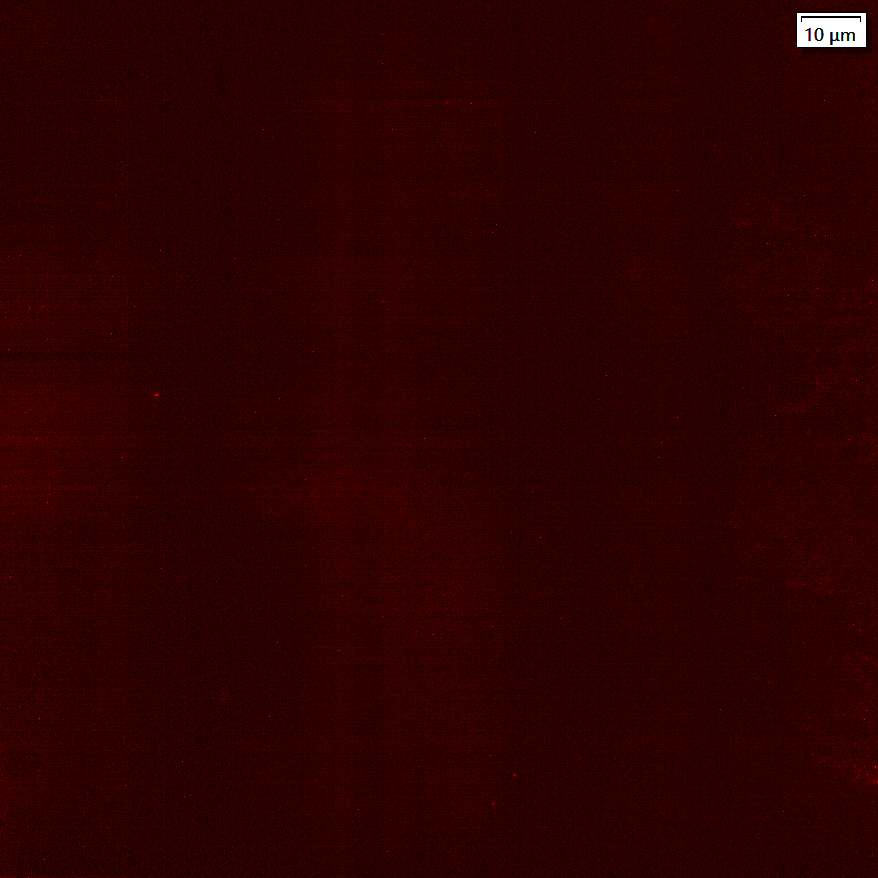

Supplement: Supplementary file 11 — Source data Fig. 5 [file 44318_2025_465_MOESM11_ESM.zip › EMBOJ-2025-120195-Figure 5-Source data/Figure 5/5B/HeLa siATRX #1 Anti-GFP.tif]

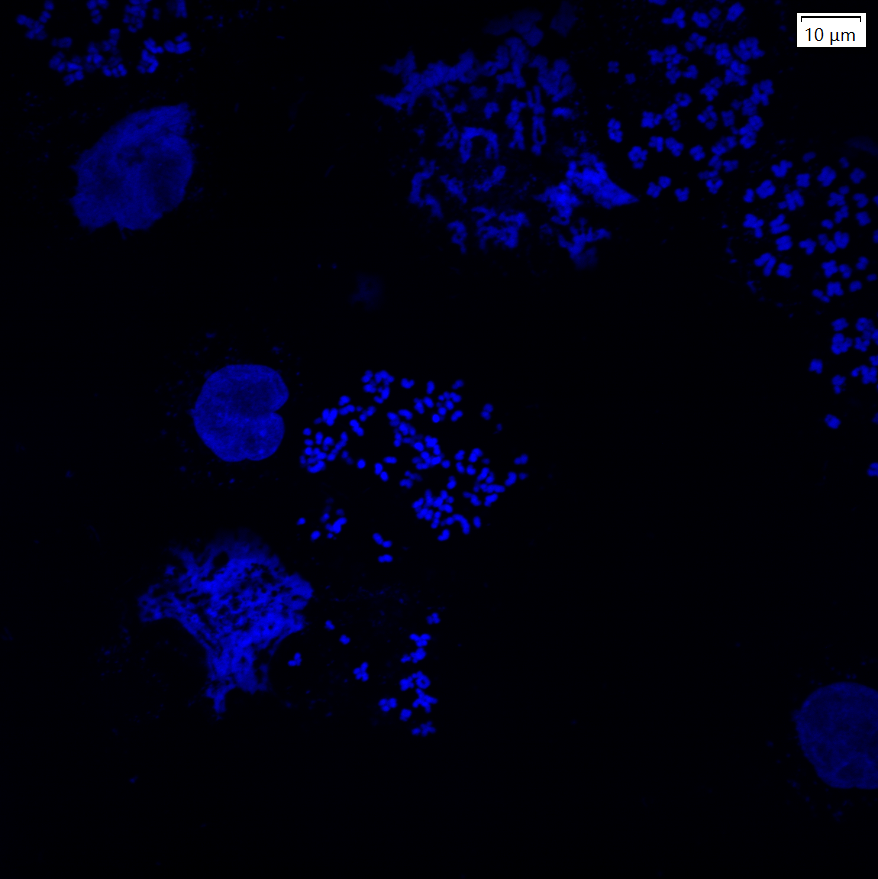

Supplement: Supplementary file 11 — Source data Fig. 5 [file 44318_2025_465_MOESM11_ESM.zip › EMBOJ-2025-120195-Figure 5-Source data/Figure 5/5B/HeLa siATRX #1 DNA.tif]

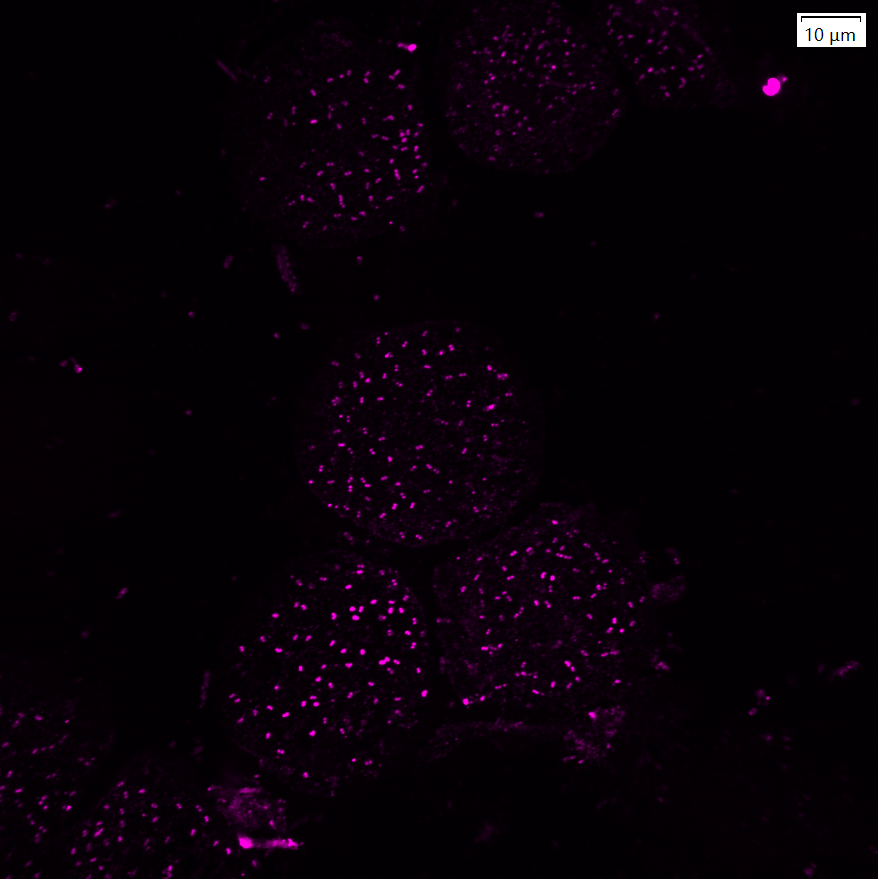

Supplement: Supplementary file 11 — Source data Fig. 5 [file 44318_2025_465_MOESM11_ESM.zip › EMBOJ-2025-120195-Figure 5-Source data/Figure 5/5B/HeLa siControl ACA.tif]
