# Supplementary figures and images for: A chromatin-remodeling-independent role for ATRX in protecting centromeric cohesion (part 2 of 3)
Source: EMBO J. 2025 May 28;44(14):4037–64. doi: 10.1038/s44318-025-00465-6 (PMC12264150; doi:10.1038/s44318-025-00465-6)

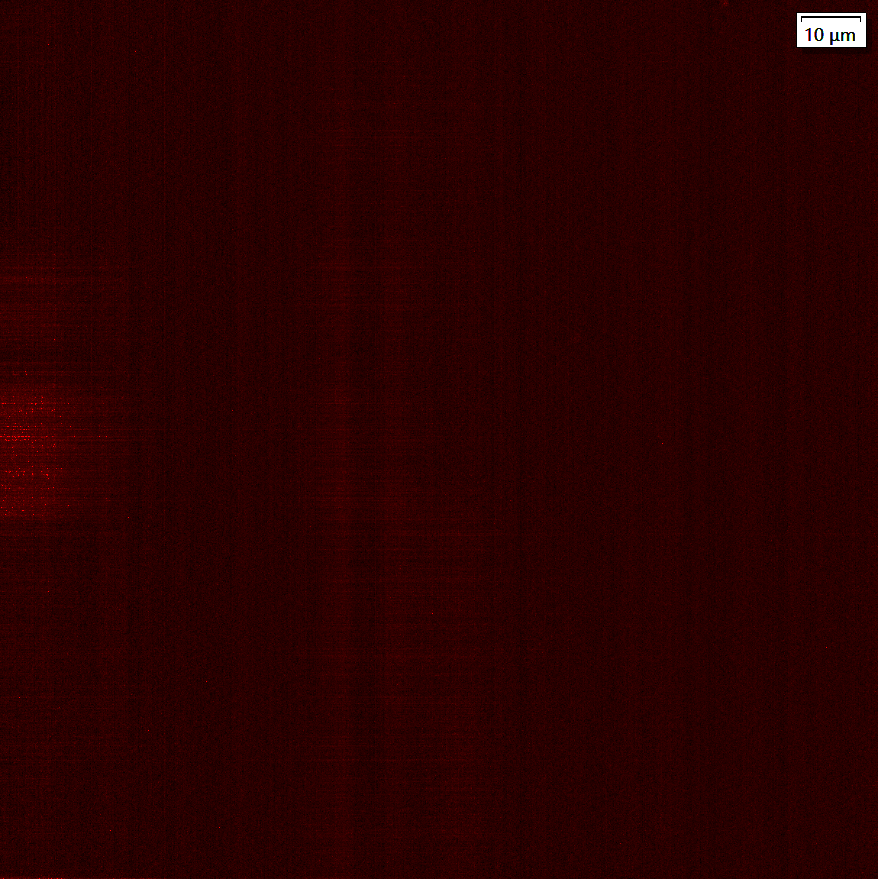

Supplement: Supplementary file 11 — Source data Fig. 5 [file 44318_2025_465_MOESM11_ESM.zip › EMBOJ-2025-120195-Figure 5-Source data/Figure 5/5B/HeLa siControl Anti-GFP.tif]

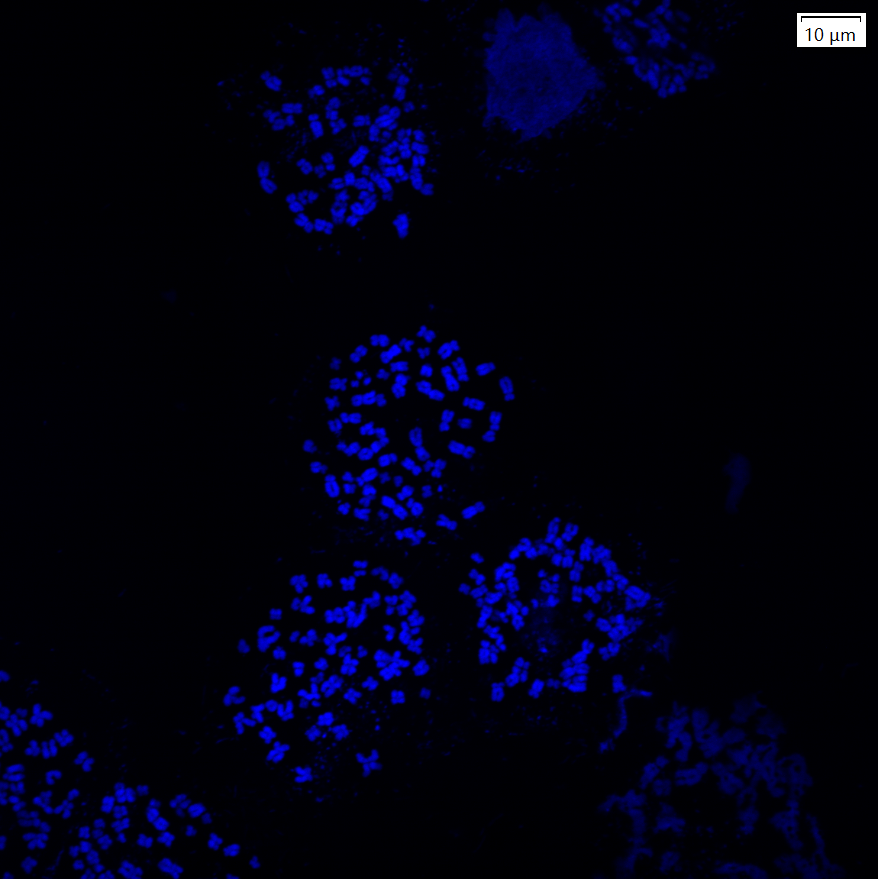

Supplement: Supplementary file 11 — Source data Fig. 5 [file 44318_2025_465_MOESM11_ESM.zip › EMBOJ-2025-120195-Figure 5-Source data/Figure 5/5B/HeLa siControl DNA.tif]

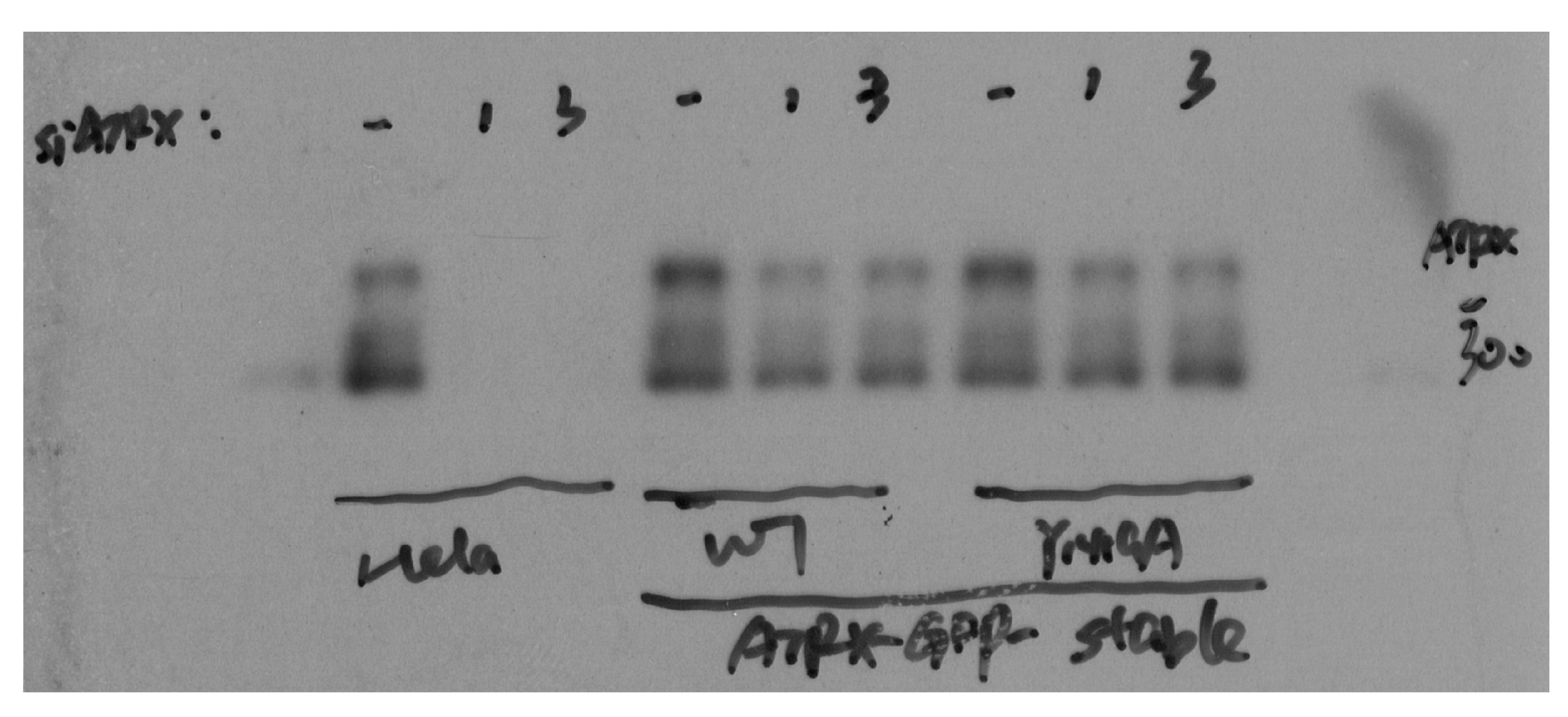

Supplement: Supplementary file 11 — Source data Fig. 5 [file 44318_2025_465_MOESM11_ESM.zip › EMBOJ-2025-120195-Figure 5-Source data/Figure 5/5C/western ATRX.tif]

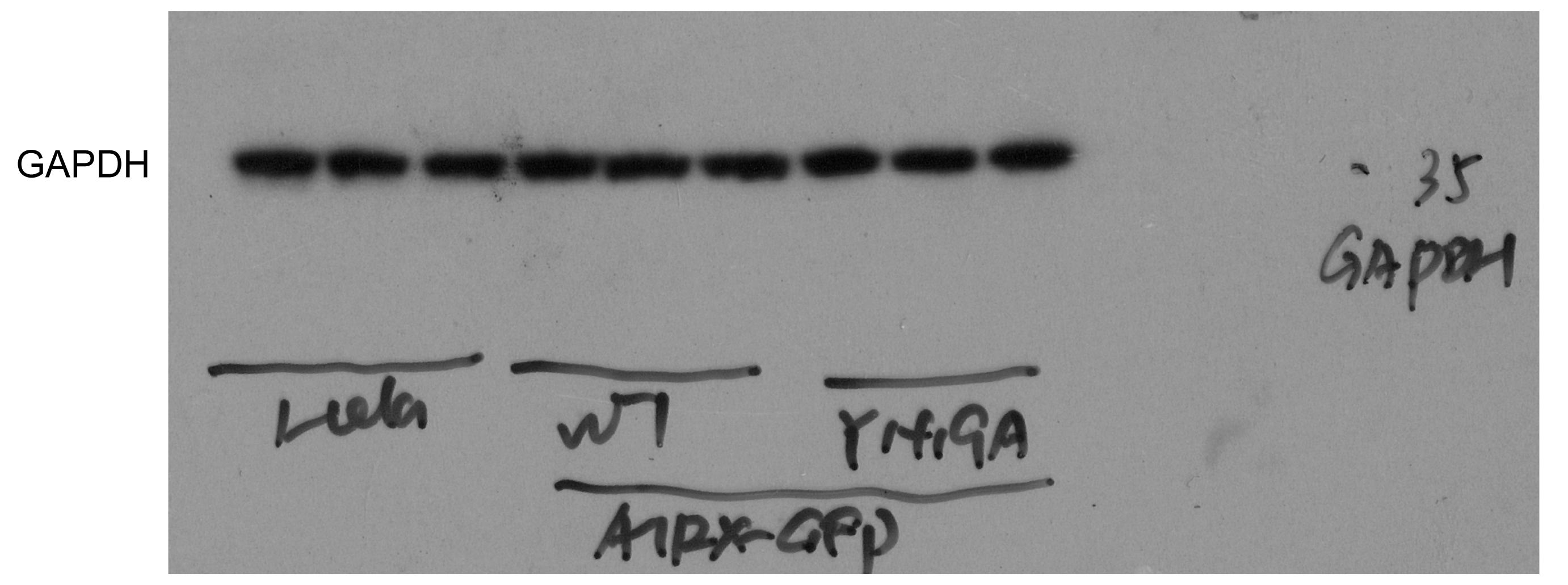

Supplement: Supplementary file 11 — Source data Fig. 5 [file 44318_2025_465_MOESM11_ESM.zip › EMBOJ-2025-120195-Figure 5-Source data/Figure 5/5C/western GAPDH.tif]

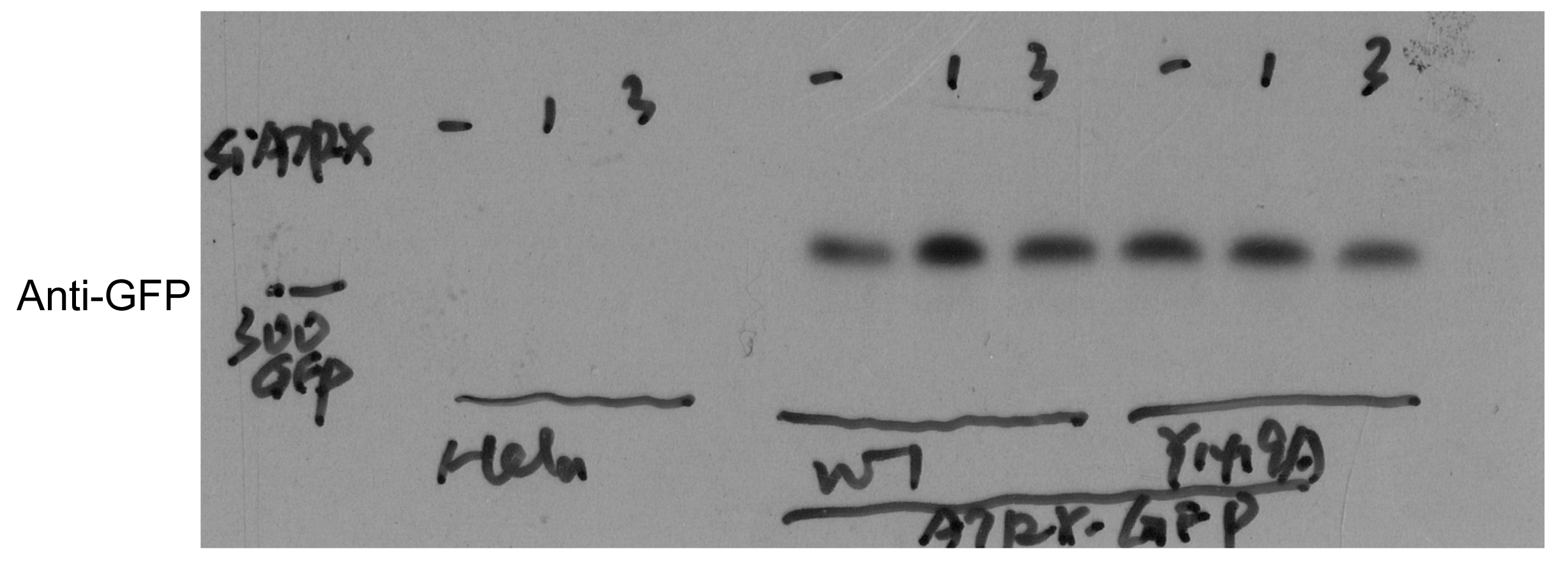

Supplement: Supplementary file 11 — Source data Fig. 5 [file 44318_2025_465_MOESM11_ESM.zip › EMBOJ-2025-120195-Figure 5-Source data/Figure 5/5C/western GFP.tif]

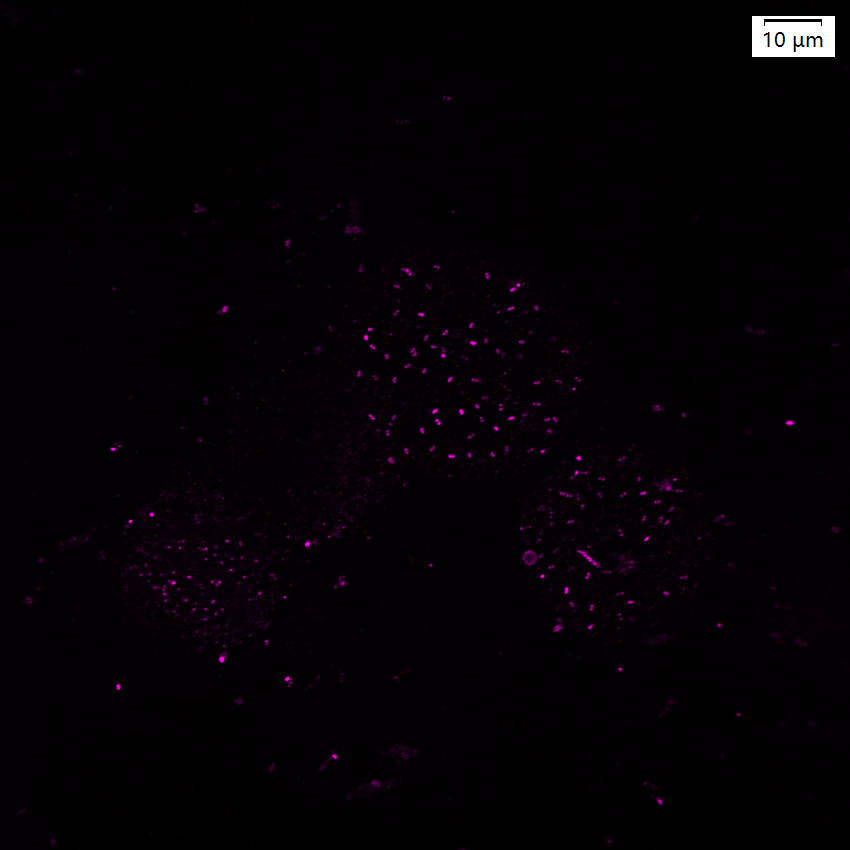

Supplement: Supplementary file 11 — Source data Fig. 5 [file 44318_2025_465_MOESM11_ESM.zip › EMBOJ-2025-120195-Figure 5-Source data/Figure 5/5E/ATRX (Y1419A)-GFP siATRX #1 ACA.tif]

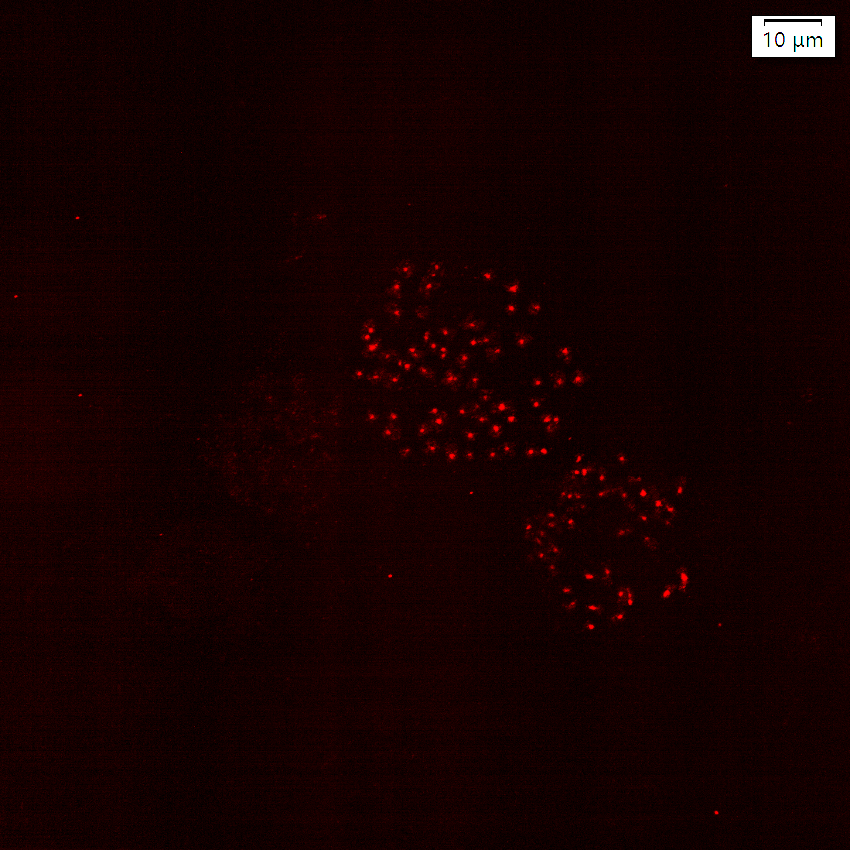

Supplement: Supplementary file 11 — Source data Fig. 5 [file 44318_2025_465_MOESM11_ESM.zip › EMBOJ-2025-120195-Figure 5-Source data/Figure 5/5E/ATRX (Y1419A)-GFP siATRX #1 Anti-GFP.tif]

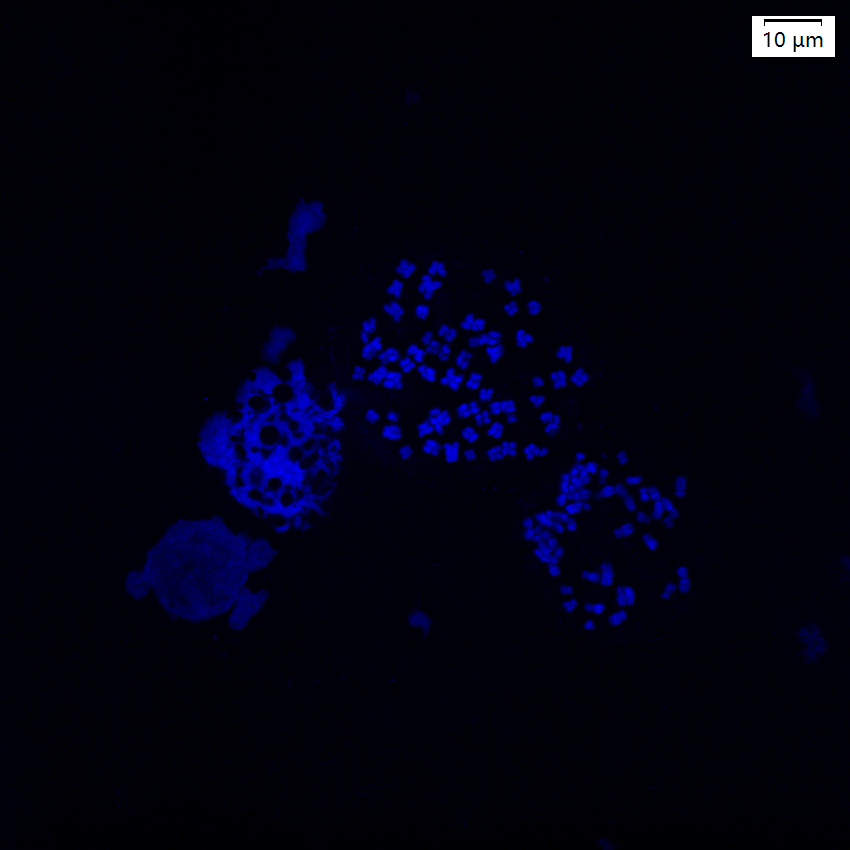

Supplement: Supplementary file 11 — Source data Fig. 5 [file 44318_2025_465_MOESM11_ESM.zip › EMBOJ-2025-120195-Figure 5-Source data/Figure 5/5E/ATRX (Y1419A)-GFP siATRX #1 DNA.tif]

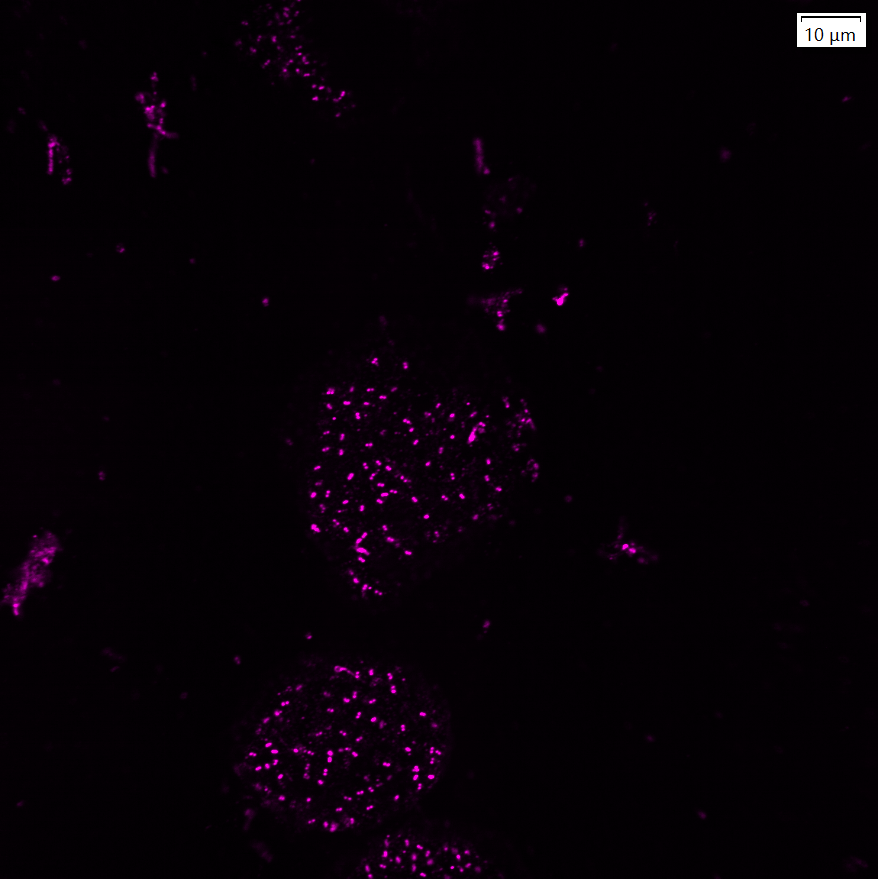

Supplement: Supplementary file 11 — Source data Fig. 5 [file 44318_2025_465_MOESM11_ESM.zip › EMBOJ-2025-120195-Figure 5-Source data/Figure 5/5E/ATRX (Y1419A)-GFP siControl ACA.tif]

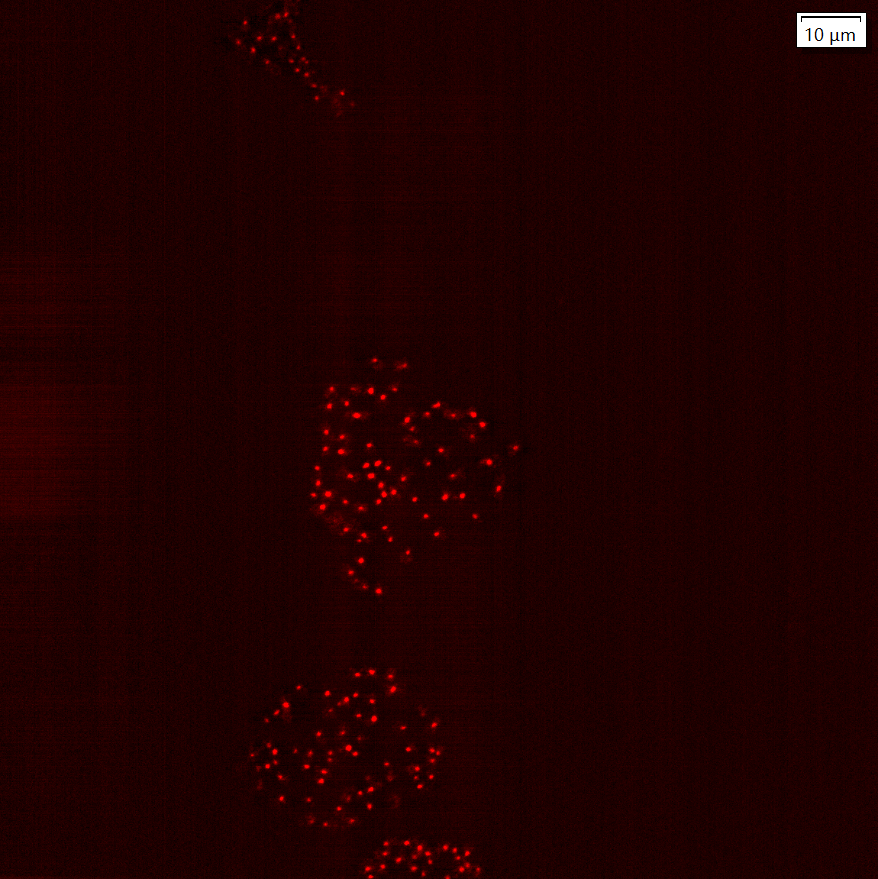

Supplement: Supplementary file 11 — Source data Fig. 5 [file 44318_2025_465_MOESM11_ESM.zip › EMBOJ-2025-120195-Figure 5-Source data/Figure 5/5E/ATRX (Y1419A)-GFP siControl Anti-GFP.tif]

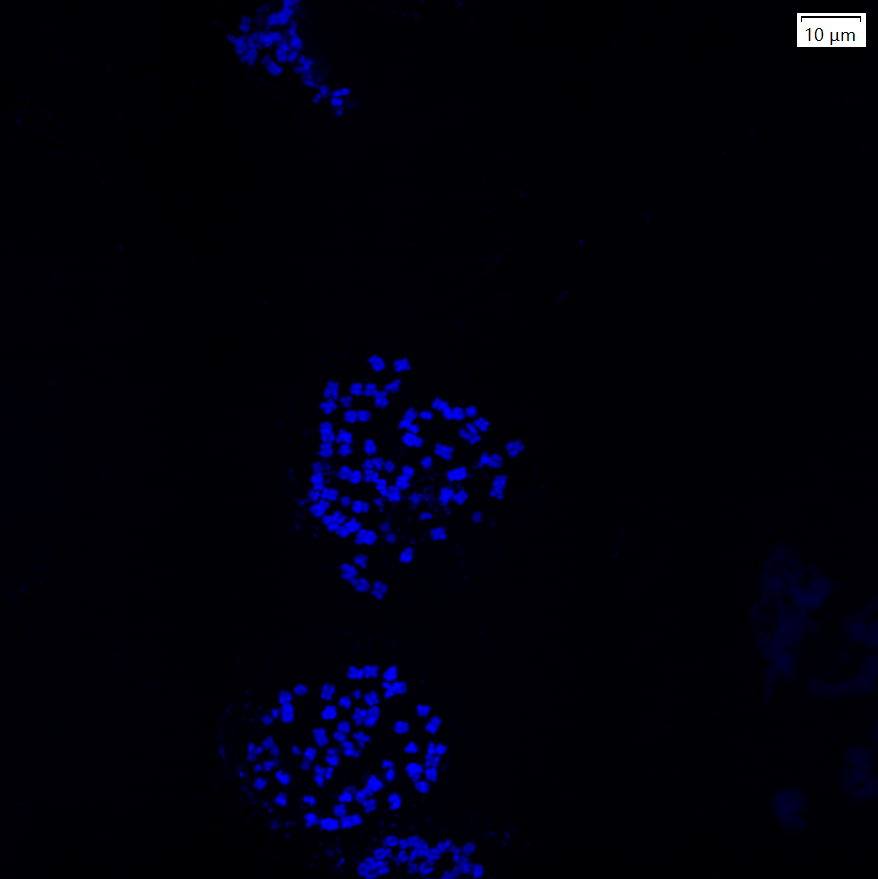

Supplement: Supplementary file 11 — Source data Fig. 5 [file 44318_2025_465_MOESM11_ESM.zip › EMBOJ-2025-120195-Figure 5-Source data/Figure 5/5E/ATRX (Y1419A)-GFP siControl DNA.tif]

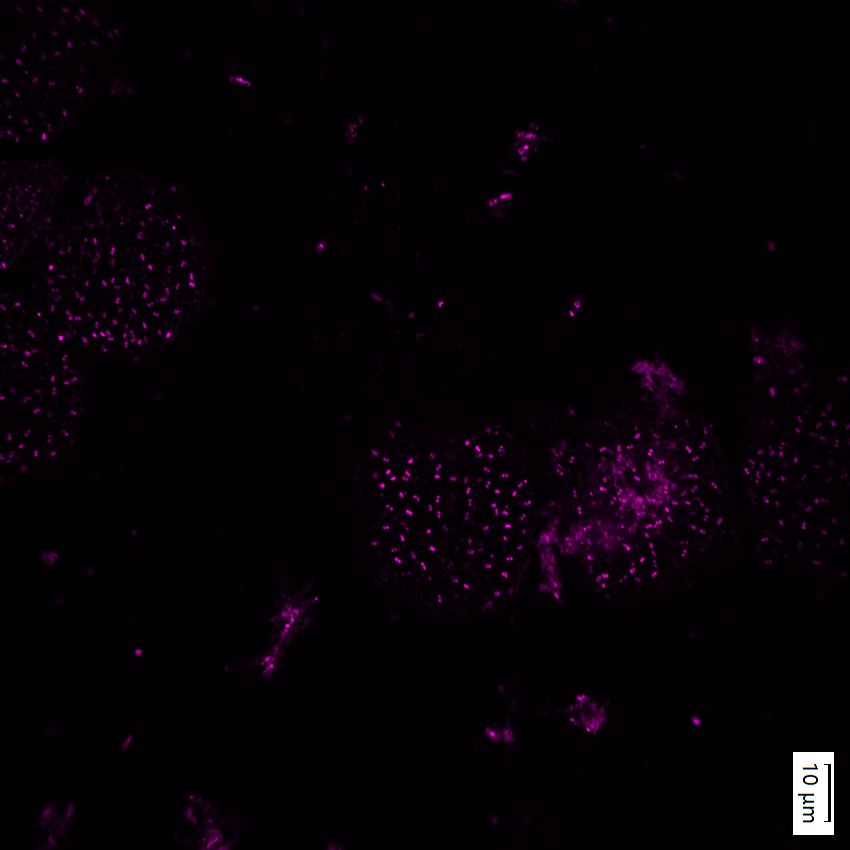

Supplement: Supplementary file 11 — Source data Fig. 5 [file 44318_2025_465_MOESM11_ESM.zip › EMBOJ-2025-120195-Figure 5-Source data/Figure 5/5E/ATRX-GFP siATRX #1 ACA.tif]

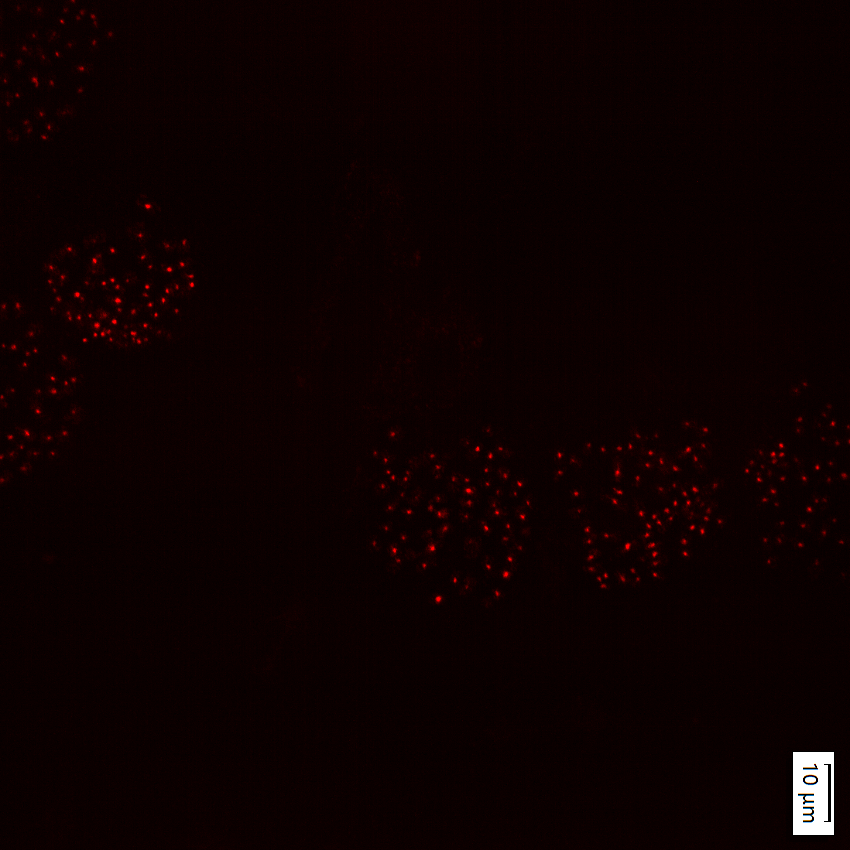

Supplement: Supplementary file 11 — Source data Fig. 5 [file 44318_2025_465_MOESM11_ESM.zip › EMBOJ-2025-120195-Figure 5-Source data/Figure 5/5E/ATRX-GFP siATRX #1 Anti-GFP.tif]

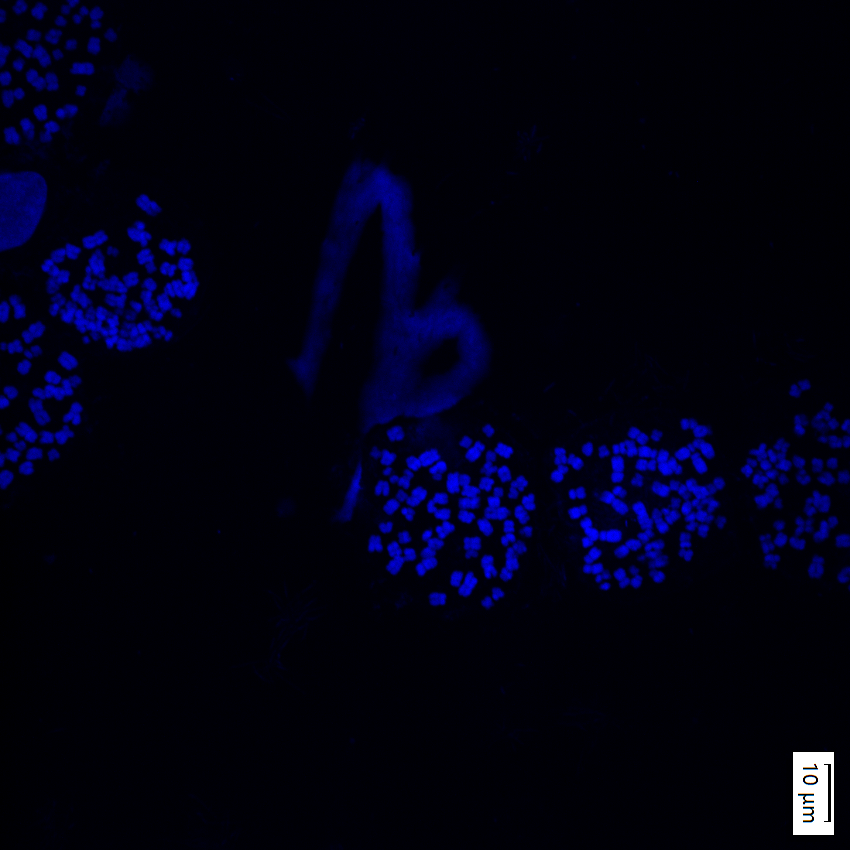

Supplement: Supplementary file 11 — Source data Fig. 5 [file 44318_2025_465_MOESM11_ESM.zip › EMBOJ-2025-120195-Figure 5-Source data/Figure 5/5E/ATRX-GFP siATRX #1 DNA.tif]

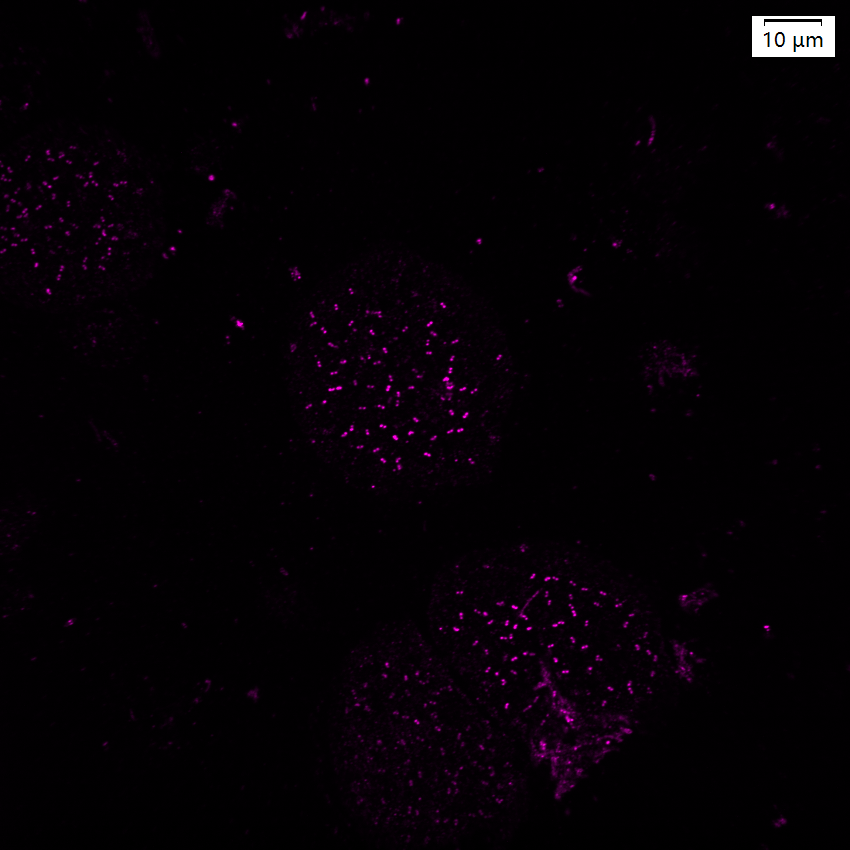

Supplement: Supplementary file 11 — Source data Fig. 5 [file 44318_2025_465_MOESM11_ESM.zip › EMBOJ-2025-120195-Figure 5-Source data/Figure 5/5E/ATRX-GFP siControl ACA.tif]

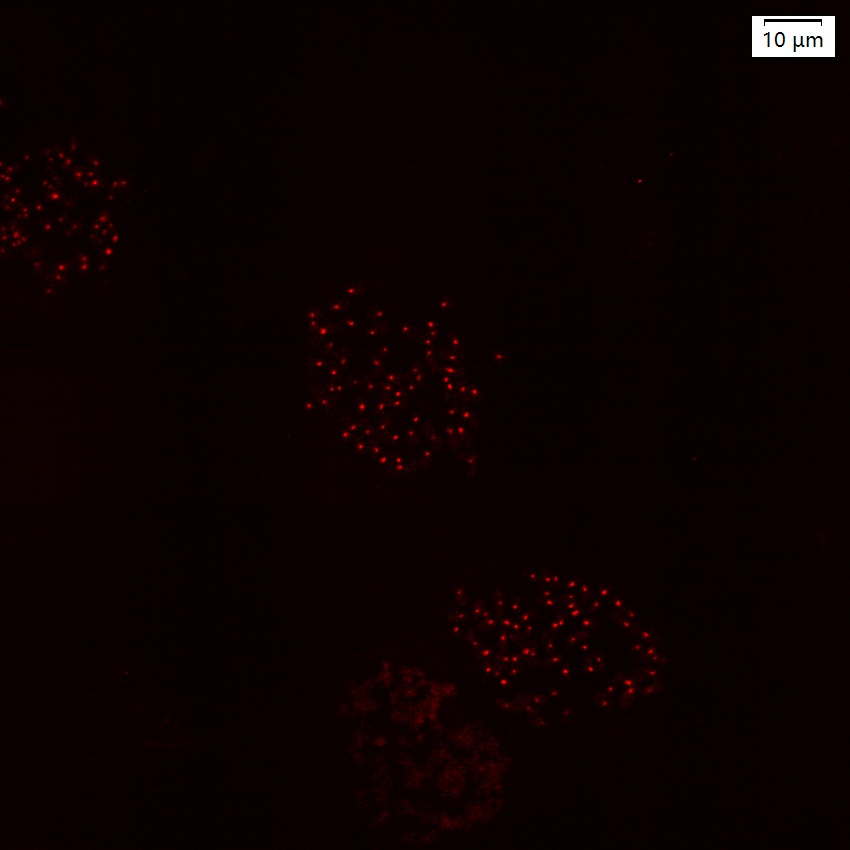

Supplement: Supplementary file 11 — Source data Fig. 5 [file 44318_2025_465_MOESM11_ESM.zip › EMBOJ-2025-120195-Figure 5-Source data/Figure 5/5E/ATRX-GFP siControl Anti-GFP.tif]

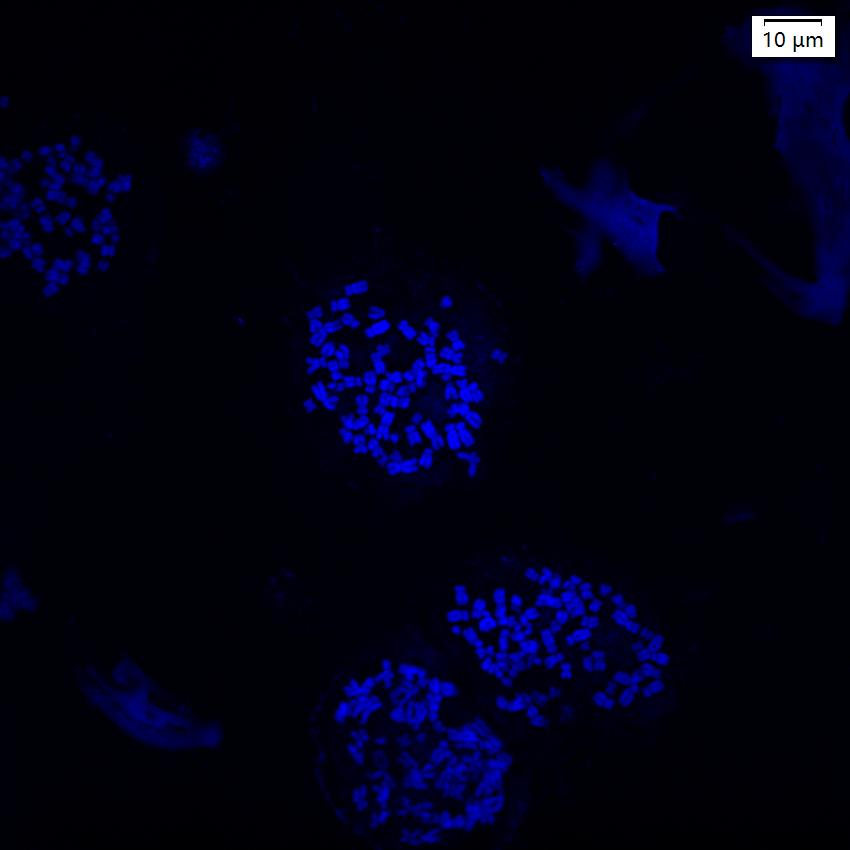

Supplement: Supplementary file 11 — Source data Fig. 5 [file 44318_2025_465_MOESM11_ESM.zip › EMBOJ-2025-120195-Figure 5-Source data/Figure 5/5E/ATRX-GFP siControl DNA.tif]

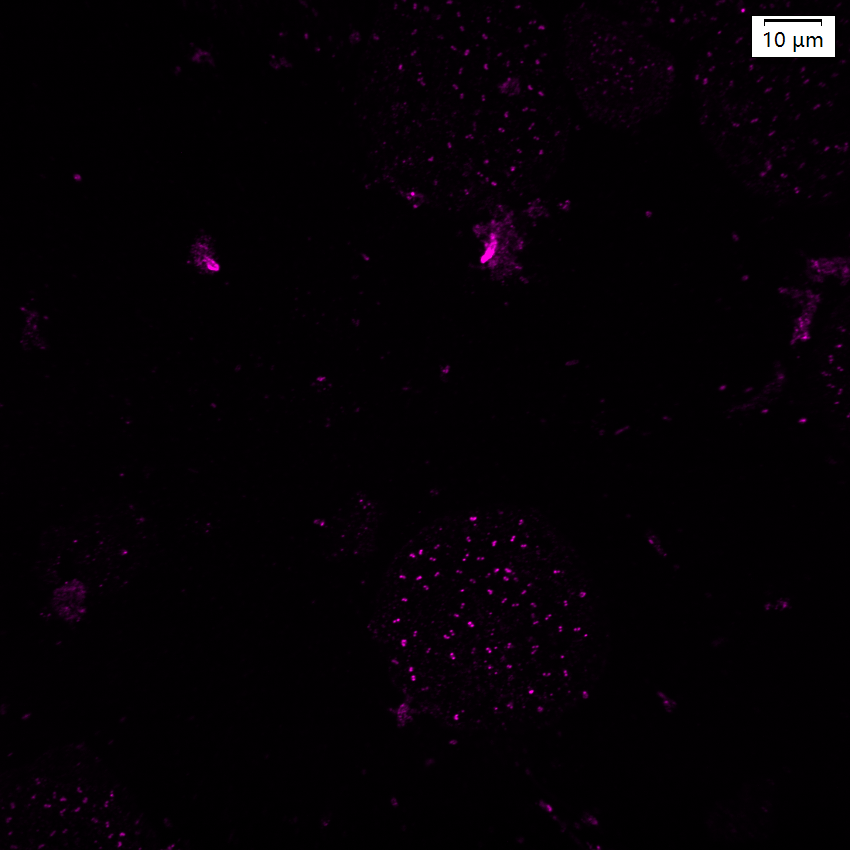

Supplement: Supplementary file 11 — Source data Fig. 5 [file 44318_2025_465_MOESM11_ESM.zip › EMBOJ-2025-120195-Figure 5-Source data/Figure 5/5E/HeLa siATRX #1 ACA.tif]

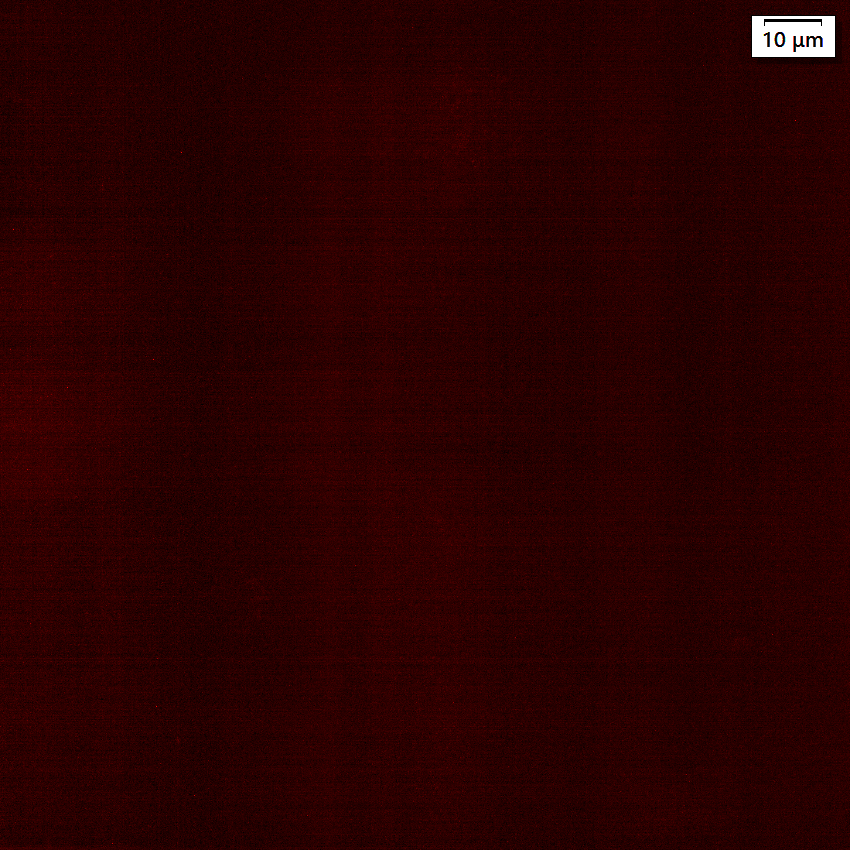

Supplement: Supplementary file 11 — Source data Fig. 5 [file 44318_2025_465_MOESM11_ESM.zip › EMBOJ-2025-120195-Figure 5-Source data/Figure 5/5E/HeLa siATRX #1 Anti-GFP.tif]

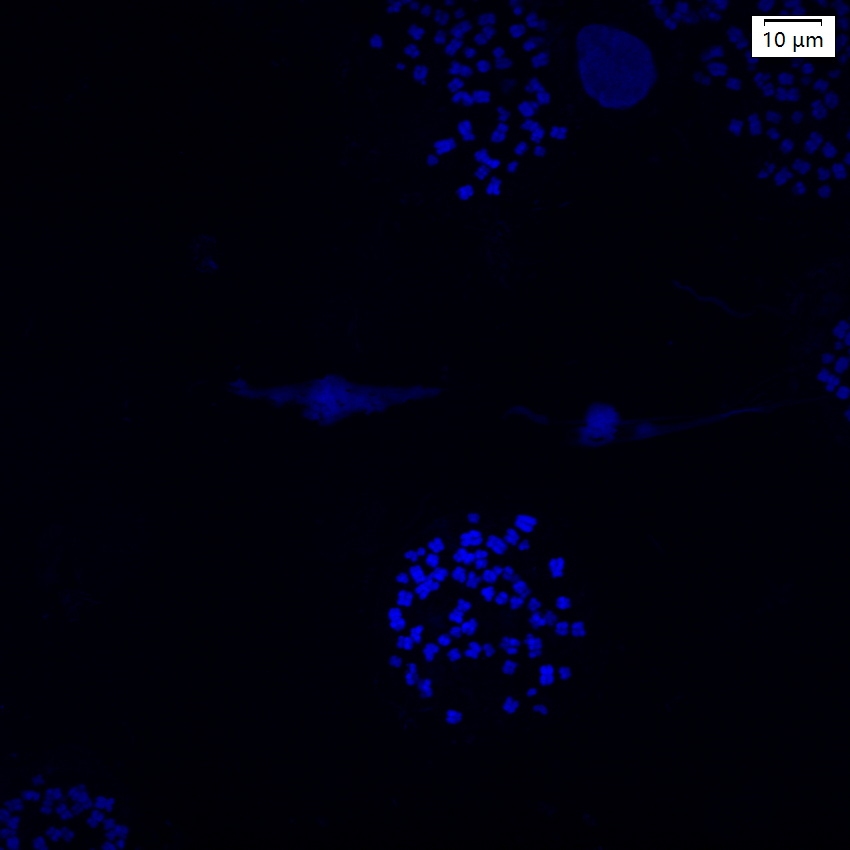

Supplement: Supplementary file 11 — Source data Fig. 5 [file 44318_2025_465_MOESM11_ESM.zip › EMBOJ-2025-120195-Figure 5-Source data/Figure 5/5E/HeLa siATRX #1 DNA.tif]

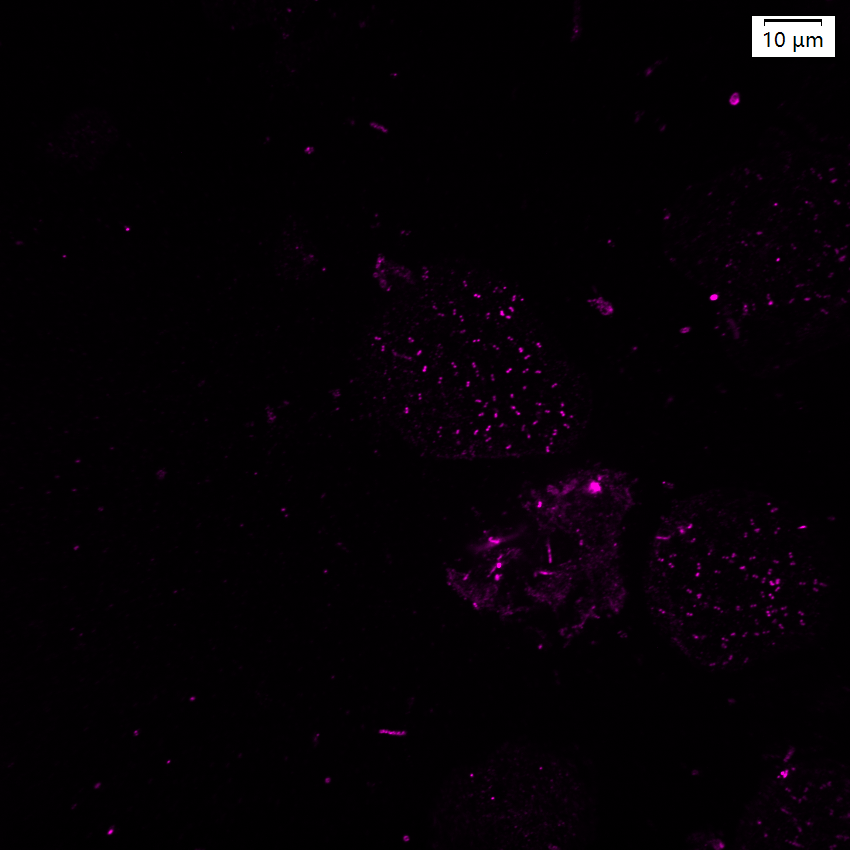

Supplement: Supplementary file 11 — Source data Fig. 5 [file 44318_2025_465_MOESM11_ESM.zip › EMBOJ-2025-120195-Figure 5-Source data/Figure 5/5E/HeLa siControl ACA.tif]

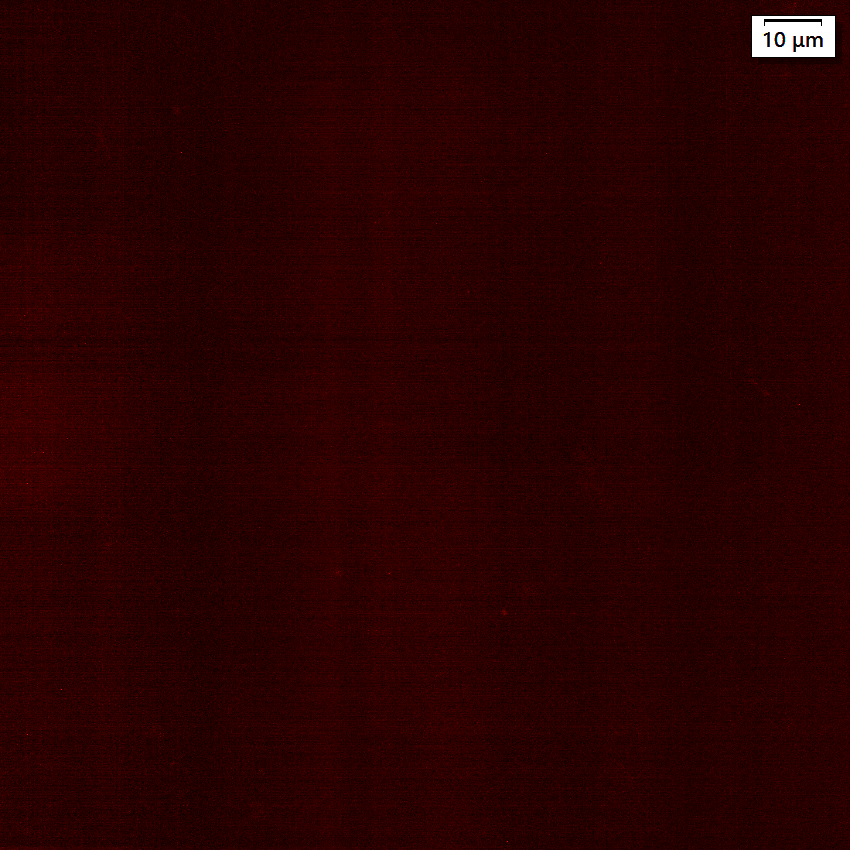

Supplement: Supplementary file 11 — Source data Fig. 5 [file 44318_2025_465_MOESM11_ESM.zip › EMBOJ-2025-120195-Figure 5-Source data/Figure 5/5E/HeLa siControl Anti-GFP.tif]

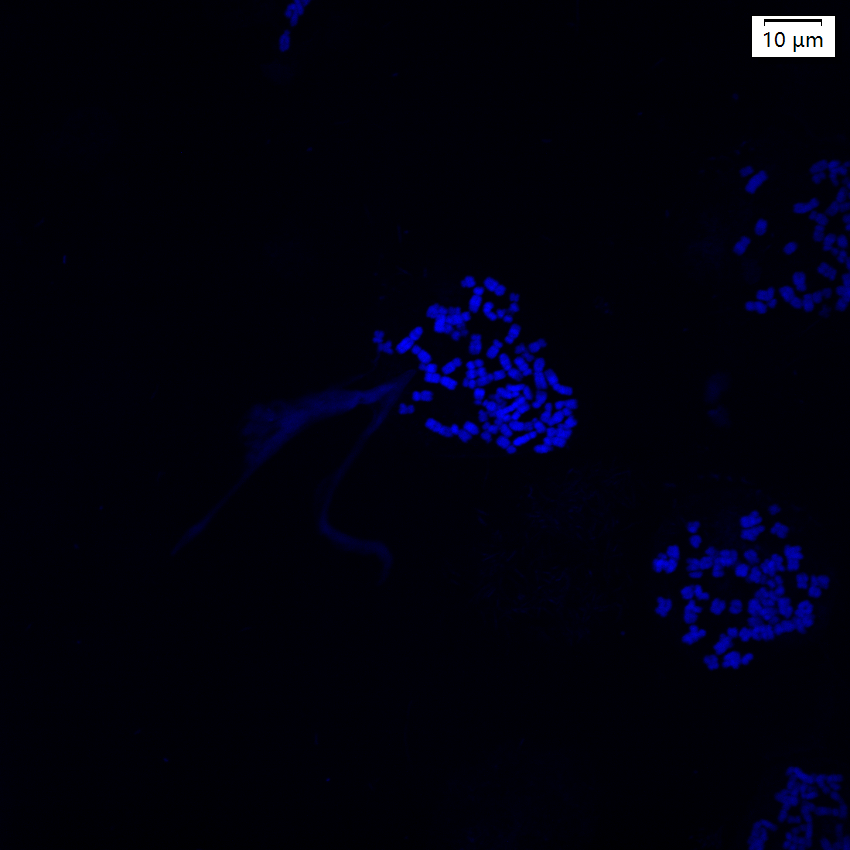

Supplement: Supplementary file 11 — Source data Fig. 5 [file 44318_2025_465_MOESM11_ESM.zip › EMBOJ-2025-120195-Figure 5-Source data/Figure 5/5E/HeLa siControl DNA.tif]

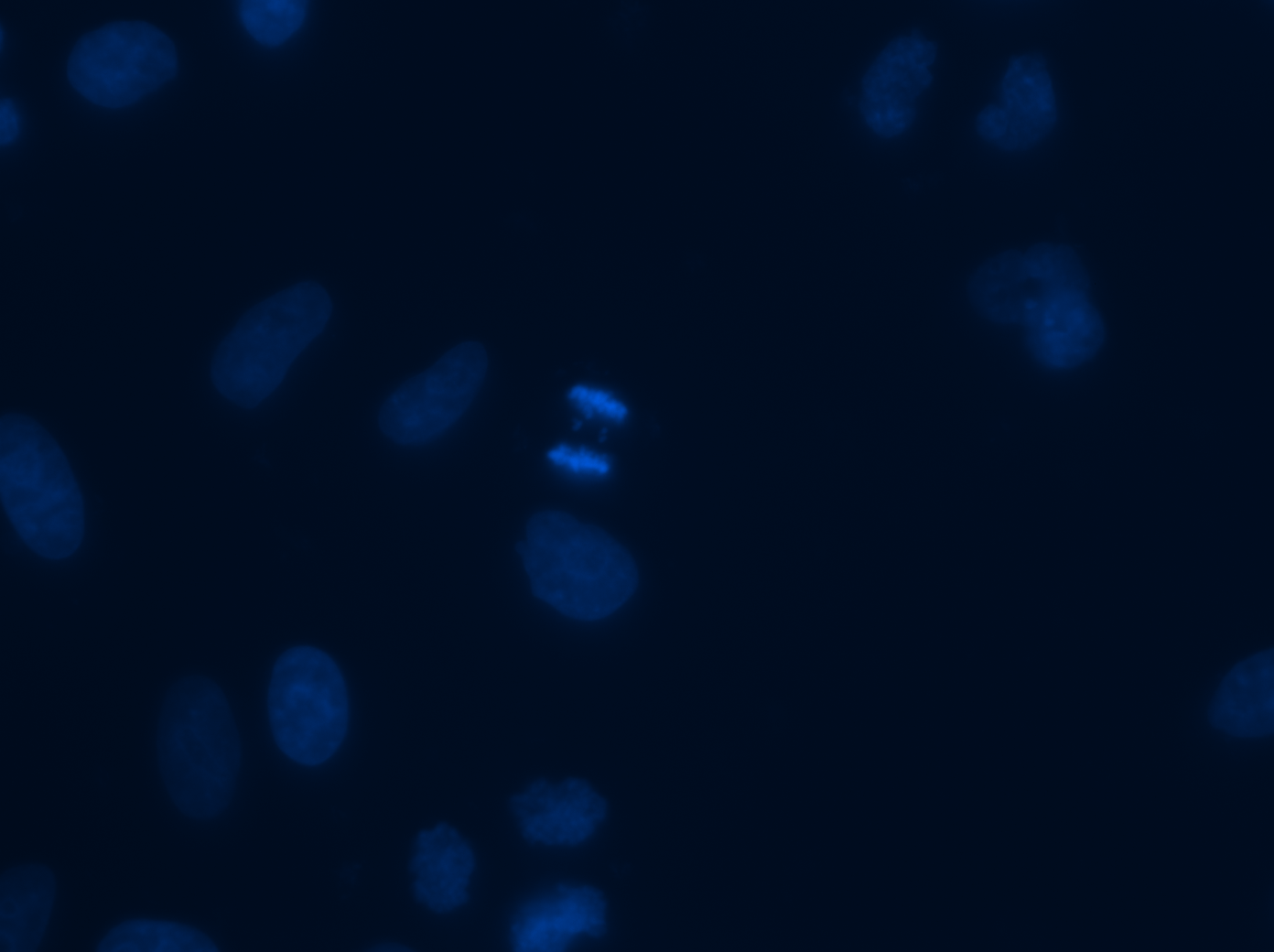

Supplement: Supplementary file 11 — Source data Fig. 5 [file 44318_2025_465_MOESM11_ESM.zip › EMBOJ-2025-120195-Figure 5-Source data/Figure 5/5H/ATRX (Y1419A)-GFP siATRX #1.tif]

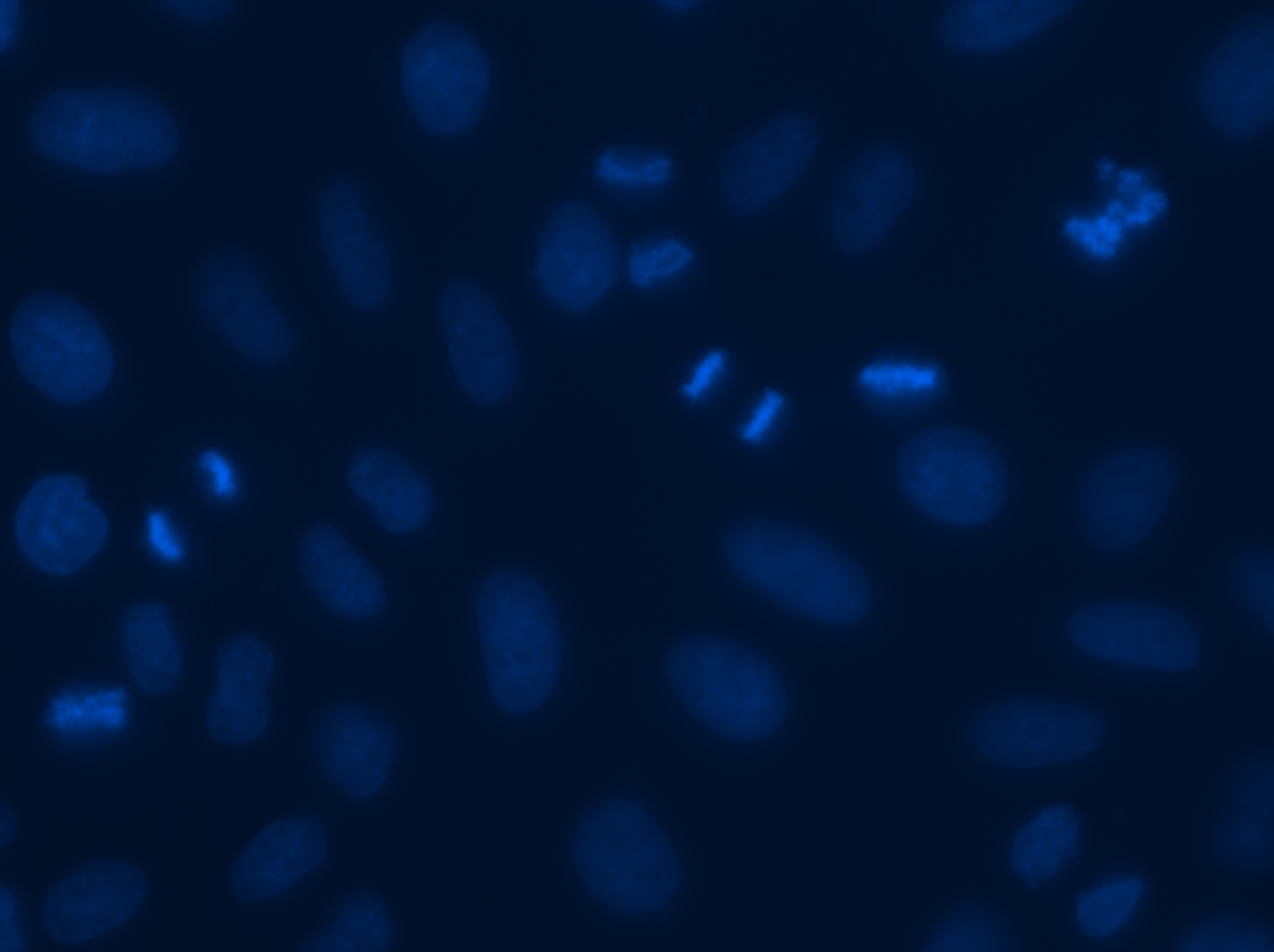

Supplement: Supplementary file 11 — Source data Fig. 5 [file 44318_2025_465_MOESM11_ESM.zip › EMBOJ-2025-120195-Figure 5-Source data/Figure 5/5H/ATRX (Y1419A)-GFP siControl.tif]

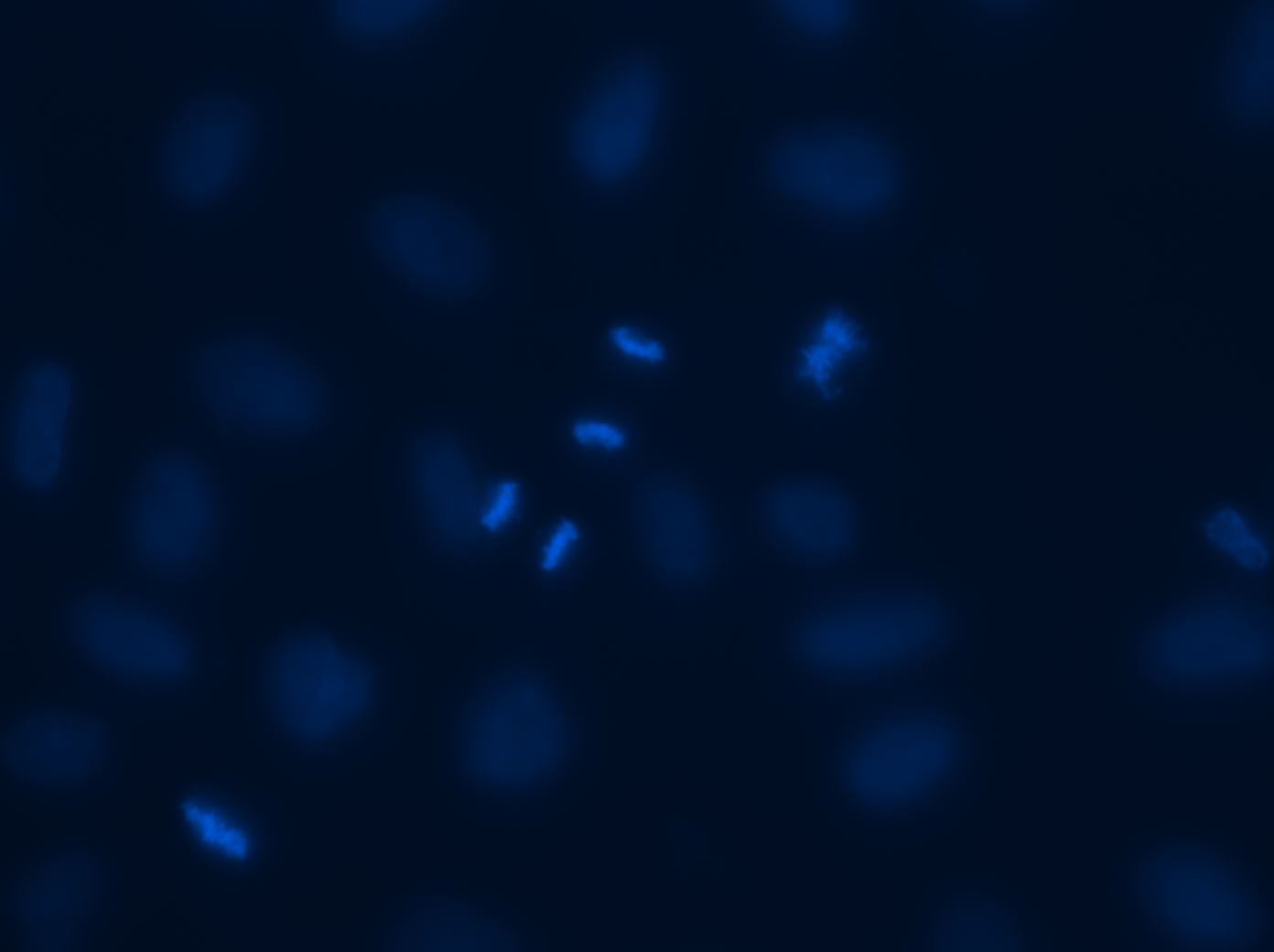

Supplement: Supplementary file 11 — Source data Fig. 5 [file 44318_2025_465_MOESM11_ESM.zip › EMBOJ-2025-120195-Figure 5-Source data/Figure 5/5H/ATRX-GFP siATRX #1.tif]

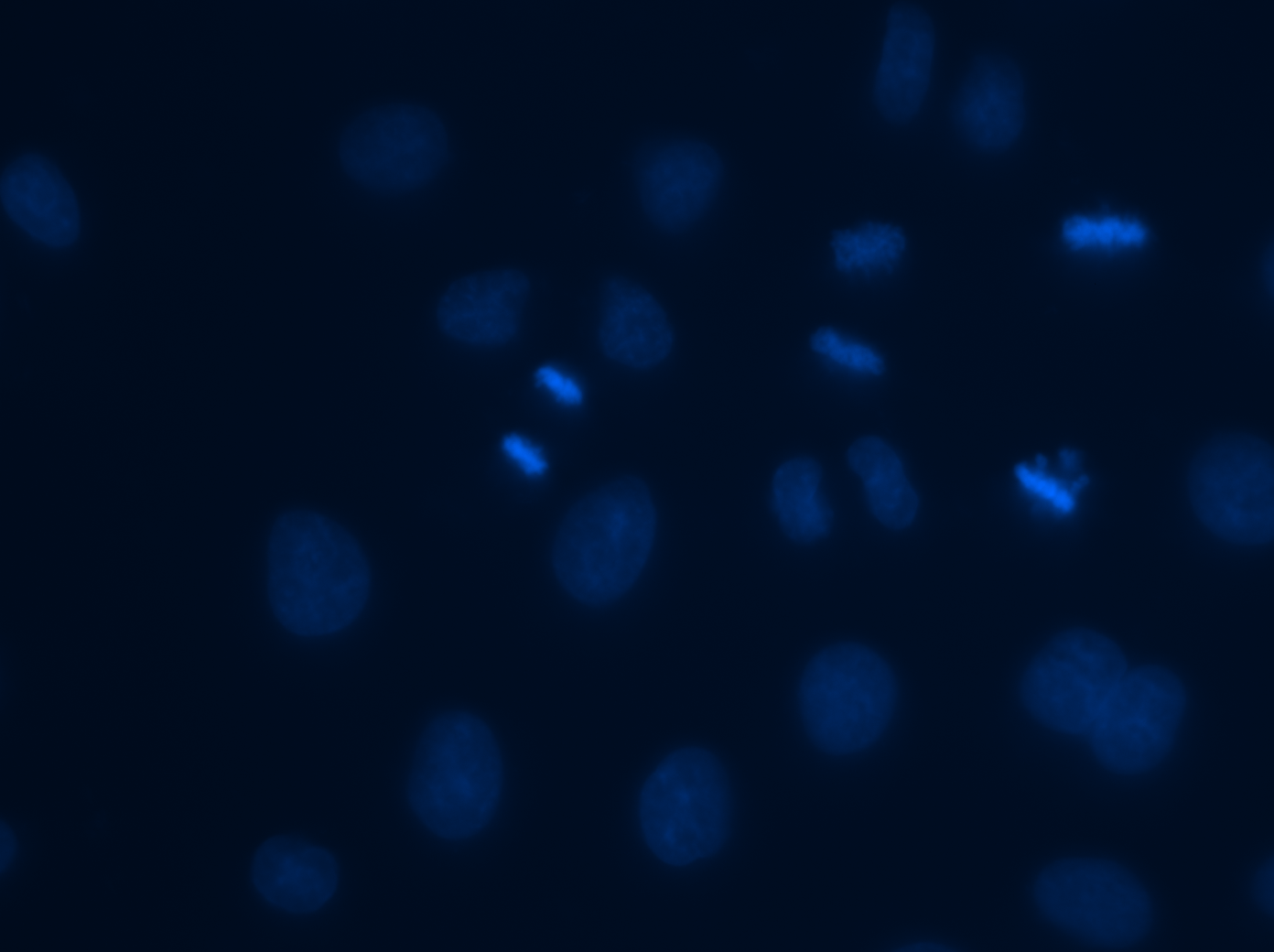

Supplement: Supplementary file 11 — Source data Fig. 5 [file 44318_2025_465_MOESM11_ESM.zip › EMBOJ-2025-120195-Figure 5-Source data/Figure 5/5H/ATRX-GFP siControl.tif]

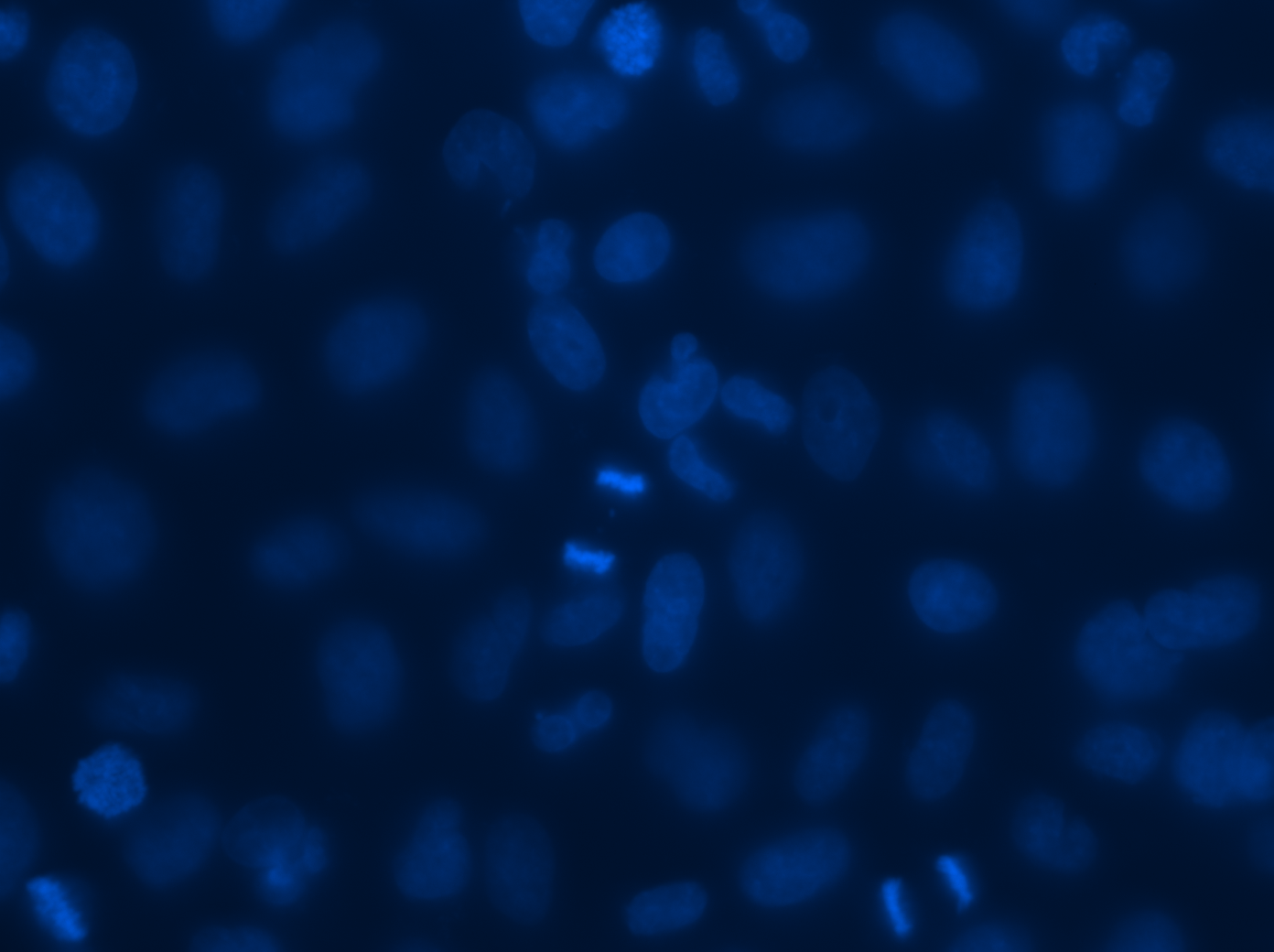

Supplement: Supplementary file 11 — Source data Fig. 5 [file 44318_2025_465_MOESM11_ESM.zip › EMBOJ-2025-120195-Figure 5-Source data/Figure 5/5H/HeLa siATRX #1.tif]

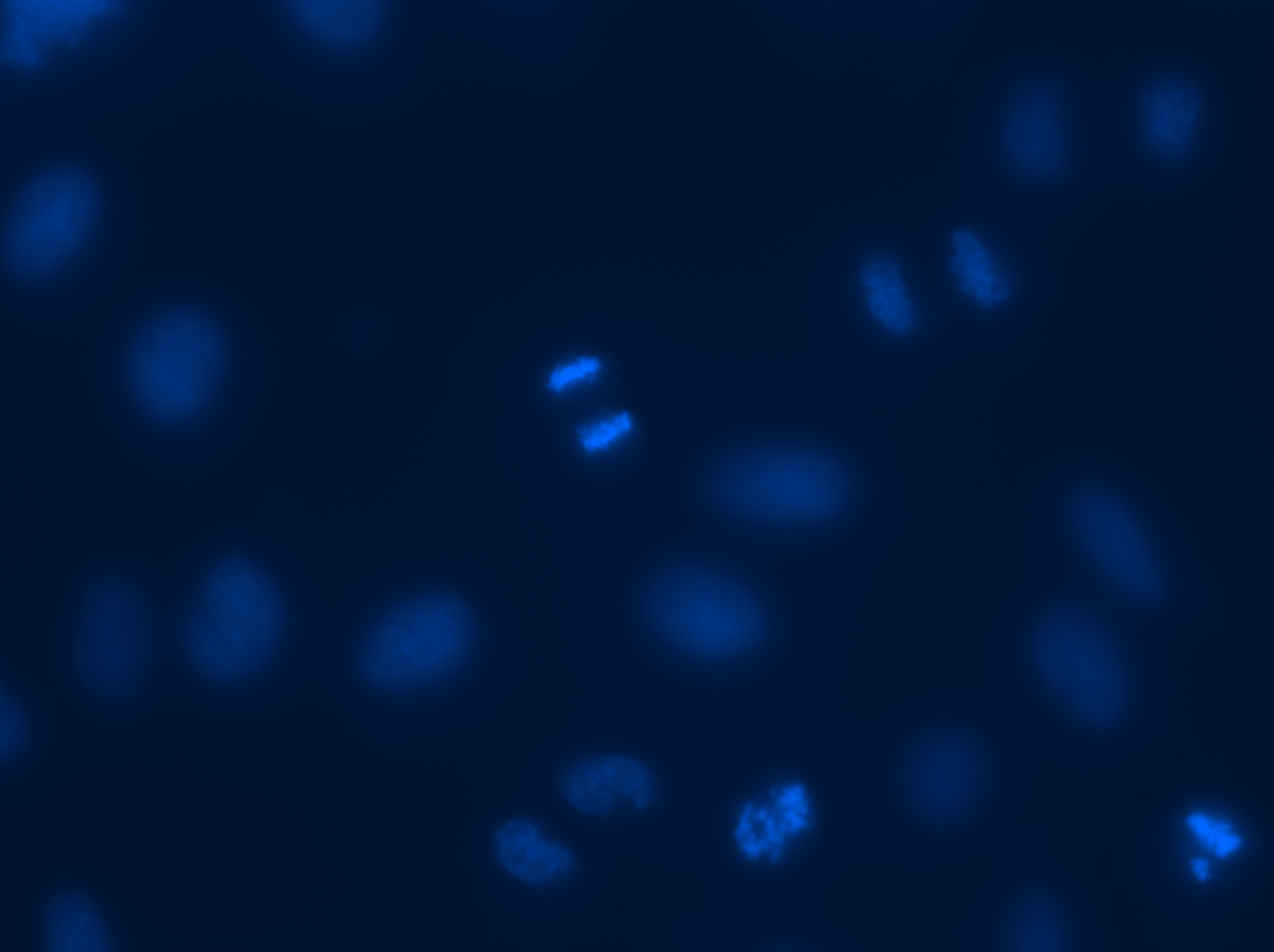

Supplement: Supplementary file 11 — Source data Fig. 5 [file 44318_2025_465_MOESM11_ESM.zip › EMBOJ-2025-120195-Figure 5-Source data/Figure 5/5H/HeLa siControl.tif]

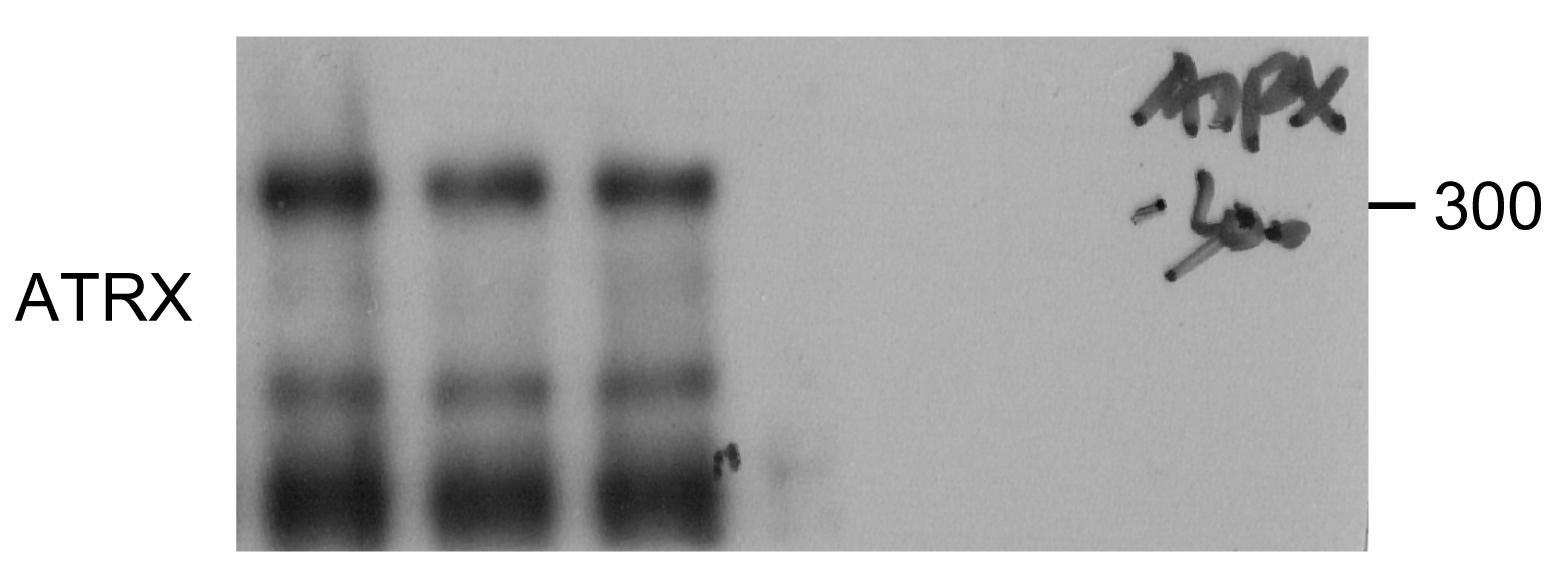

Supplement: Supplementary file 12 — Source data Fig. 6 [file 44318_2025_465_MOESM12_ESM.zip › EMBOJ-2025-120195-Figure 6-Source data/Figure 6/6A/western ATRX.tif]

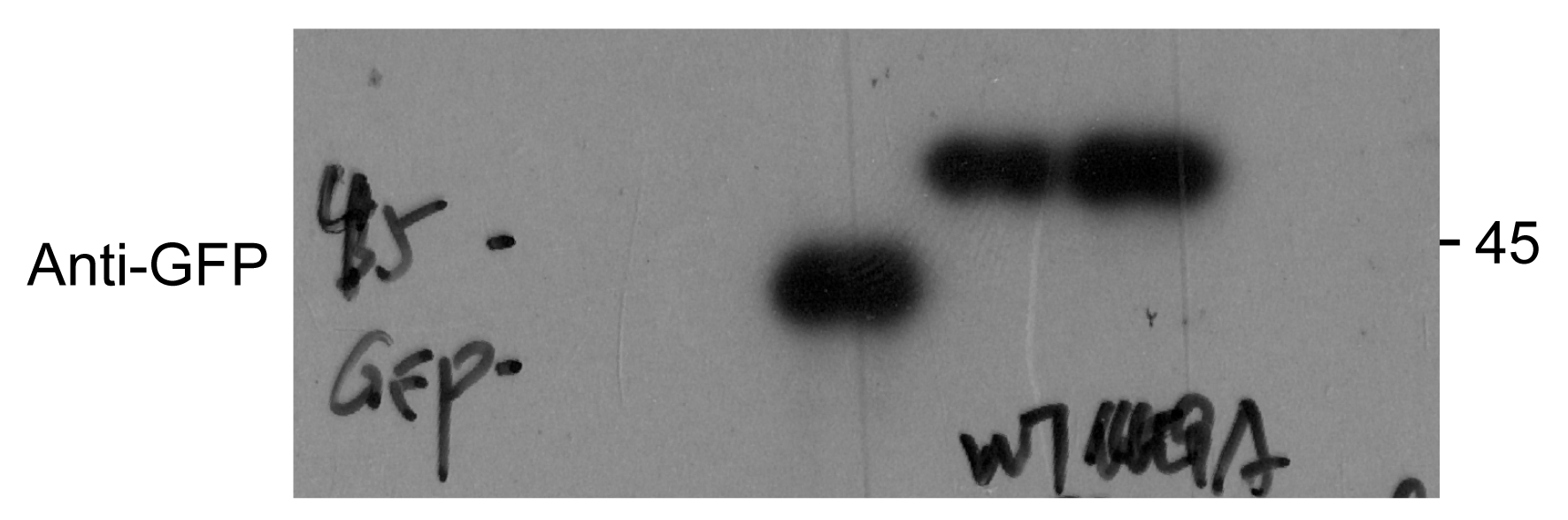

Supplement: Supplementary file 12 — Source data Fig. 6 [file 44318_2025_465_MOESM12_ESM.zip › EMBOJ-2025-120195-Figure 6-Source data/Figure 6/6A/western GFP.tif]

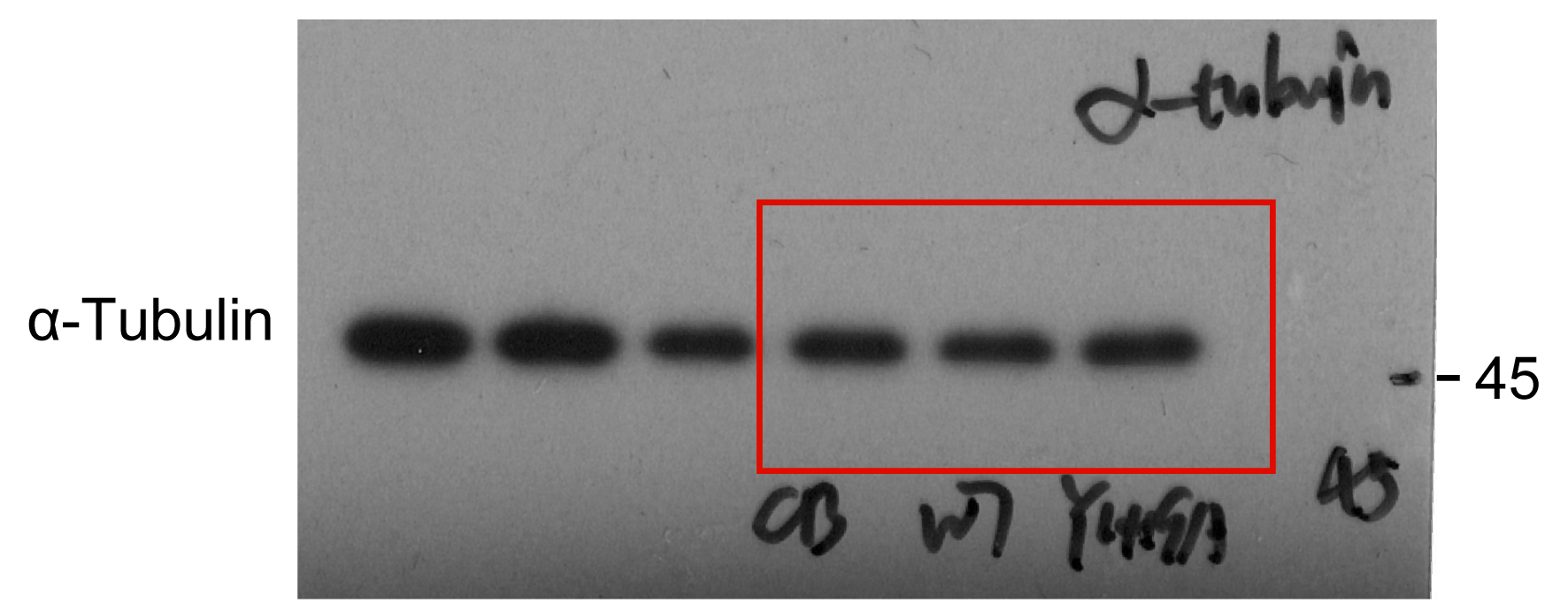

Supplement: Supplementary file 12 — Source data Fig. 6 [file 44318_2025_465_MOESM12_ESM.zip › EMBOJ-2025-120195-Figure 6-Source data/Figure 6/6A/western α-Tubulin-.tif]

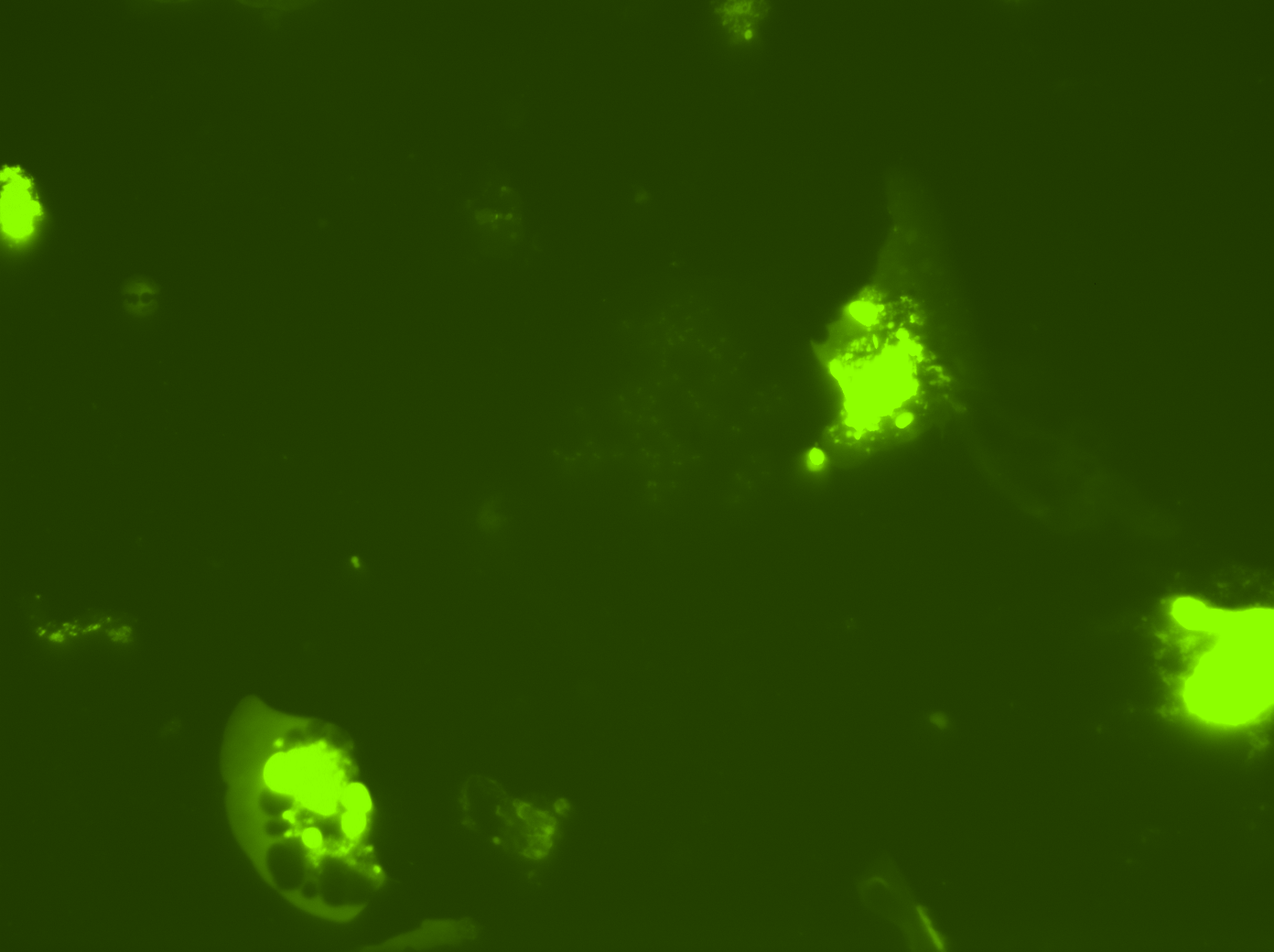

Supplement: Supplementary file 12 — Source data Fig. 6 [file 44318_2025_465_MOESM12_ESM.zip › EMBOJ-2025-120195-Figure 6-Source data/Figure 6/6C/CB-ATRX (1394-1443)-GFP-siATRX #1 GFP.tif]

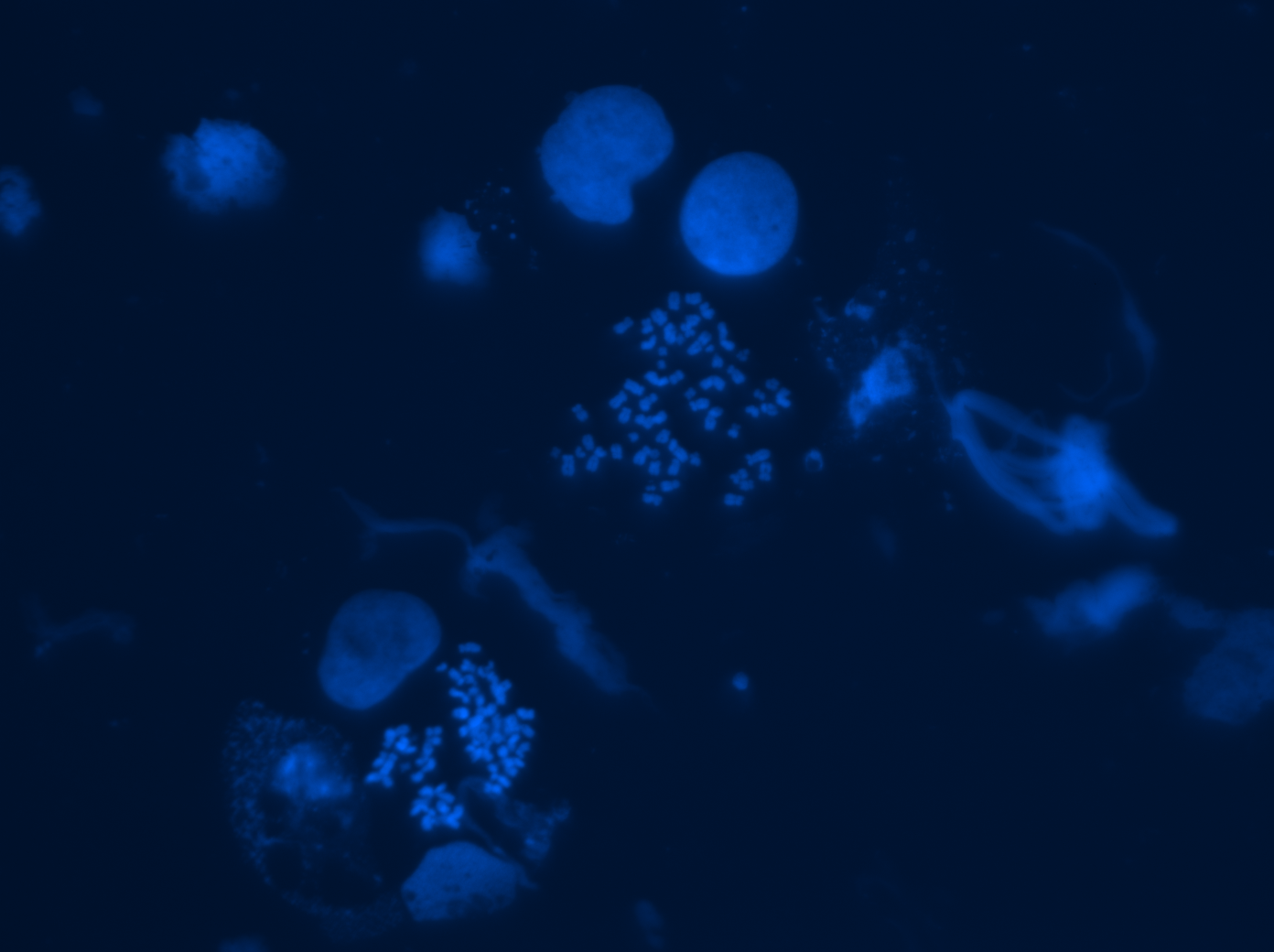

Supplement: Supplementary file 12 — Source data Fig. 6 [file 44318_2025_465_MOESM12_ESM.zip › EMBOJ-2025-120195-Figure 6-Source data/Figure 6/6C/CB-ATRX (1394-1443)-GFP-siATRX #1 DNA.tif]

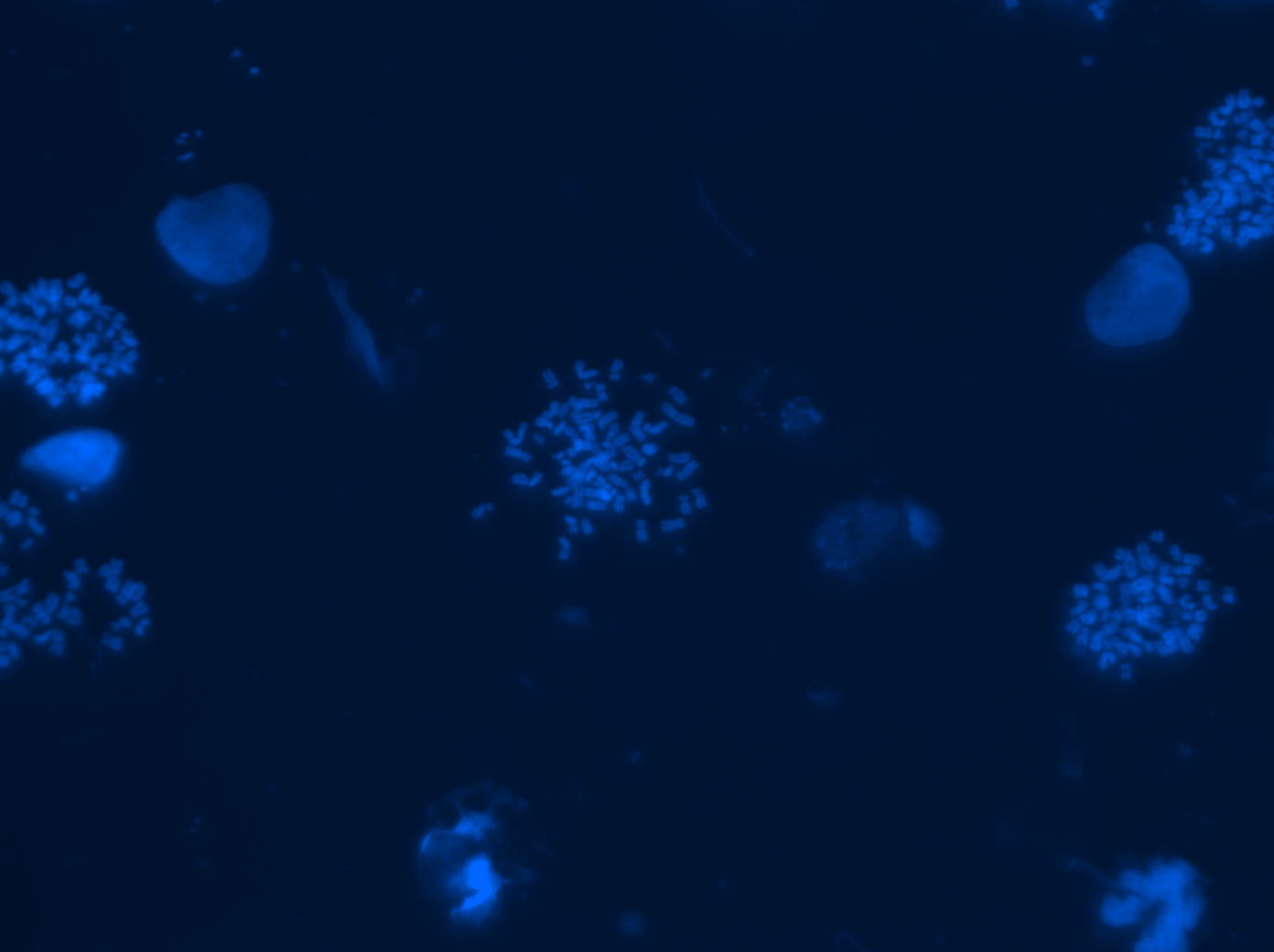

Supplement: Supplementary file 12 — Source data Fig. 6 [file 44318_2025_465_MOESM12_ESM.zip › EMBOJ-2025-120195-Figure 6-Source data/Figure 6/6C/CB-ATRX (1394-1443)-GFP-siControl DNA.tif]

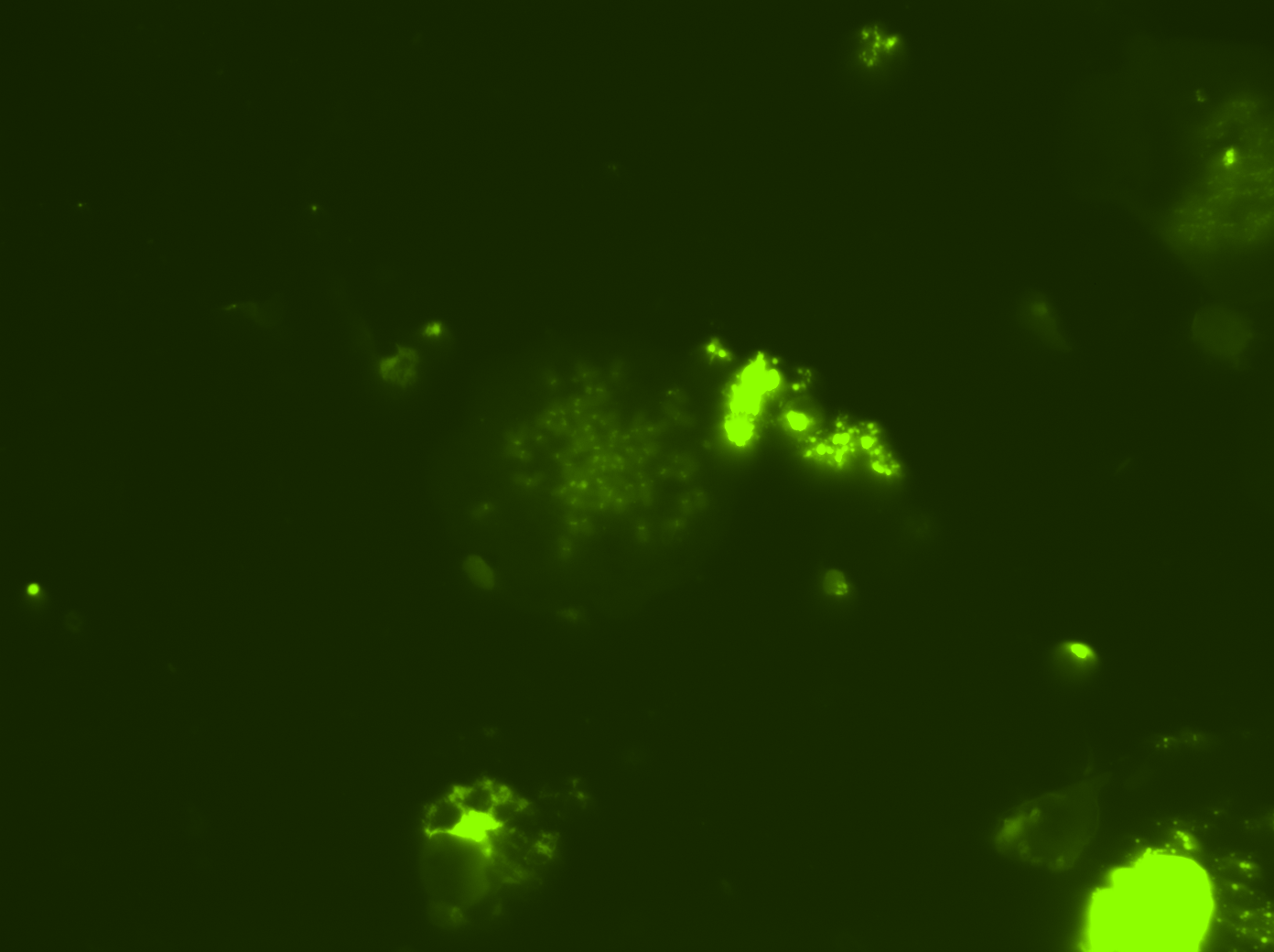

Supplement: Supplementary file 12 — Source data Fig. 6 [file 44318_2025_465_MOESM12_ESM.zip › EMBOJ-2025-120195-Figure 6-Source data/Figure 6/6C/CB-ATRX (1394-1443)-GFP-siControl GFP.tif]

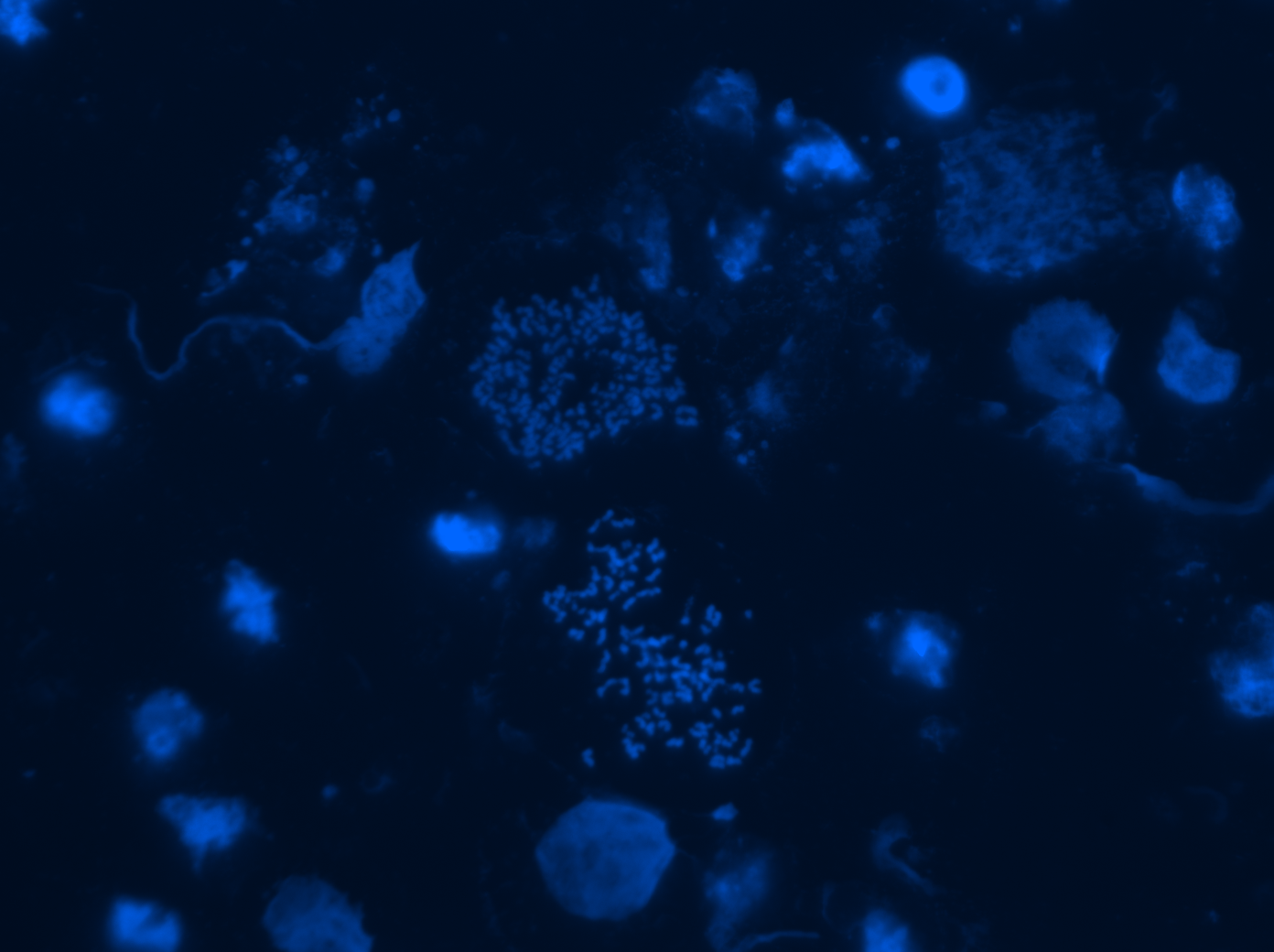

Supplement: Supplementary file 12 — Source data Fig. 6 [file 44318_2025_465_MOESM12_ESM.zip › EMBOJ-2025-120195-Figure 6-Source data/Figure 6/6C/CB-ATRX (1394-1443)-GFP-Y1419A-siATRX #1 DNA.tif]

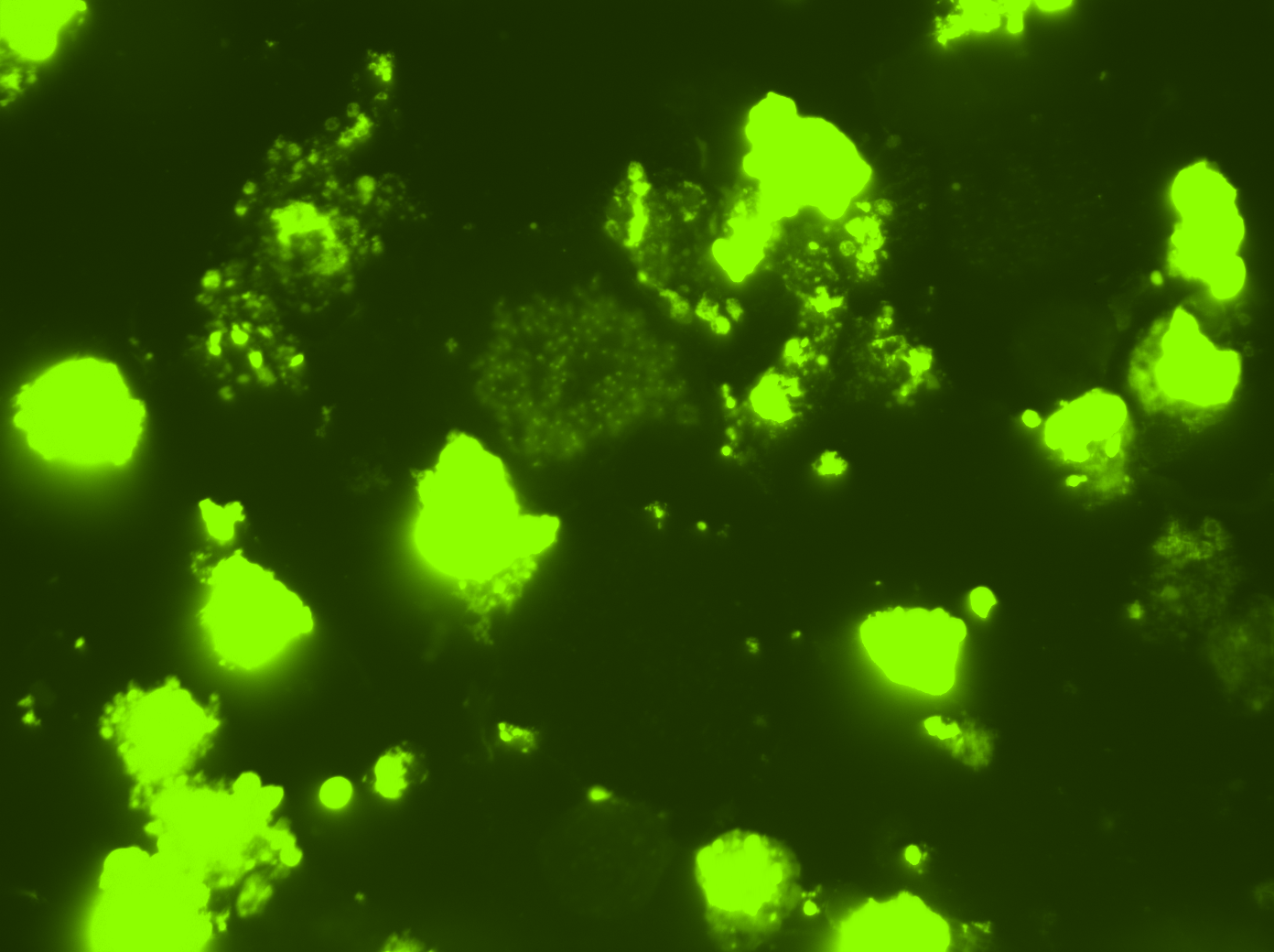

Supplement: Supplementary file 12 — Source data Fig. 6 [file 44318_2025_465_MOESM12_ESM.zip › EMBOJ-2025-120195-Figure 6-Source data/Figure 6/6C/CB-ATRX (1394-1443)-GFP-Y1419A-siATRX #1 GFP.tif]

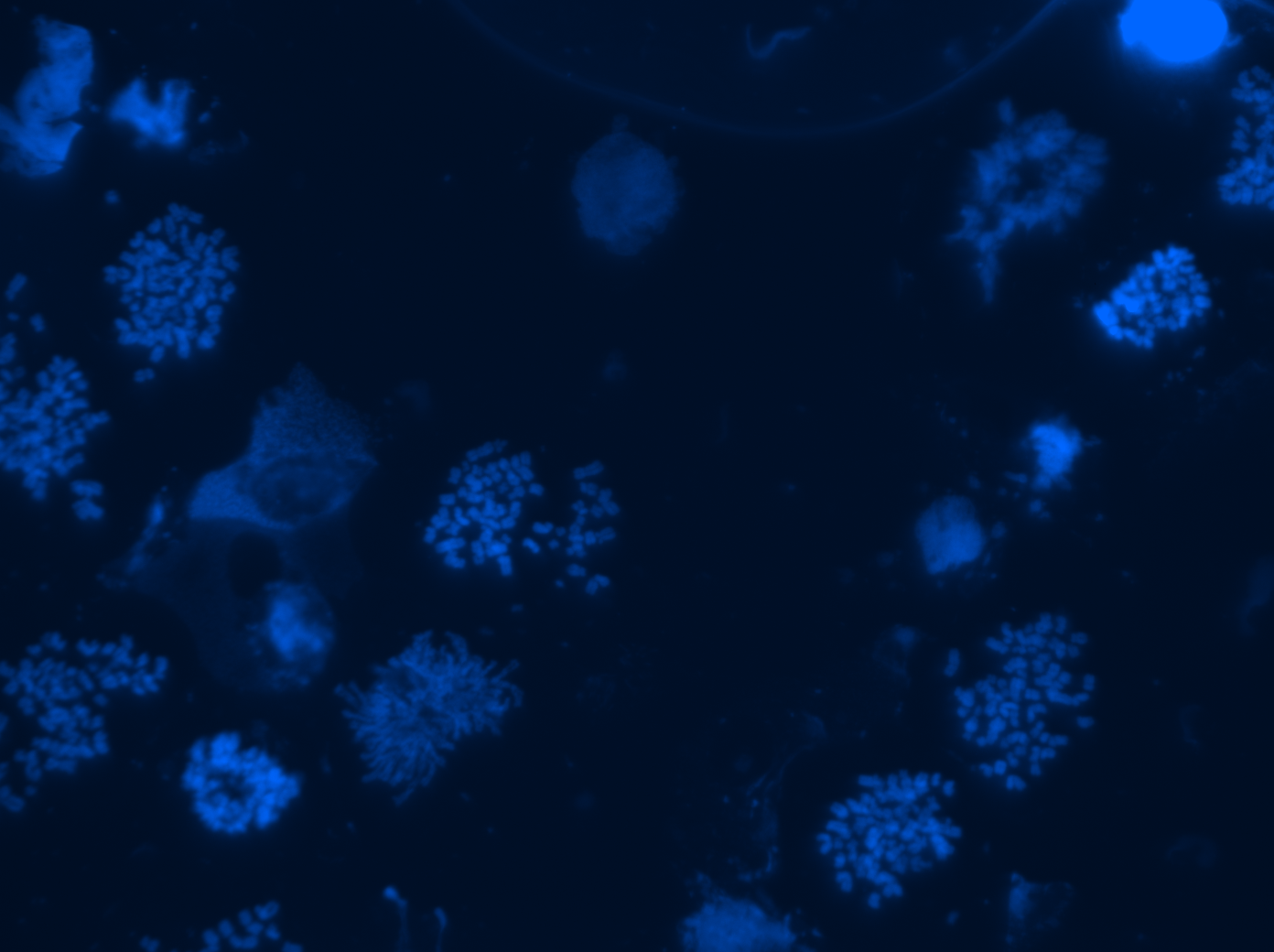

Supplement: Supplementary file 12 — Source data Fig. 6 [file 44318_2025_465_MOESM12_ESM.zip › EMBOJ-2025-120195-Figure 6-Source data/Figure 6/6C/CB-ATRX (1394-1443)-GFP-Y1419A-siControl DNA.tif]

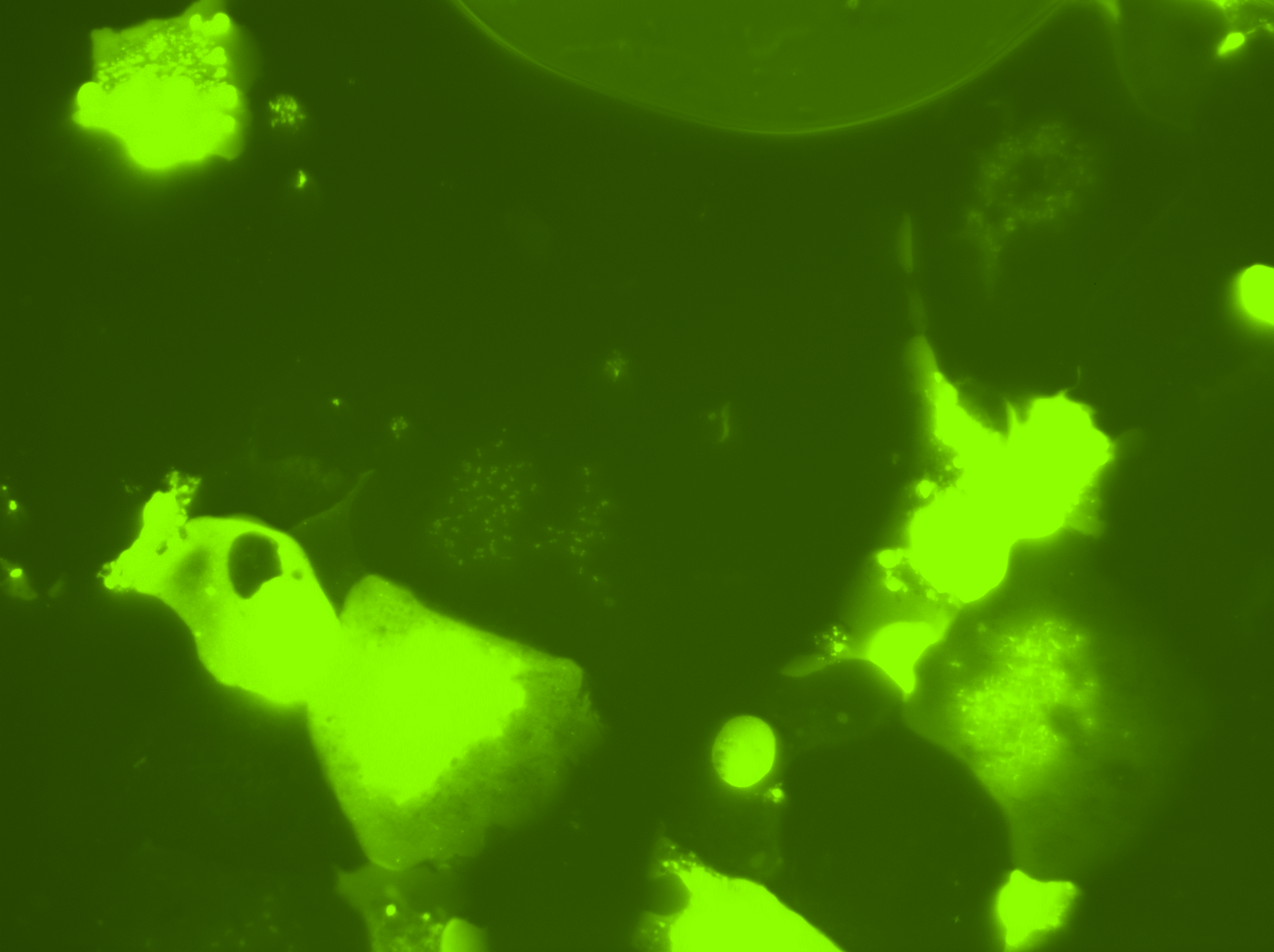

Supplement: Supplementary file 12 — Source data Fig. 6 [file 44318_2025_465_MOESM12_ESM.zip › EMBOJ-2025-120195-Figure 6-Source data/Figure 6/6C/CB-ATRX (1394-1443)-GFP-Y1419A-siControl GFP.tif]

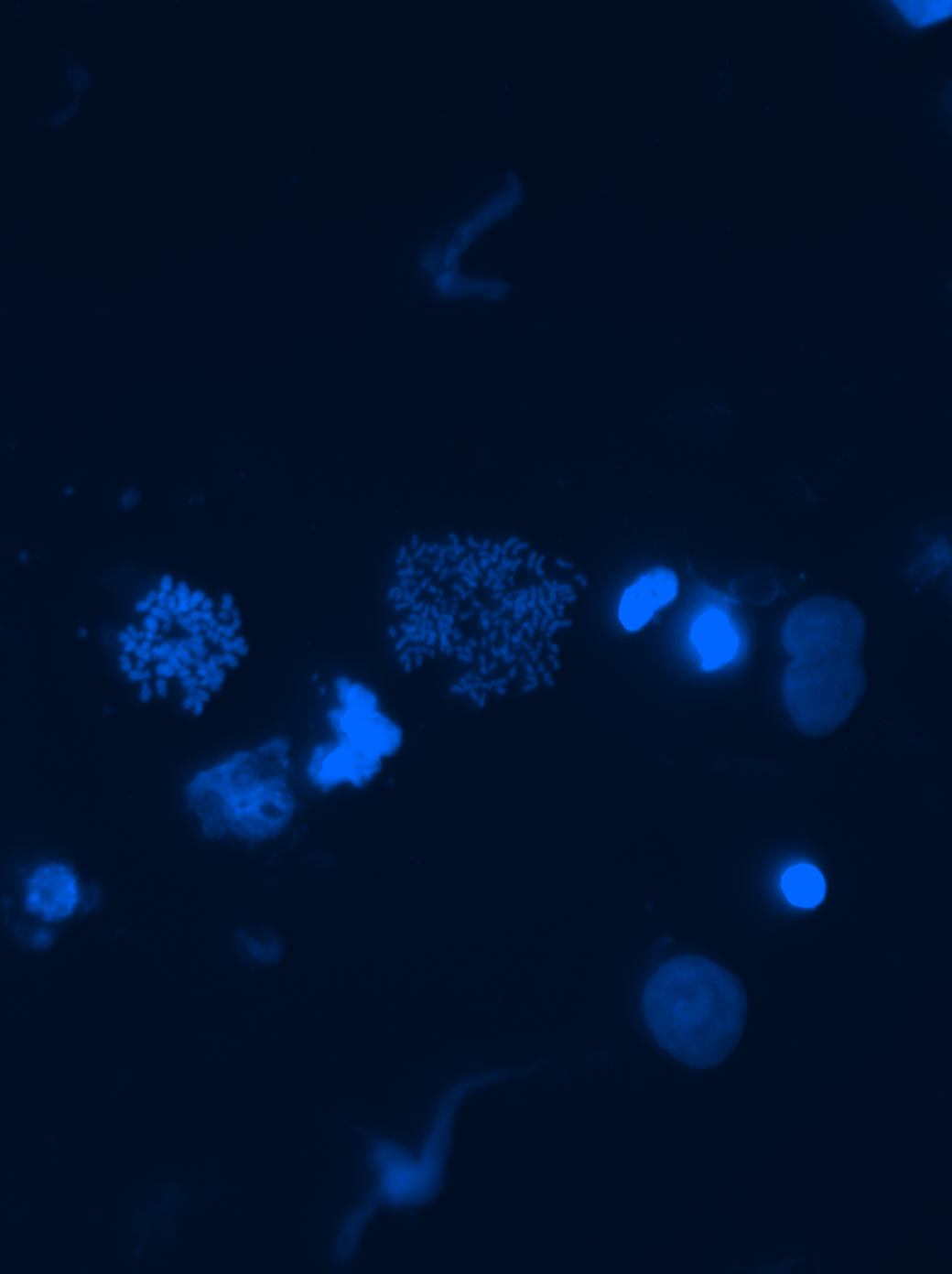

Supplement: Supplementary file 12 — Source data Fig. 6 [file 44318_2025_465_MOESM12_ESM.zip › EMBOJ-2025-120195-Figure 6-Source data/Figure 6/6C/CB-GFP-siATRX #1 DNA.tif]

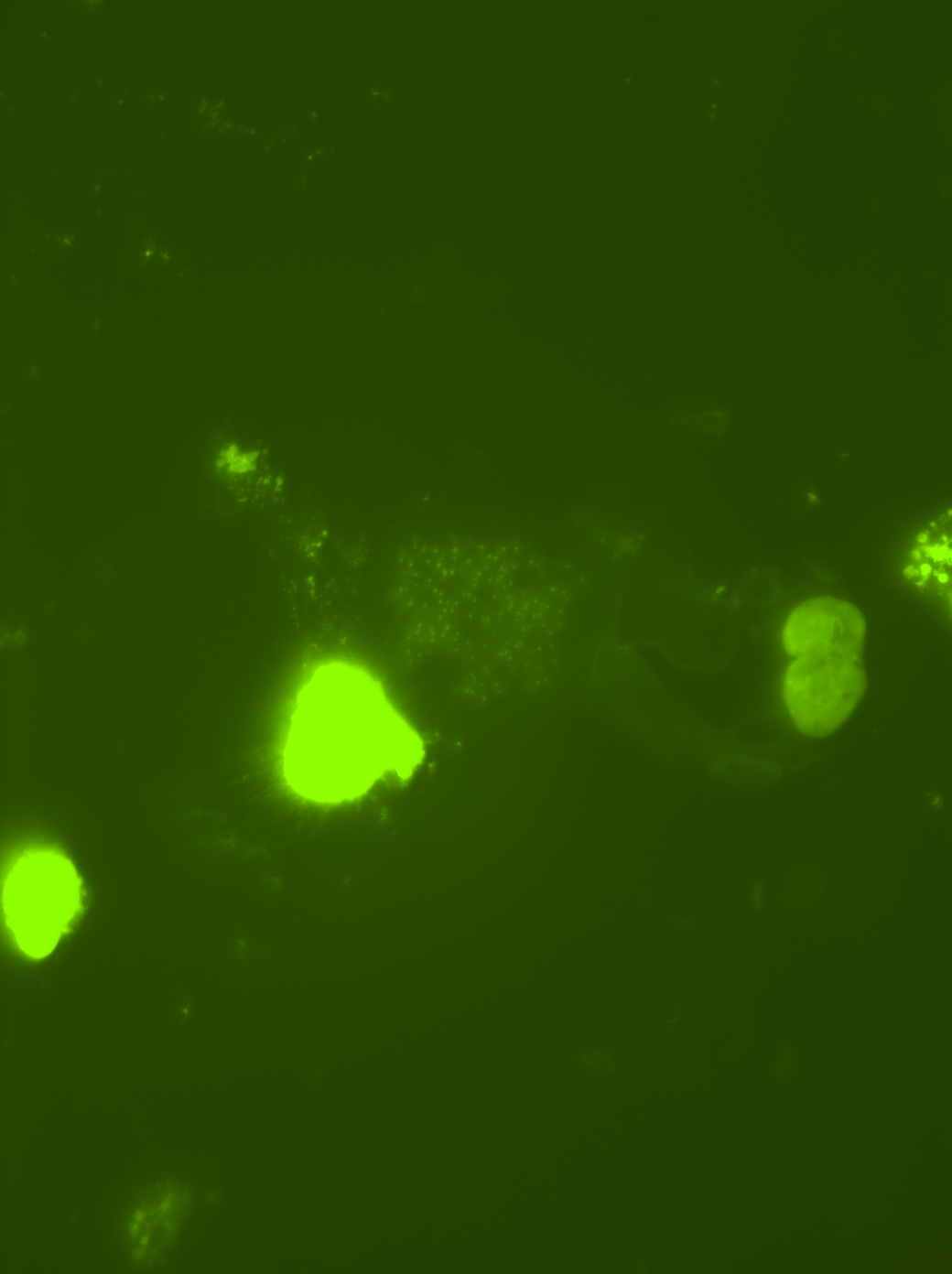

Supplement: Supplementary file 12 — Source data Fig. 6 [file 44318_2025_465_MOESM12_ESM.zip › EMBOJ-2025-120195-Figure 6-Source data/Figure 6/6C/CB-GFP-siATRX #1 GFP.tif]

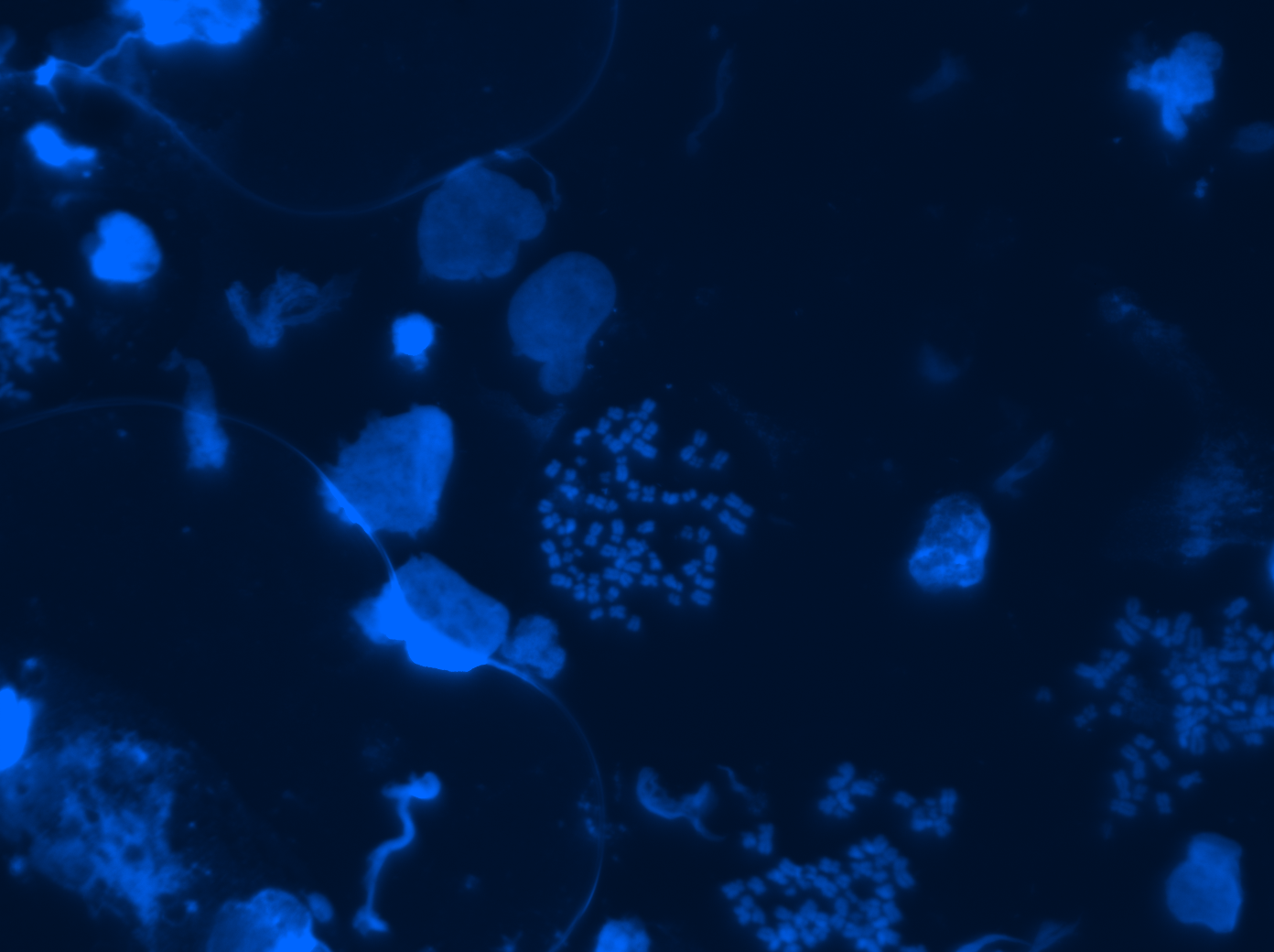

Supplement: Supplementary file 12 — Source data Fig. 6 [file 44318_2025_465_MOESM12_ESM.zip › EMBOJ-2025-120195-Figure 6-Source data/Figure 6/6C/CB-GFP-siControl DNA.tif]

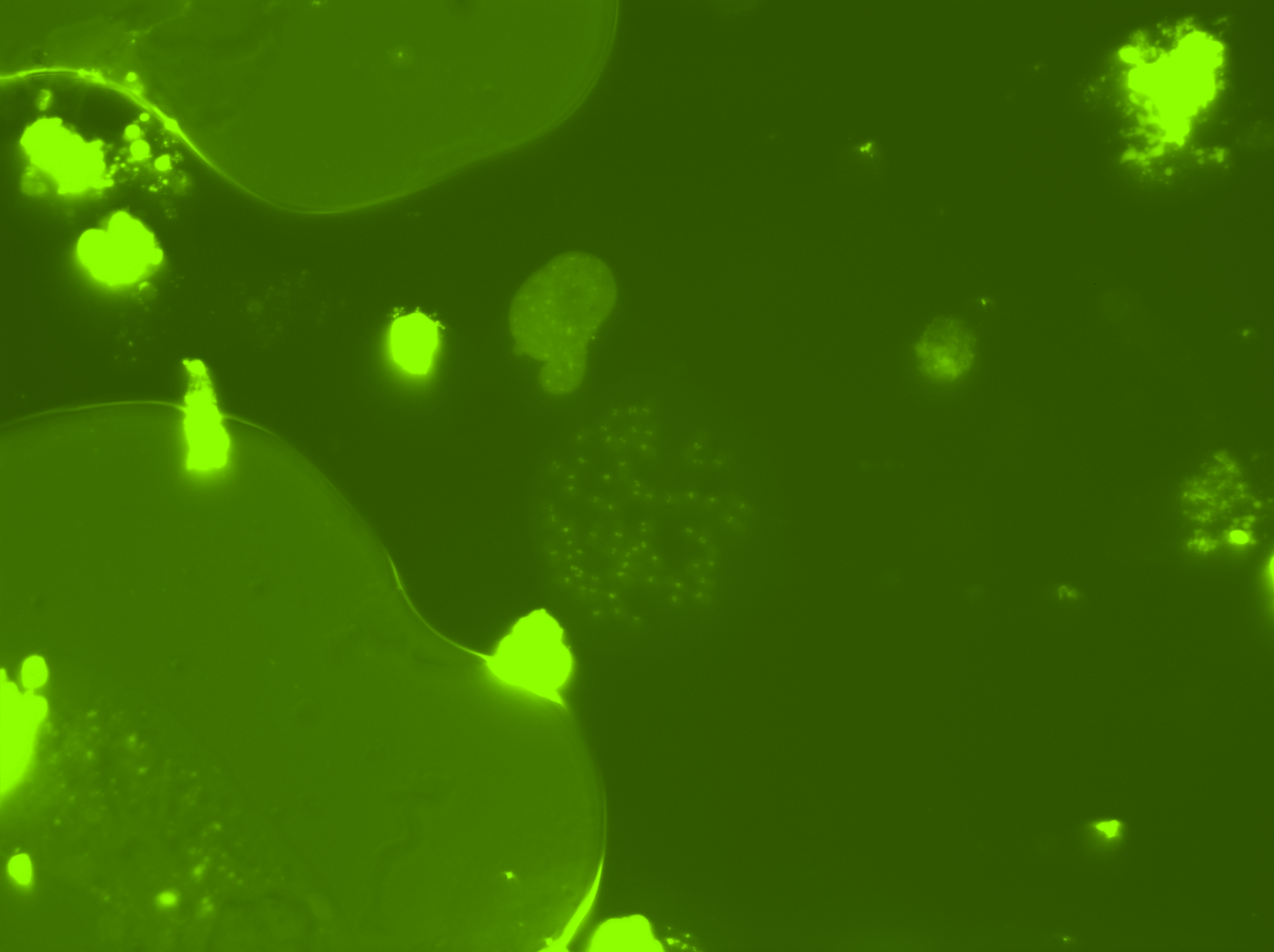

Supplement: Supplementary file 12 — Source data Fig. 6 [file 44318_2025_465_MOESM12_ESM.zip › EMBOJ-2025-120195-Figure 6-Source data/Figure 6/6C/CB-GFP-siControl GFP.tif]

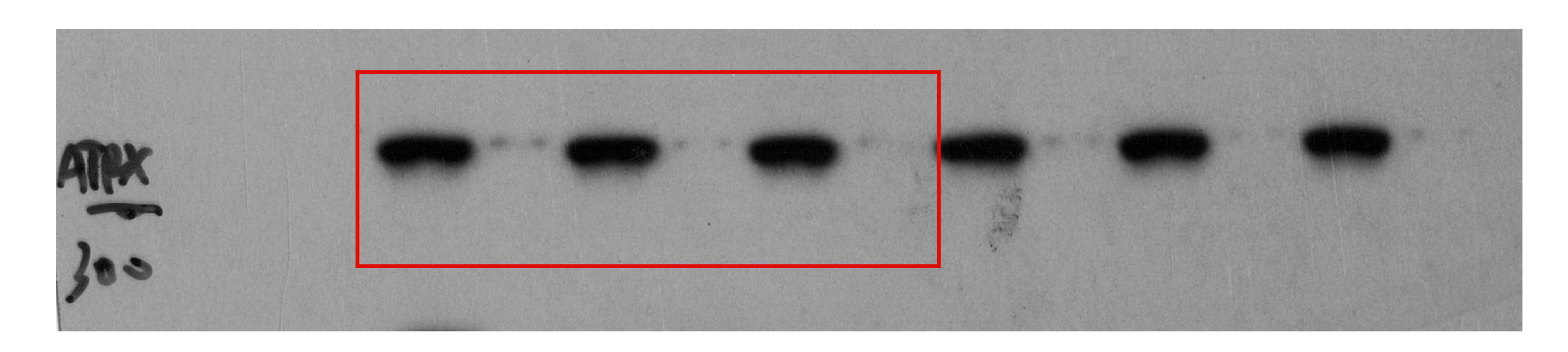

Supplement: Supplementary file 12 — Source data Fig. 6 [file 44318_2025_465_MOESM12_ESM.zip › EMBOJ-2025-120195-Figure 6-Source data/Figure 6/6D/western ATRX.tif]

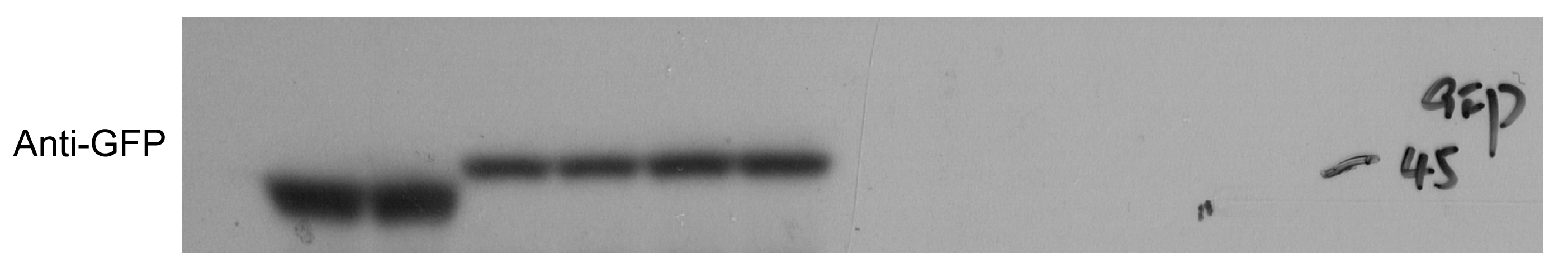

Supplement: Supplementary file 12 — Source data Fig. 6 [file 44318_2025_465_MOESM12_ESM.zip › EMBOJ-2025-120195-Figure 6-Source data/Figure 6/6D/western GFP.tif]

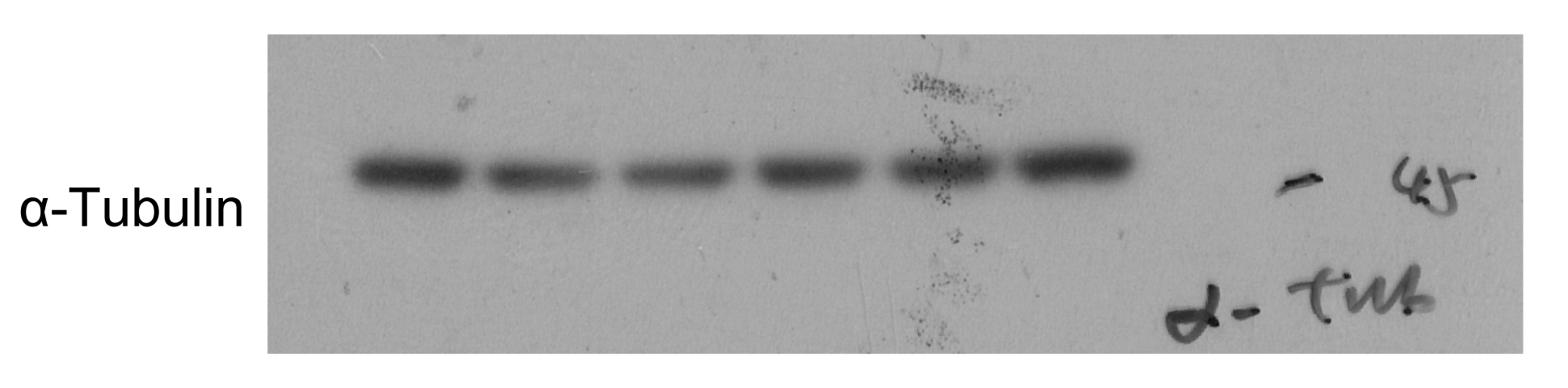

Supplement: Supplementary file 12 — Source data Fig. 6 [file 44318_2025_465_MOESM12_ESM.zip › EMBOJ-2025-120195-Figure 6-Source data/Figure 6/6D/western α-Tubulin.tif]

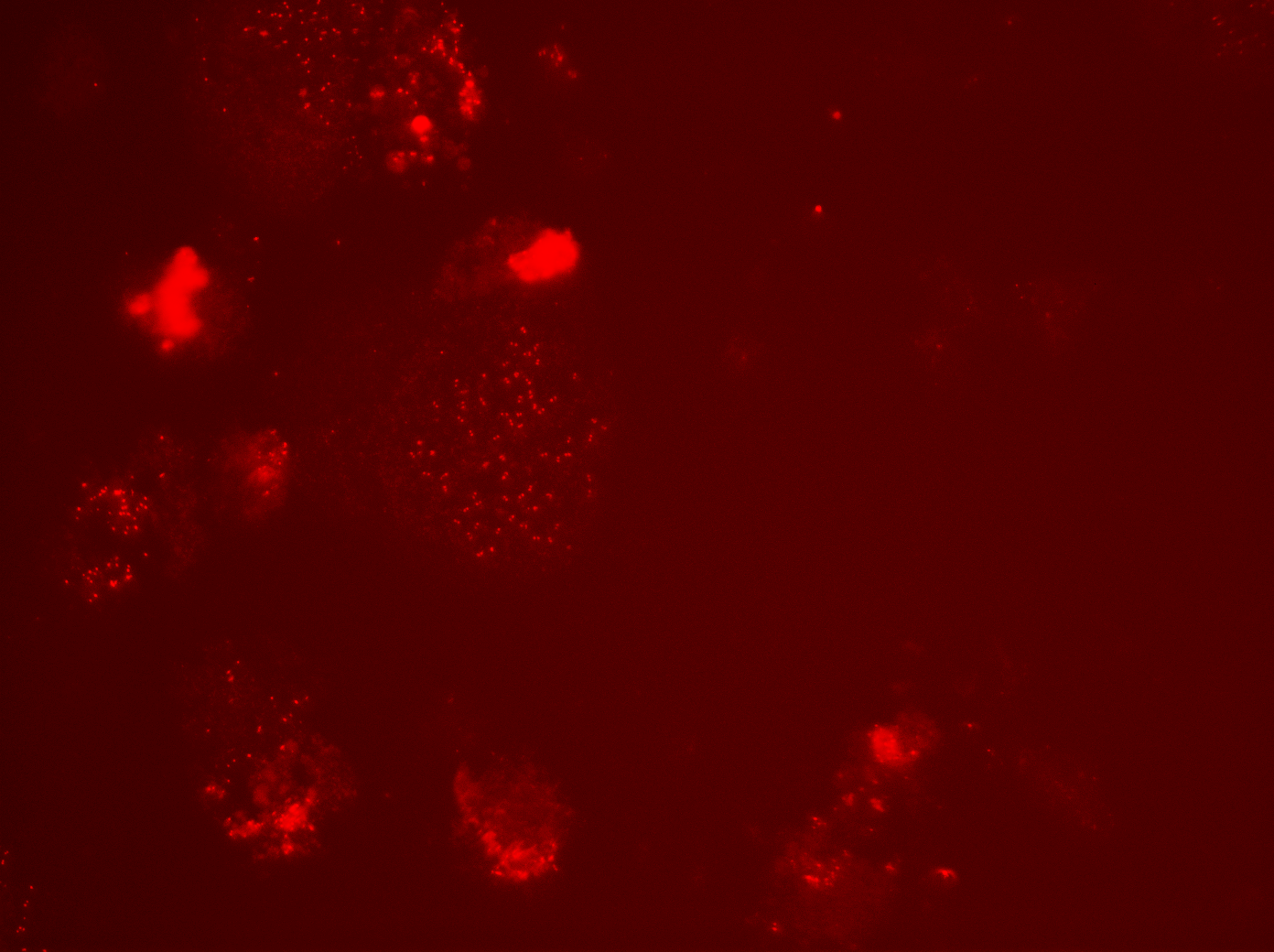

Supplement: Supplementary file 12 — Source data Fig. 6 [file 44318_2025_465_MOESM12_ESM.zip › EMBOJ-2025-120195-Figure 6-Source data/Figure 6/6H/CB-ATRX (1394-1443)-GFP-siATRX #1 CENP-A.tif]

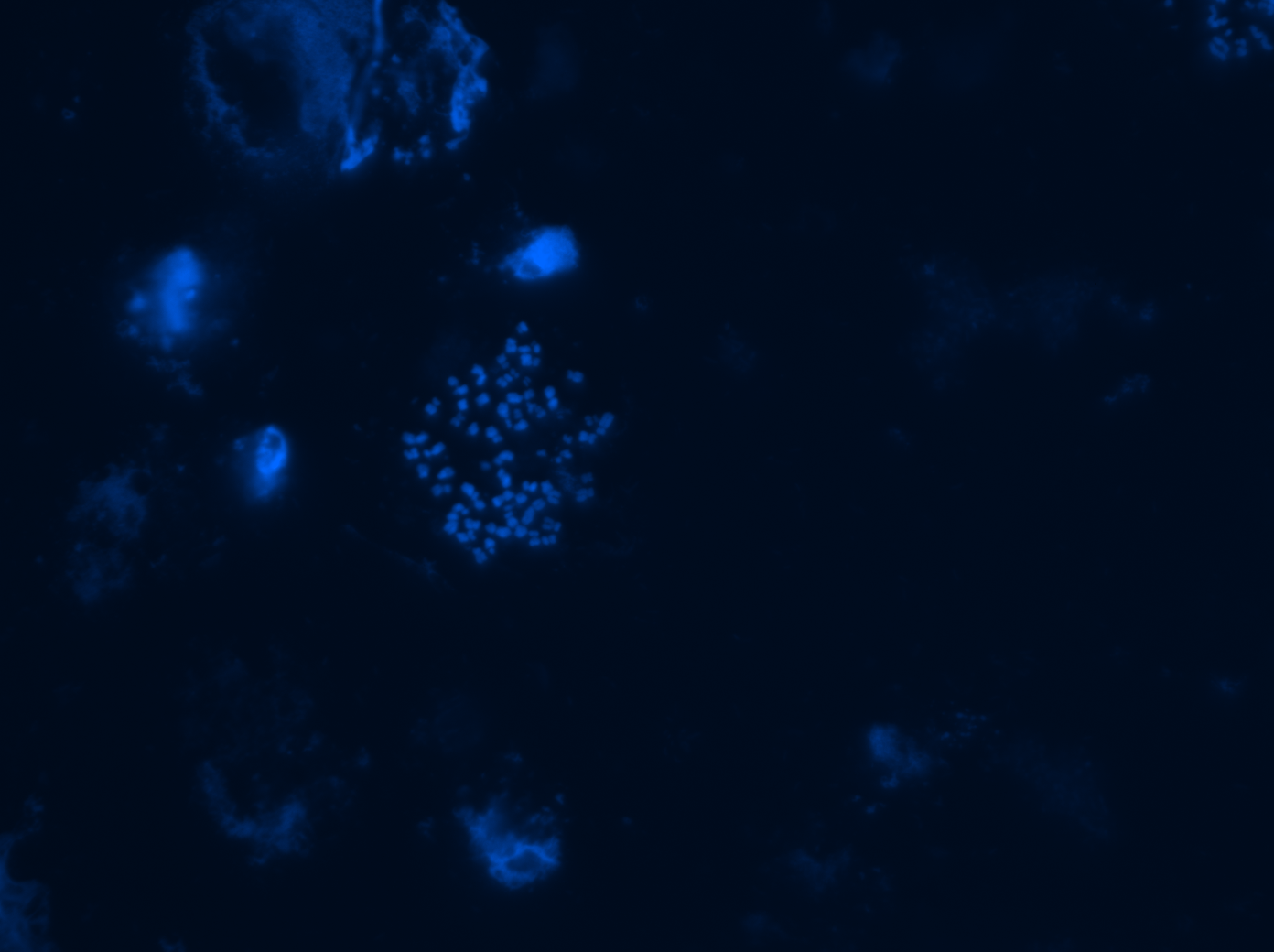

Supplement: Supplementary file 12 — Source data Fig. 6 [file 44318_2025_465_MOESM12_ESM.zip › EMBOJ-2025-120195-Figure 6-Source data/Figure 6/6H/CB-ATRX (1394-1443)-GFP-siATRX #1 DNA.tif]

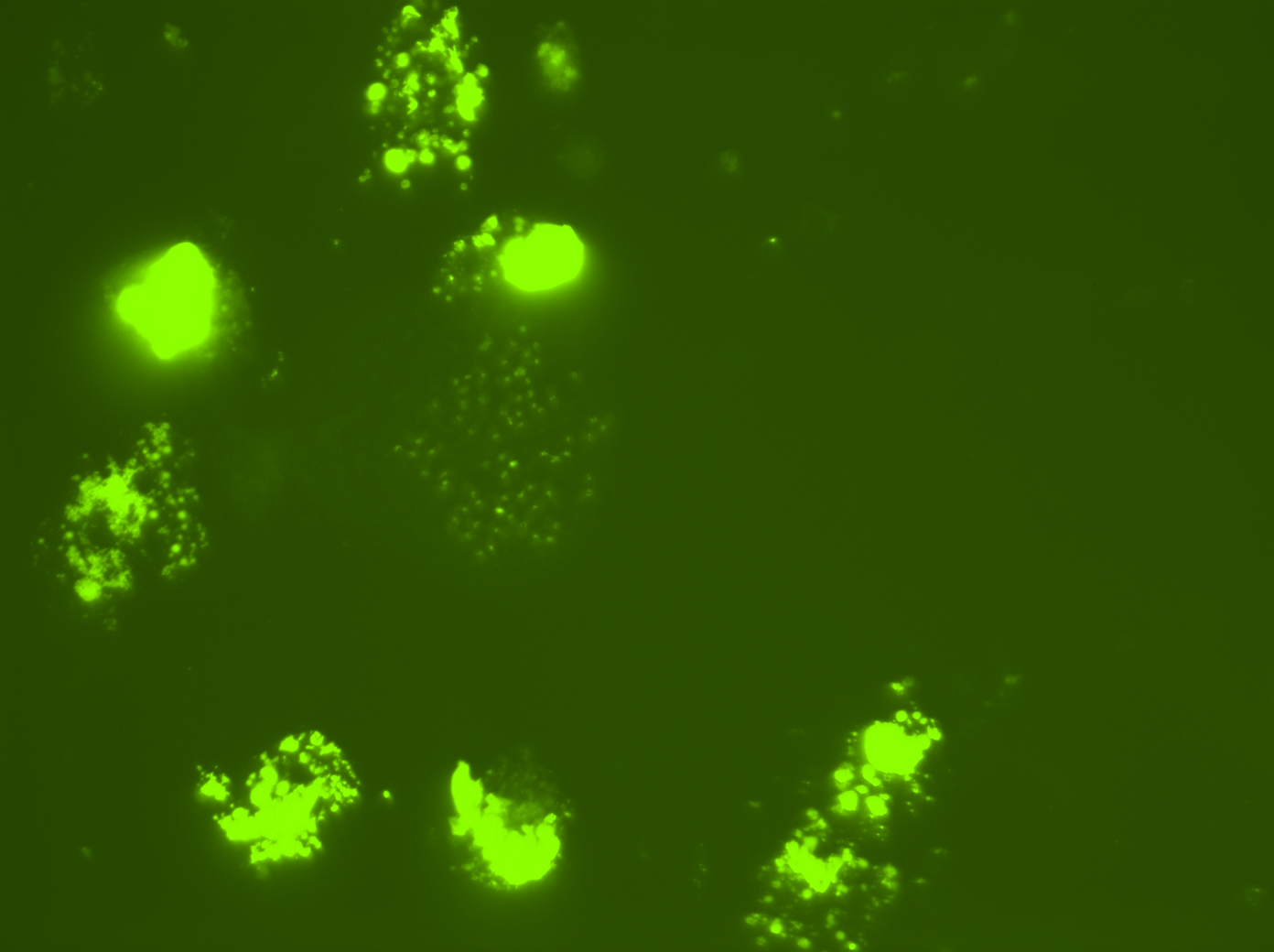

Supplement: Supplementary file 12 — Source data Fig. 6 [file 44318_2025_465_MOESM12_ESM.zip › EMBOJ-2025-120195-Figure 6-Source data/Figure 6/6H/CB-ATRX (1394-1443)-GFP-siATRX #1 GFP.tif]

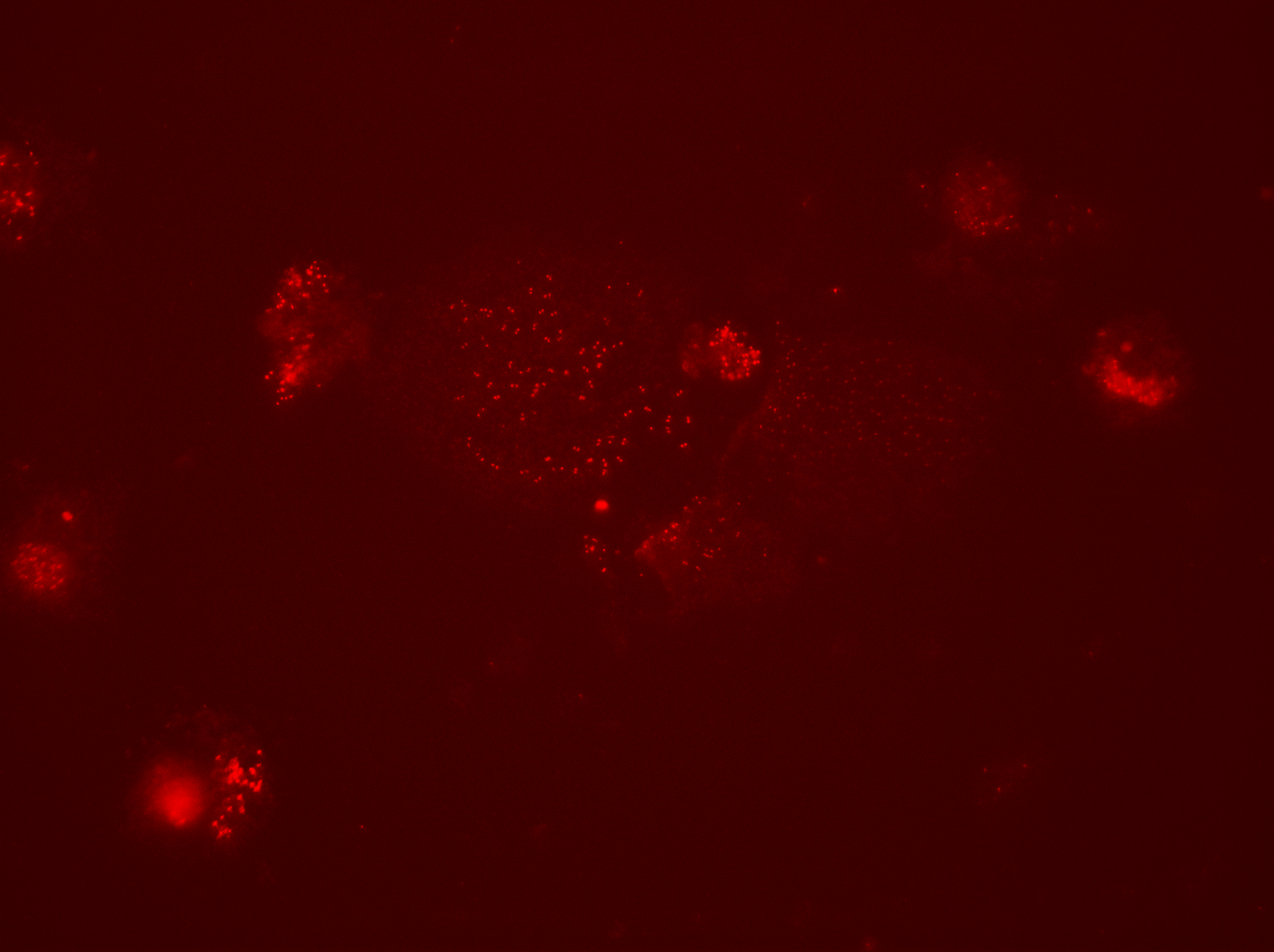

Supplement: Supplementary file 12 — Source data Fig. 6 [file 44318_2025_465_MOESM12_ESM.zip › EMBOJ-2025-120195-Figure 6-Source data/Figure 6/6H/CB-ATRX (1394-1443)-GFP-siControl CENP-A.tif]

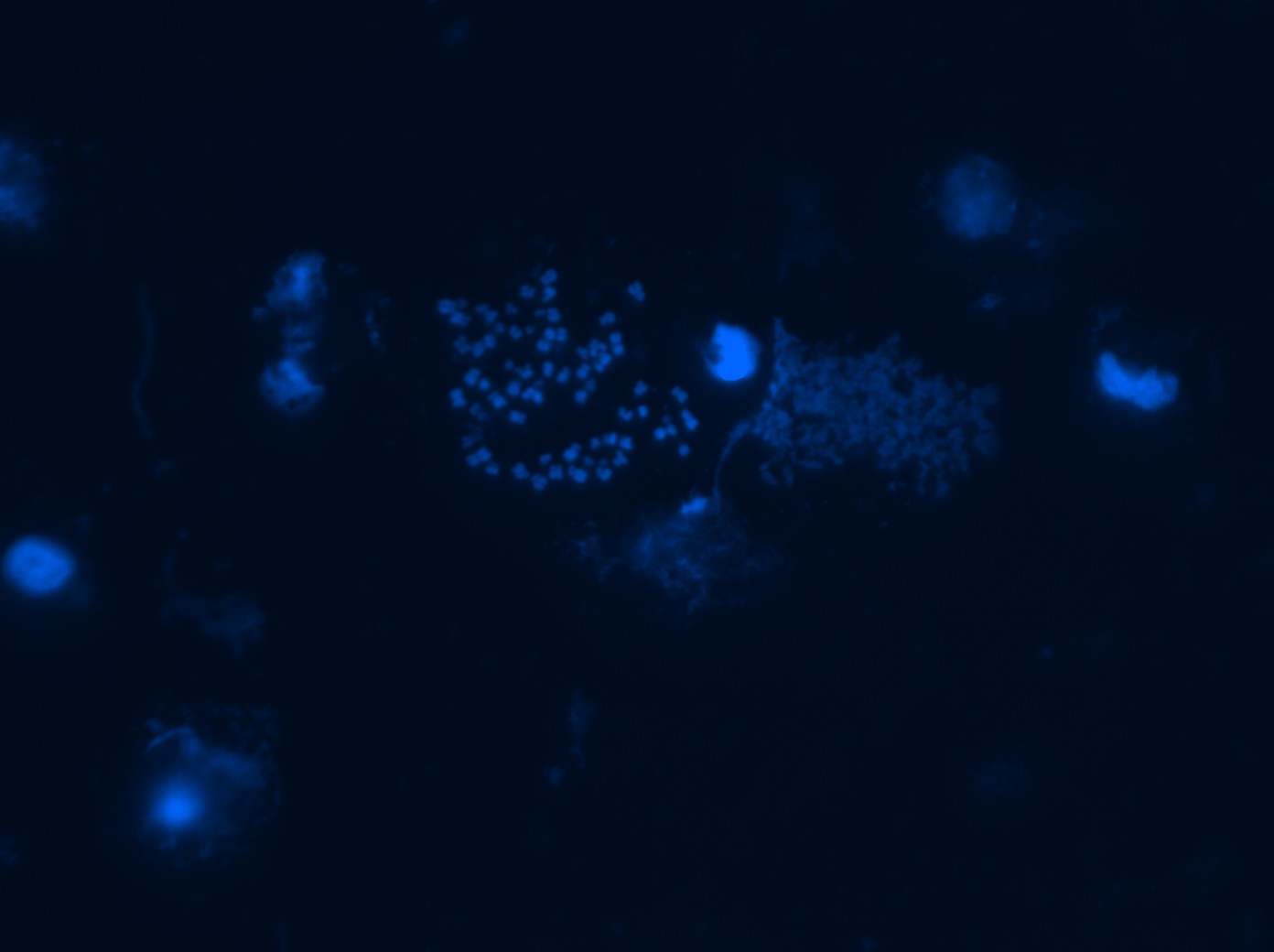

Supplement: Supplementary file 12 — Source data Fig. 6 [file 44318_2025_465_MOESM12_ESM.zip › EMBOJ-2025-120195-Figure 6-Source data/Figure 6/6H/CB-ATRX (1394-1443)-GFP-siControl DNA.tif]

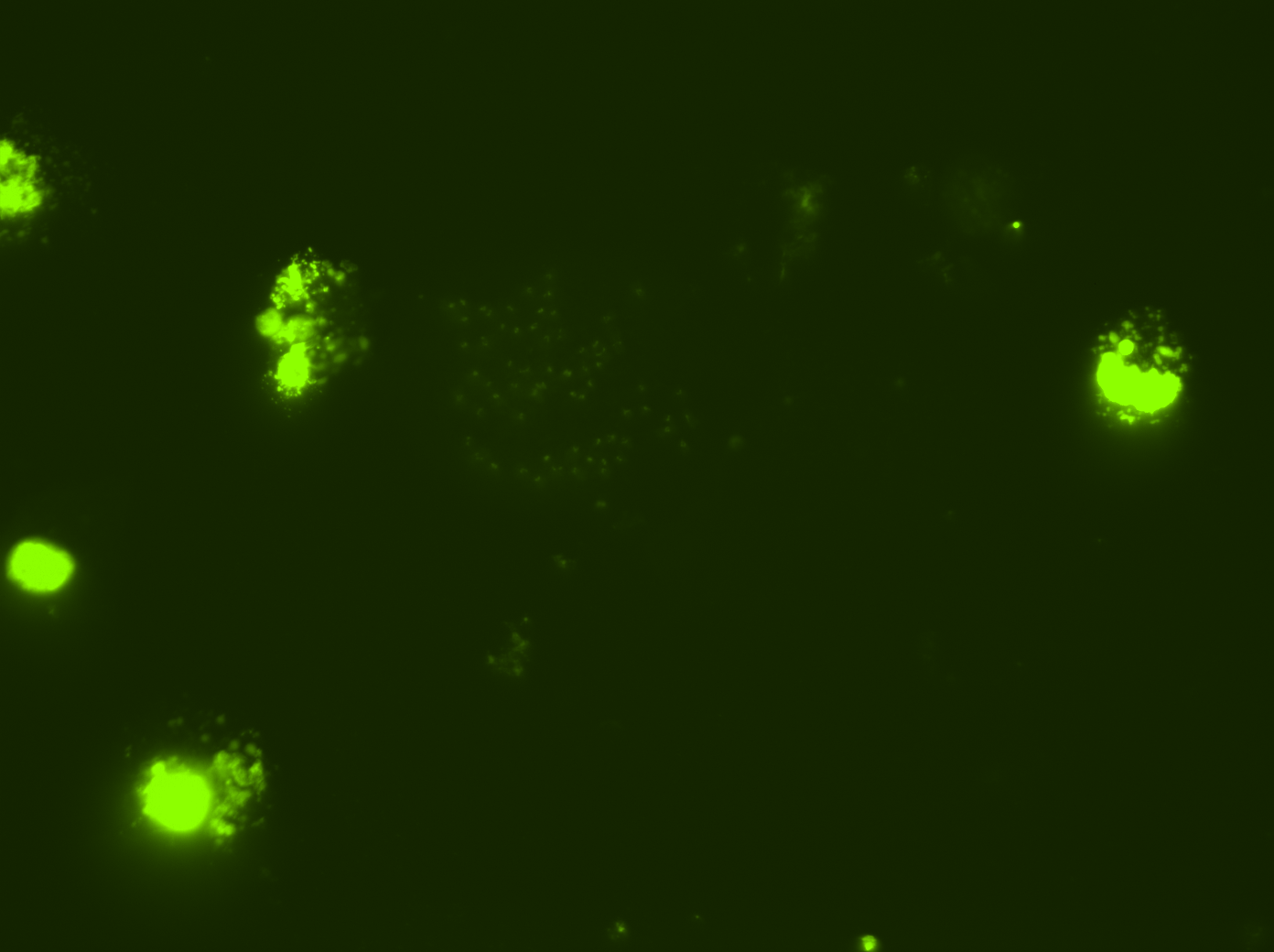

Supplement: Supplementary file 12 — Source data Fig. 6 [file 44318_2025_465_MOESM12_ESM.zip › EMBOJ-2025-120195-Figure 6-Source data/Figure 6/6H/CB-ATRX (1394-1443)-GFP-siControl GFP.tif]

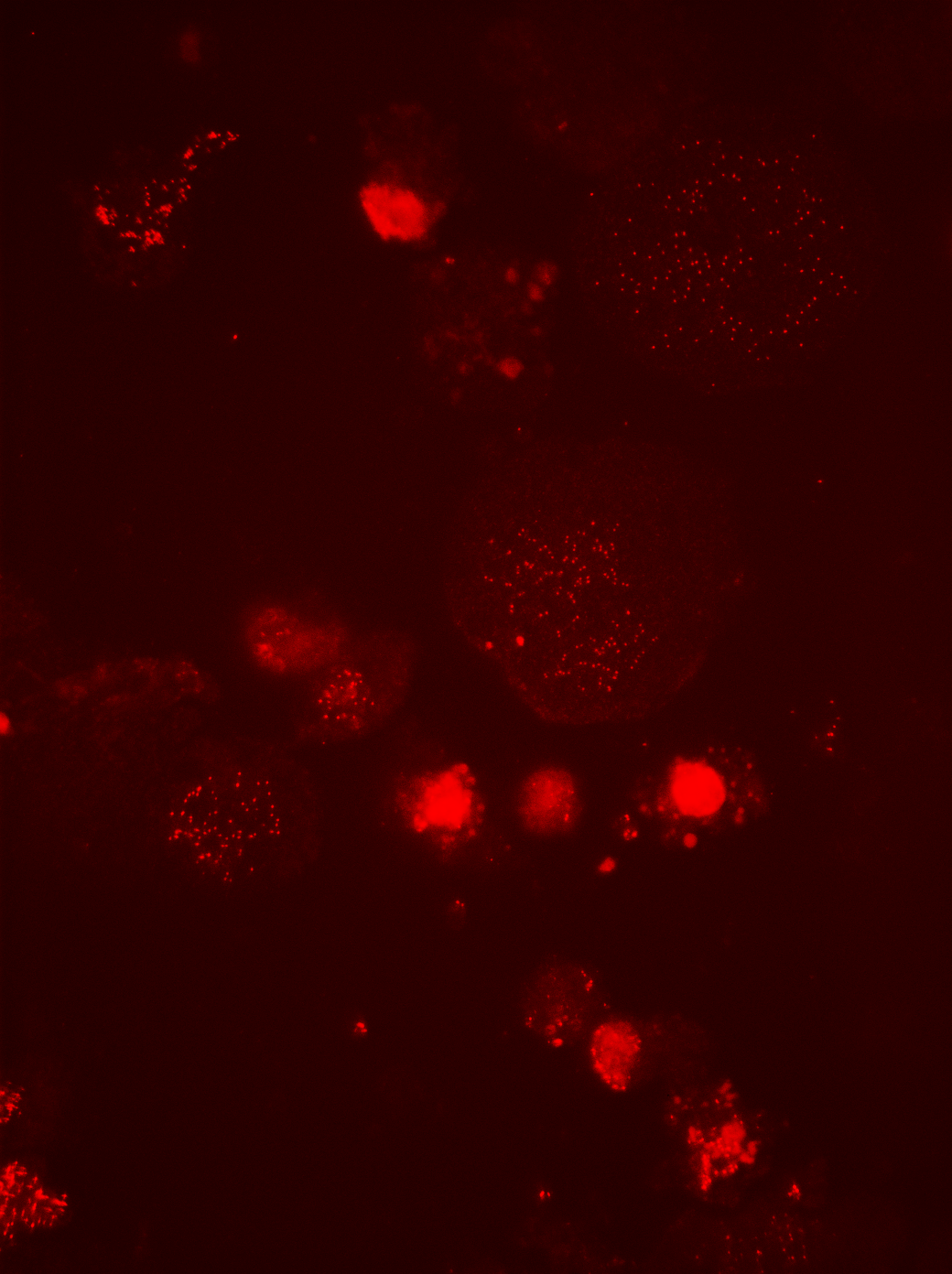

Supplement: Supplementary file 12 — Source data Fig. 6 [file 44318_2025_465_MOESM12_ESM.zip › EMBOJ-2025-120195-Figure 6-Source data/Figure 6/6H/CB-ATRX (1394-1443)-GFP-Y1419A-siATRX #1 CENP-A.tif]

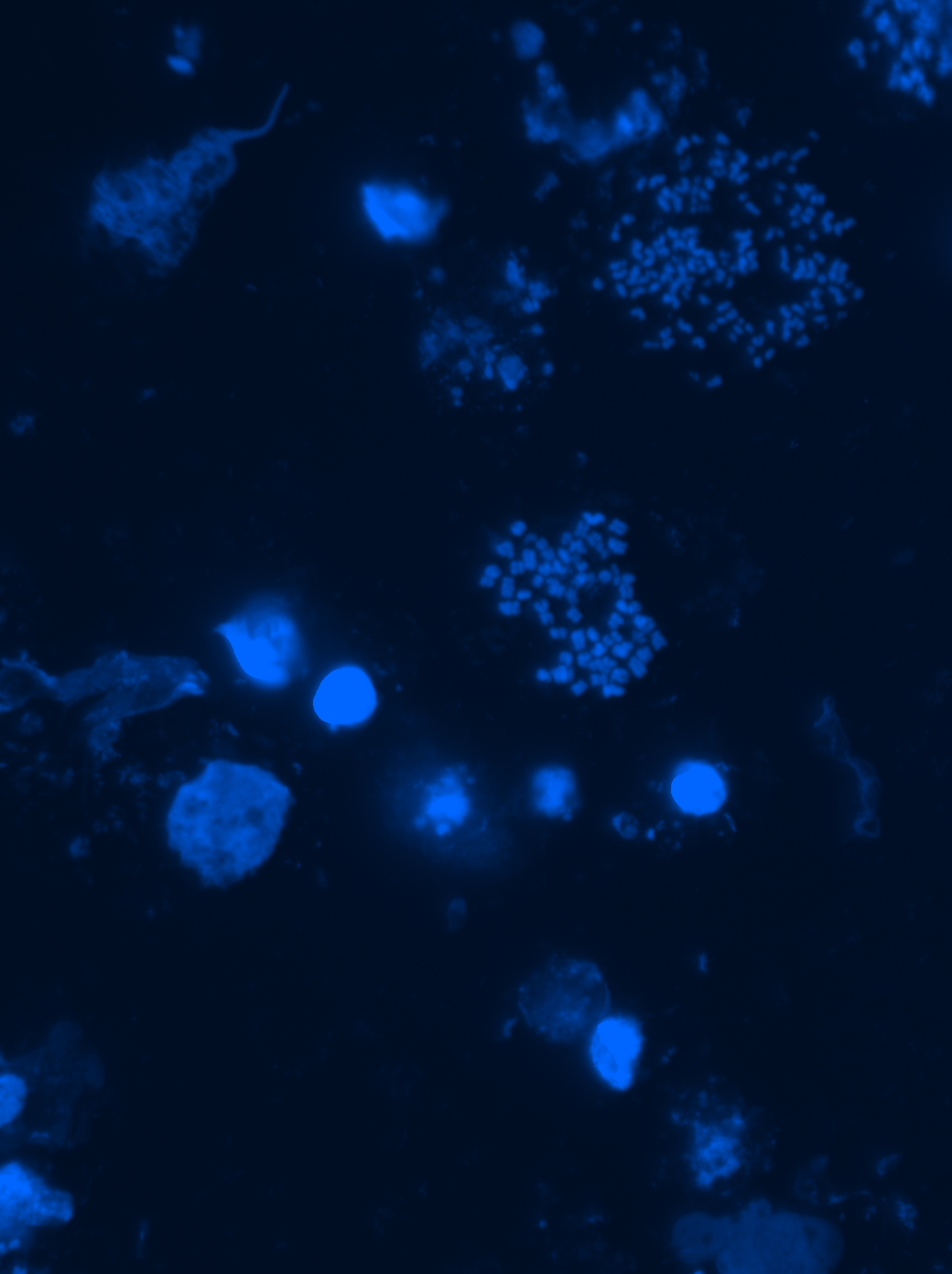

Supplement: Supplementary file 12 — Source data Fig. 6 [file 44318_2025_465_MOESM12_ESM.zip › EMBOJ-2025-120195-Figure 6-Source data/Figure 6/6H/CB-ATRX (1394-1443)-GFP-Y1419A-siATRX #1 DNA.tif]

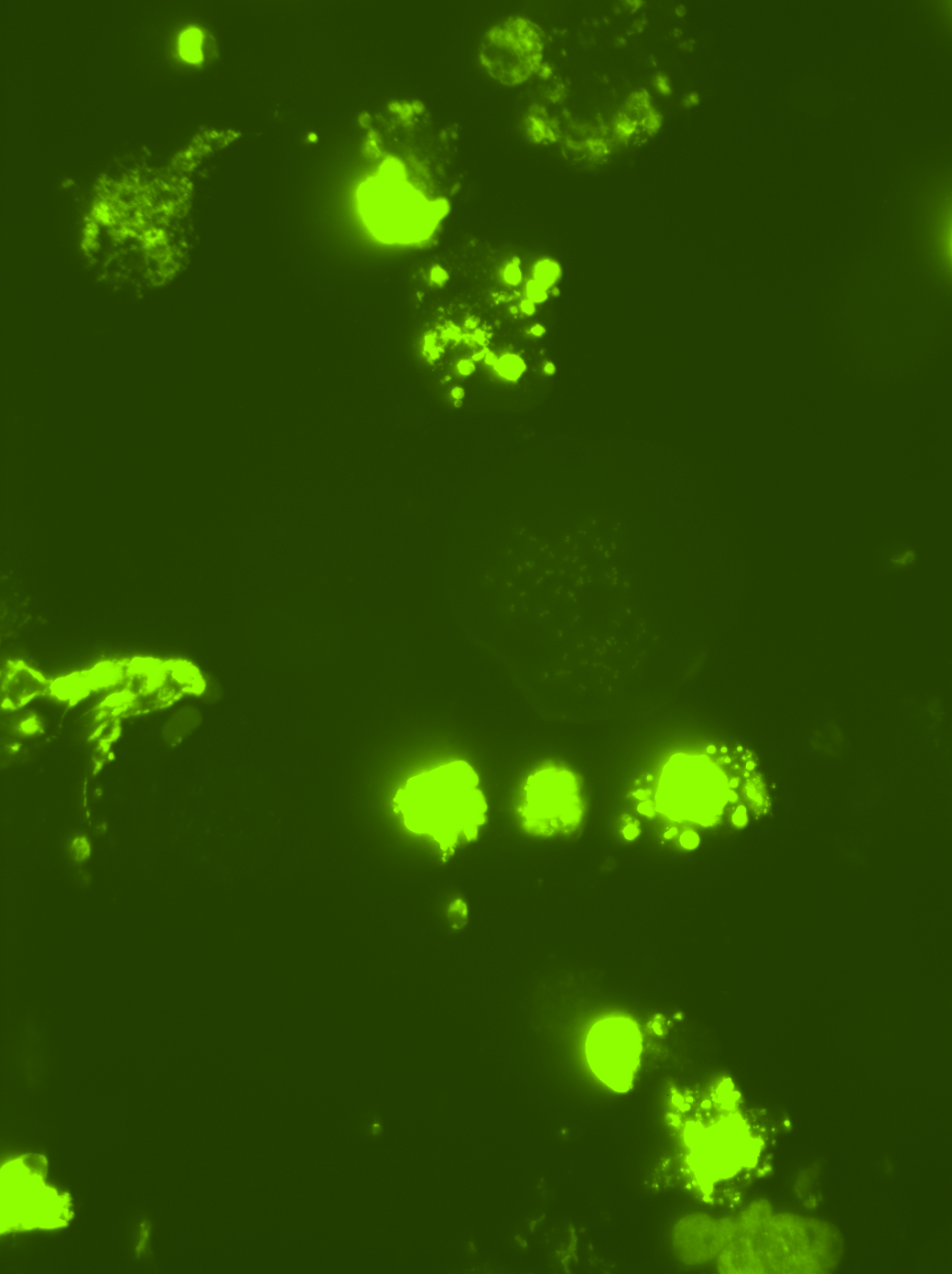

Supplement: Supplementary file 12 — Source data Fig. 6 [file 44318_2025_465_MOESM12_ESM.zip › EMBOJ-2025-120195-Figure 6-Source data/Figure 6/6H/CB-ATRX (1394-1443)-GFP-Y1419A-siATRX #1 GFP.tif]

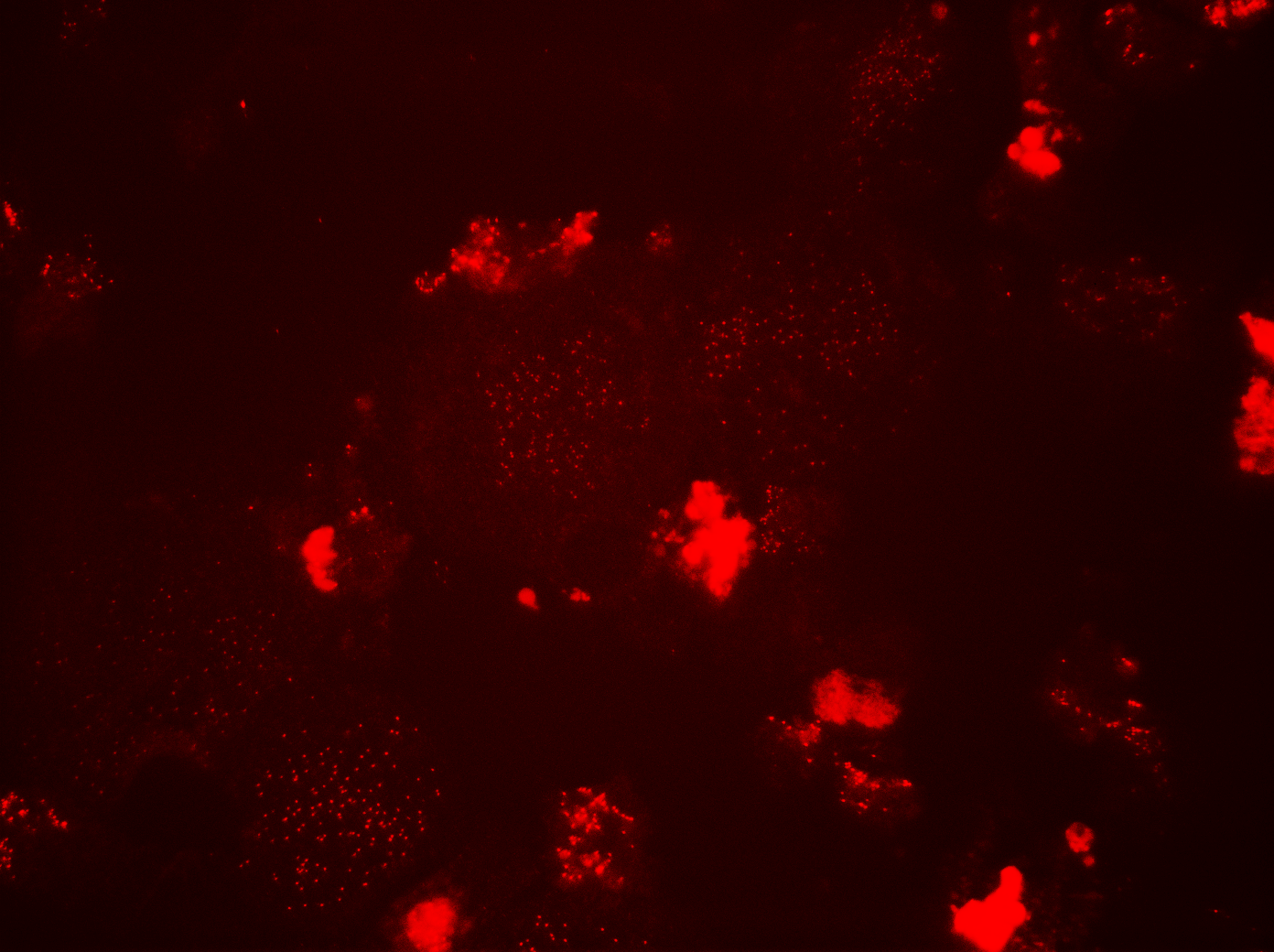

Supplement: Supplementary file 12 — Source data Fig. 6 [file 44318_2025_465_MOESM12_ESM.zip › EMBOJ-2025-120195-Figure 6-Source data/Figure 6/6H/CB-ATRX (1394-1443)-GFP-Y1419A-siControl CENP-A.tif]

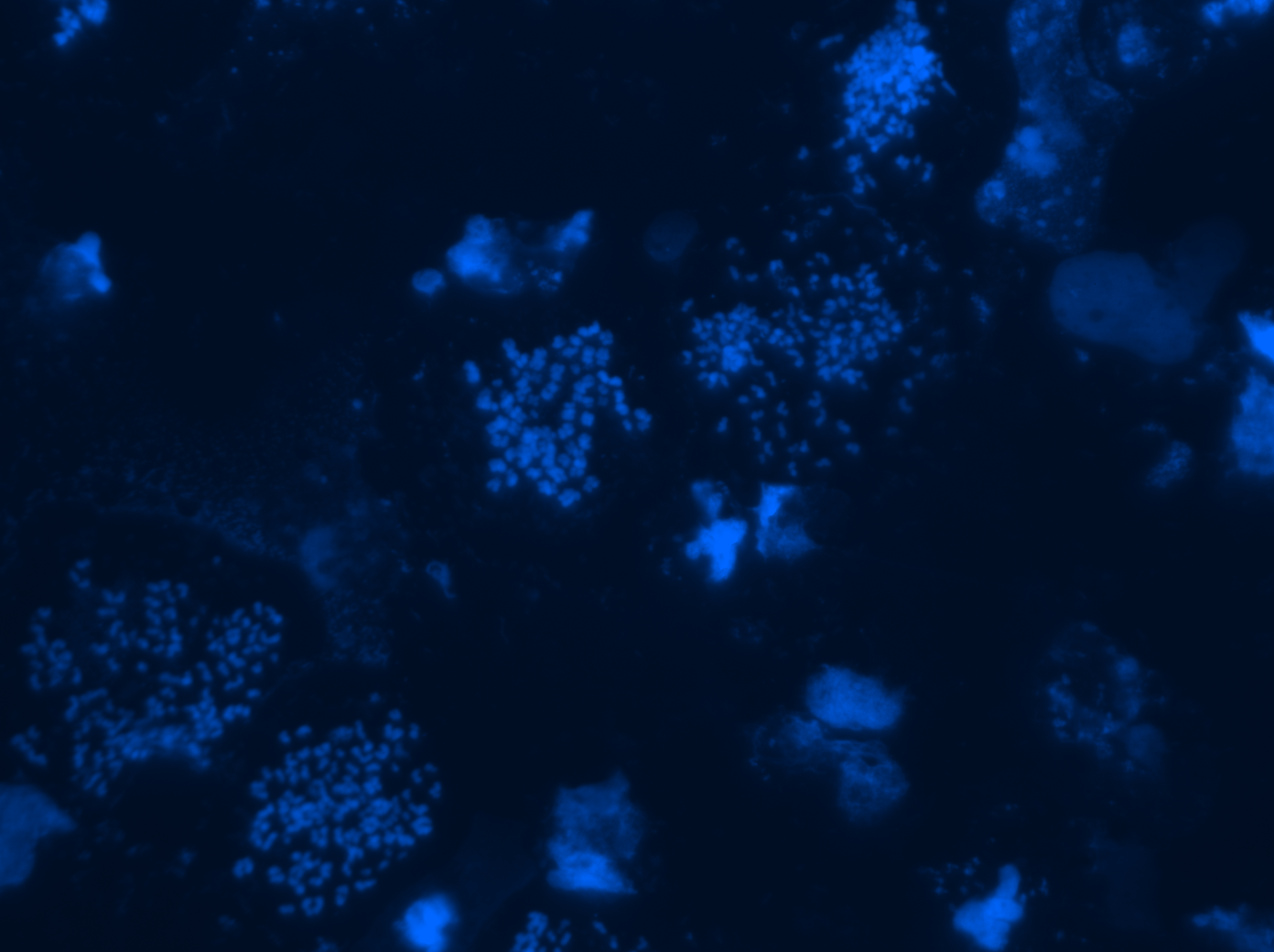

Supplement: Supplementary file 12 — Source data Fig. 6 [file 44318_2025_465_MOESM12_ESM.zip › EMBOJ-2025-120195-Figure 6-Source data/Figure 6/6H/CB-ATRX (1394-1443)-GFP-Y1419A-siControl DNA.tif]

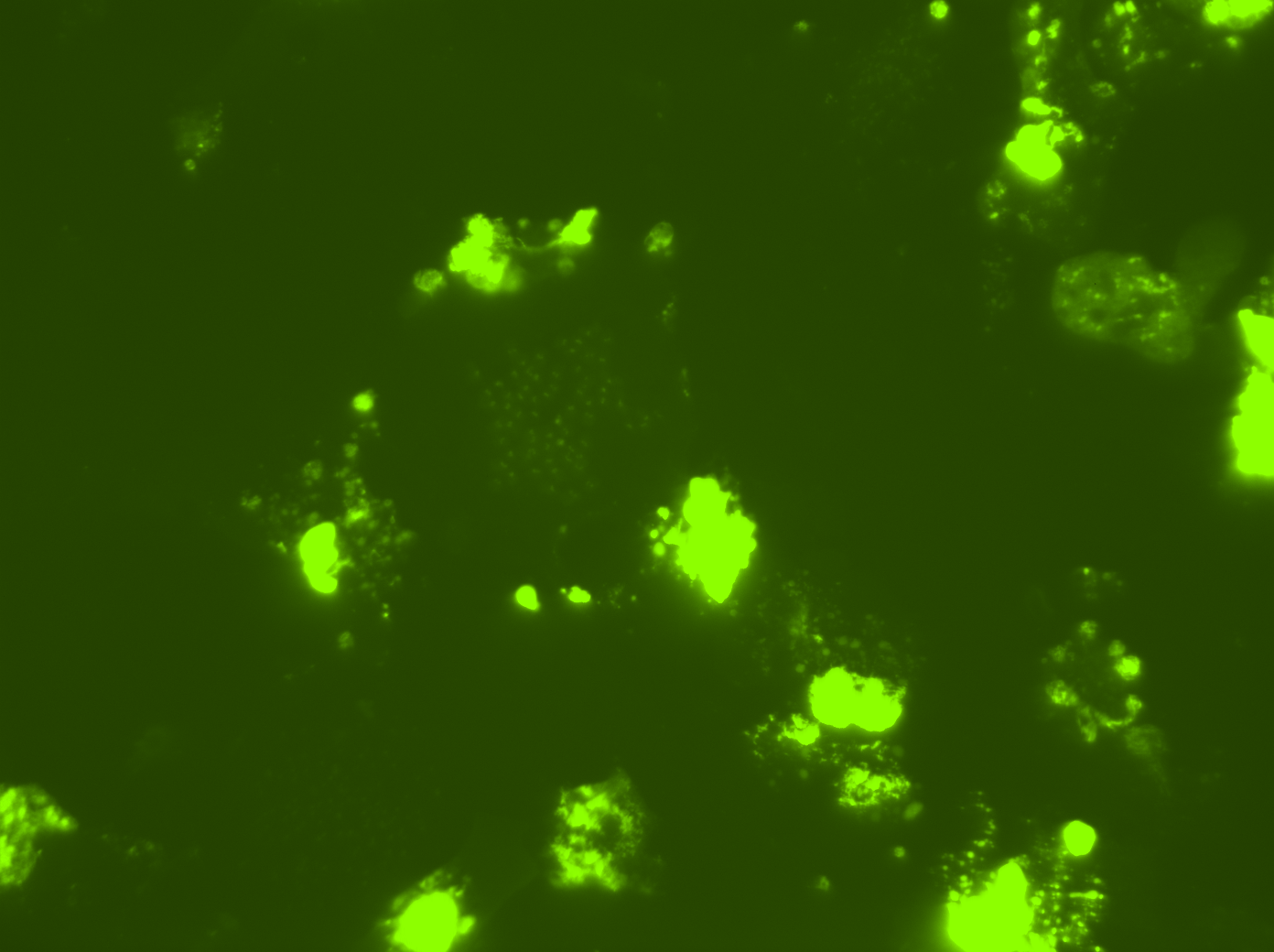

Supplement: Supplementary file 12 — Source data Fig. 6 [file 44318_2025_465_MOESM12_ESM.zip › EMBOJ-2025-120195-Figure 6-Source data/Figure 6/6H/CB-ATRX (1394-1443)-GFP-Y1419A-siControl GFP.tif]

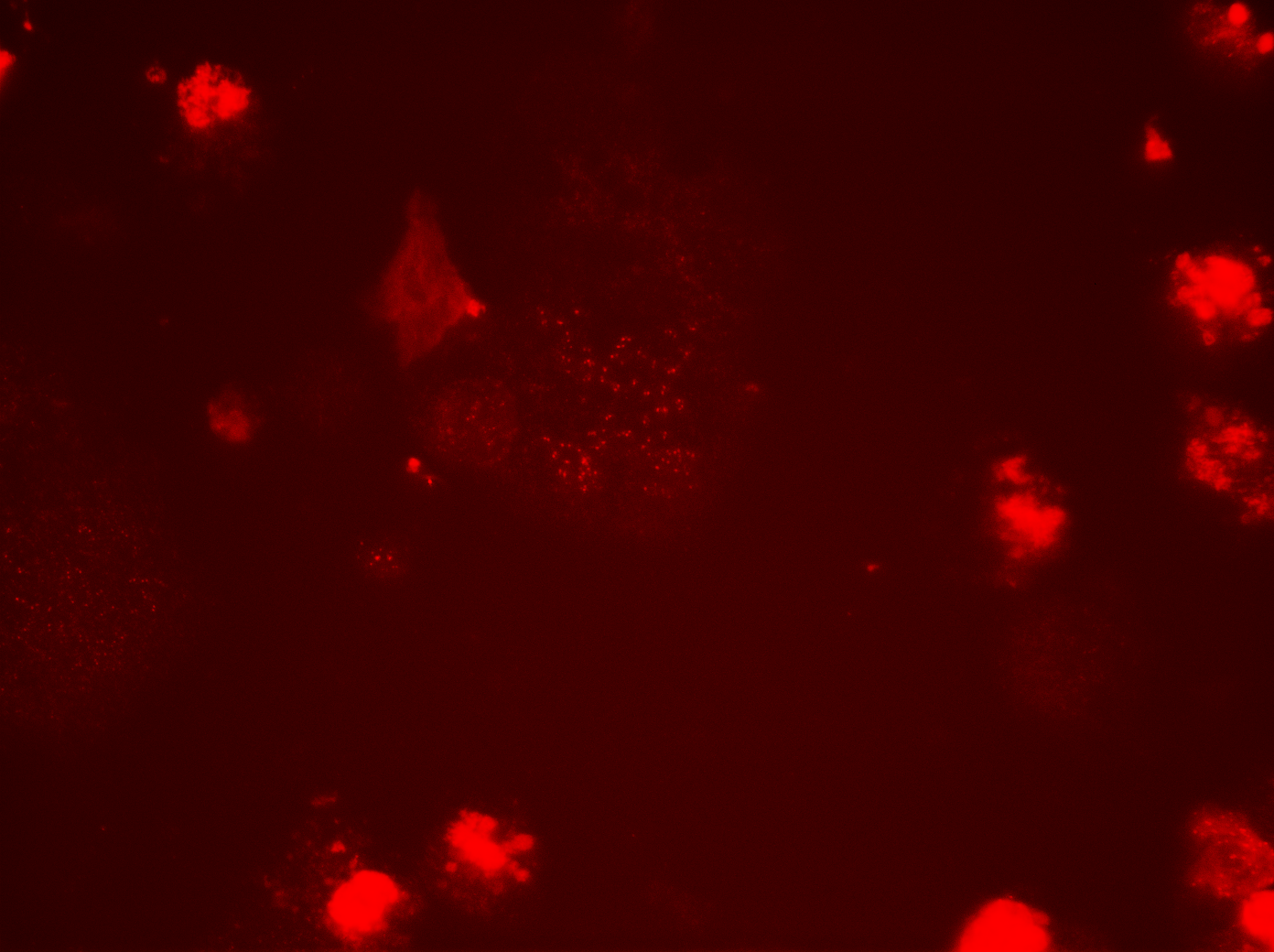

Supplement: Supplementary file 12 — Source data Fig. 6 [file 44318_2025_465_MOESM12_ESM.zip › EMBOJ-2025-120195-Figure 6-Source data/Figure 6/6H/CB-GFP-siATRX #1 CENP-A.tif]

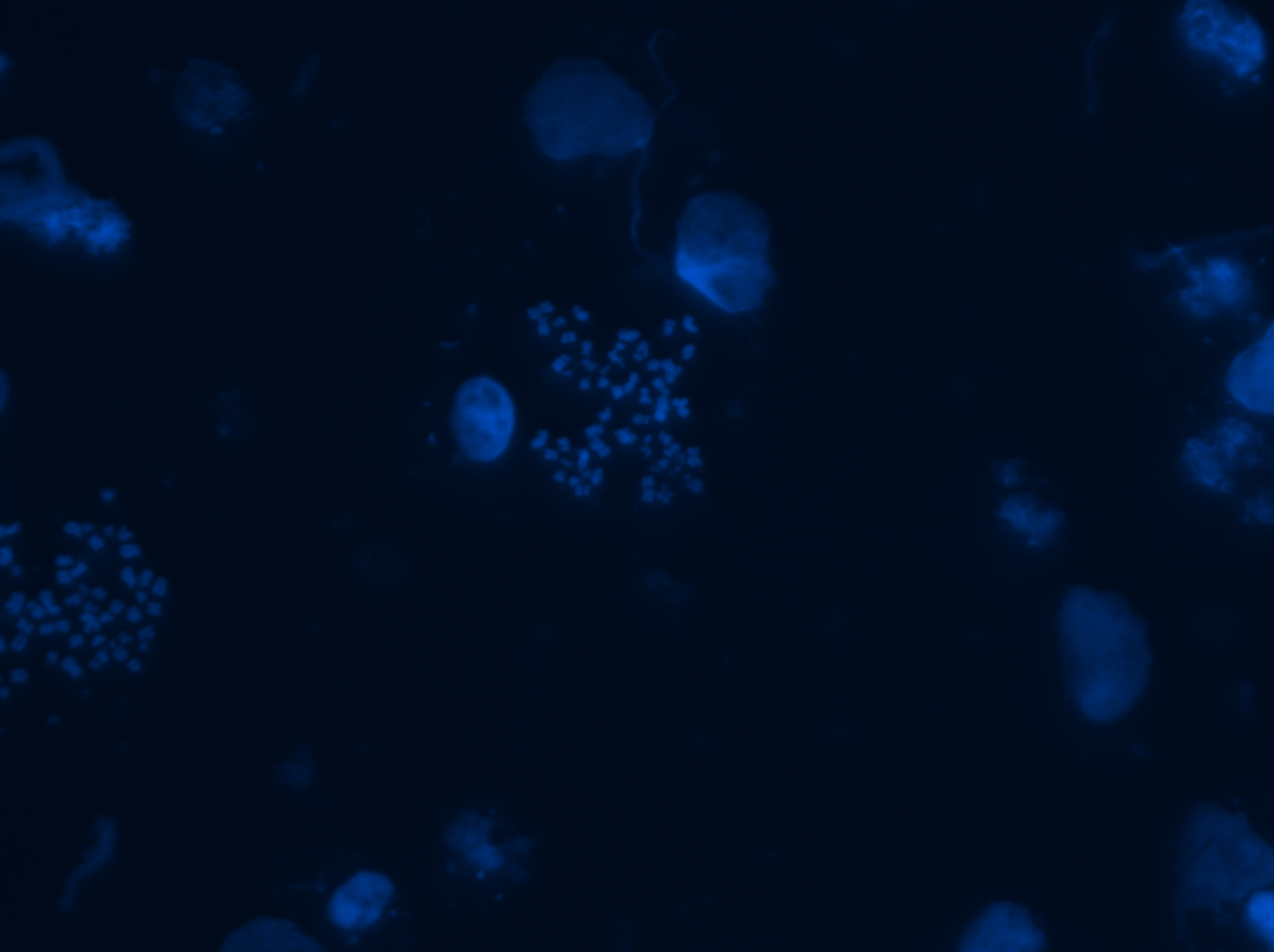

Supplement: Supplementary file 12 — Source data Fig. 6 [file 44318_2025_465_MOESM12_ESM.zip › EMBOJ-2025-120195-Figure 6-Source data/Figure 6/6H/CB-GFP-siATRX #1 DNA.tif]

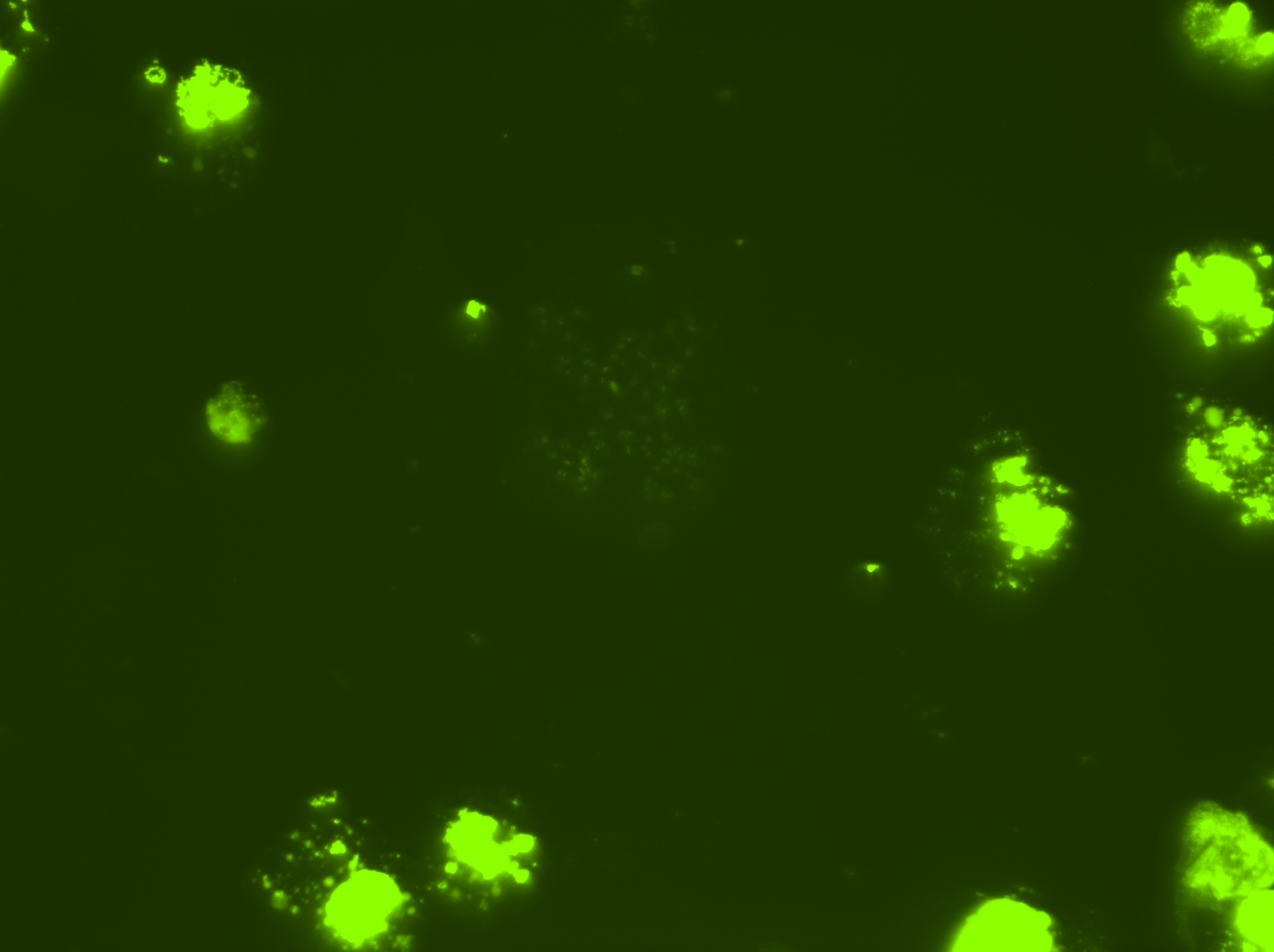

Supplement: Supplementary file 12 — Source data Fig. 6 [file 44318_2025_465_MOESM12_ESM.zip › EMBOJ-2025-120195-Figure 6-Source data/Figure 6/6H/CB-GFP-siATRX #1 GFP.tif]

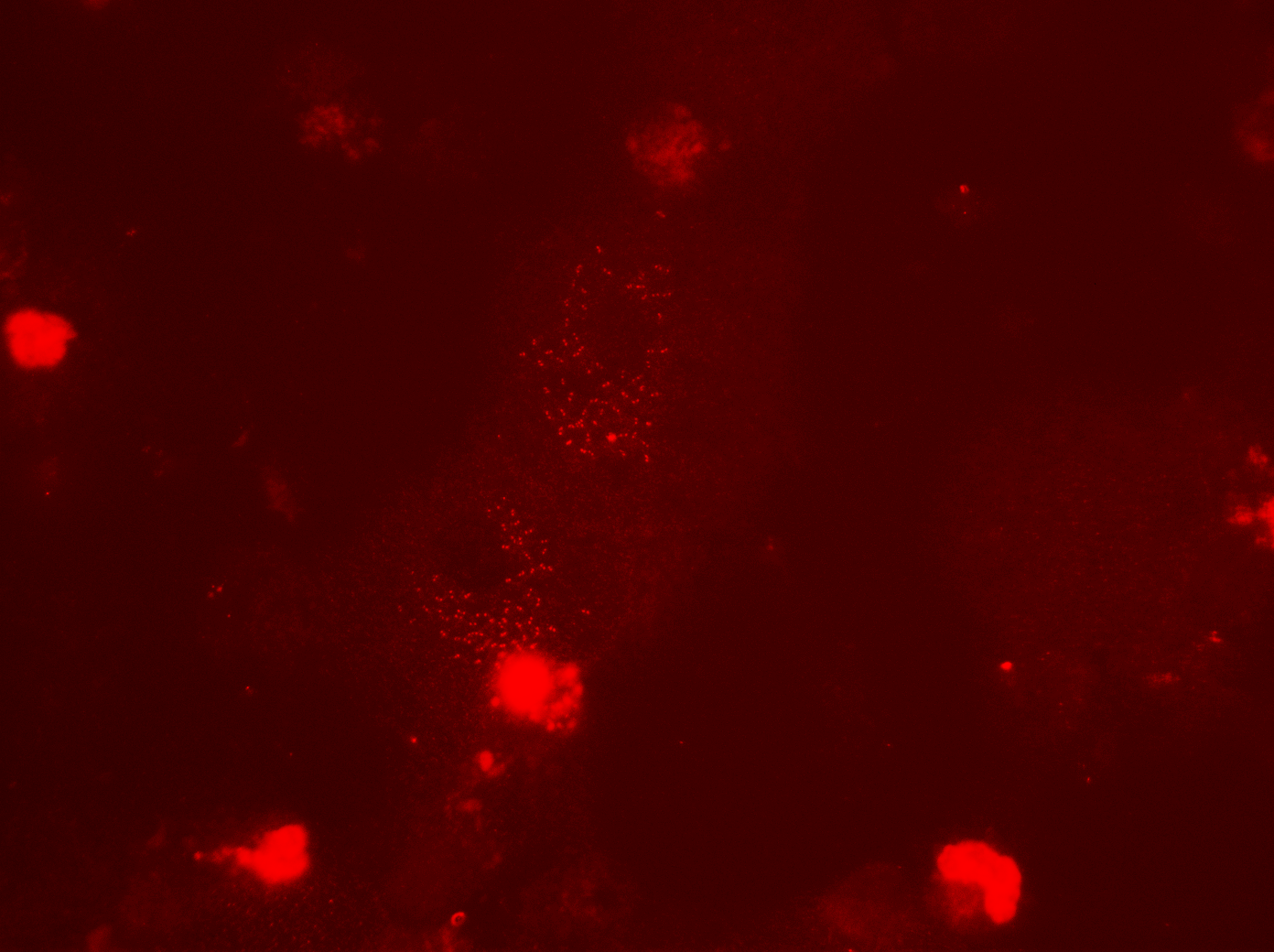

Supplement: Supplementary file 12 — Source data Fig. 6 [file 44318_2025_465_MOESM12_ESM.zip › EMBOJ-2025-120195-Figure 6-Source data/Figure 6/6H/CB-GFP-siControl CENP-A.tif]

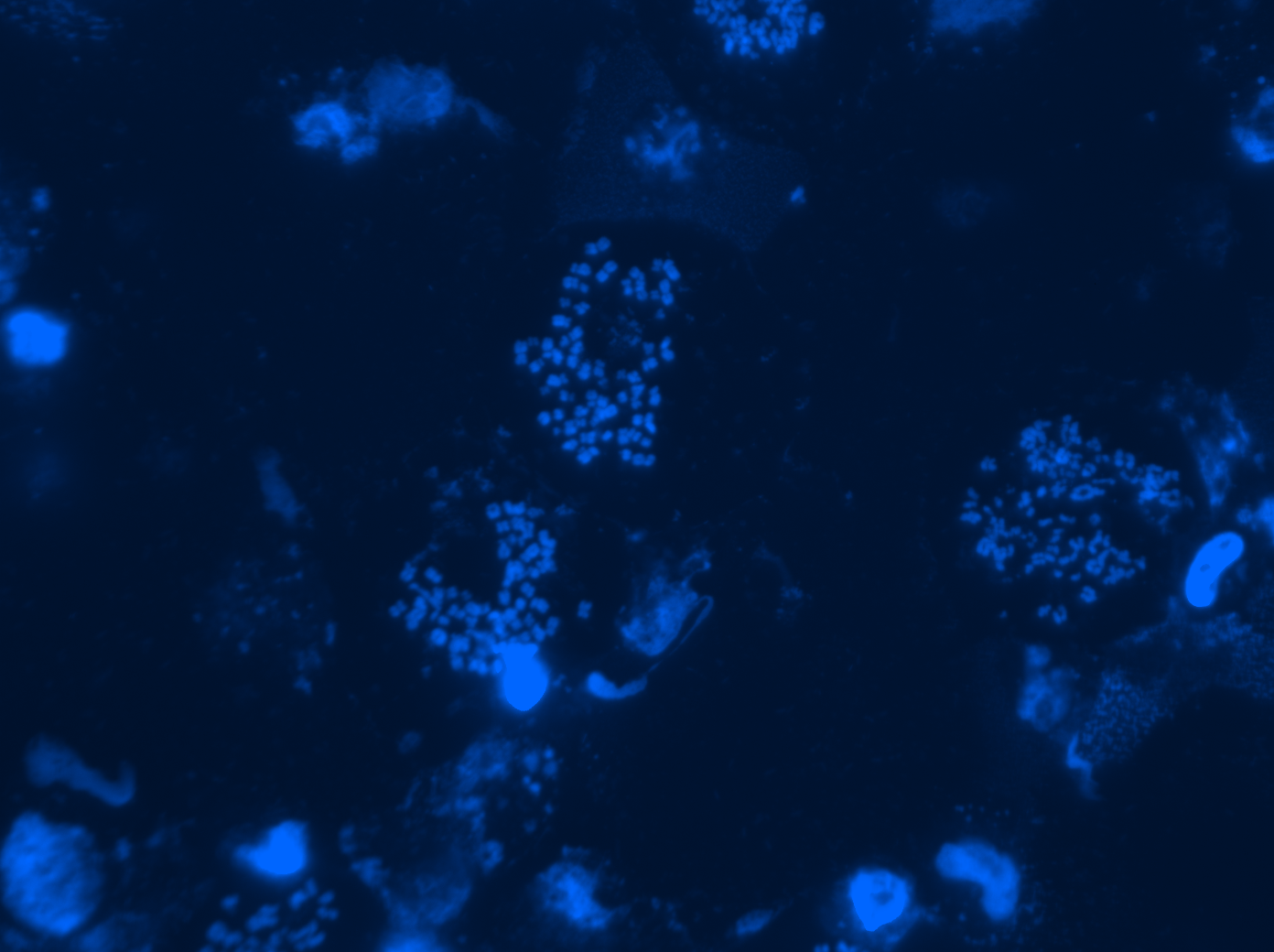

Supplement: Supplementary file 12 — Source data Fig. 6 [file 44318_2025_465_MOESM12_ESM.zip › EMBOJ-2025-120195-Figure 6-Source data/Figure 6/6H/CB-GFP-siControl DNA.tif]

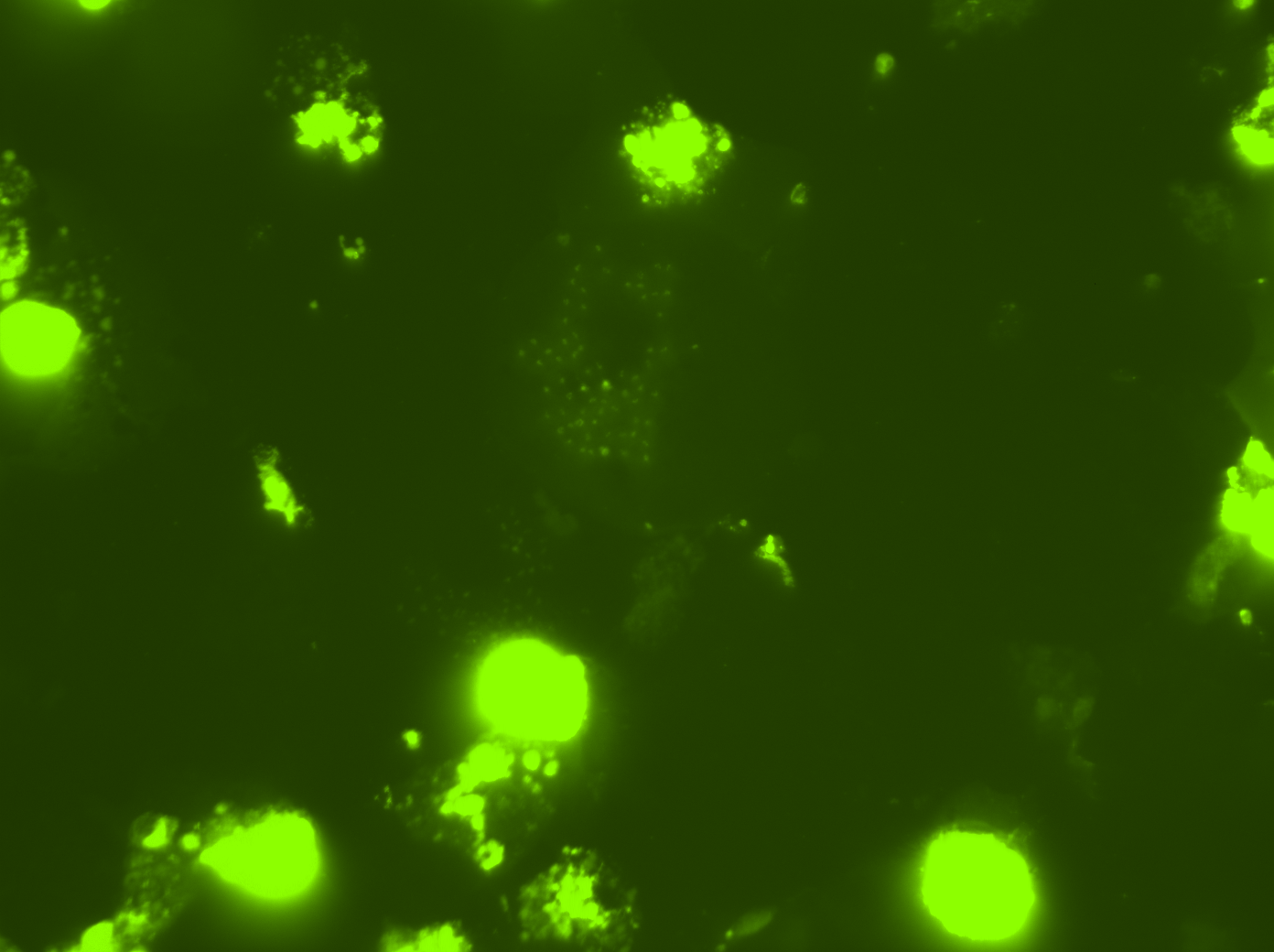

Supplement: Supplementary file 12 — Source data Fig. 6 [file 44318_2025_465_MOESM12_ESM.zip › EMBOJ-2025-120195-Figure 6-Source data/Figure 6/6H/CB-GFP-siControl GFP.tif]

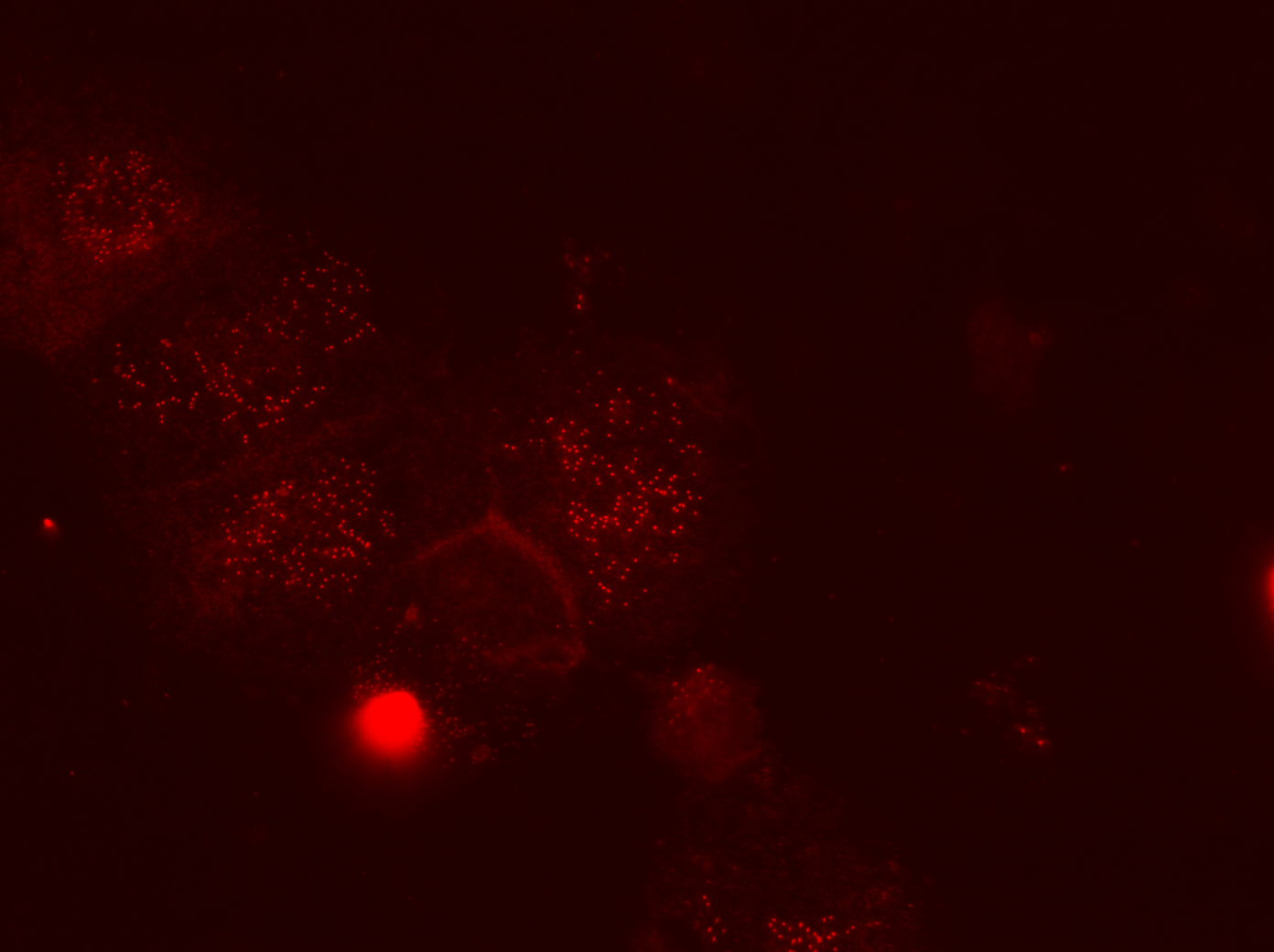

Supplement: Supplementary file 13 — Source data Fig. 7 [file 44318_2025_465_MOESM13_ESM.zip › EMBOJ-2025-120195-Figure 7-Source data/Figure 7/7B/HeLa-siATRX# 1 CENP-C.tif]

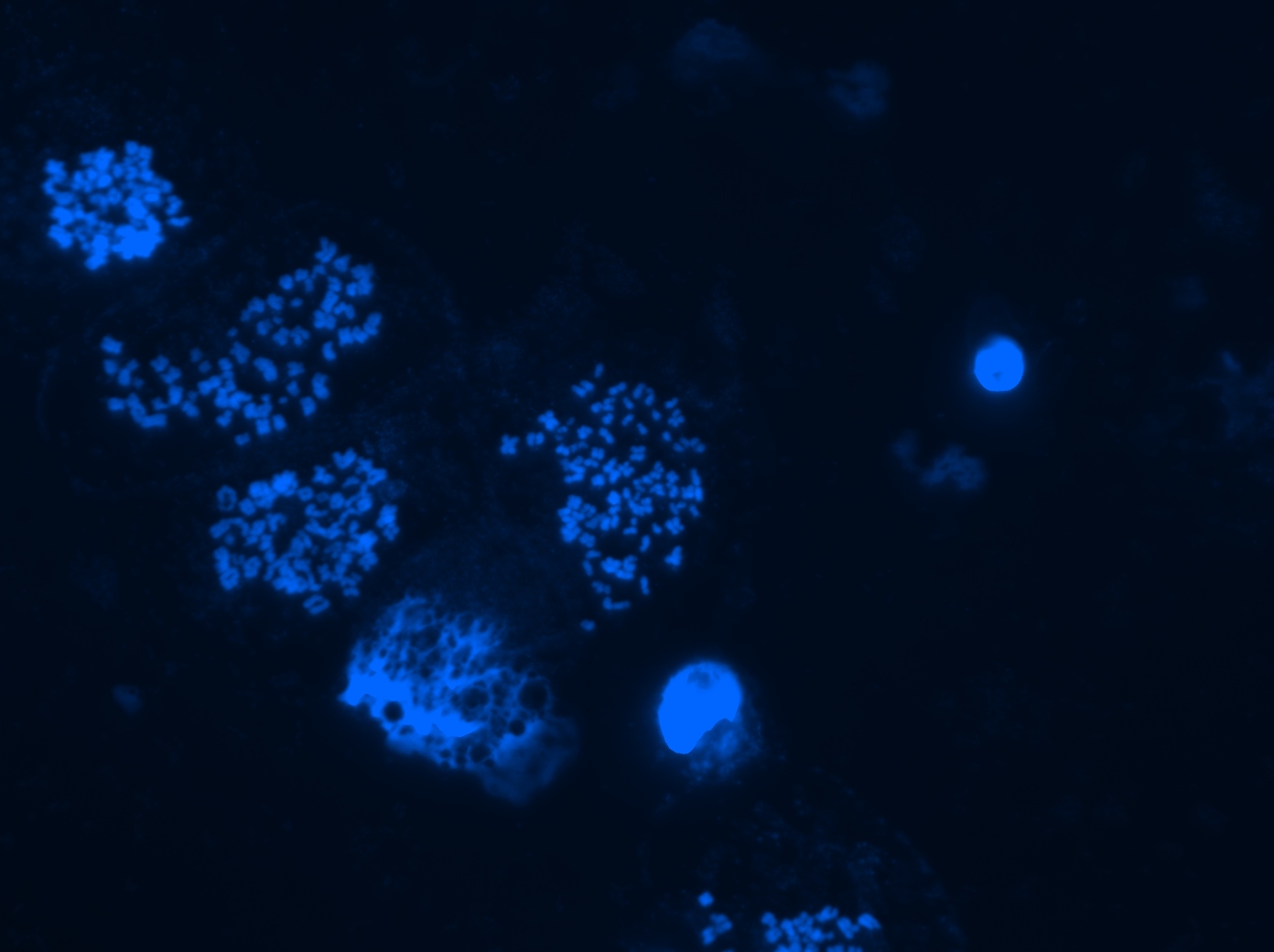

Supplement: Supplementary file 13 — Source data Fig. 7 [file 44318_2025_465_MOESM13_ESM.zip › EMBOJ-2025-120195-Figure 7-Source data/Figure 7/7B/HeLa-siATRX# 1 DNA.tif]

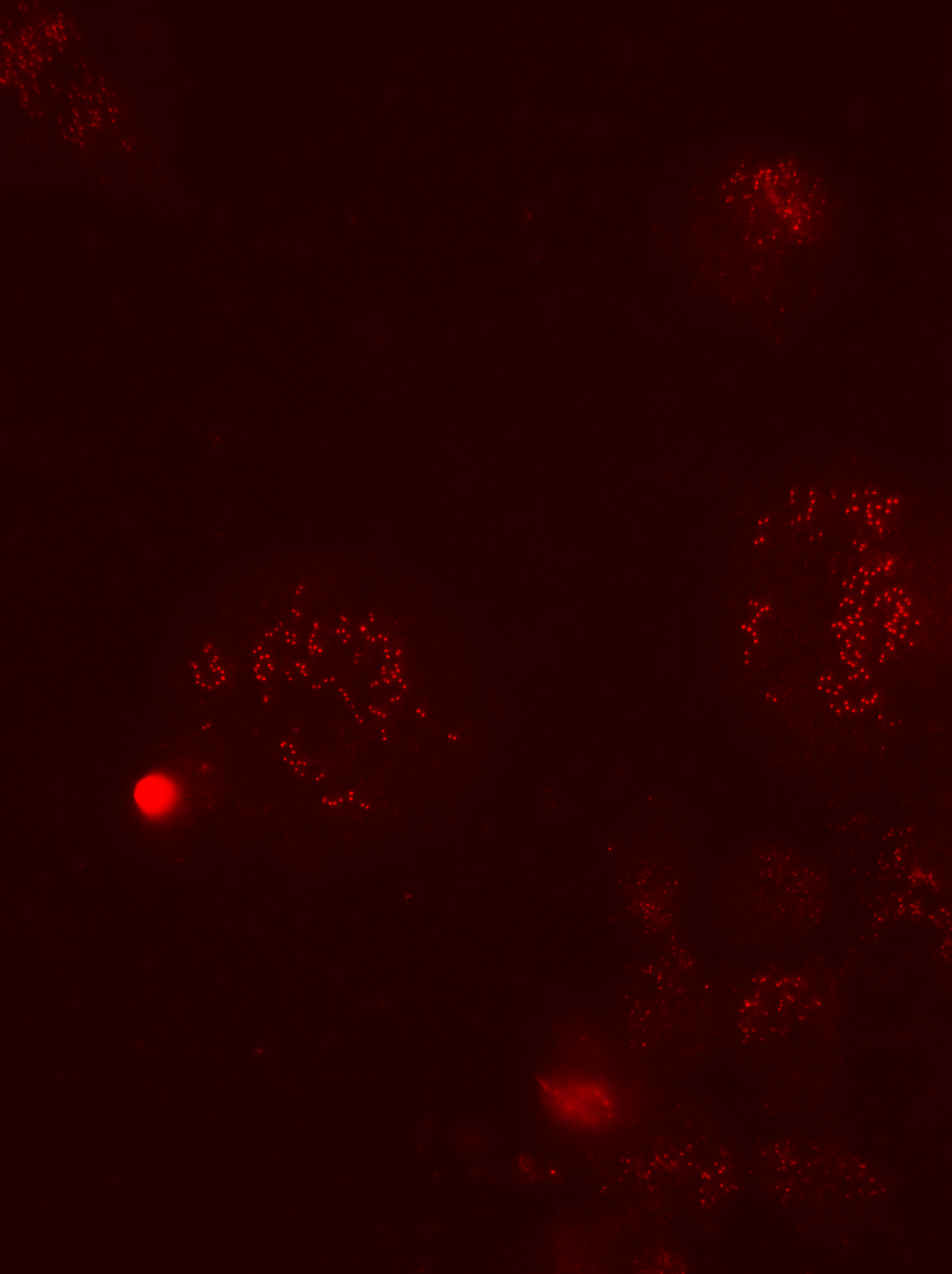

Supplement: Supplementary file 13 — Source data Fig. 7 [file 44318_2025_465_MOESM13_ESM.zip › EMBOJ-2025-120195-Figure 7-Source data/Figure 7/7B/Hela-siControl CENP-C.tif]

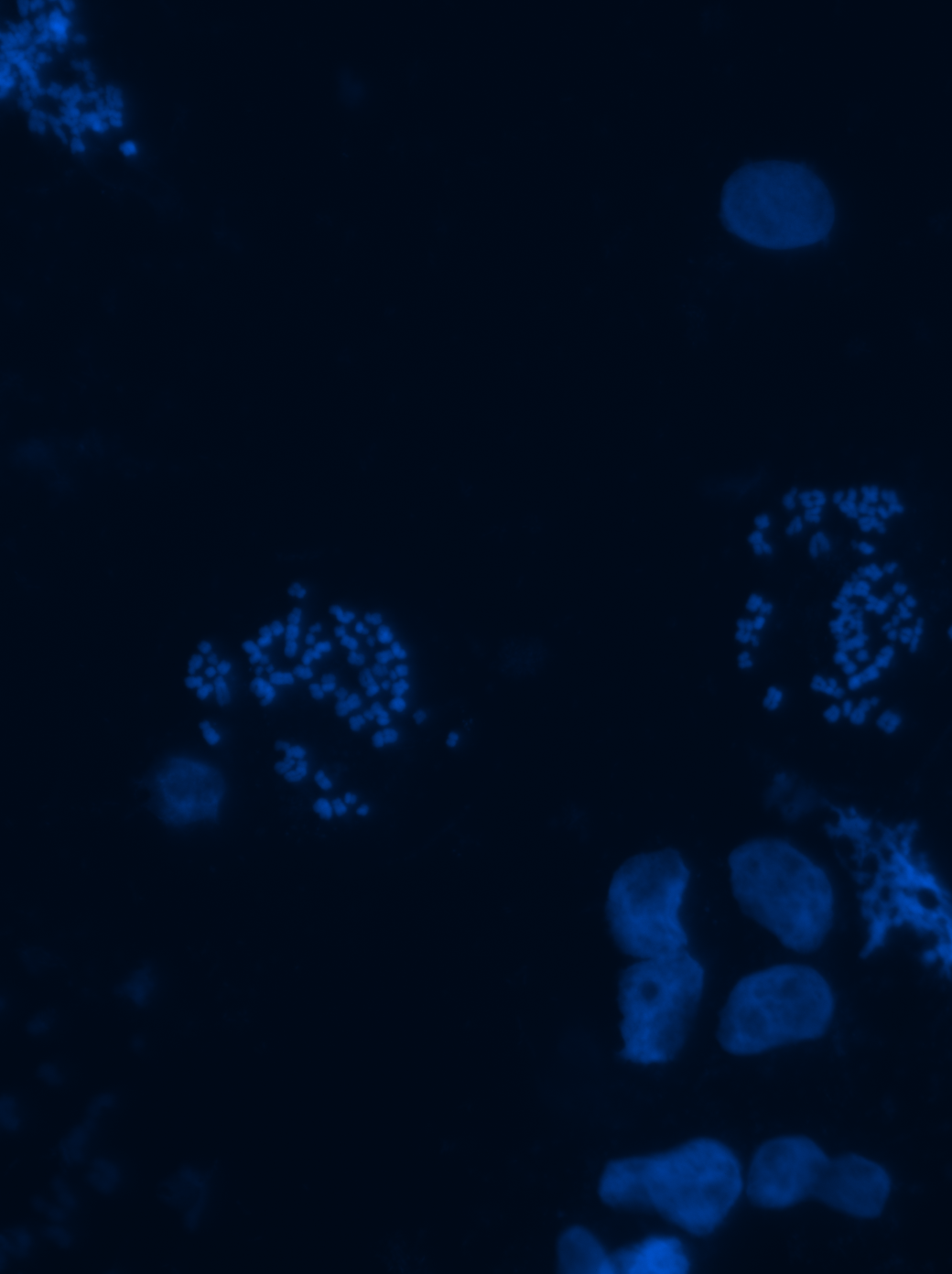

Supplement: Supplementary file 13 — Source data Fig. 7 [file 44318_2025_465_MOESM13_ESM.zip › EMBOJ-2025-120195-Figure 7-Source data/Figure 7/7B/Hela-siControl DNA.tif]

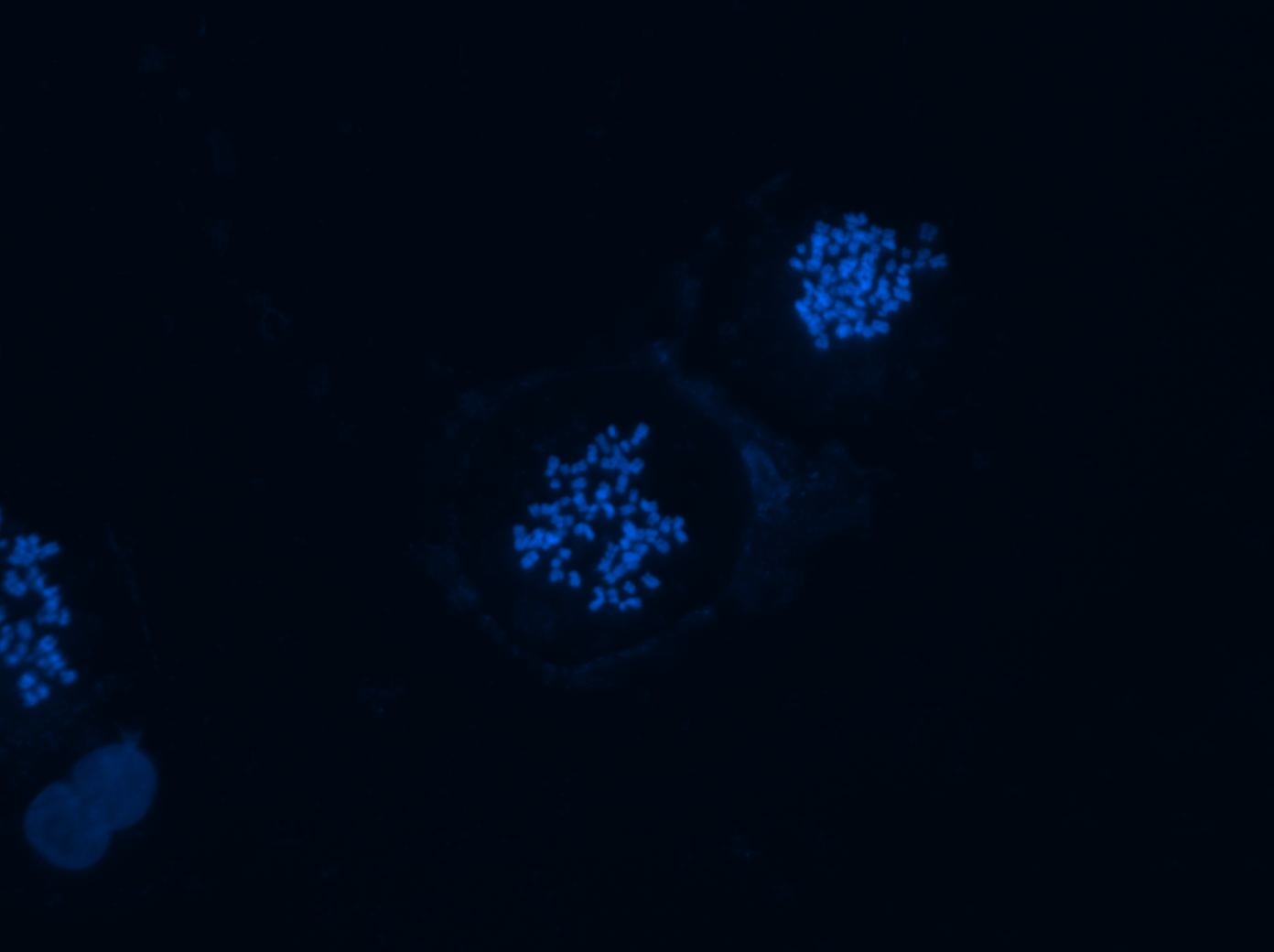

Supplement: Supplementary file 13 — Source data Fig. 7 [file 44318_2025_465_MOESM13_ESM.zip › EMBOJ-2025-120195-Figure 7-Source data/Figure 7/7B/HeLa-siWapl DNA.tif]

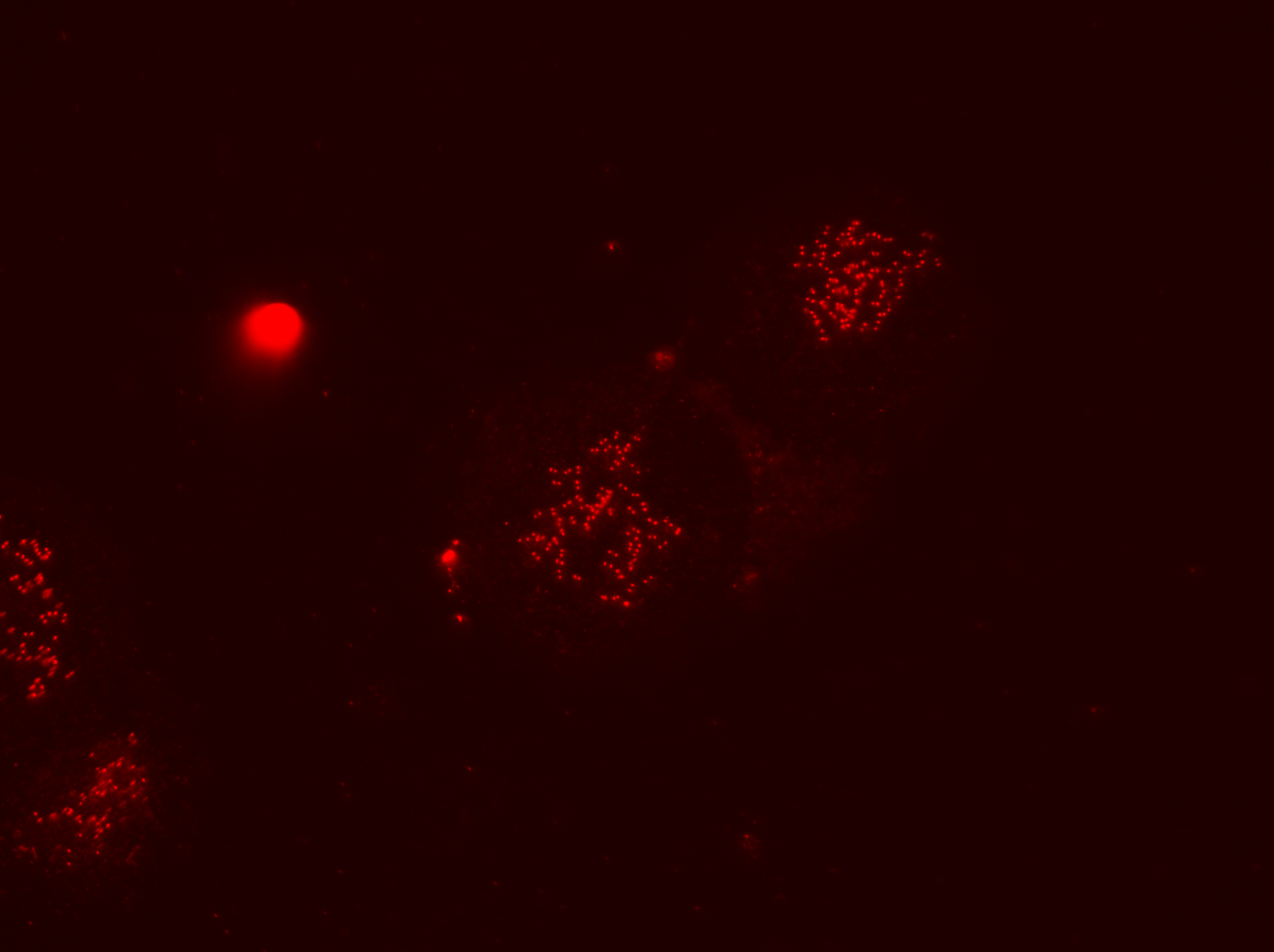

Supplement: Supplementary file 13 — Source data Fig. 7 [file 44318_2025_465_MOESM13_ESM.zip › EMBOJ-2025-120195-Figure 7-Source data/Figure 7/7B/HeLa-siWapl CENP-C.tif]

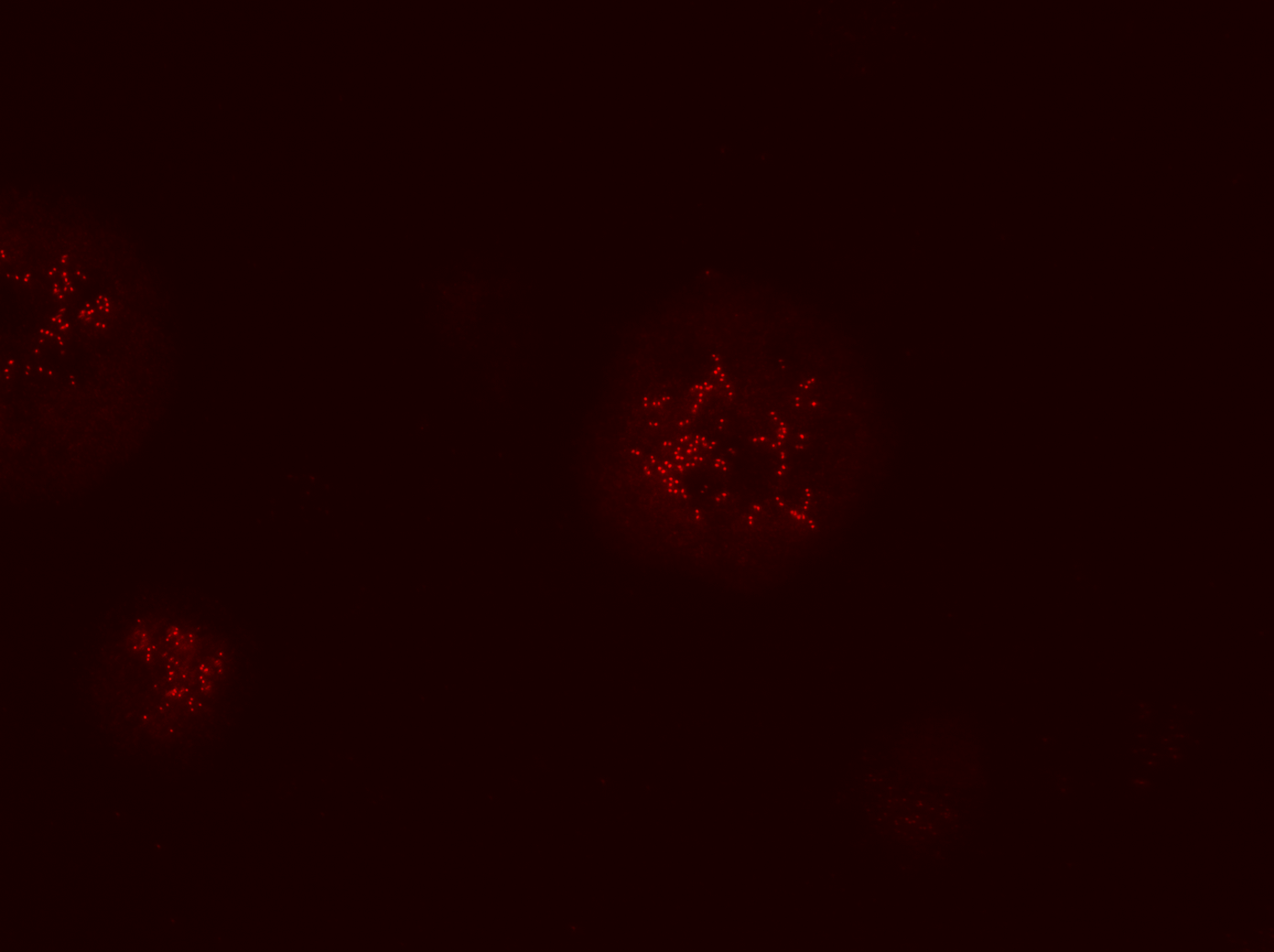

Supplement: Supplementary file 13 — Source data Fig. 7 [file 44318_2025_465_MOESM13_ESM.zip › EMBOJ-2025-120195-Figure 7-Source data/Figure 7/7B/HeLa-siWapl+siATRX #1 CENP-C.tif]

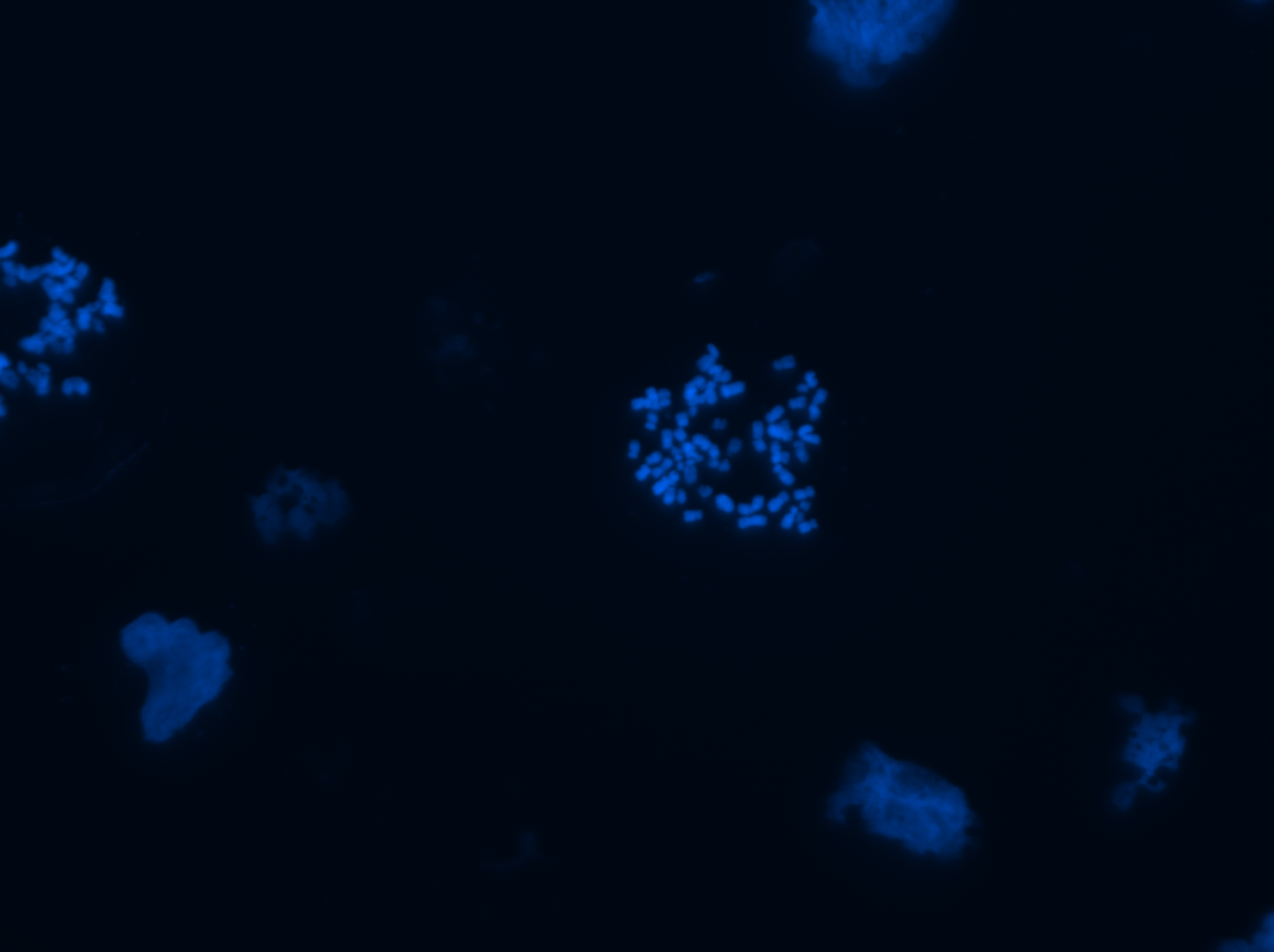

Supplement: Supplementary file 13 — Source data Fig. 7 [file 44318_2025_465_MOESM13_ESM.zip › EMBOJ-2025-120195-Figure 7-Source data/Figure 7/7B/HeLa-siWapl+siATRX #1 DNA.tif]

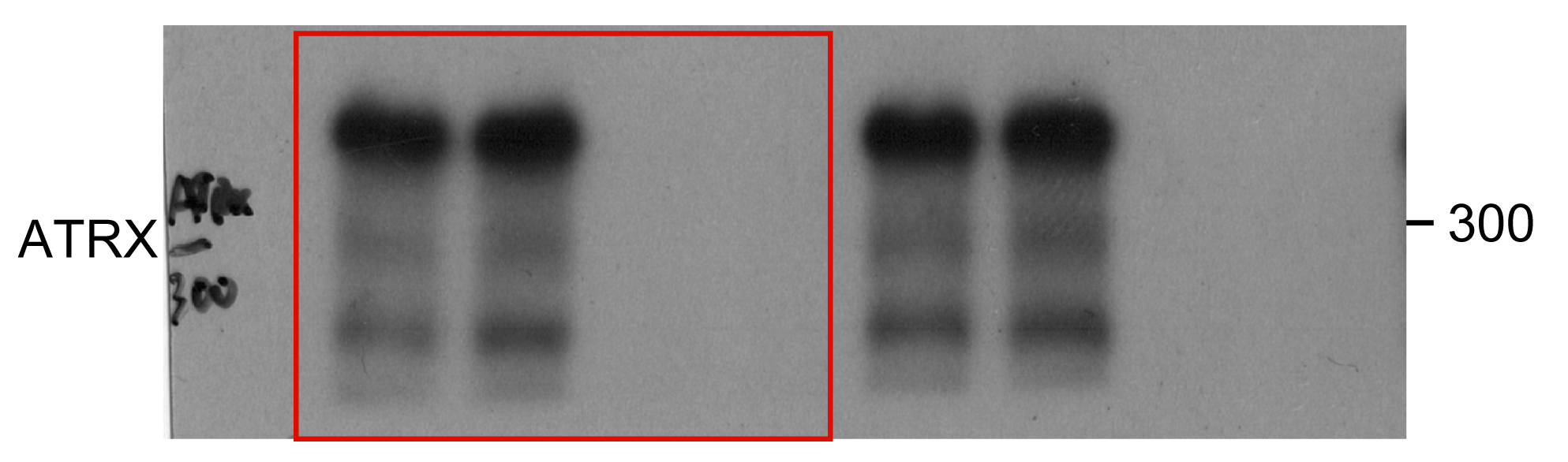

Supplement: Supplementary file 13 — Source data Fig. 7 [file 44318_2025_465_MOESM13_ESM.zip › EMBOJ-2025-120195-Figure 7-Source data/Figure 7/7C/western ATRX.tif]

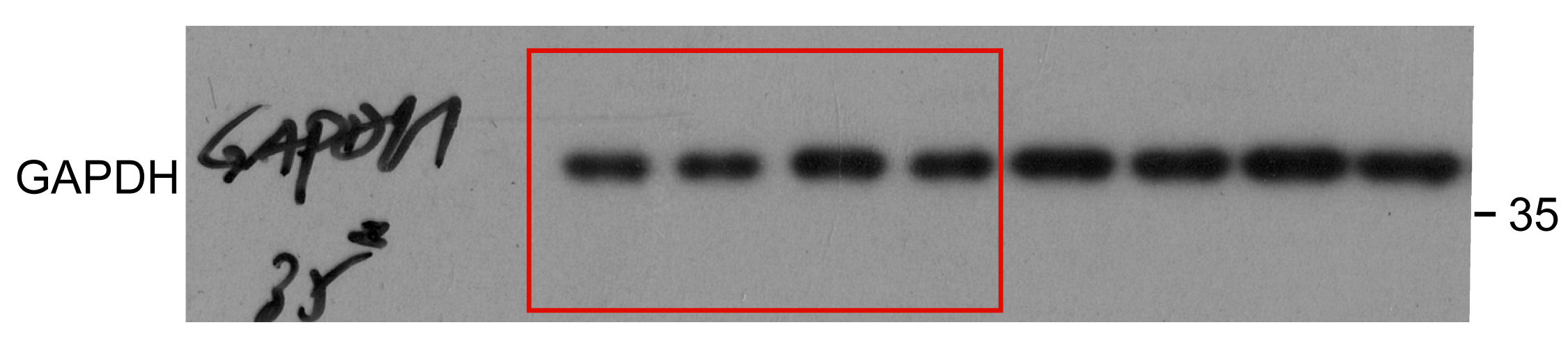

Supplement: Supplementary file 13 — Source data Fig. 7 [file 44318_2025_465_MOESM13_ESM.zip › EMBOJ-2025-120195-Figure 7-Source data/Figure 7/7C/western GAPDH.tif]

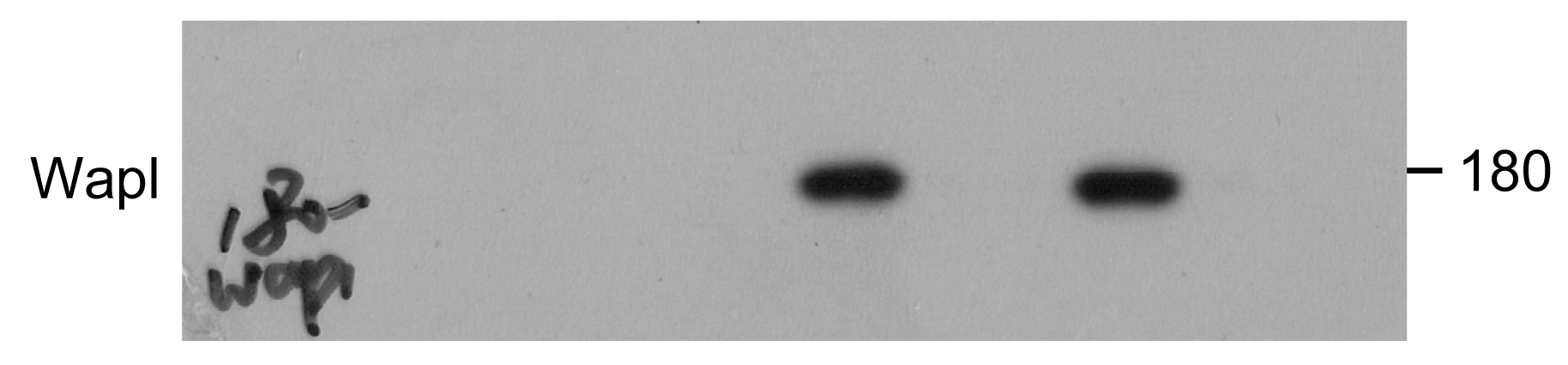

Supplement: Supplementary file 13 — Source data Fig. 7 [file 44318_2025_465_MOESM13_ESM.zip › EMBOJ-2025-120195-Figure 7-Source data/Figure 7/7C/western Wapl.tif]

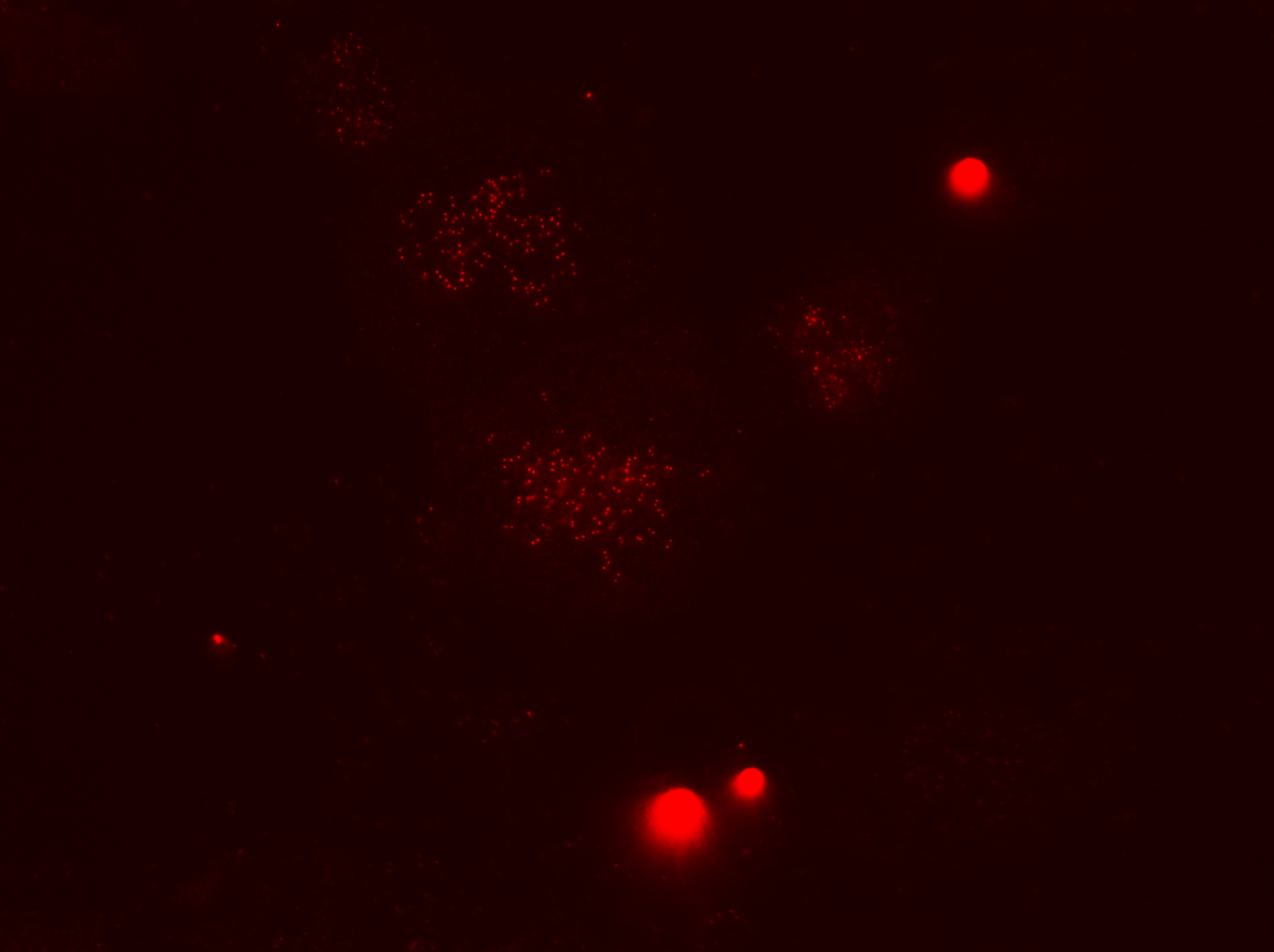

Supplement: Supplementary file 13 — Source data Fig. 7 [file 44318_2025_465_MOESM13_ESM.zip › EMBOJ-2025-120195-Figure 7-Source data/Figure 7/7G/HeLa-siATRX#1 CENP-C.tif]

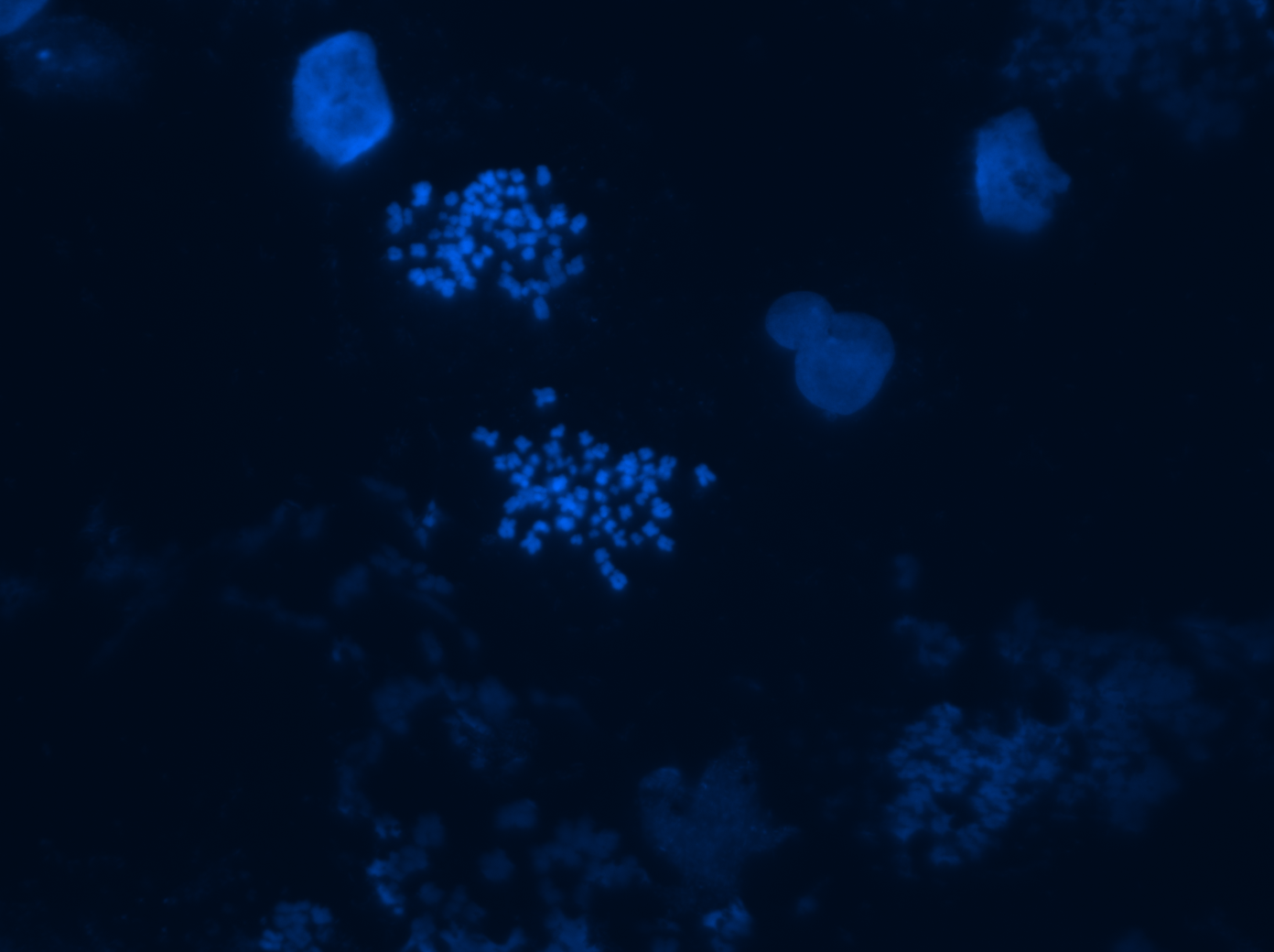

Supplement: Supplementary file 13 — Source data Fig. 7 [file 44318_2025_465_MOESM13_ESM.zip › EMBOJ-2025-120195-Figure 7-Source data/Figure 7/7G/HeLa-siATRX#1 DNA.tif]

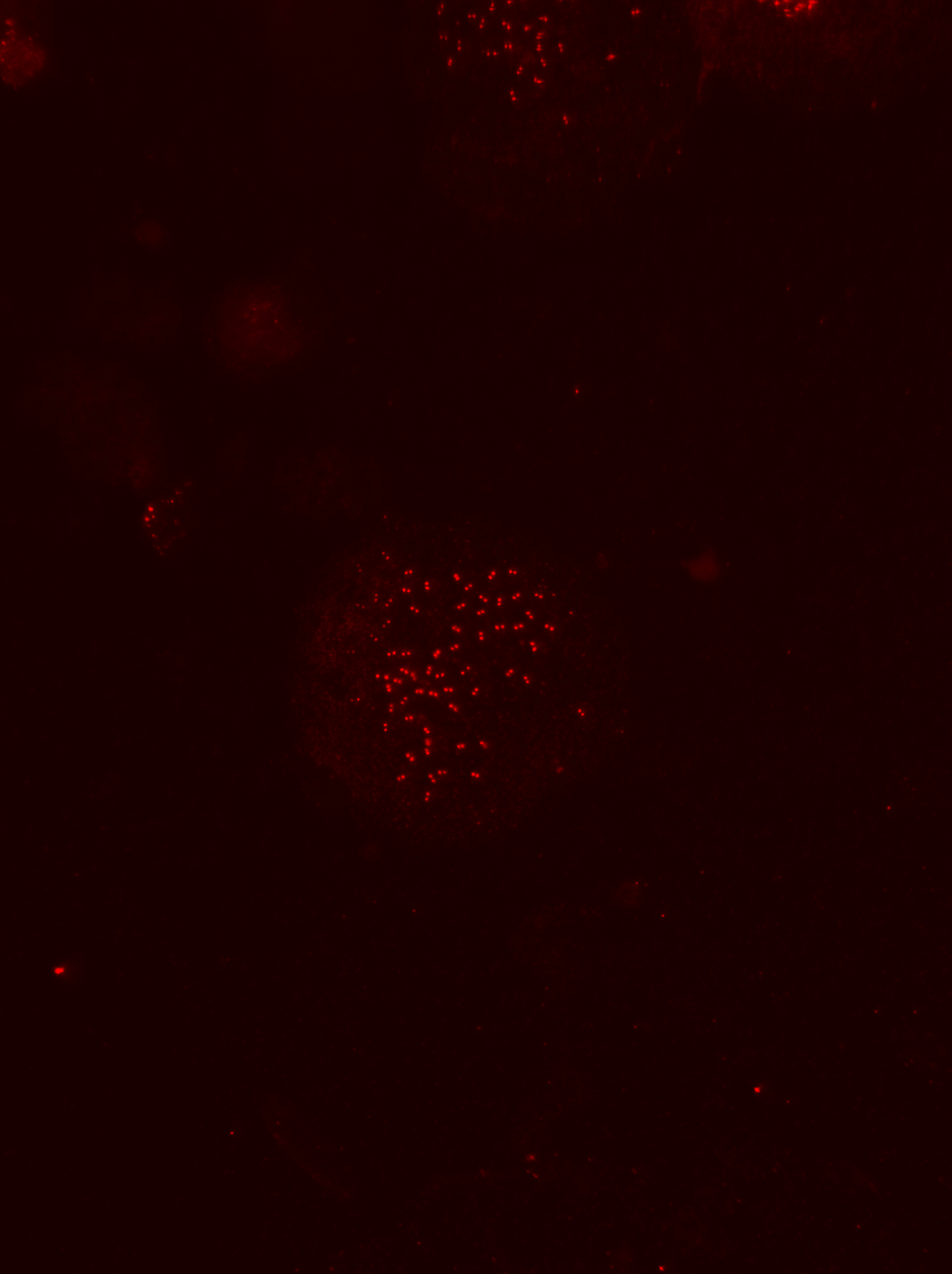

Supplement: Supplementary file 13 — Source data Fig. 7 [file 44318_2025_465_MOESM13_ESM.zip › EMBOJ-2025-120195-Figure 7-Source data/Figure 7/7G/HeLa-siControl CENP-C.tif]

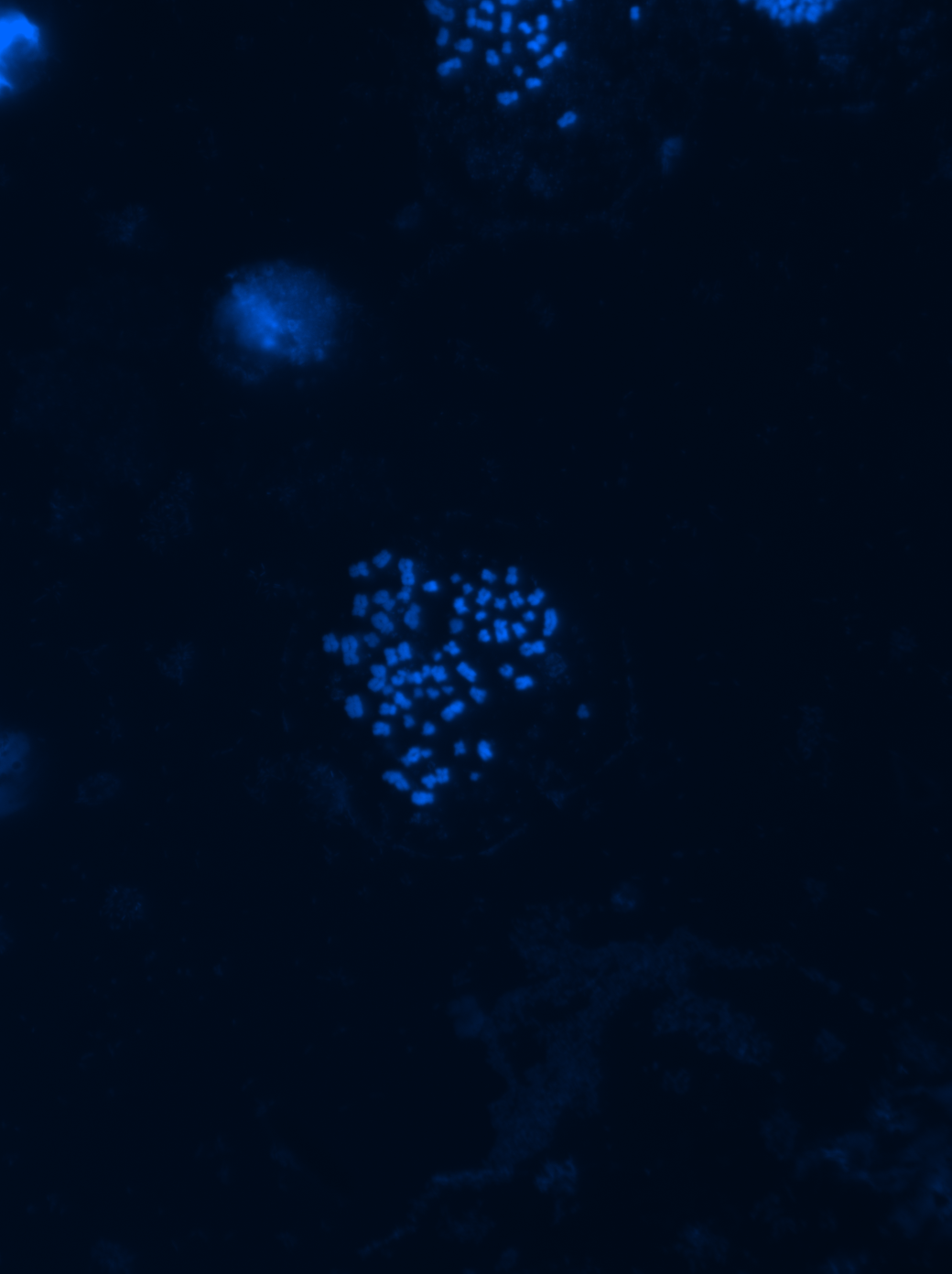

Supplement: Supplementary file 13 — Source data Fig. 7 [file 44318_2025_465_MOESM13_ESM.zip › EMBOJ-2025-120195-Figure 7-Source data/Figure 7/7G/HeLa-siControl DNA.tif]

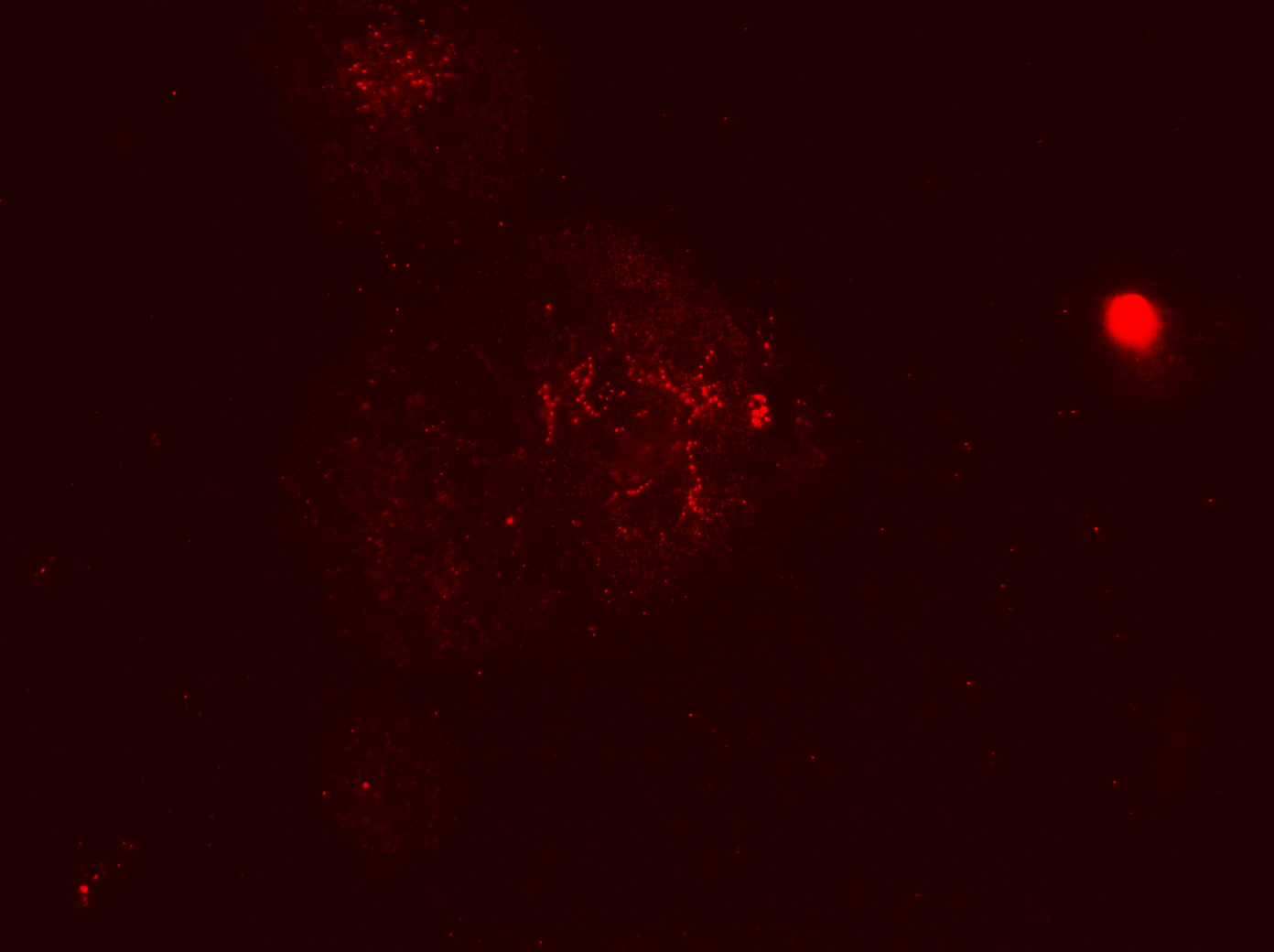

Supplement: Supplementary file 13 — Source data Fig. 7 [file 44318_2025_465_MOESM13_ESM.zip › EMBOJ-2025-120195-Figure 7-Source data/Figure 7/7G/HeLa-siWapl CENP-C.tif]

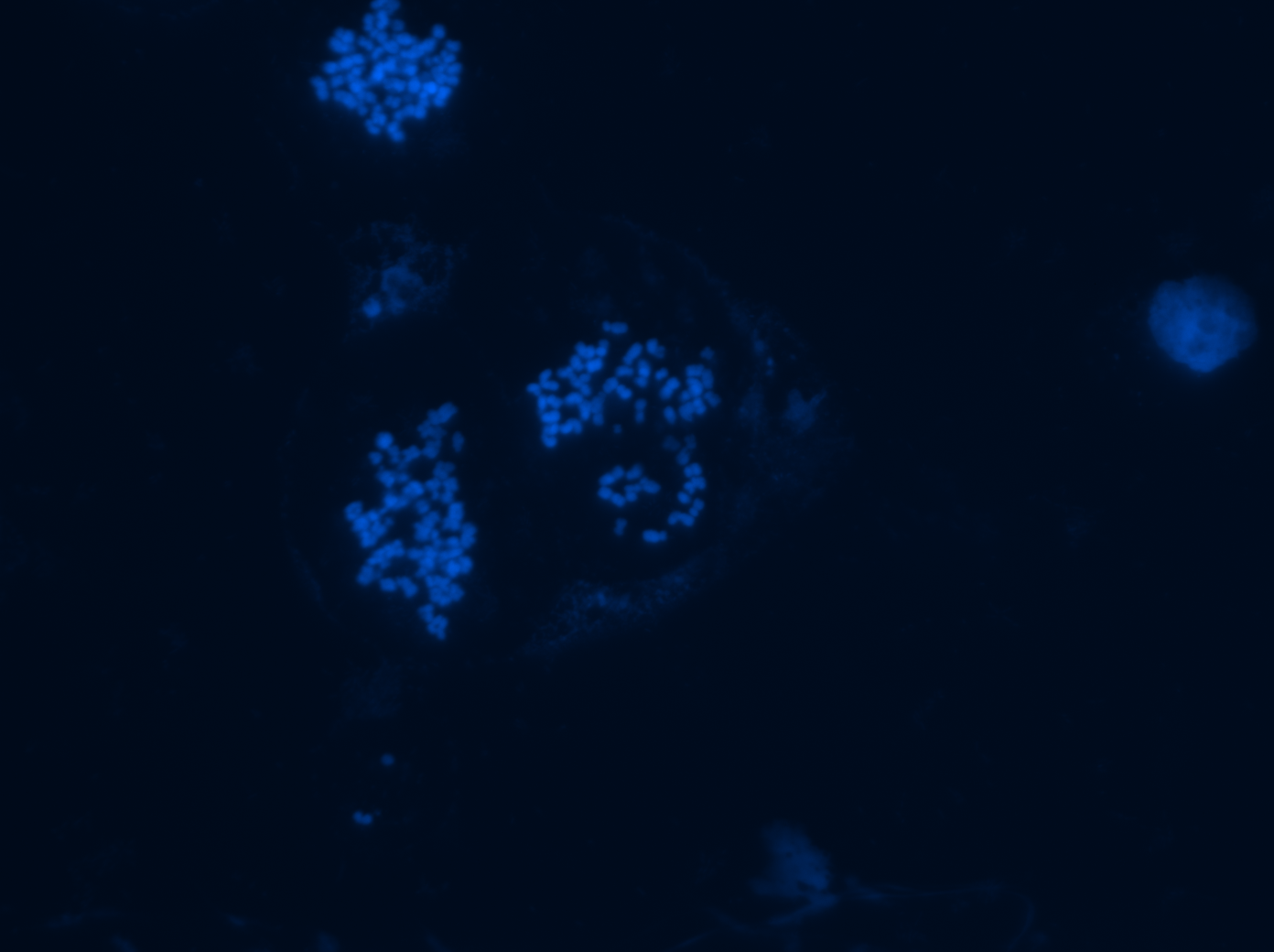

Supplement: Supplementary file 13 — Source data Fig. 7 [file 44318_2025_465_MOESM13_ESM.zip › EMBOJ-2025-120195-Figure 7-Source data/Figure 7/7G/HeLa-siWapl DNA.tif]

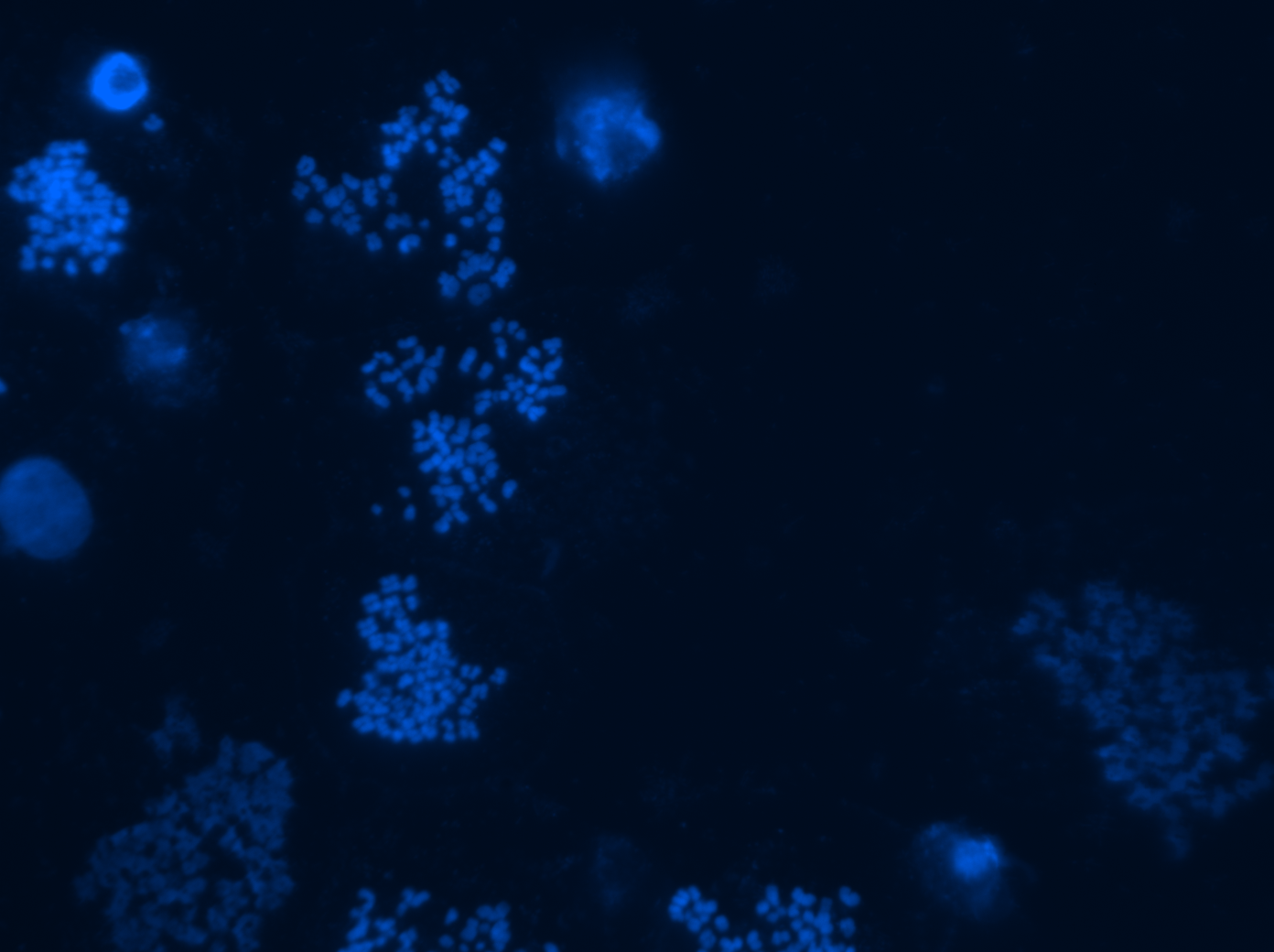

Supplement: Supplementary file 13 — Source data Fig. 7 [file 44318_2025_465_MOESM13_ESM.zip › EMBOJ-2025-120195-Figure 7-Source data/Figure 7/7G/siWapl+siATRX #1 DNA.tif]

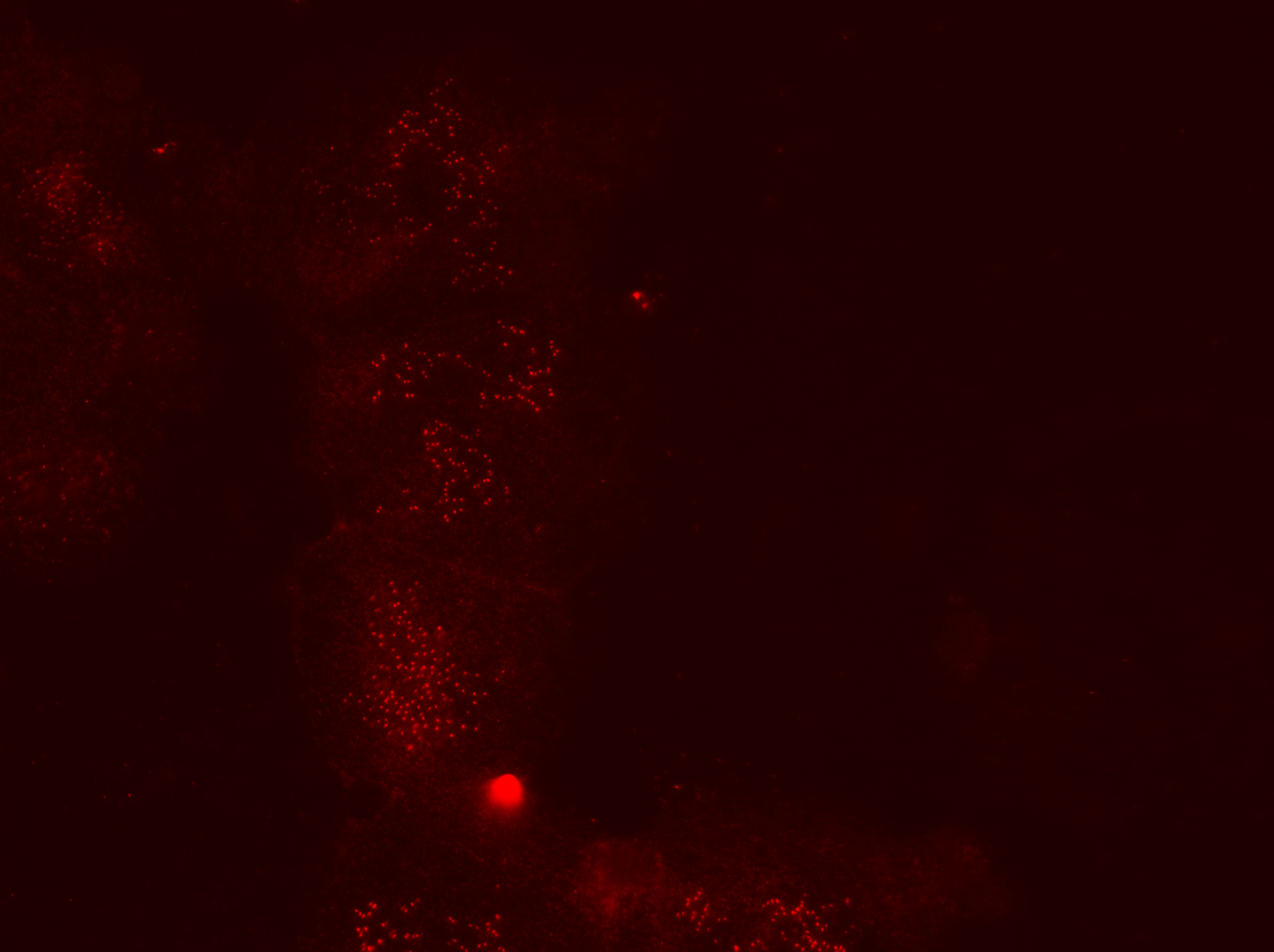

Supplement: Supplementary file 13 — Source data Fig. 7 [file 44318_2025_465_MOESM13_ESM.zip › EMBOJ-2025-120195-Figure 7-Source data/Figure 7/7G/siWapl+siATRX #1 CENP-C.tif]

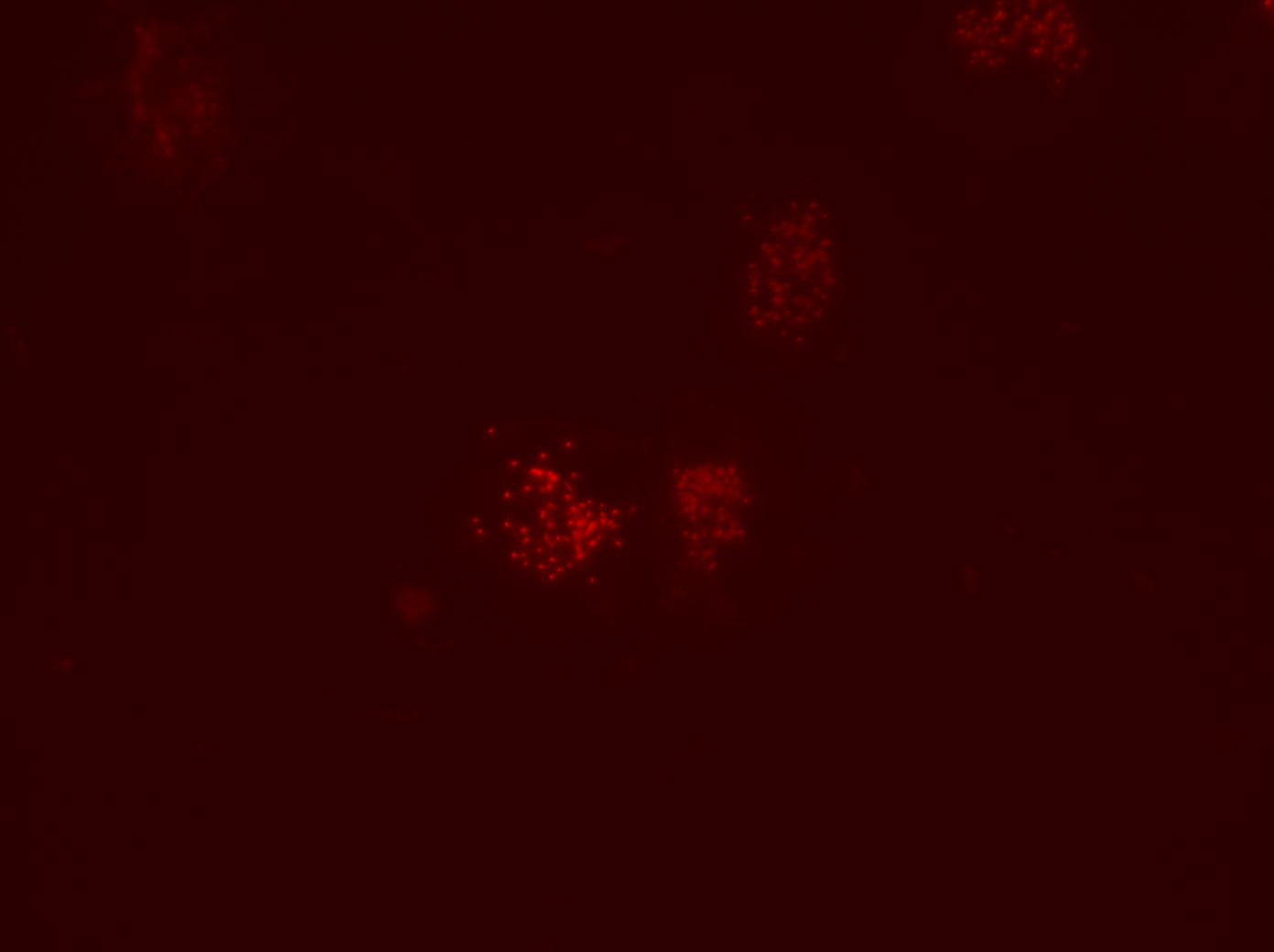

Supplement: Supplementary file 13 — Source data Fig. 7 [file 44318_2025_465_MOESM13_ESM.zip › EMBOJ-2025-120195-Figure 7-Source data/Figure 7/7H/HeLa-siControl ATRX.tif]

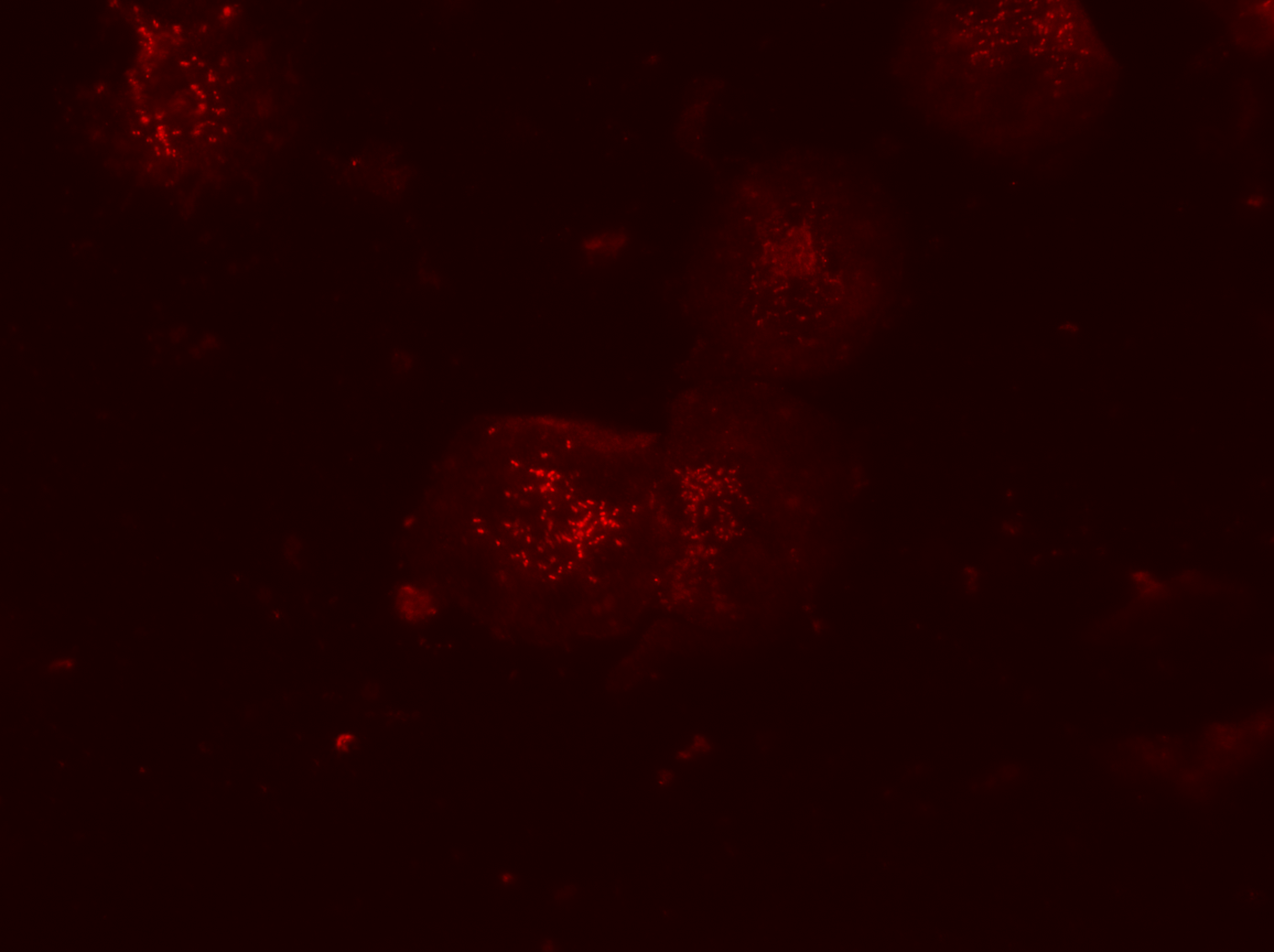

Supplement: Supplementary file 13 — Source data Fig. 7 [file 44318_2025_465_MOESM13_ESM.zip › EMBOJ-2025-120195-Figure 7-Source data/Figure 7/7H/HeLa-siControl ACA.tif]

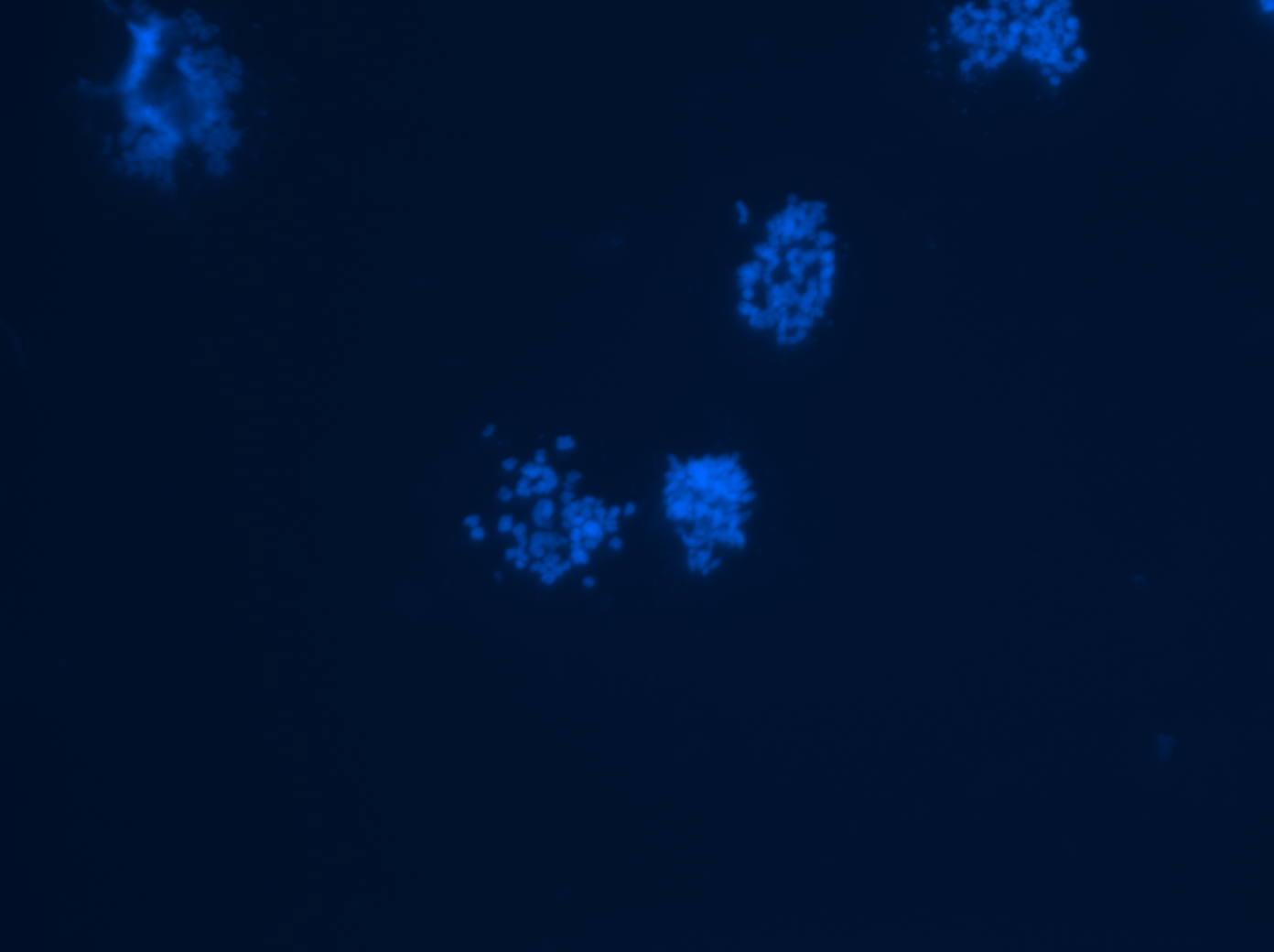

Supplement: Supplementary file 13 — Source data Fig. 7 [file 44318_2025_465_MOESM13_ESM.zip › EMBOJ-2025-120195-Figure 7-Source data/Figure 7/7H/HeLa-siControl DNA.tif]

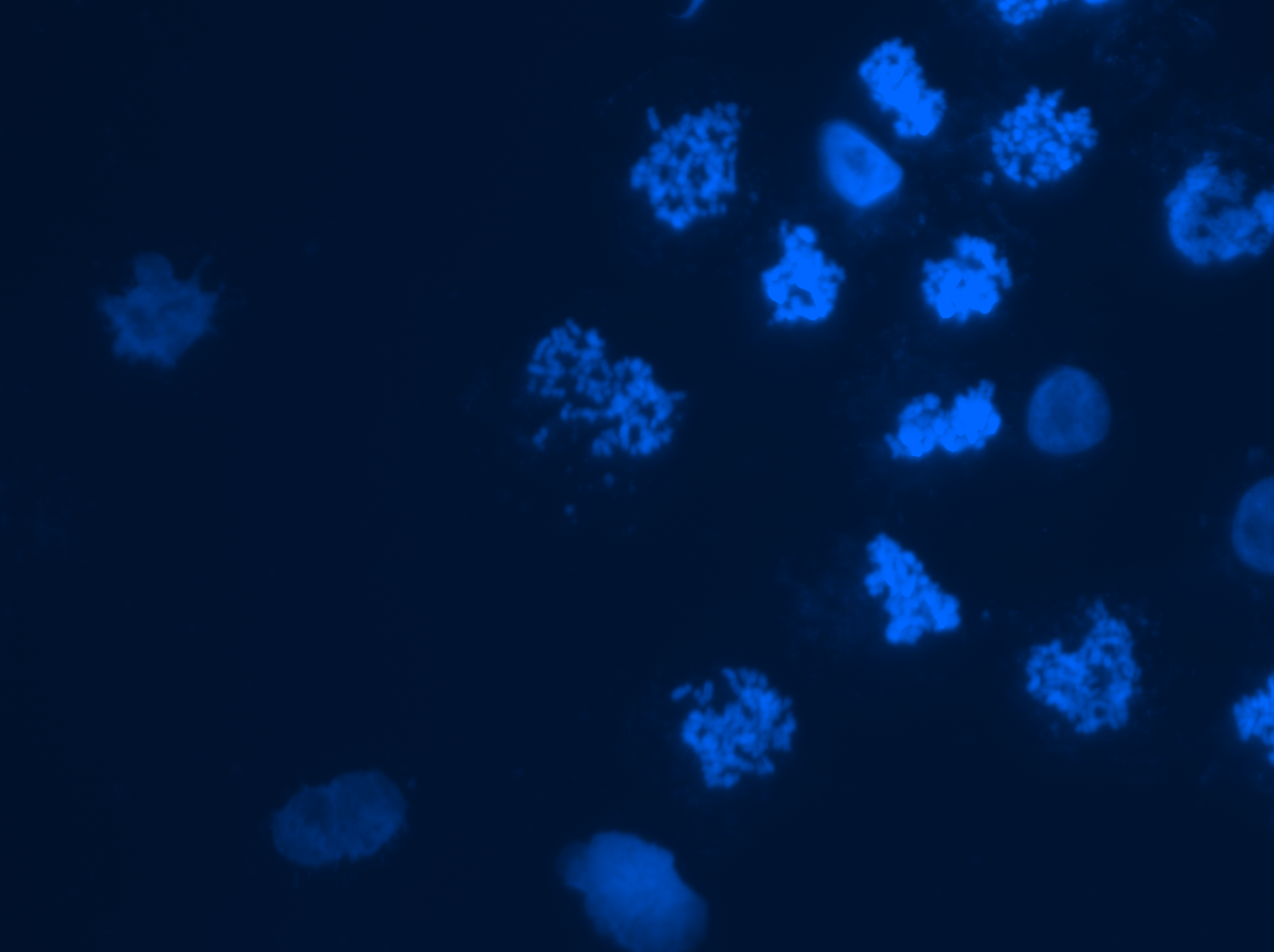

Supplement: Supplementary file 13 — Source data Fig. 7 [file 44318_2025_465_MOESM13_ESM.zip › EMBOJ-2025-120195-Figure 7-Source data/Figure 7/7H/HeLa-siWapl DNA.tif]

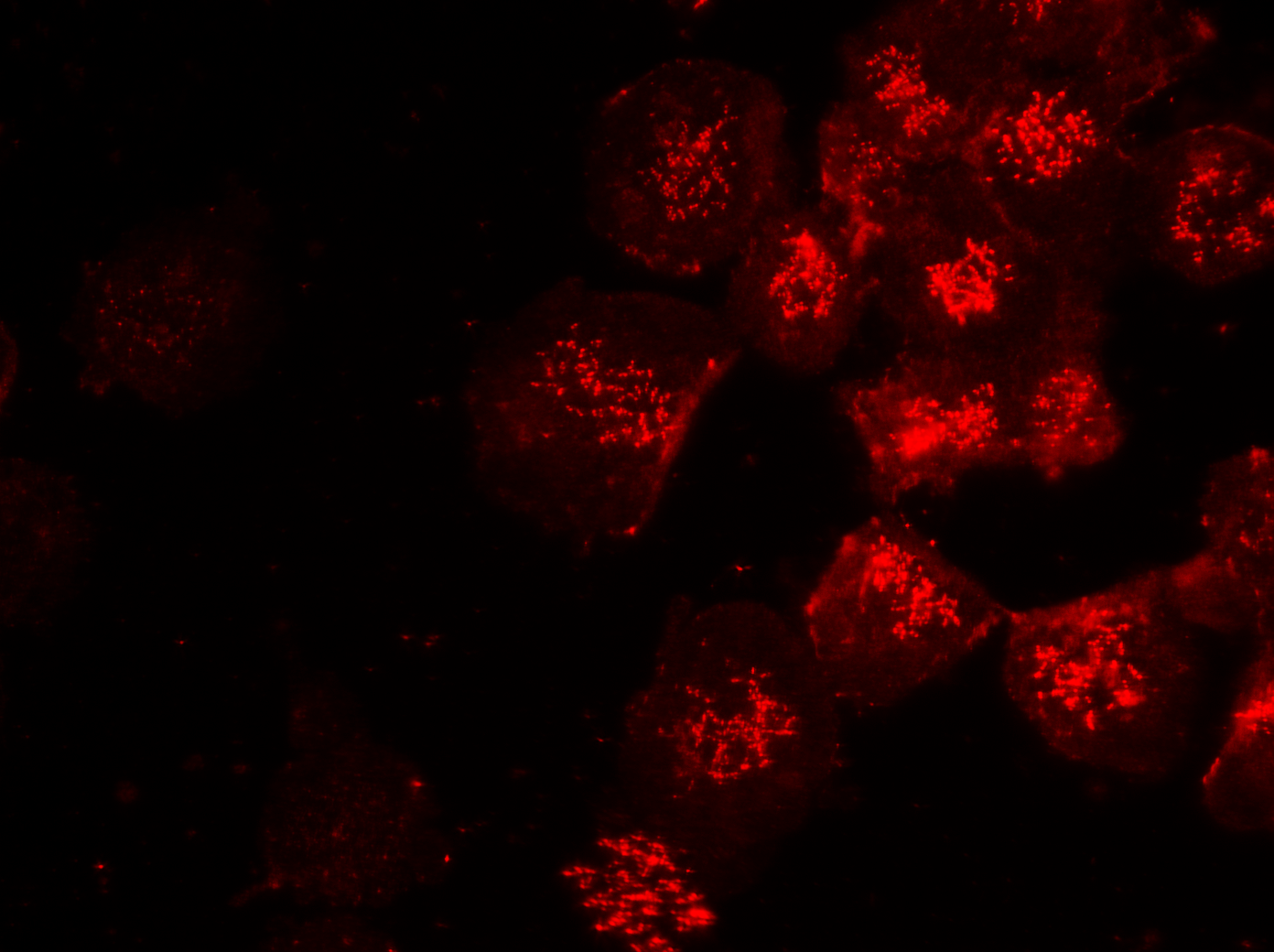

Supplement: Supplementary file 13 — Source data Fig. 7 [file 44318_2025_465_MOESM13_ESM.zip › EMBOJ-2025-120195-Figure 7-Source data/Figure 7/7H/HeLa-siWapl ACA.tif]

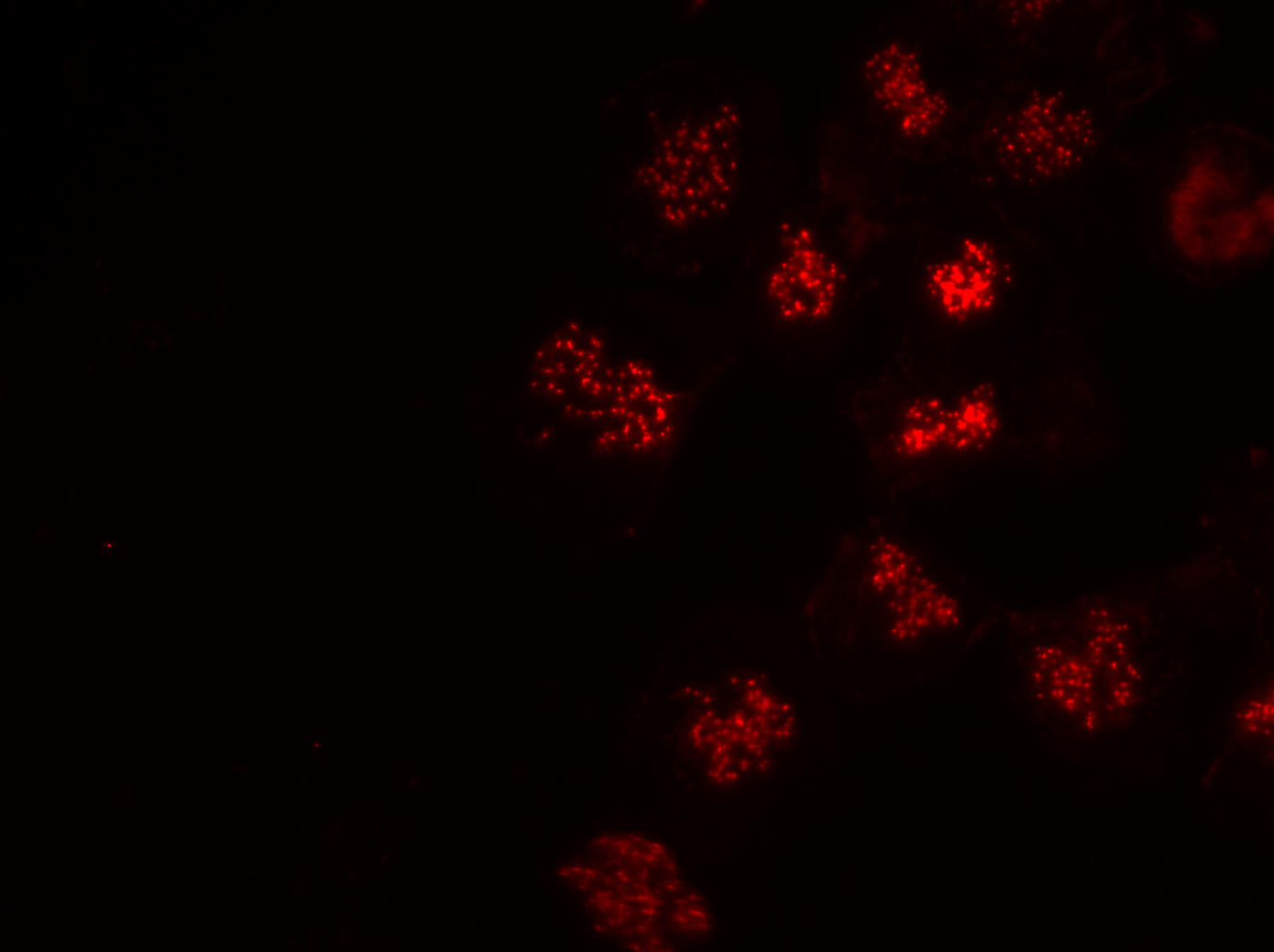

Supplement: Supplementary file 13 — Source data Fig. 7 [file 44318_2025_465_MOESM13_ESM.zip › EMBOJ-2025-120195-Figure 7-Source data/Figure 7/7H/HeLa-siWapl ATRX.tif]

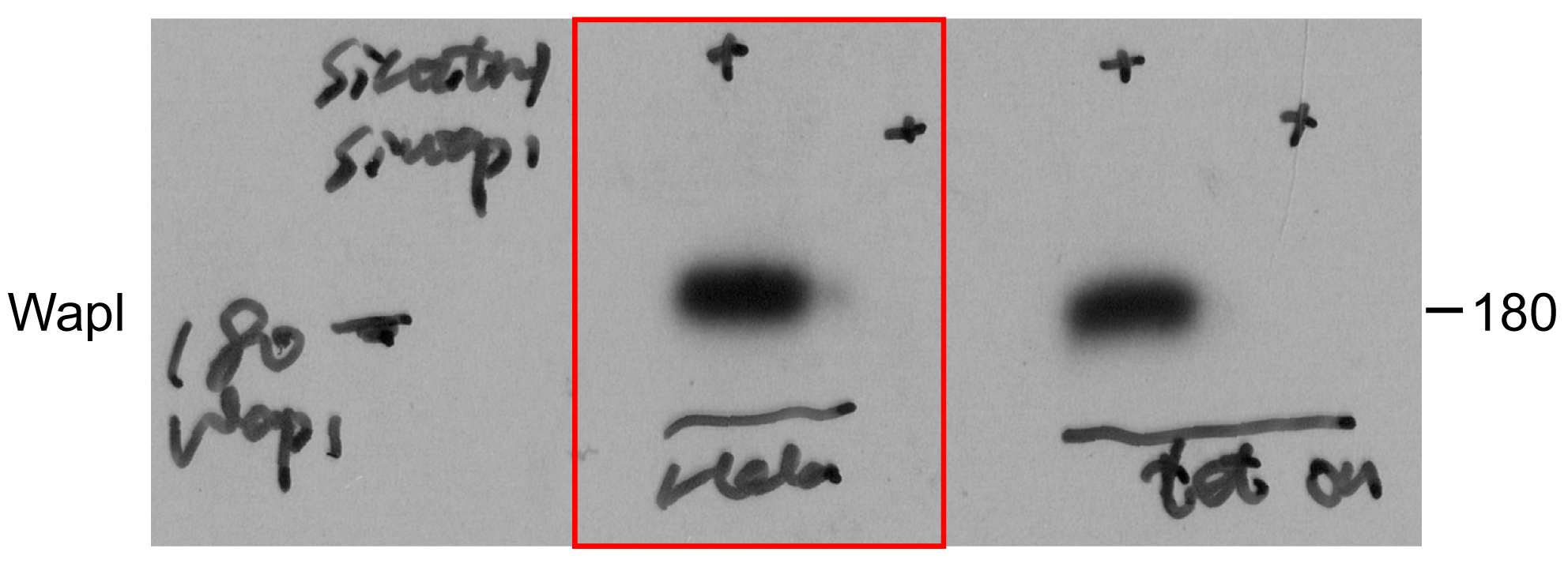

Supplement: Supplementary file 13 — Source data Fig. 7 [file 44318_2025_465_MOESM13_ESM.zip › EMBOJ-2025-120195-Figure 7-Source data/Figure 7/7I/western Wapl.tif]

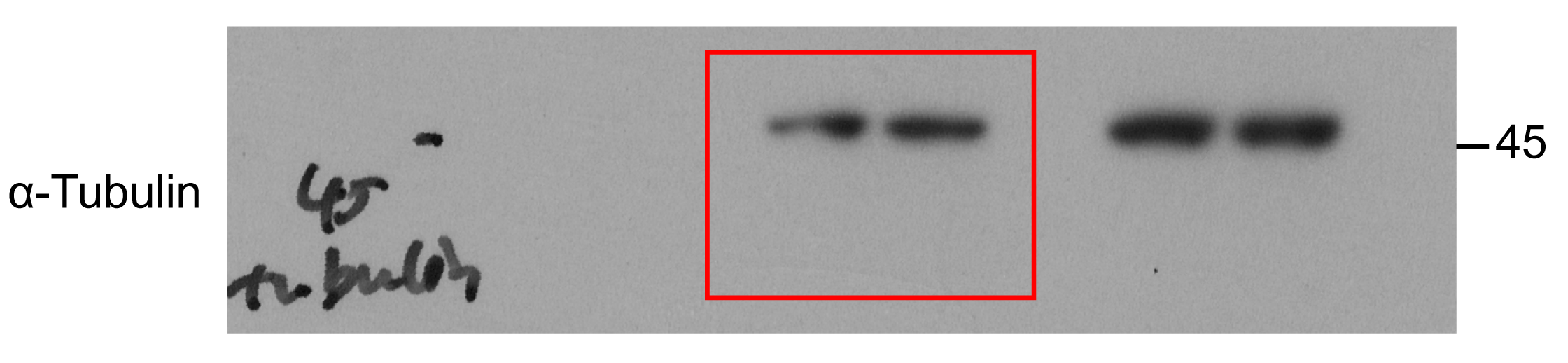

Supplement: Supplementary file 13 — Source data Fig. 7 [file 44318_2025_465_MOESM13_ESM.zip › EMBOJ-2025-120195-Figure 7-Source data/Figure 7/7I/western α-Tubulin.tif]

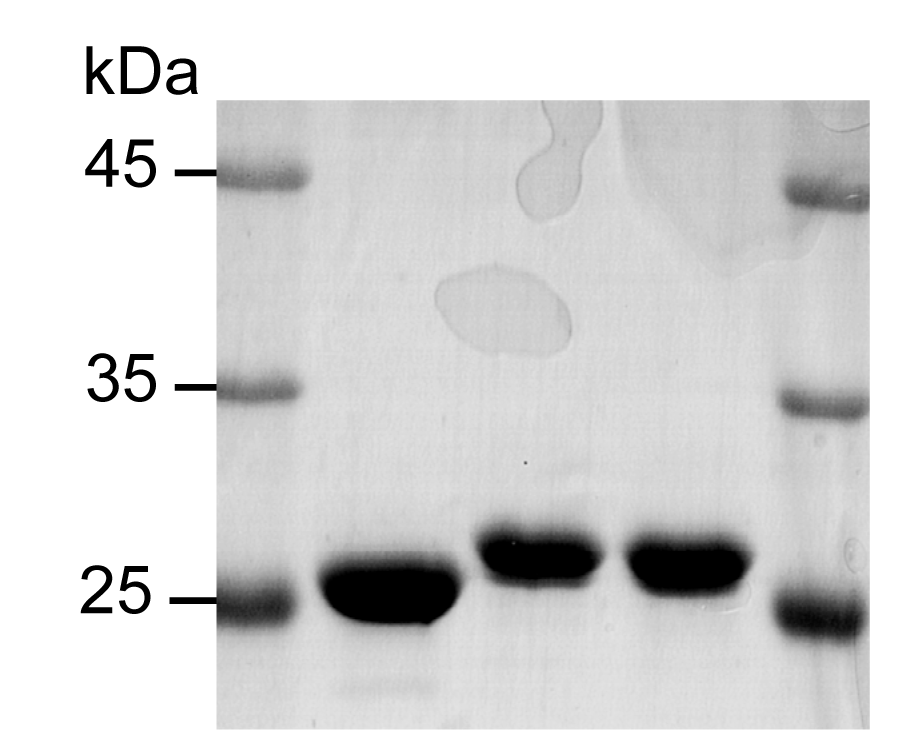

Supplement: Supplementary file 14 — Source data Fig. 8 [file 44318_2025_465_MOESM14_ESM.zip › EMBOJ-2025-120195-Figure 8-Source data/Figure 8/8A/CBB.tif]

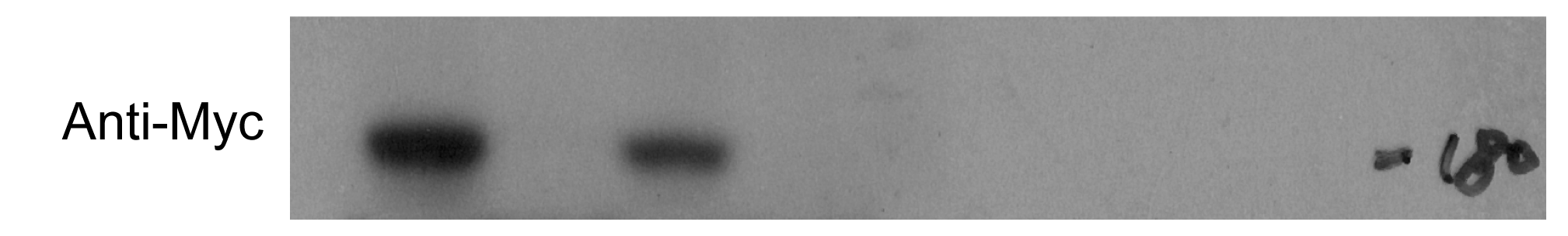

Supplement: Supplementary file 14 — Source data Fig. 8 [file 44318_2025_465_MOESM14_ESM.zip › EMBOJ-2025-120195-Figure 8-Source data/Figure 8/8A/western Myc.tif]

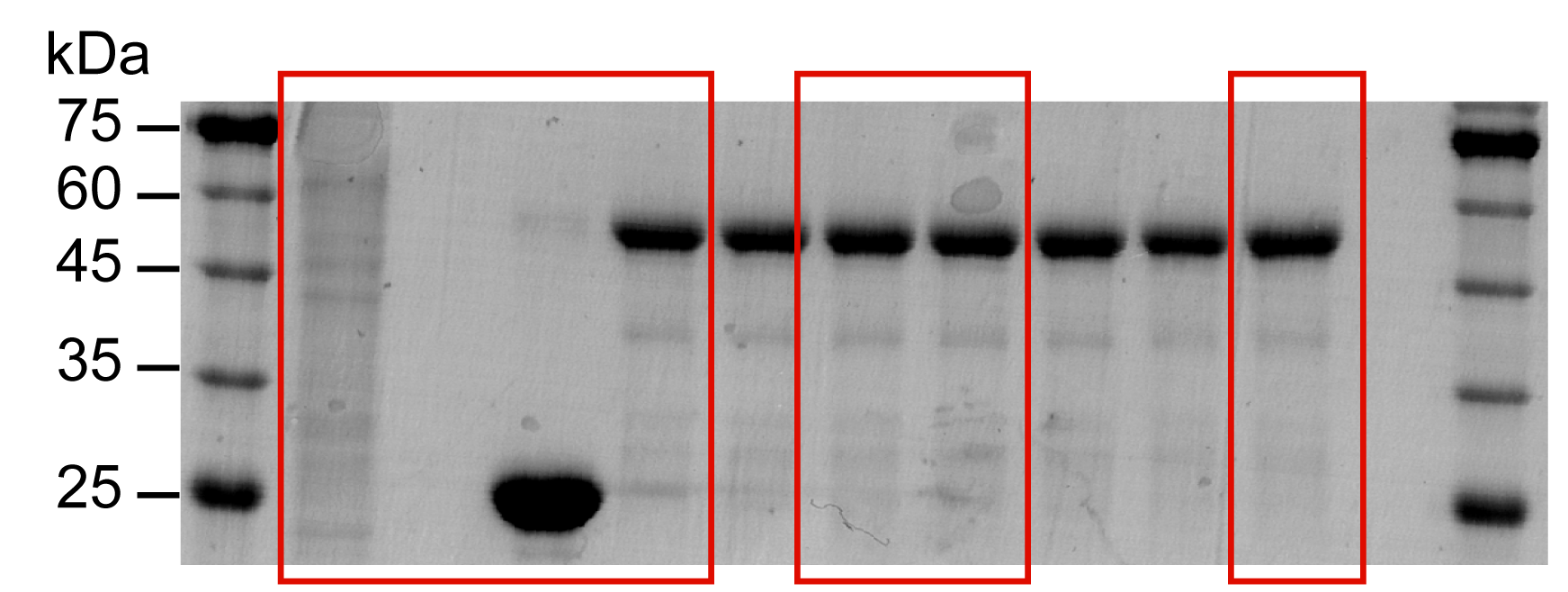

Supplement: Supplementary file 14 — Source data Fig. 8 [file 44318_2025_465_MOESM14_ESM.zip › EMBOJ-2025-120195-Figure 8-Source data/Figure 8/8C/CBB.tif]

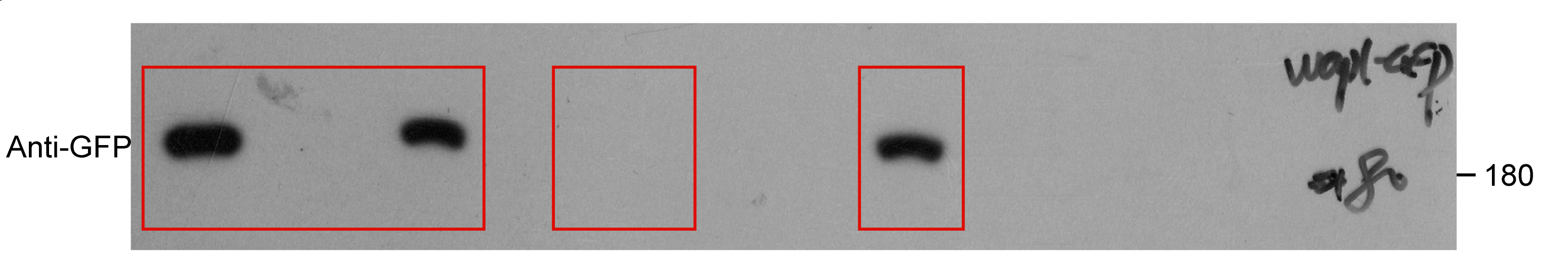

Supplement: Supplementary file 14 — Source data Fig. 8 [file 44318_2025_465_MOESM14_ESM.zip › EMBOJ-2025-120195-Figure 8-Source data/Figure 8/8C/western GFP.tif]

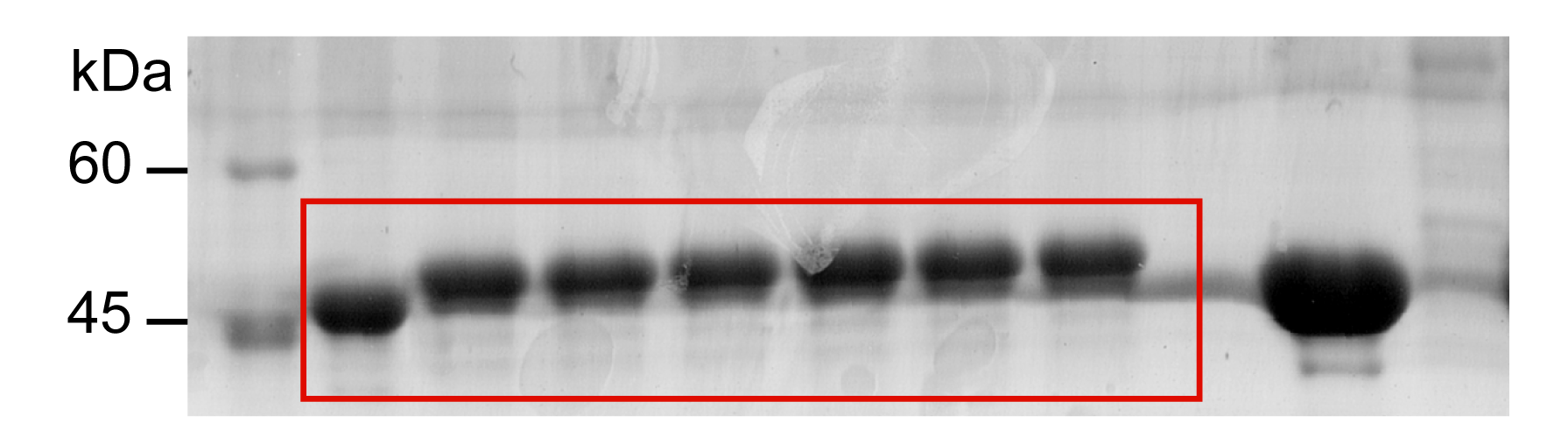

Supplement: Supplementary file 14 — Source data Fig. 8 [file 44318_2025_465_MOESM14_ESM.zip › EMBOJ-2025-120195-Figure 8-Source data/Figure 8/8D/CBB.tif]

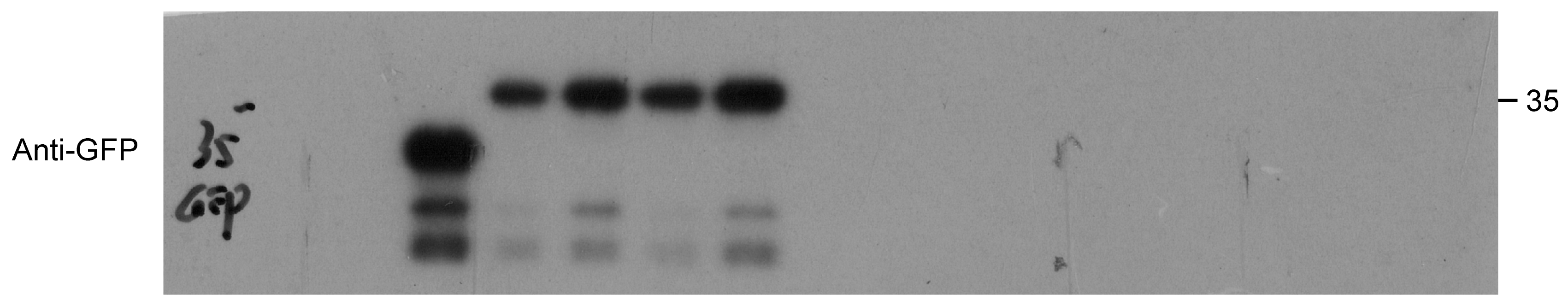

Supplement: Supplementary file 14 — Source data Fig. 8 [file 44318_2025_465_MOESM14_ESM.zip › EMBOJ-2025-120195-Figure 8-Source data/Figure 8/8D/western GFP.tif]

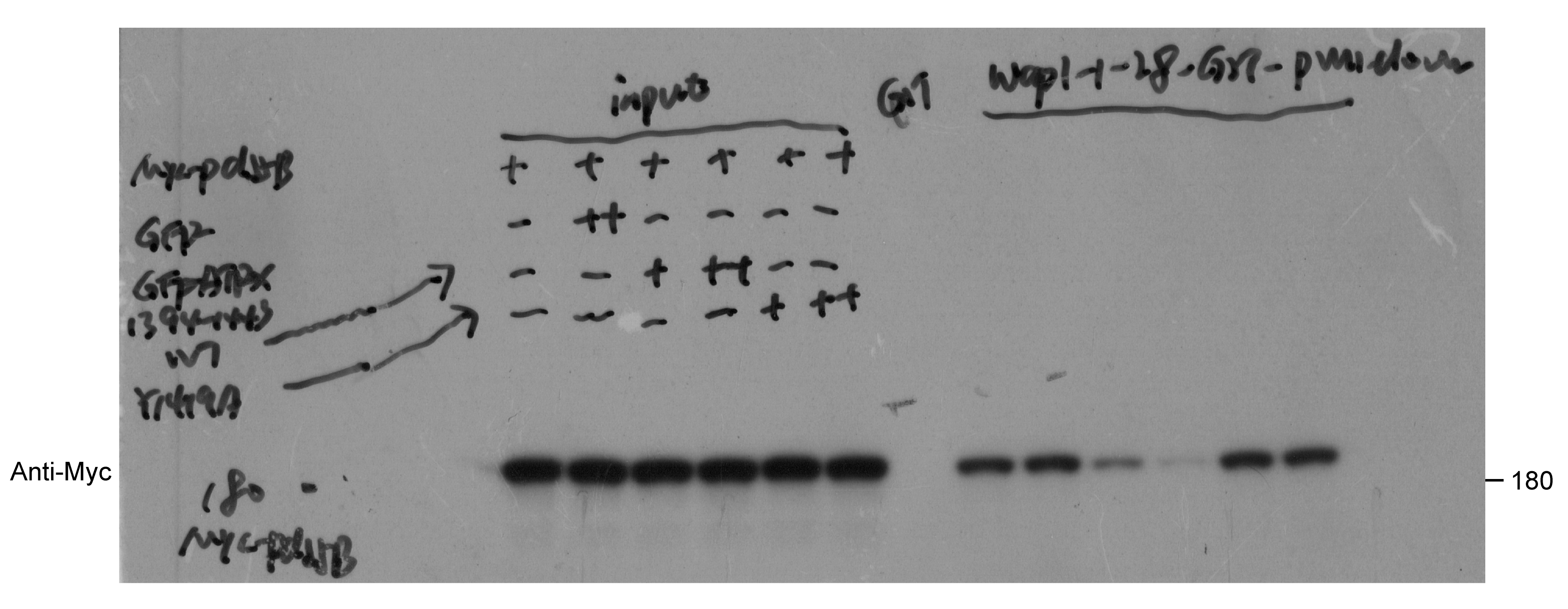

Supplement: Supplementary file 14 — Source data Fig. 8 [file 44318_2025_465_MOESM14_ESM.zip › EMBOJ-2025-120195-Figure 8-Source data/Figure 8/8D/western Myc.tif]

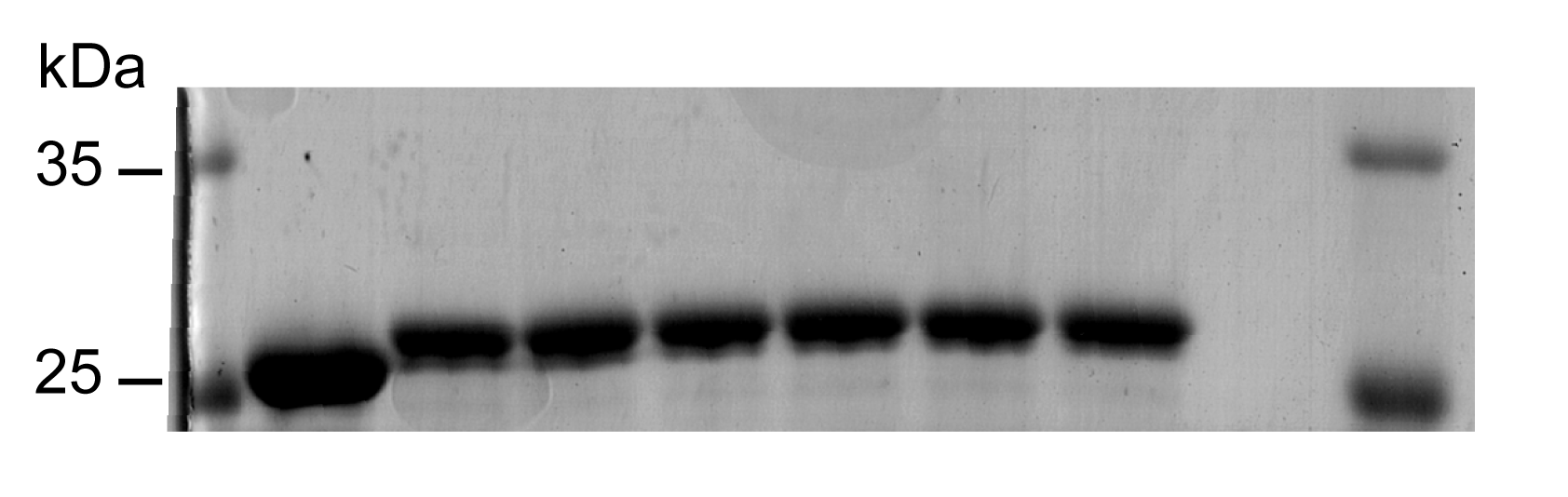

Supplement: Supplementary file 14 — Source data Fig. 8 [file 44318_2025_465_MOESM14_ESM.zip › EMBOJ-2025-120195-Figure 8-Source data/Figure 8/8E/CBB.tif]
