# Supplementary figures and images for: A chromatin-remodeling-independent role for ATRX in protecting centromeric cohesion (part 3 of 3)
Source: EMBO J. 2025 May 28;44(14):4037–64. doi: 10.1038/s44318-025-00465-6 (PMC12264150; doi:10.1038/s44318-025-00465-6)

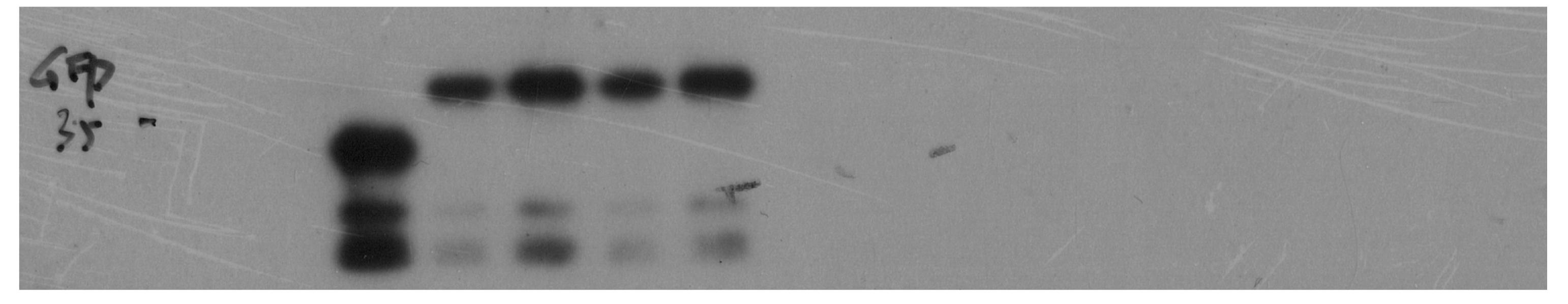

Supplement: Supplementary file 14 — Source data Fig. 8 [file 44318_2025_465_MOESM14_ESM.zip › EMBOJ-2025-120195-Figure 8-Source data/Figure 8/8E/western GFP.tif]

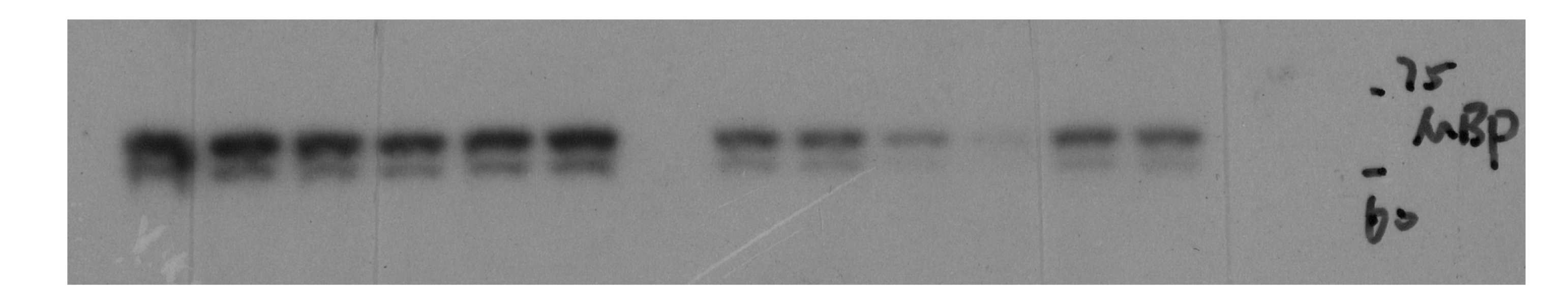

Supplement: Supplementary file 14 — Source data Fig. 8 [file 44318_2025_465_MOESM14_ESM.zip › EMBOJ-2025-120195-Figure 8-Source data/Figure 8/8E/westren MBP.tif]

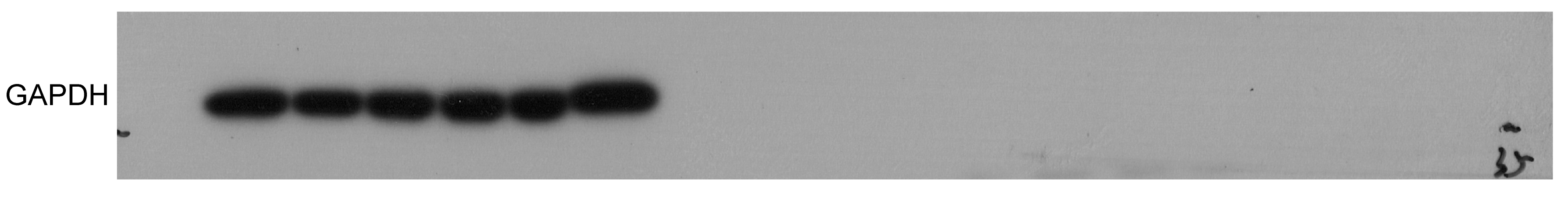

Supplement: Supplementary file 14 — Source data Fig. 8 [file 44318_2025_465_MOESM14_ESM.zip › EMBOJ-2025-120195-Figure 8-Source data/Figure 8/8F/western GAPDH.tif]

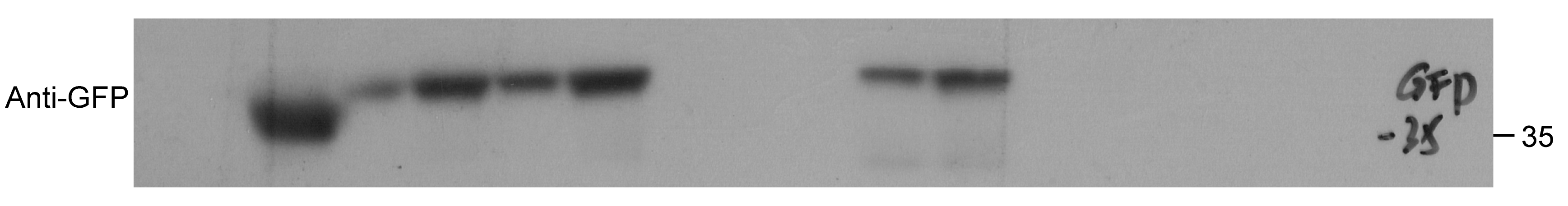

Supplement: Supplementary file 14 — Source data Fig. 8 [file 44318_2025_465_MOESM14_ESM.zip › EMBOJ-2025-120195-Figure 8-Source data/Figure 8/8F/western GFP.tif]

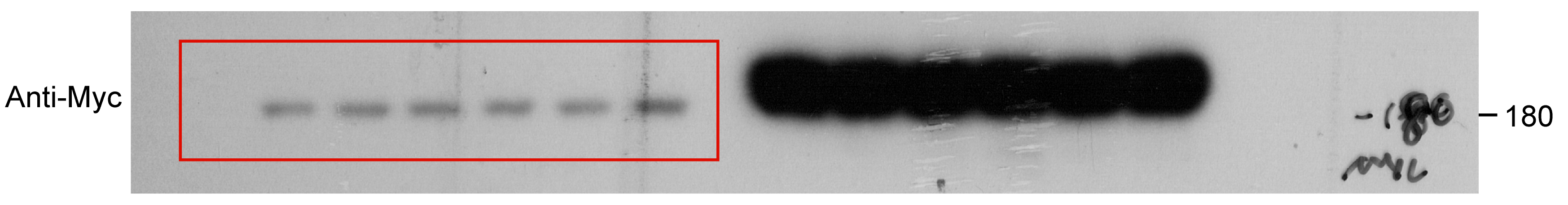

Supplement: Supplementary file 14 — Source data Fig. 8 [file 44318_2025_465_MOESM14_ESM.zip › EMBOJ-2025-120195-Figure 8-Source data/Figure 8/8F/western Myc-L.exp..tif]

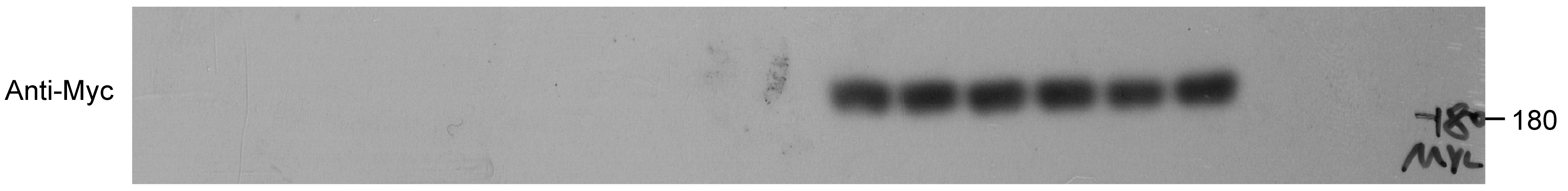

Supplement: Supplementary file 14 — Source data Fig. 8 [file 44318_2025_465_MOESM14_ESM.zip › EMBOJ-2025-120195-Figure 8-Source data/Figure 8/8F/western Myc-S.exp..tif]

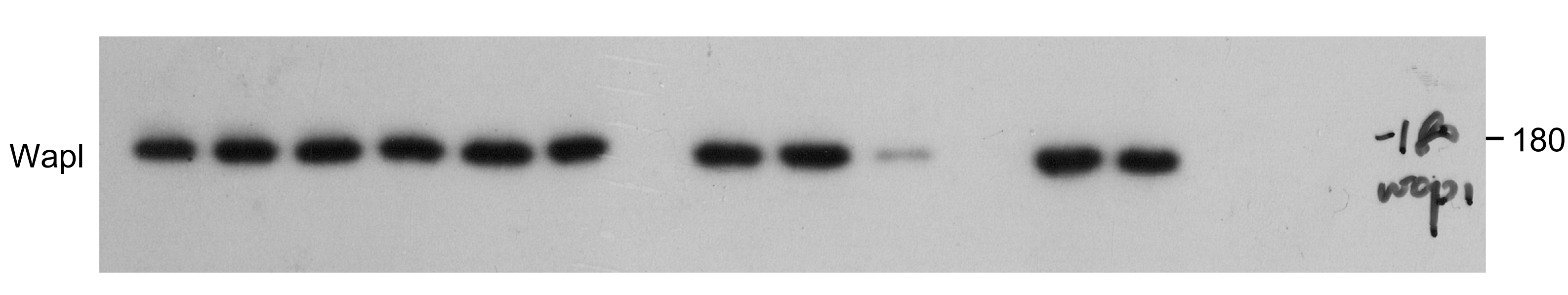

Supplement: Supplementary file 14 — Source data Fig. 8 [file 44318_2025_465_MOESM14_ESM.zip › EMBOJ-2025-120195-Figure 8-Source data/Figure 8/8F/western Wapl.tif]

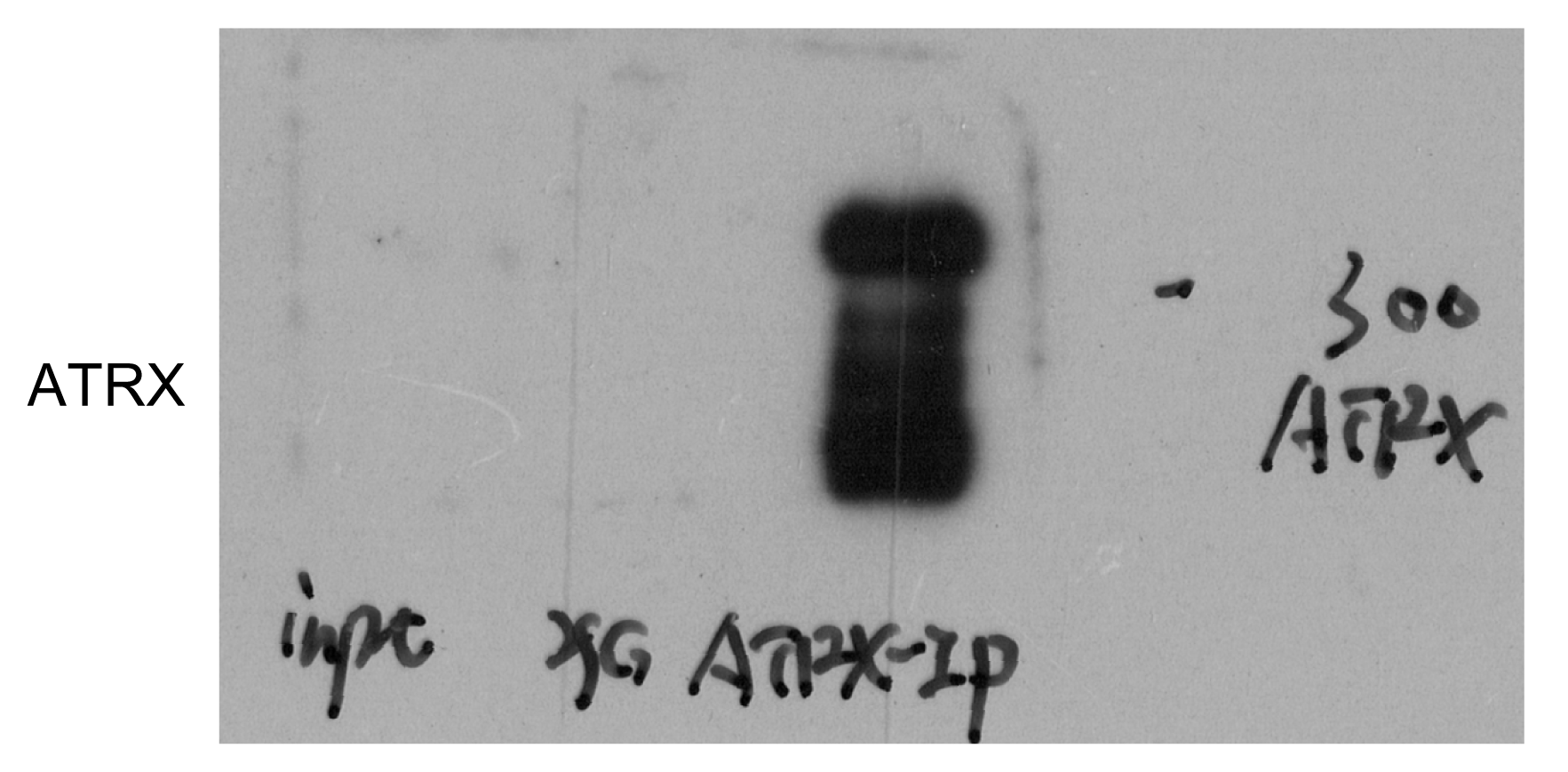

Supplement: Supplementary file 14 — Source data Fig. 8 [file 44318_2025_465_MOESM14_ESM.zip › EMBOJ-2025-120195-Figure 8-Source data/Figure 8/8G/western ATRX.tif]

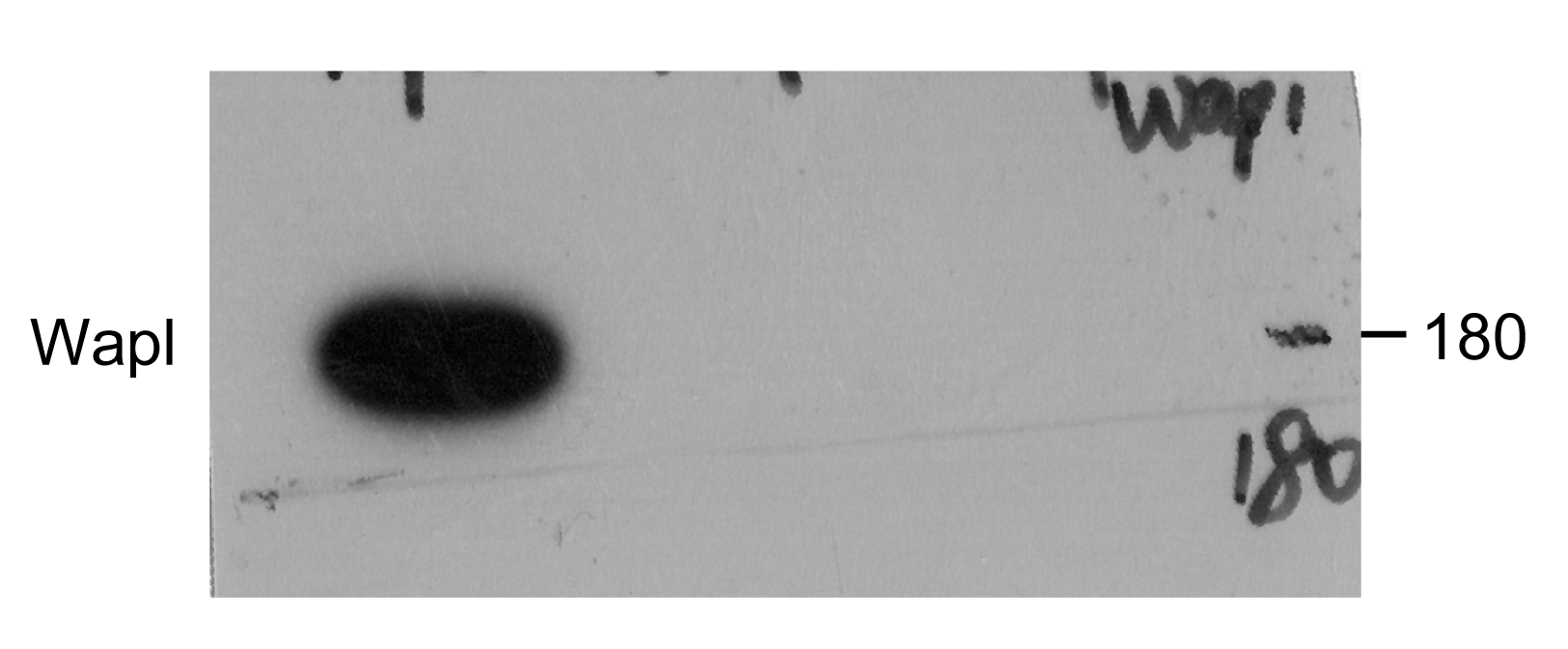

Supplement: Supplementary file 14 — Source data Fig. 8 [file 44318_2025_465_MOESM14_ESM.zip › EMBOJ-2025-120195-Figure 8-Source data/Figure 8/8G/western Wapl.tif]

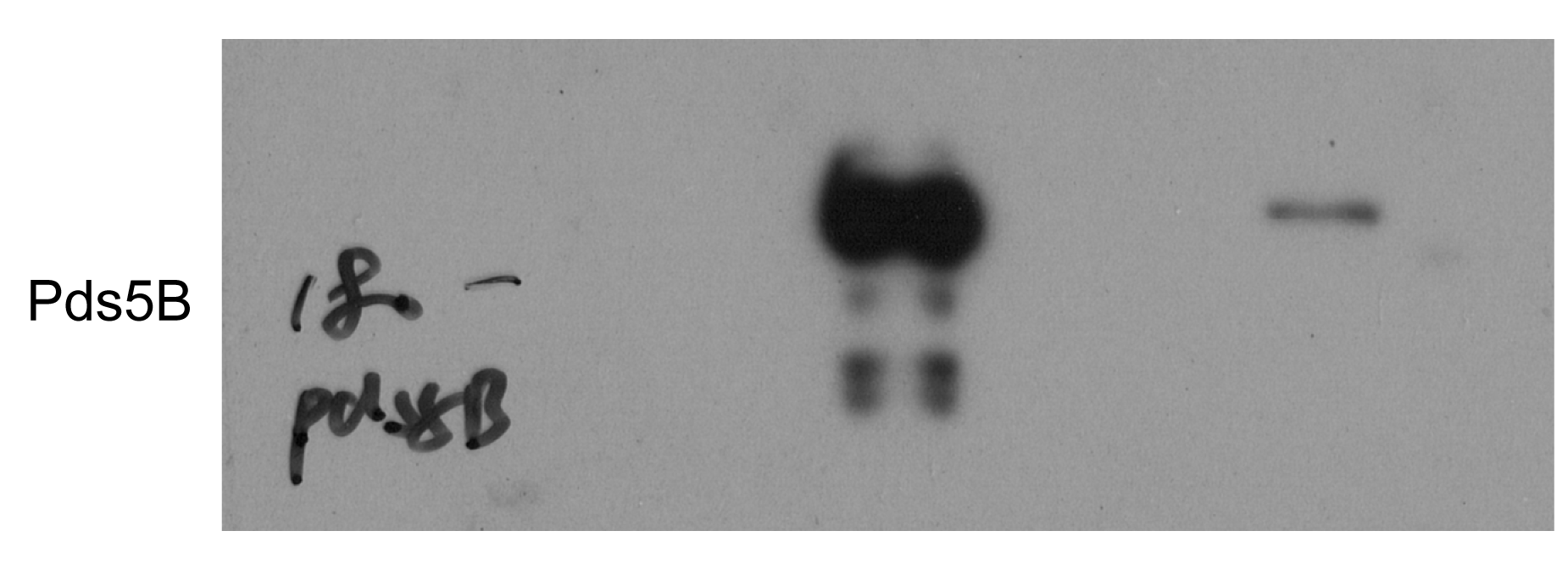

Supplement: Supplementary file 14 — Source data Fig. 8 [file 44318_2025_465_MOESM14_ESM.zip › EMBOJ-2025-120195-Figure 8-Source data/Figure 8/8G/westren Pds5B.tif]

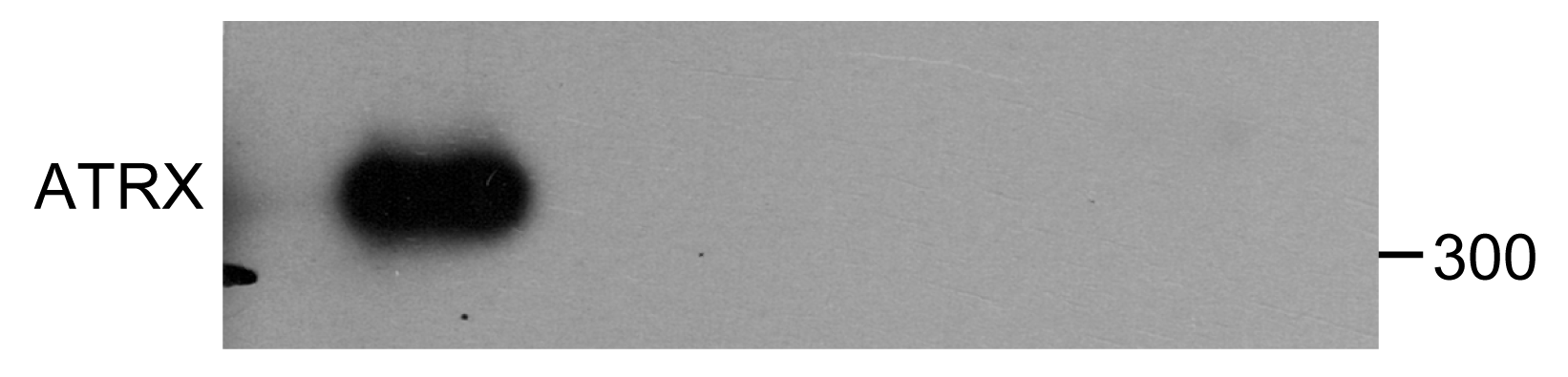

Supplement: Supplementary file 14 — Source data Fig. 8 [file 44318_2025_465_MOESM14_ESM.zip › EMBOJ-2025-120195-Figure 8-Source data/Figure 8/8H/western ATRX.tif]

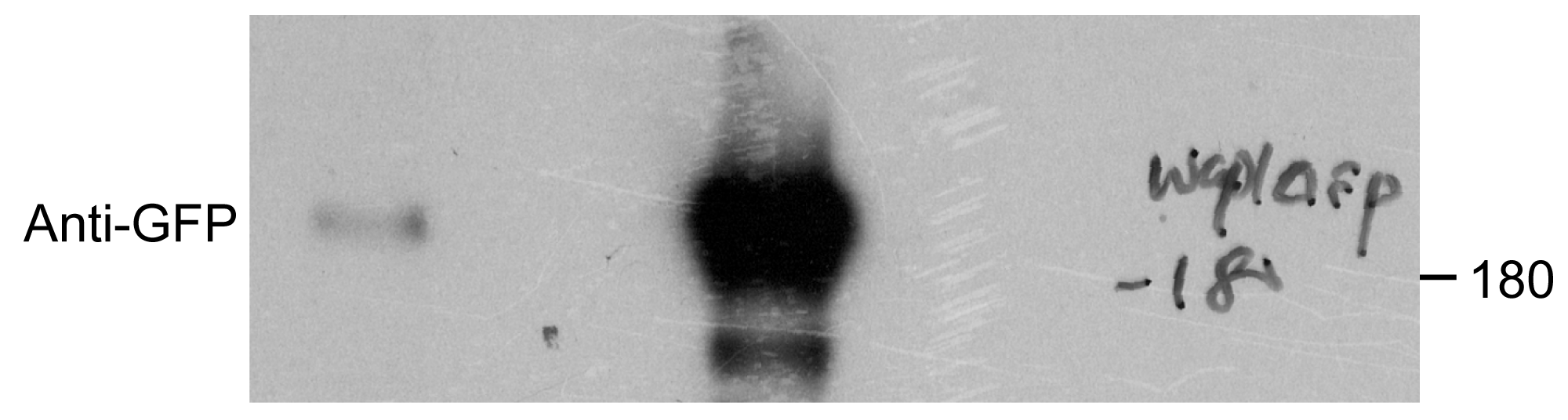

Supplement: Supplementary file 14 — Source data Fig. 8 [file 44318_2025_465_MOESM14_ESM.zip › EMBOJ-2025-120195-Figure 8-Source data/Figure 8/8H/western GFP.tif]

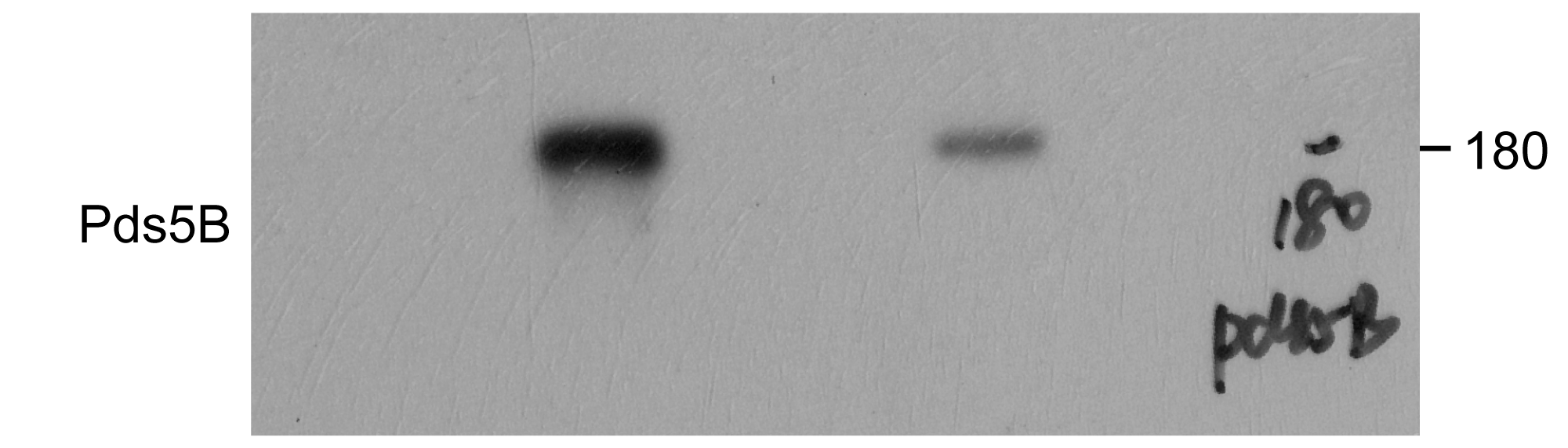

Supplement: Supplementary file 14 — Source data Fig. 8 [file 44318_2025_465_MOESM14_ESM.zip › EMBOJ-2025-120195-Figure 8-Source data/Figure 8/8H/western Pds5B.tif]

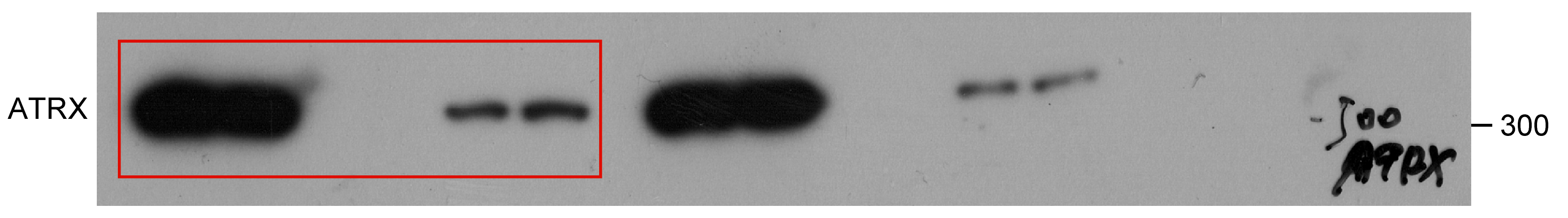

Supplement: Supplementary file 14 — Source data Fig. 8 [file 44318_2025_465_MOESM14_ESM.zip › EMBOJ-2025-120195-Figure 8-Source data/Figure 8/8I/western ATRX.tif]

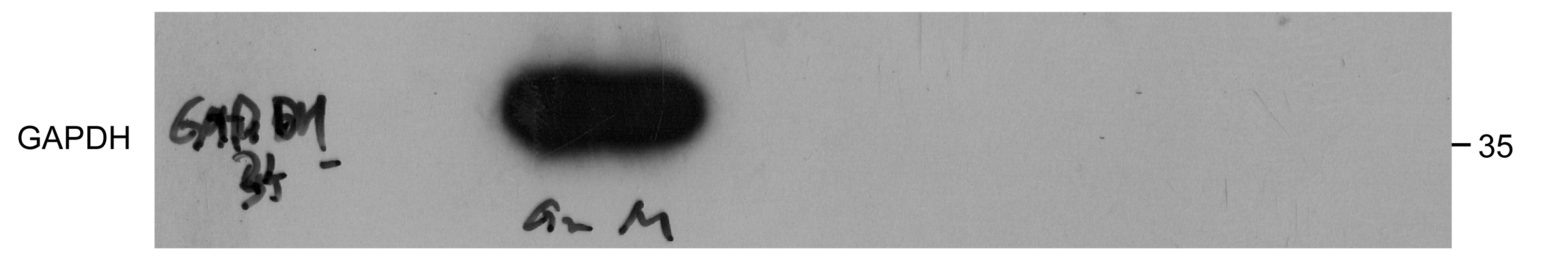

Supplement: Supplementary file 14 — Source data Fig. 8 [file 44318_2025_465_MOESM14_ESM.zip › EMBOJ-2025-120195-Figure 8-Source data/Figure 8/8I/western GAPDH.tif]

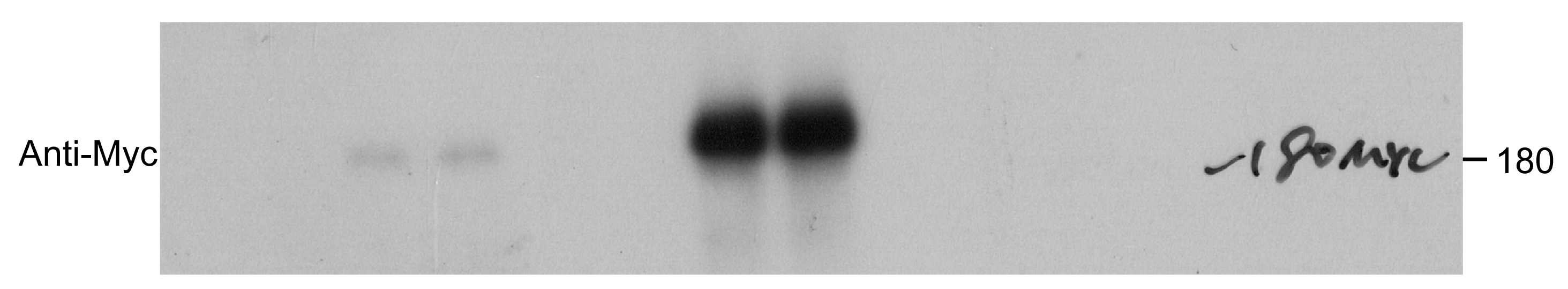

Supplement: Supplementary file 14 — Source data Fig. 8 [file 44318_2025_465_MOESM14_ESM.zip › EMBOJ-2025-120195-Figure 8-Source data/Figure 8/8I/western Myc.tif]

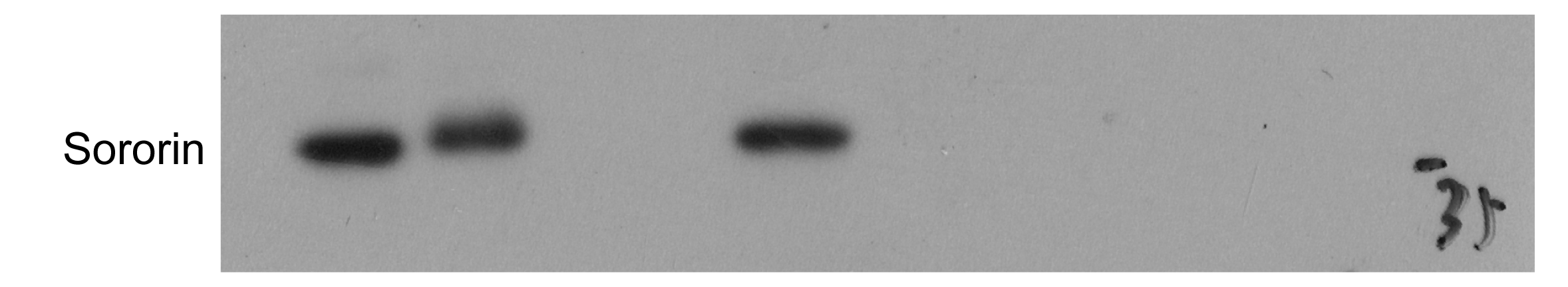

Supplement: Supplementary file 14 — Source data Fig. 8 [file 44318_2025_465_MOESM14_ESM.zip › EMBOJ-2025-120195-Figure 8-Source data/Figure 8/8I/western Sororin.tif]

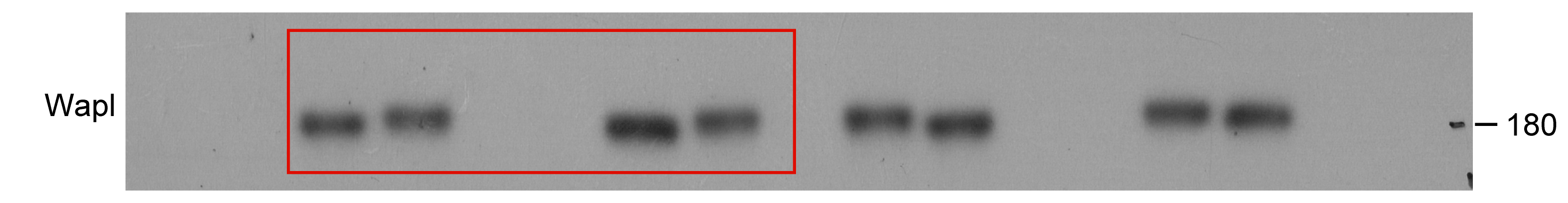

Supplement: Supplementary file 14 — Source data Fig. 8 [file 44318_2025_465_MOESM14_ESM.zip › EMBOJ-2025-120195-Figure 8-Source data/Figure 8/8I/western Wapl.tif]

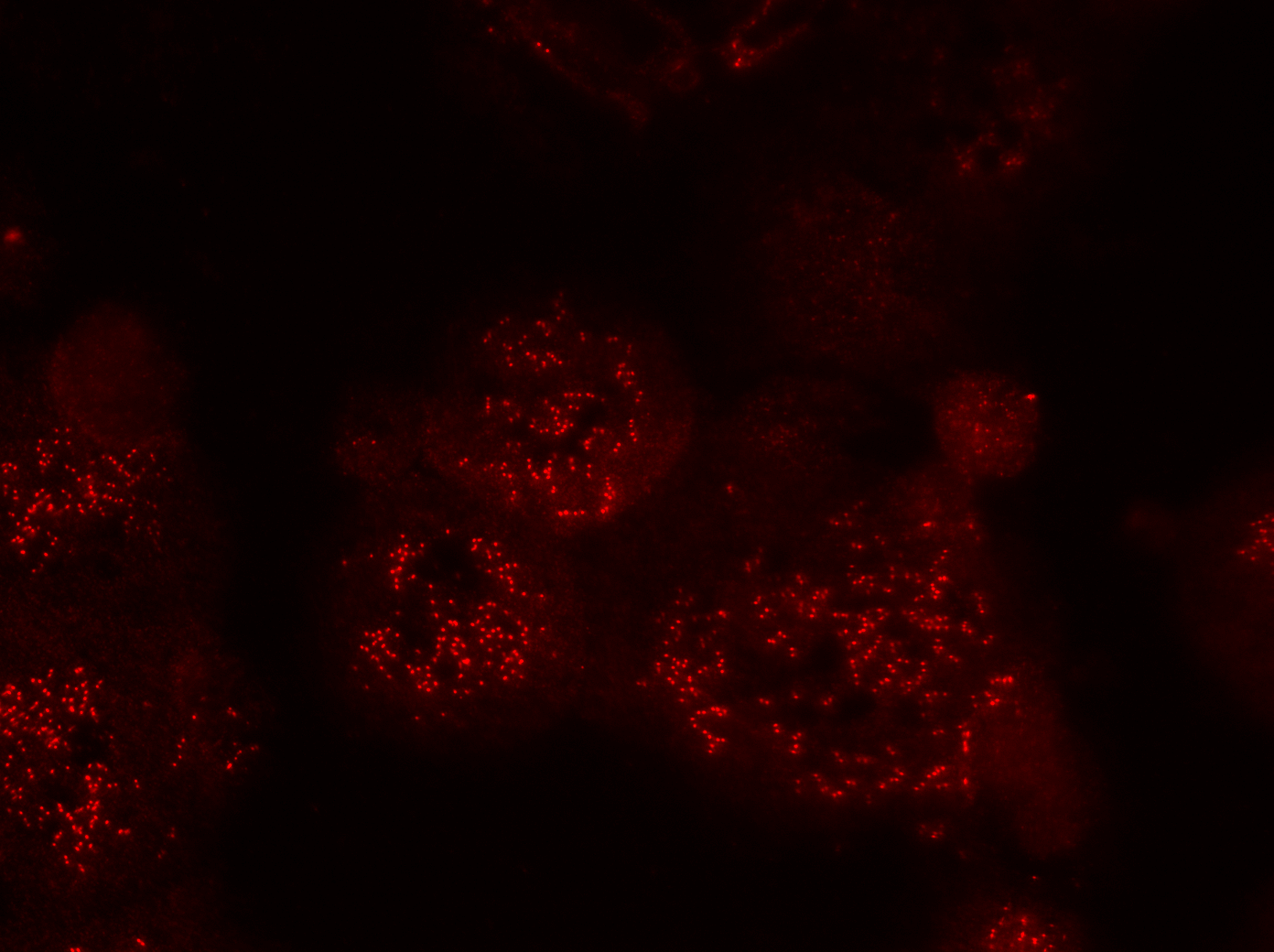

Supplement: Supplementary file 15 — Source data Fig. 9 [file 44318_2025_465_MOESM15_ESM.zip › EMBOJ-2025-120195-Figure 9-Source data/Figure 9/9B/Haspin KO-siATRX #3 CENP-C.tif]

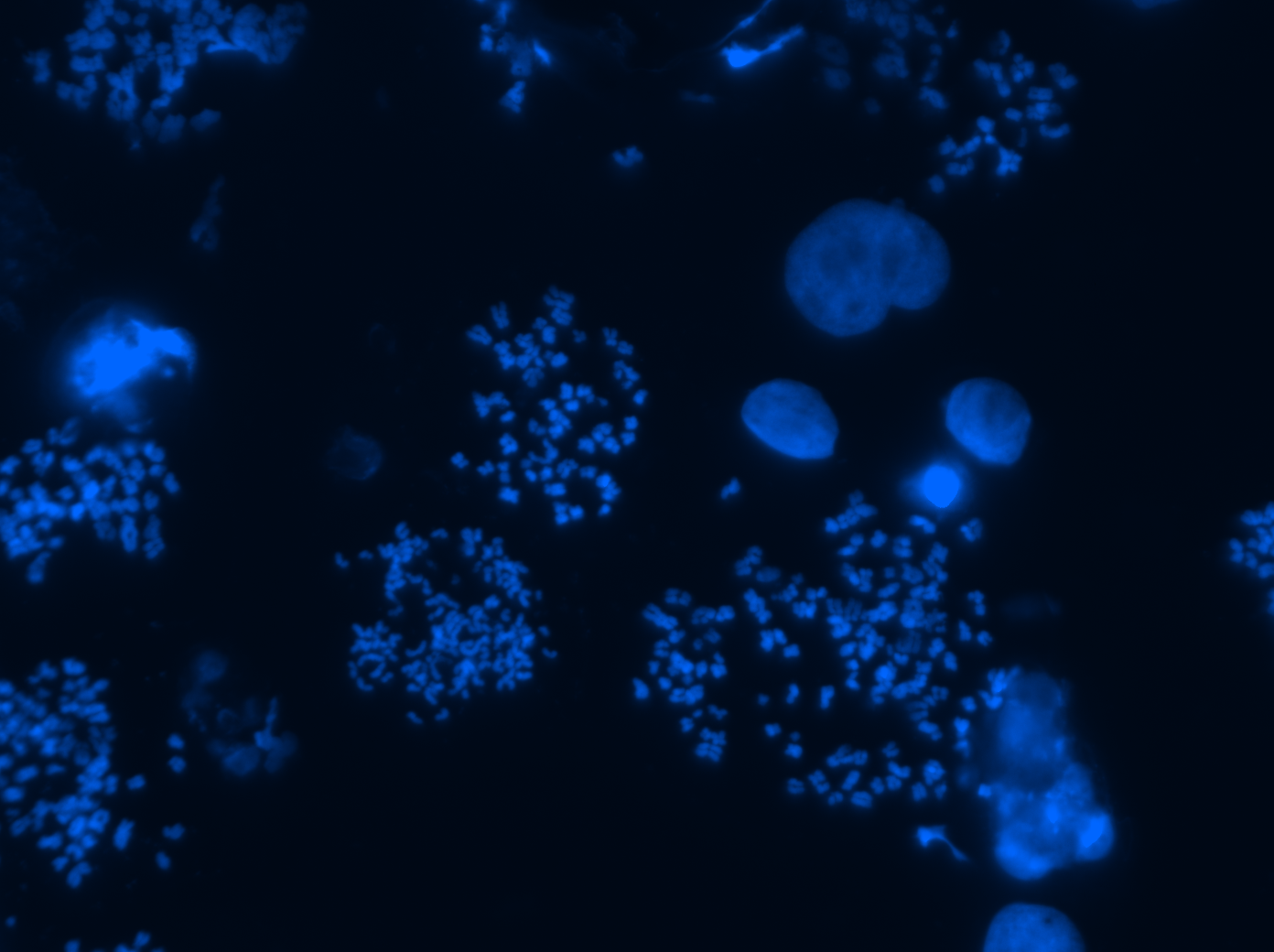

Supplement: Supplementary file 15 — Source data Fig. 9 [file 44318_2025_465_MOESM15_ESM.zip › EMBOJ-2025-120195-Figure 9-Source data/Figure 9/9B/Haspin KO-siATRX #3 DNA.tif]

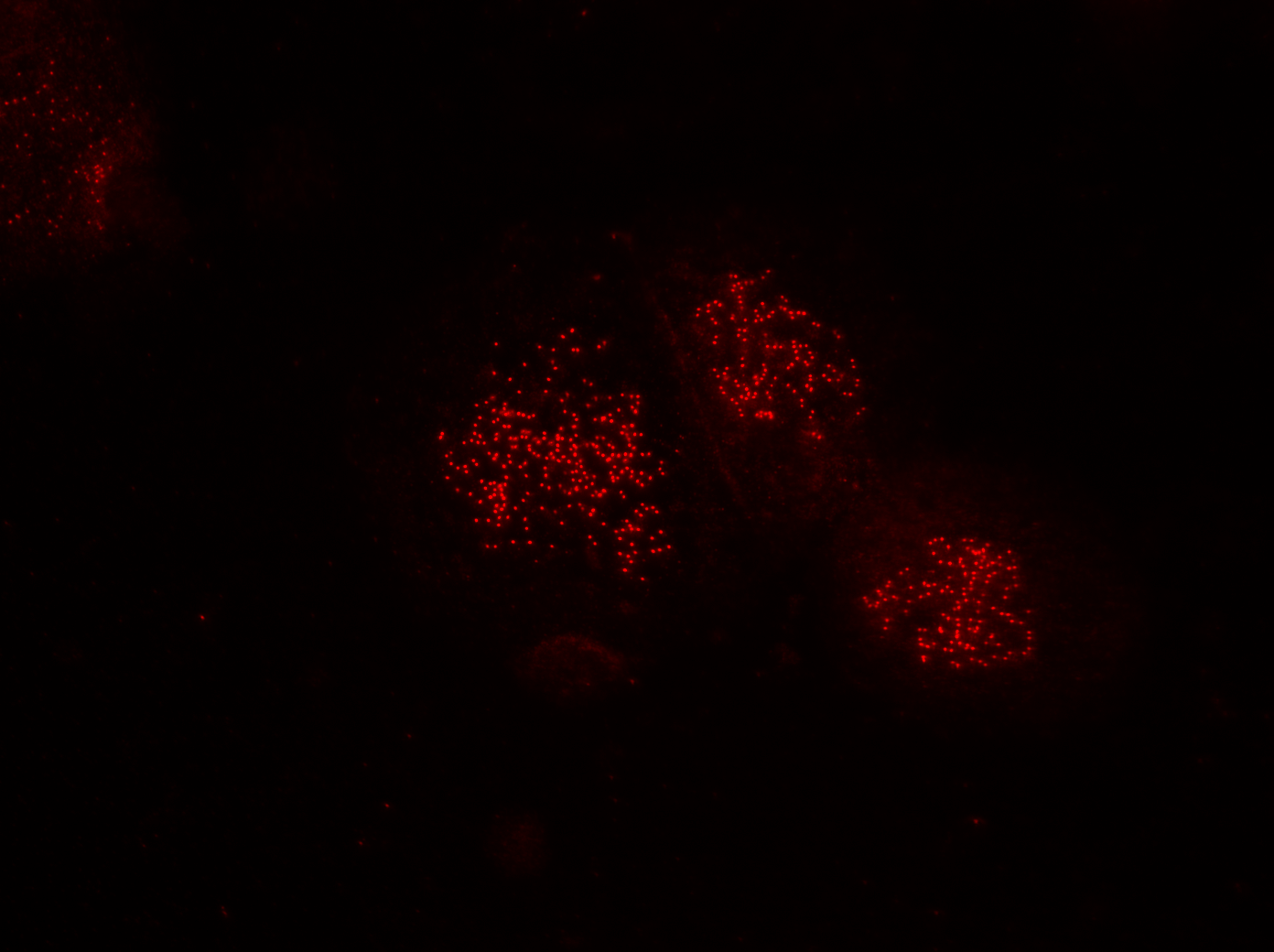

Supplement: Supplementary file 15 — Source data Fig. 9 [file 44318_2025_465_MOESM15_ESM.zip › EMBOJ-2025-120195-Figure 9-Source data/Figure 9/9B/Haspin-KO-siATRX#1 CENP-C.tif]

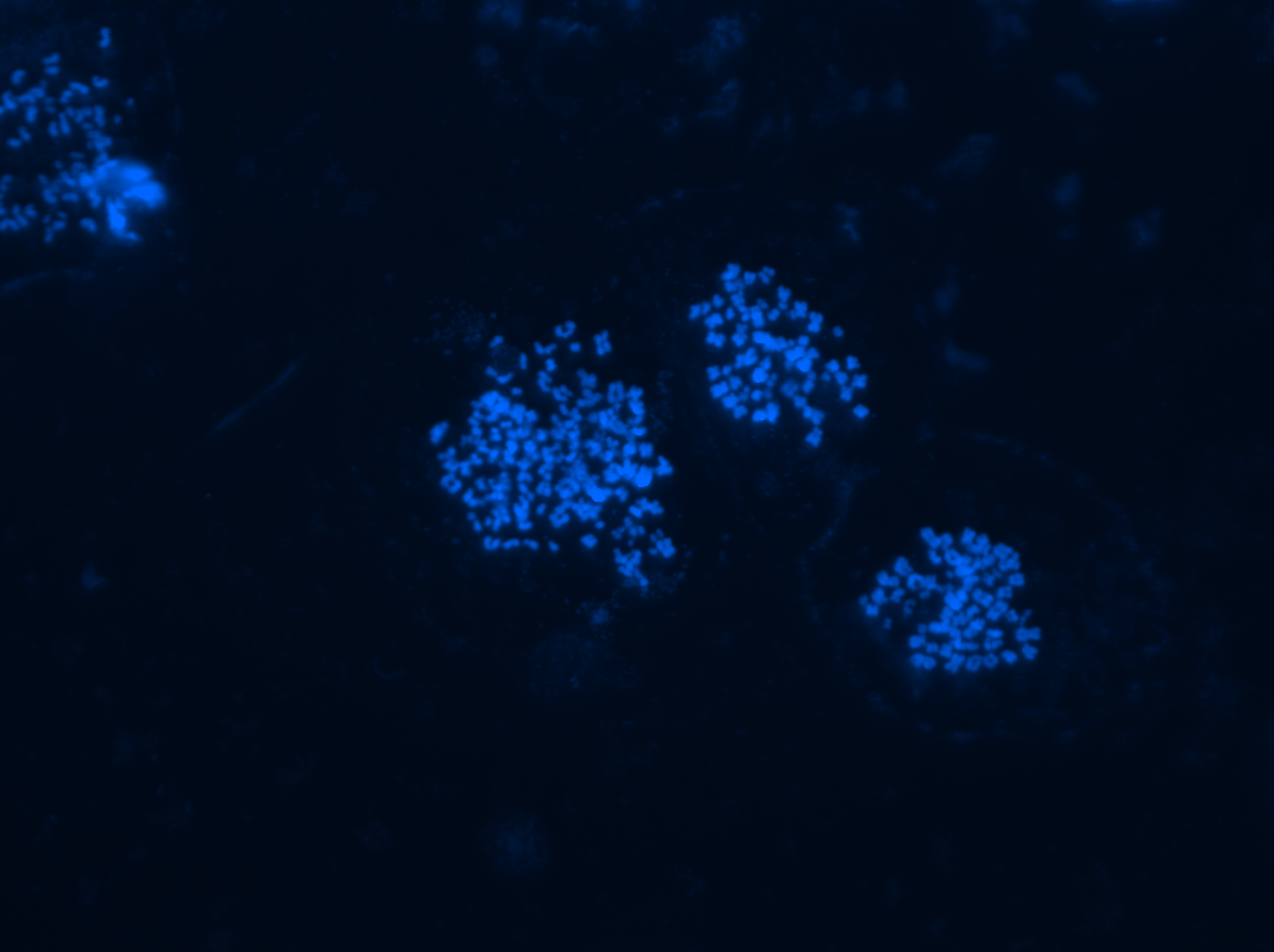

Supplement: Supplementary file 15 — Source data Fig. 9 [file 44318_2025_465_MOESM15_ESM.zip › EMBOJ-2025-120195-Figure 9-Source data/Figure 9/9B/Haspin-KO-siATRX#1 DNA.tif]

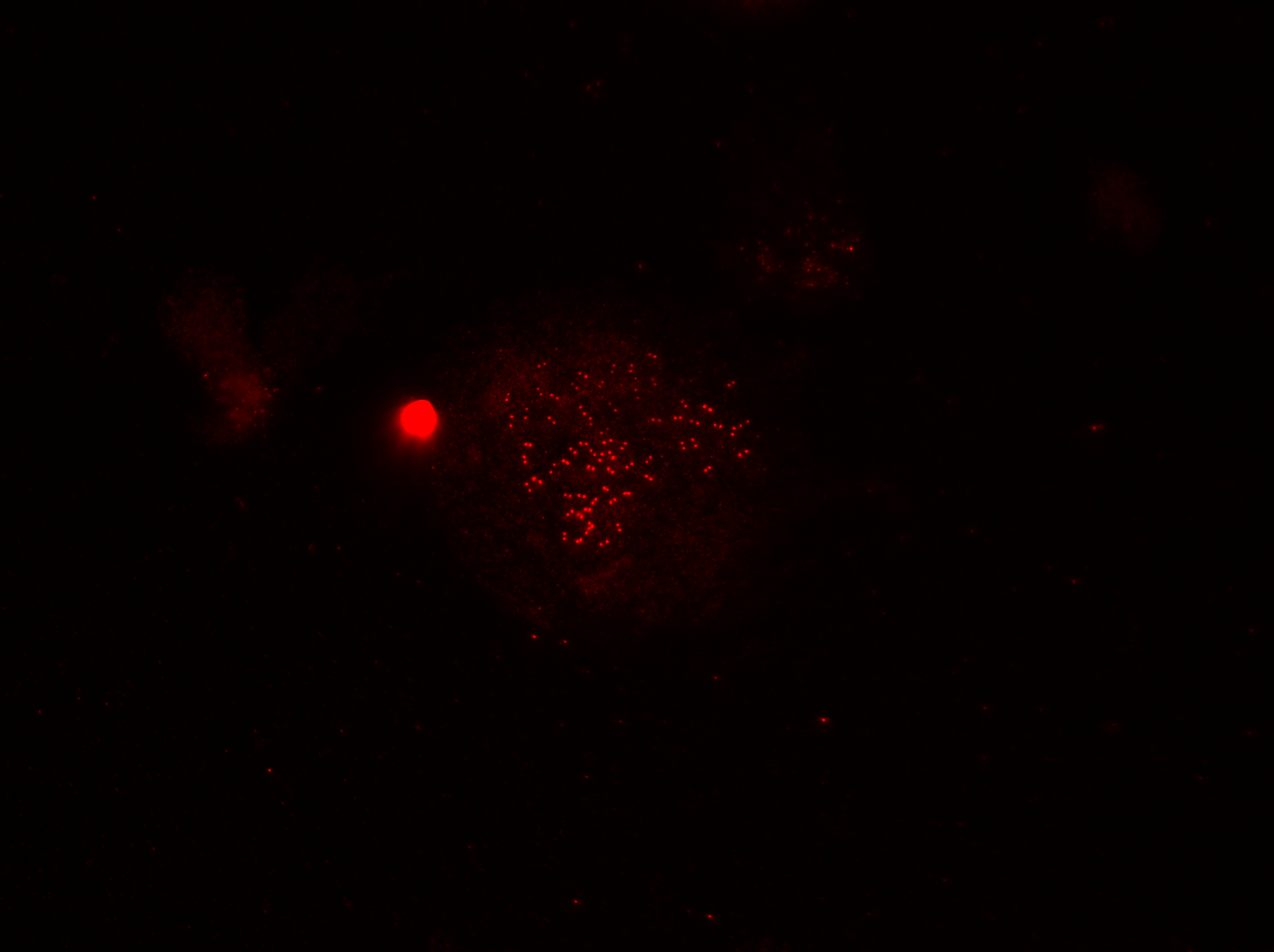

Supplement: Supplementary file 15 — Source data Fig. 9 [file 44318_2025_465_MOESM15_ESM.zip › EMBOJ-2025-120195-Figure 9-Source data/Figure 9/9B/Haspin-siControl CENP-C.tif]

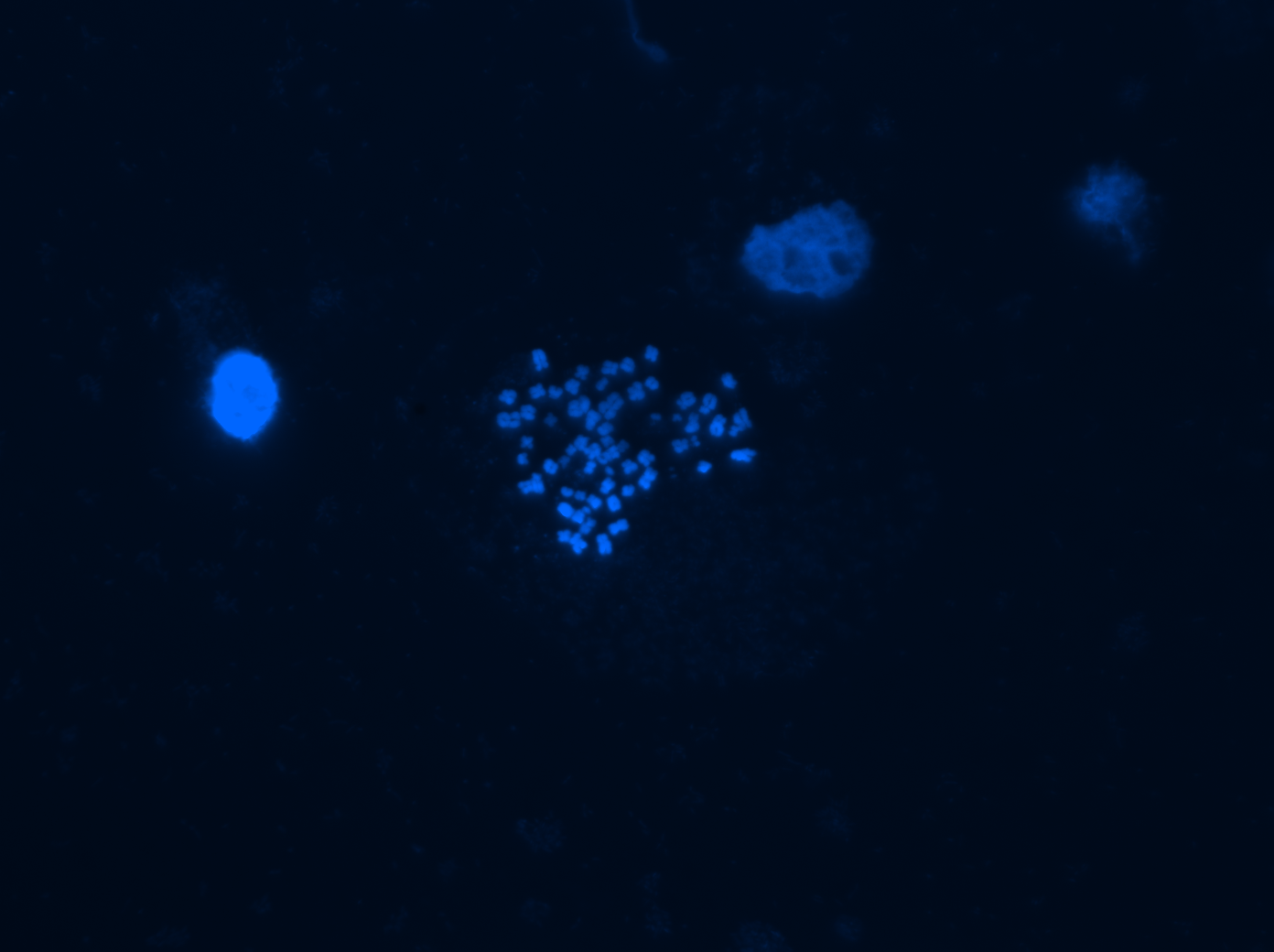

Supplement: Supplementary file 15 — Source data Fig. 9 [file 44318_2025_465_MOESM15_ESM.zip › EMBOJ-2025-120195-Figure 9-Source data/Figure 9/9B/Haspin-siControl DNA.tif]

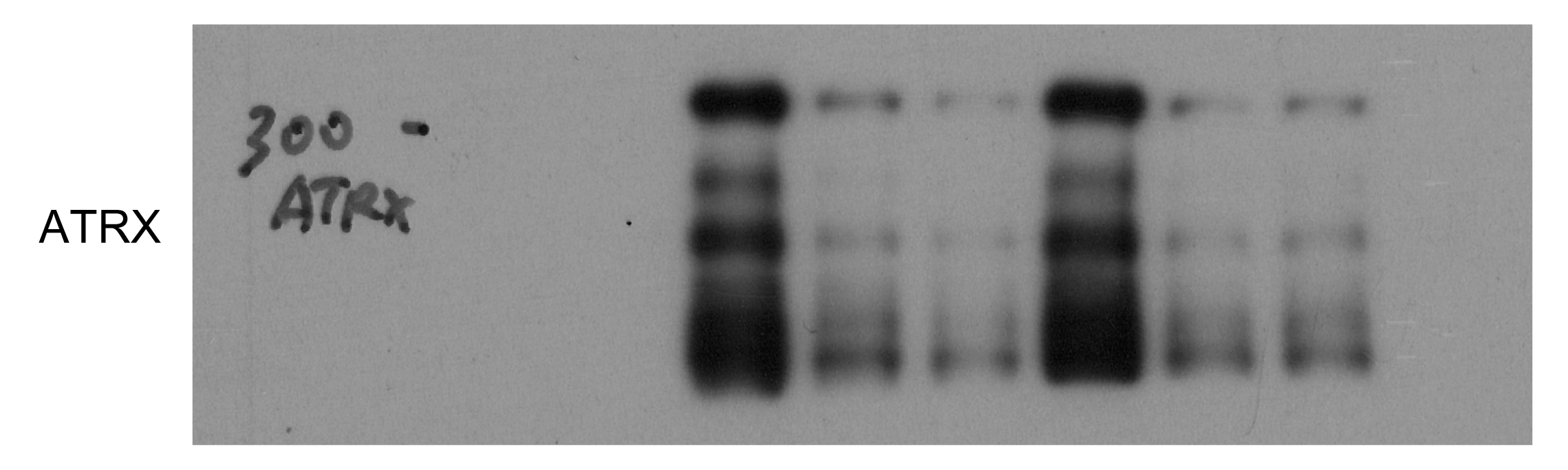

Supplement: Supplementary file 15 — Source data Fig. 9 [file 44318_2025_465_MOESM15_ESM.zip › EMBOJ-2025-120195-Figure 9-Source data/Figure 9/9C/western ATRX.tif]

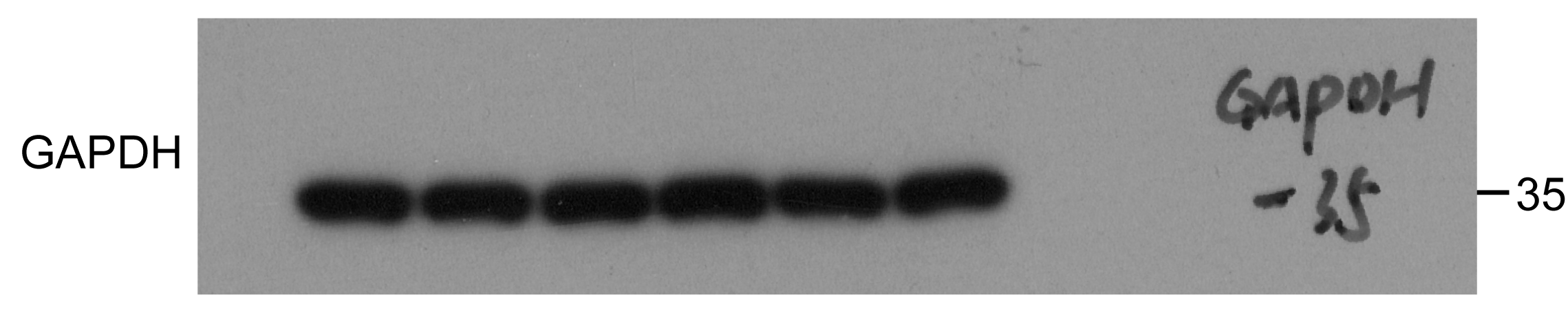

Supplement: Supplementary file 15 — Source data Fig. 9 [file 44318_2025_465_MOESM15_ESM.zip › EMBOJ-2025-120195-Figure 9-Source data/Figure 9/9C/western GAPDH.tif]
