# Supplementary material for: A chromatin-remodeling-independent role for ATRX in protecting centromeric cohesion
Source: EMBO J. 2025 May 28;44(14):4037–64. doi: 10.1038/s44318-025-00465-6 (PMC12264150; doi:10.1038/s44318-025-00465-6)
Supplement: Supplementary file 16 — Expanded View Figures [file 44318_2025_465_MOESM16_ESM.pdf]

Expanded View Figures

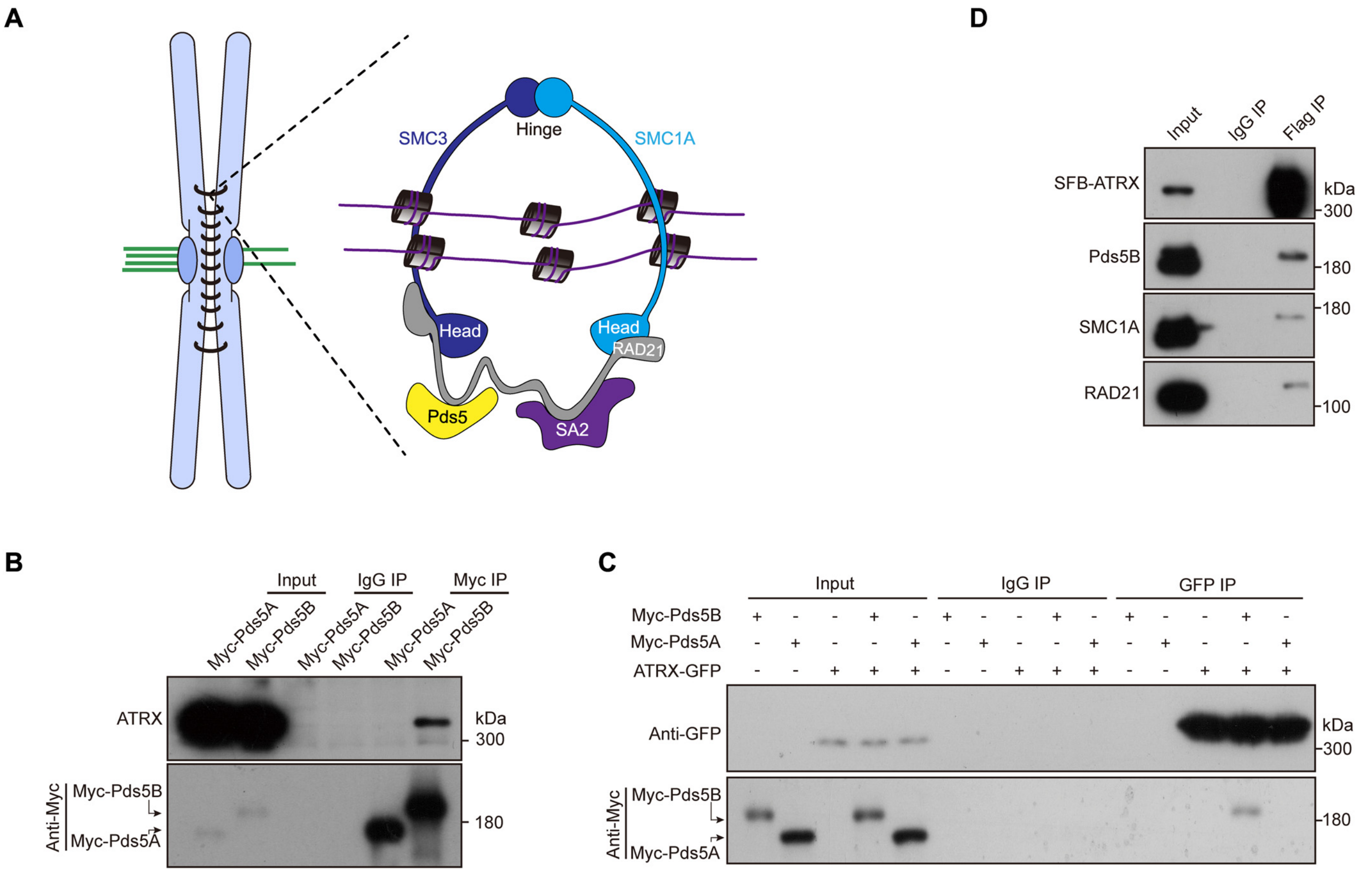

Figure EV1. A proteomic screen identifies ATRX as a Pds5B-associated factor.

(**A**) Schematic diagram illustrating sister chromatid cohesion mediated by the ring-shaped cohesin complex. (**B**) Nocodazole-arrested mitotic HeLa cells stably expressing Myc-Pds5A or Myc-Pds5B were immunoprecipitated using anti-Myc beads or control IgG, followed by immunoblotting for ATRX and the Myc tag. (**C**) HEK-293T cells co-expressing ATRX-GFP with Myc-Pds5A or Myc-Pds5B were immunoprecipitated using anti-GFP beads or control IgG, followed by immunoblotting for GFP and the Myc tag. (**D**) HEK-293T cells expressing SFB-ATRX were immunoprecipitated using anti-Flag beads or control IgG, followed by immunoblotting for the Flag tag, Pds5B, SMC1A, and RAD21.

© The Author(s)

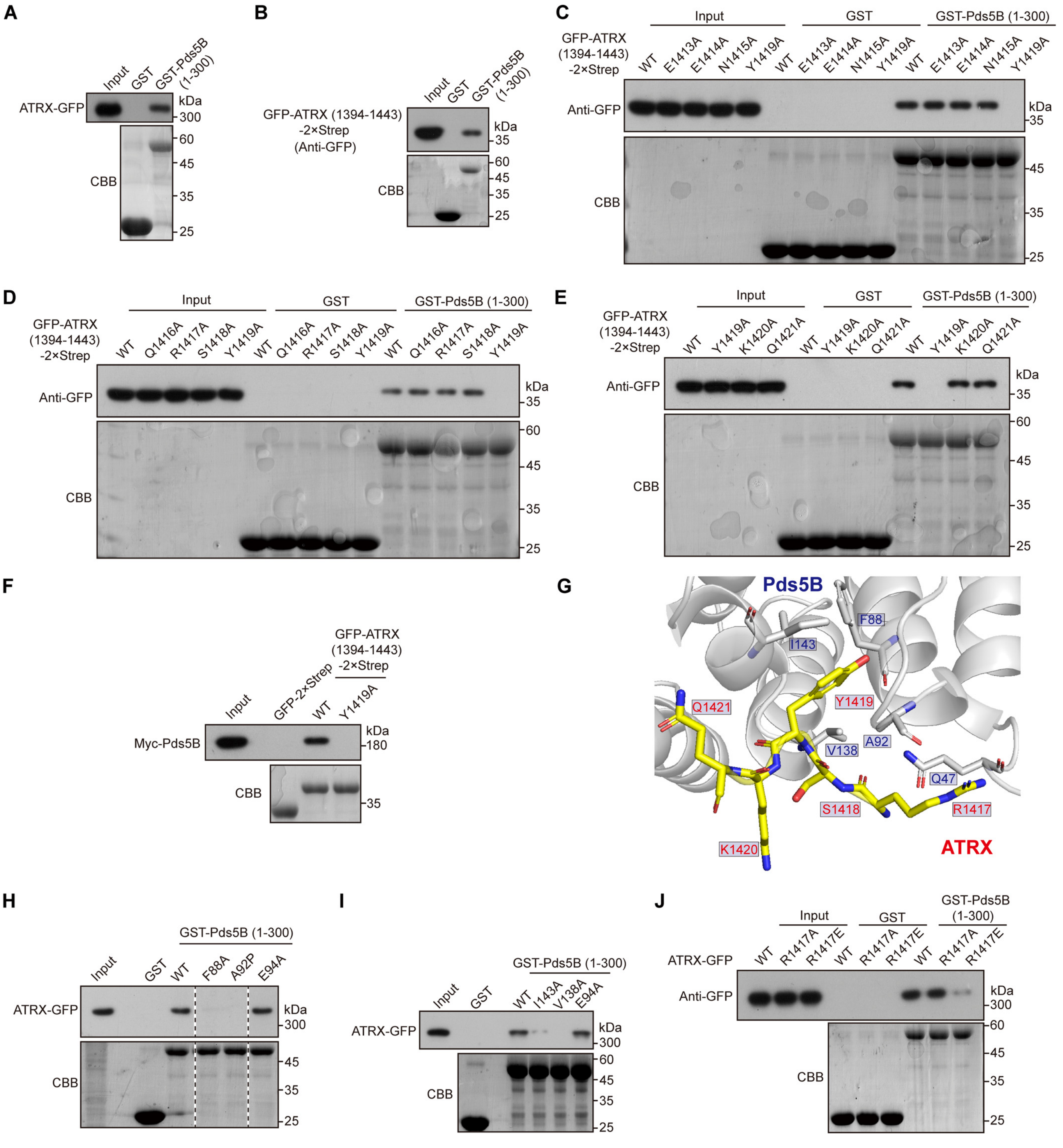

Figure EV2. Mapping the molecular interface between ATRX and Pds5B.

(**A**) HEK-293T cells expressing ATRX-GFP were subjected to pull-down using GST or GST-Pds5B (1–300), followed by immunoblotting for GFP and CBB staining. (**B–E**) Recombinant GFP-ATRX (1394–1443)-2xStrep (wild-type or mutants) proteins were subjected to pull-down using GST or GST-Pds5B (1–300), followed by immunoblotting for GFP and CBB staining. (**F**) HEK-293T cells expressing Myc-Pds5B were subjected to pull-down using GFP-2xStrep or GFP-ATRX (1394–1443)-2xStrep (WT or Y1419A), followed by immunoblotting for the Myc tag, and CBB staining. (**G**) Cartoon presentation of the structure for ATRX (RSYKQ) binding to Pds5B (1–300). ATRX and Pds5B residues are shown in yellow and gray, respectively. The binding details of ATRX residues R1417, S1418, Y1419, K1420, and Q1421 with Pds5B residues Q47, F88, A92, V138, and I143 are depicted. (**H**, **I**) HEK-293T cells expressing ATRX-GFP were subjected to pull-down using GST or GST-Pds5B (1–300) (WT or mutants), followed by immunoblotting for GFP and CBB staining. (**J**) HEK-293T cells expressing ATRX-GFP (WT, R1417A or R1417E) were subjected to pull-down using GST or GST-Pds5B (1–300), followed by immunoblotting for GFP and CBB staining.

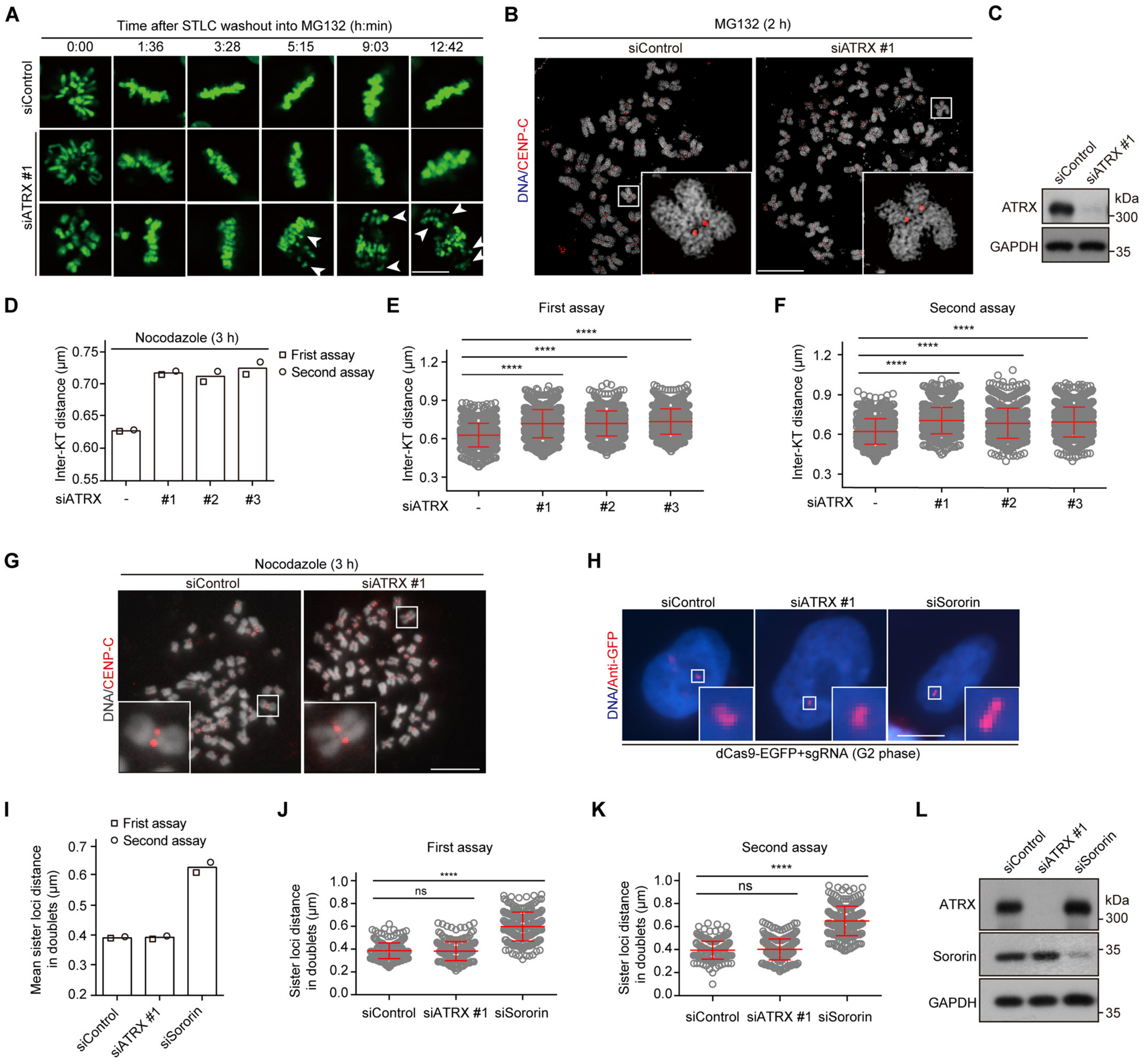

Figure EV3. ATRX promotes centromeric cohesion and prevents chromosome missegregation.

(**A**) HeLa cells stably expressing H2B-GFP were transfected with control or ATRX siRNA, followed by live imaging of mitosis progression. Selected movie frames are shown. Time is indicated in hours: minutes. Arrows point to scattering chromosomes. Related to Fig. 3D. (**B, C**) HeLa cells were transfected with control or ATRX siRNA. Forty-eight hours post-transfection, cells were treated with MG132 for 2 h, and mitotic chromosome spreads were stained for CENP-C and DAPI. Representative super-resolution images are shown (**B**). Cell lysates were analyzed by immunoblotting for ATRX and GAPDH (**C**). (**D–G**) HeLa cells transfected as described above were treated with nocodazole for 3 h, and mitotic chromosome spreads were stained for CENP-C and DAPI. The inter-KT distance was measured on more than 2000 chromosomes from 95 cells per condition across two independent experiments, and the means and ranges are plotted (**D**). Inter-KT distances from two individual experiments are shown. p values from left to right: ****$p < 1.00E-15$, ****$p < 1.00E-15$, ****$p < 1.00E-15$ (**E**). ****$p < 1.00E-15$, ****$p < 1.00E-15$, ****$p < 1.00E-15$ (**F**). Representative images are shown (**G**). (**H–L**) HeLa cells were transfected with the indicated siRNAs, co-transfected with NLS-dCas9-EGFP-expressing vector and the sgRNA-expressing vector, and synchronized using double thymidine treatment. Eight hours after release from thymidine, cells were stained for GFP and DAPI. Representative images are shown (**H**). The sister loci distance was measured in more than 450 cells per condition across two independent experiments, and the means and ranges are plotted (**I**). Sister loci distances from two individual experiments are shown. p values from left to right: ns $p = 6.12E-01$, ****$p < 1.00E-15$ (**J**). ns $p = 3.95E-01$, ****$p < 1.00E-15$ (**K**). Cell lysates were analyzed by immunoblotting for ATRX, Sororin, and GAPDH (**L**). Data information: Statistics were performed using unpaired Student's t-test (**E, F, J, K**). Means and SDs are shown (**E, F, J, K**). ns, no significance. Scale bars, 10 µm (**A, B, G, H**).

 © The Author(s)

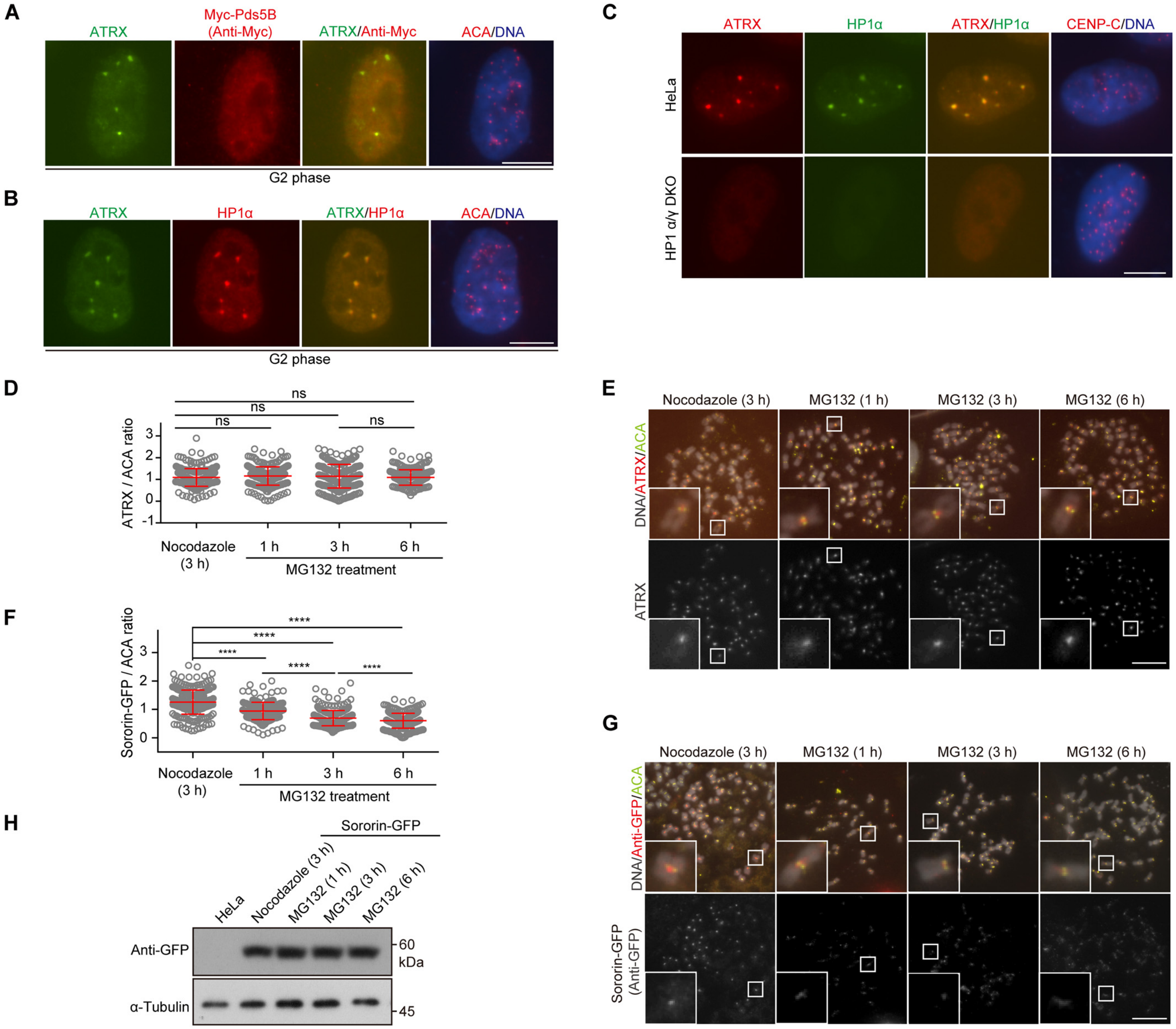

Figure EV4. ATRX is enriched at mitotic centromeres in an HP1-dependent manner.

(**A**) HeLa cells stably expressing Myc-Pds5B were synchronized using double thymidine treatment. Eight hours after release from thymidine, cells were stained for ATRX, the Myc tag, ACA, and DAPI. Representative images are shown. (**B**) HeLa cells were synchronized in G2 phase as described above and stained for ATRX, HP1α, ACA, and DAPI. Representative images are shown. (**C**) HeLa and HP1α/γ DKO cells were stained for ATRX, HP1α, CENP-C, and DAPI. Representative images are shown. (**D, E**) HeLa cells were treated with nocodazole for 3 h or with MG132 for 1, 3, and 6 h, and mitotic chromosome spreads were stained for ATRX, ACA, and DAPI. The fluorescence intensity ratio of ATRX to ACA was quantified on more than 230 chromosomes from 15–20 cells. *p* values from left to right: ns $p = 6.59E-02$, ns $p = 1.91E-01$, ns $p = 9.35E-01$, ns $p = 2.07E-01$ (**D**). Representative images are shown (**E**). (**F–H**) HeLa cells stably expressing Sororin-GFP were treated with nocodazole for 3 h or with MG132 for 1, 3, and 6 h, and mitotic chromosome spreads were stained for GFP, ACA, and DAPI. The fluorescence intensity ratio of Sororin-GFP to ACA was quantified on more than 195 chromosomes from 15 to 20 cells. *p* values from left to right: ****$p < 1E-15$, ****$p < 1E-15$, ****$p < 1E-15$, ****$p < 1E-15$ (**F**). Representative images are shown (**G**). Cell lysates were analyzed by immunoblotting for GFP and α-Tubulin (**H**). Data information: Statistics were performed using unpaired Student's t-test (**D, F**). Means and SDs are shown (**D, F**). N.S., no significance. Scale bars, 10 μm (**A, B, C, E, G**).

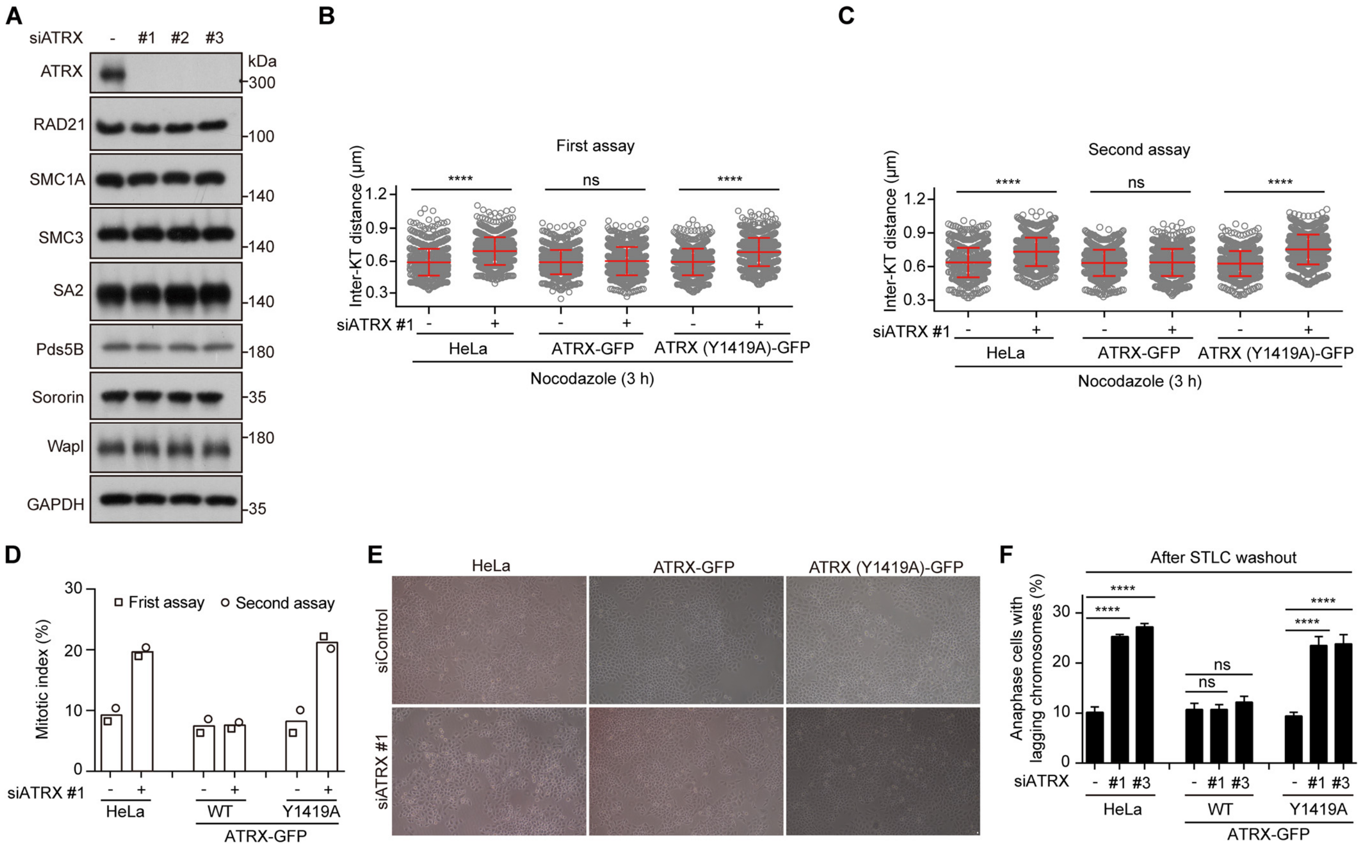

Figure EV5. ATRX maintains centromeric cohesion through interaction with Pds5B.

(**A**) HeLa cells were transfected with control or ATRX siRNAs. Forty-eight hours post-transfection, cells were analyzed by immunoblotting for ATRX, RAD21, SMC1A, SMC3, SA2, Pds5B, Sororin, Wapl, and GAPDH. (**B**, **C**) HeLa and HeLa cells stably expressing ATRX-GFP (WT or Y1419A) were transfected as described above, treated with nocodazole for 3 h, and mitotic chromosome spreads were stained for GFP, ACA, and DAPI. The inter-KT distance was measured on more than 1750 chromosomes from 80 cells per condition across two independent experiments. *p* values from left to right: *****p* < 1.00E−15. ns *p* = 1.01E−01, *****p* < 1.00E−15 (**B**). *****p* < 1.00E−15 (**J**). ns *p* = 5.10E−01, *****p* < 1.00E−15 (**C**). Related to Fig. 5D. (**D**, **E**) HeLa and HeLa cells stably expressing ATRX-GFP (WT or Y1419A) were transfected with control or ATRX siRNA. Forty-eight hours post-transfection, cells were subjected to mitotic index analysis. The mitotic index was determined from more than 940 cells per condition across two independent experiments (**D**). Representative images of cells are shown, with round cells classified as mitotic (**E**). (**F**) HeLa and HeLa cells stably expressing ATRX-GFP (WT or Y1419A) transfected as described above were treated with STLC for 5 h. Two hours after STLC release, cells were fixed and stained with DAPI. The percentage of anaphase cells with lagging chromosomes was quantified from more than 560 cells per condition across three independent experiments. *p* values from left to right: *****p* = 5.30E−05. *****p* = 5.00E−05, ns *p* = 9.92E−01, ns *p* = 2.09E−01. *****p* = 2.44E−04. *****p* = 2.29E−04. Data information: Statistics were performed using unpaired Student's t-test (**B**, **C**, **F**). Means and SDs are shown (**B**, **C**, **F**). ns, no significance. Scale bars, 10 μm (**E**).

 © The Author(s)
